# From 16S rDNA-seq count data to microbial interactions: A comprehensive benchmark of co-occurrence network inference methods

**DOI:** 10.1371/journal.pcbi.1014898

**Published:** 2026-10-29

**Authors:** Piero Mariotto, Matteo Baldan, Barbara Di Camillo, Giacomo Baruzzo

**Affiliations:** 1 Department of Information Engineering, University of Padova, Padova, Italy; 2 Department of Comparative Biomedicine and Food Science, University of Padova, Padova, Italy; 3 Padua Center of Network Medicine, University of Padova, Padova, Italy; Lawrence Berkeley National Lab (LBNL), UNITED STATES OF AMERICA

## Abstract

The study of the ecological interactions within microbial communities is crucial for gaining deeper insights into community ensemble and evolution, enabling targeted modification of the community’s functionality. Many inference approaches have been developed to derive ecological associations. Here, we focus on co-occurrence computational methods, in which associations are inferred from metagenomic abundance data by measuring the statistical dependence between species counts across different sampled populations. The limitations in culture-based experiments have hindered the ability to assess these methods’ validity, leading to scarce application in metagenomics studies. To provide a comprehensive assessment of network inference method performance, we benchmark 18 different computational approaches across a broad spectrum of experimental scenarios. We use synthetic 16S rDNA-seq count data generated from known underlying interaction networks, enabling systematic and controlled evaluation. The benchmark spans scenarios varying in numbers of species and samples, sample richness, sequencing depth, and interaction network structure. We evaluated the performance of the methods both in terms of accuracy of the inferred network, using various metrics from binary classification theory and metrics coming from network science, and in terms of computational burden. We found that methods have valid results only in a restricted, and often unrealistic, field of application, whereas their effectiveness diminishes with more realistic simulation parameters. Our findings highlight the limits of the co-occurrence-based approach and the need for further development in microbial ecological association inference.

## Author summary

The microbiota is the collection of microorganisms that reside within a given ecological environment or niche. Numerous studies have demonstrated that the microbiota is involved in a wide range of biological processes that affect the health or balance of the host organism or ecosystem. Recent advances in high-throughput sequencing technologies, together with progress in bioinformatic and computational techniques, have enabled new discoveries and deeper understanding in this field. One of the most promising analyses in this landscape is the inference of the community interaction network. Researchers are therefore faced with the challenge of selecting appropriate methods from a large and diverse set of available approaches. To address this, we performed a comprehensive benchmark of established co-occurrence microbial network inference tools using simulated data. The use of simulated data was necessary due to the lack of reliable ground-truth references from culture-based experiments, which has limited systematic evaluation of method performance. Our work aims to assess the general applicability of network inference methods and to support researchers in the choice of the best methods available.

## Introduction

Microbes are everywhere “*but the environment selects*”, as stated by Baas Becking [1], and in a given ecological niche they form a community [2], often denoted as microbiota. Within this community, microorganisms interact with the environment and with each other. These interactions, which can have various forms like cross-feeding, competition or horizontal gene transfer, have a major impact on the evolution of the community and play a major role in determining its functionality [3–7], and can be conceptualised as an interaction network.

The study of these communities has garnered much interest as more and more evidence points to their impact on various ecosystems like the soil [8], plants [9], oceans [10] and the human body. In particular, in humans, symbiotic microbial communities are essential to maintain host health [11] while altered, dysbiotic, communities have been linked to various diseases like cardiovascular diseases [12], inflammatory bowel disease [13], vaginosis [14,15], HIV [16], cancer [17] and diabetes [18,19].

The advent of Next Generation Sequencing (NGS), in particular, 16S rRNA gene targeted sequencing (16S rDNA-seq), has allowed the study of bacterial communities to reach a level previously not possible with culture-based experiments [20], leading to the generation of ever-growing amounts of data on microbial community composition. Many insights can be obtained by analysing these data with various techniques, like alpha and beta diversity or differential abundance analysis [21–26]. Recently, much attention has been devoted to identifying microbial interactions, mainly to gain insights into the community functionality, and thus enable targeted modification of the community to obtain a desired outcome. There are many approaches to derive a microbial community interaction network depending on the available sequencing data. Metagenome-scale metabolic network reconstruction (MMNR) offers a mechanistic approach, using annotated genomic data to reconstruct the metabolic pathways of individual taxa and Constraint-Based Modelling to obtain a community level interaction network [27,28]. Alternatively, when high-resolution longitudinal data are available, generalised Lotka-Volterra (gLV) models can be fitted to dynamic population fluctuations to mathematically infer directed causal interaction coefficients between species over time [29]. Another approach to identify microbial interactions is to infer them from species co-occurrence in metagenomics data [25,30]. In the co-occurrence approach, starting from a count matrix containing the observed count of each species in each sample, obtained from a 16S rDNA-seq experiment, an association network is inferred by determining the statistical dependence between the species counts. While MMNR and gLVs provide valuable insight into microbial dynamics and metabolic dependencies, their application is strictly constrained by their data requirements. In particular, gLV models require high-resolution, longitudinal time-series data to accurately estimate intrinsic growth rates and interaction coefficients, whereas MMNR requires comprehensive genomic sequencing and highly curated biochemical pathways. In contrast, co-occurrence methods only rely on cross-sectional microbial abundance profiles, which can be generated using inexpensive, consolidated technologies like amplicon sequencing. Given the wide-spread availability of cross-sectional microbial abundance profiles, co-occurrence methods have often been used to infer microbial associations and to generate ecological hypotheses. For this reason, in this work we focus on the co-occurrence approach, and when using more general terms, like inference or reverse engineering methods, we refer only to co-occurrence-based methods.

Even if promising, the inference of microbial interactions from 16S rDNA-seq data is no trivial task as these data are plagued by a number of well-known issues like the *curse of dimensionality*, compositionality [31], batch effects [32] and missing data / zero-inflation [33,34]. To solve part of these issues, several co-occurrence-based methods have been proposed during the years.

The simplest approach consists in measuring the pair-wise associations between species. Different metrics can be used to measure different types of associations, including Pearson Correlation (linear), Spearman Correlation (monotonic), Bray-Curtis dissimilarity, and Maximal Information Coefficient (MIC) [35] for non-linear associations. These metrics were often used in the past to infer microbial interaction, due to their simplicity and intuitiveness, especially right after the advent of NGS. However, these metrics are not directly applicable to compositional data (like 16S rDNA-seq data), as the closure operation introduces spurious correlations between variables with no biological significance [36].

To avoid this limitation, various compositionally-aware methodologies have been developed, from the log-ratio-based Proportionality metric [37] to more complex methods like SparCC [38], where the basis-correlations are estimated in an iterative procedure from the log-ratio transformed compositions. As SparCC is computationally expensive due to its iterative approach, new methods, like CCLasso [39], were developed based on the $\ell_{1}$-norm penalised estimation of the basis correlation matrix.

However, correlation-based methods, like those above, may introduce spurious indirect edges. For this reason, a host of methods has been developed to infer the association network by exploiting the concept of conditional dependence, i.e., two variables are considered dependent only if they add information about each other that other variables could not explain. One could use Pearson or Spearman Partial Correlation coefficients to measure direct associations or more sophisticated methods developed specifically for compositional data, such as SPIEC-EASI [40], SPRING [41], gCoda [42] and FlashWeave [43].

Other recently developed methods focus on modelling additional characteristics of 16S rDNA-seq count data, including sparsity or sequencing-related biases. HARMONIES [44] and COZINE [45] address zero inflation by fitting a Zero Inflated Negative Binomial Model and Hurdle Model respectively, before inferring the association network using conditional dependence. SECOM [46] instead aims to estimate correlation among species correcting for taxa and sample-specific biases introduced by the 16S rDNA-seq pipeline.

Despite the interest, and the undeniable advancements in the field, co-occurrence-based association network inference has seen scarce application in metagenomics studies and the results are often questioned [47]. The challenge in evaluating the methods’ performance arises from the absence of a large-scale biological ground truth against which inference results can be compared. The only viable alternative to evaluate the methods’ performance is the use of simulated data generated with a known ground truth. The simplest approach to generate simulated data is the use of parametric distributions and to consider the covariance matrix as a proxy for the ground truth interaction network [40,48,49]. Some have criticised this approach [50], arguing that it offers too drastic of a simplification of a complex system like microbial communities. Some valuable studies [50–52] used a system of non-linear equations describing population dynamics (extended Lotka-Volterra model) to simulate microbial communities abundance data. This approach is certainly a better option, primarily because it starts from the interaction network of the community and not from an a priori covariance structure. Although they represent a better alternative, these models are not optimal to predict microbial dynamics [53,54], as they assume that the dynamics of the community emerge from the sum of pairwise interactions among species, and that the sign and strength of these interactions are intrinsic properties of the species and thus assume a constant environment state [55].

In this work we present a comprehensive benchmark of existing network inference methods to assess their applicability in the microbial ecology field. Our contribution, with respect to previous comparative studies [48–52,56], consists in (see also Table B in S3 File):

- the inclusion of the largest selection of network inference methods (18 in total).
- the analysis of a broad range of scenarios encompassing diverse data characteristics (e.g., sample size, network structure, species richness, library size, interaction type, and sample diversity), including several factors not examined in previous studies.
- the evaluation of method performance using a comprehensive set of metrics, many of which have not been employed in previous studies, including binary classification measures (Precision, Recall, and Matthews correlation coefficient (MCC)), as well as network science metrics such as graph topological indices and centrality measures.
- the use of a newly developed simulator [57] that does not rely on pairwise interaction models, thereby reducing unrealistic oversimplifications in the generated data.
- the assessment of computational burden, an aspect not addressed in previous studies.

In order to ensure reproducibility of the results, we make publicly and freely available the synthetic datasets and results files (https://zenodo.org/records/23470419) along with the code used to generate and analyse them (https://gitlab.com/sysbiobig/net_inf_bench), and a Singularity container with all tested methods installed on it.

## Materials and methods

In order to facilitate the reading, we first introduce terms and concepts that will be widely used in the following sections. Matrices are referred to with bold capital letters, whereas random variables are referred to with lowercase letters (bold lowercase letters for random vectors). Let $𝐖\in {\mathbb{N}}^{p\times n}$ denote the real abundance of *p* species in *n* samples. We indicate as $𝐗(𝐖)\in {\mathbb{N}}^{p\times n}$ the 16S rDNA-seq count table, derived from the real abundance matrix **W**, in which the *p* rows indicate different species/features measured (e.g., OTUs, operational taxonomic units, or ASVs, amplicon sequence variants) and the *n* columns refer to the various sequenced samples; for ease of notation we will often refer to **X**(**W**) simply as **X**. With indices i,j∈{1,...,p} we will refer to two generic species and with ${𝐗}_{i\cdot },{𝐗}_{j\cdot }$ to two rows of **X**, while with indices k,o∈{1,...,n} we refer to two generic samples and with ${𝐗}_{\cdot k},{𝐗}_{\cdot o}$ to two columns of **X**. With $X_{ik}$ we refer to the counts of species *i* in sample *k*.

As mentioned previously, interactions within a microbial community can take various forms. In this work, we focus on only a subset of these possible interactions, the ones mediated by resources: cross-feeding and competition for resources. The microbial interaction network can be represented as a graph 𝒢(V,E) on a set of nodes *V*, representing the community’s species (with |*V*| = *p*), and a set of edges E={(i,j) | i,j∈V}⊆V×V represents the interactions between them. The graph can also be represented as an adjacency matrix $𝐀\in {\mathbb{R}}^{p\times p}$. In this study, we will consider undirected (thus $𝐀={𝐀}^{T}$) and for the majority unweighted graphs; **A** will contain only zeros and ones, with $A_{ij}=1$ indicating an edge between node *i* and node *j*, zero otherwise. With $\Sigma \in {\mathbb{R}}^{p\times p}$ we indicate the covariance matrix of the *p* species, with $𝐑\in {\mathbb{R}}^{p\times p}$ their correlation matrix, and with $\Omega =\Sigma^{-1}\in {\mathbb{R}}^{p\times p}$ their precision matrix.

### In silico data generation

To generate the synthetic datasets used in this study, we used N2SIMBA [57], a recently developed simulator that generates a 16S rDNA-seq data table $𝐗\in {\mathbb{N}}^{p\times n}$ from a known interaction network. N2SIMBA uses Community Simulator [58] (a Python implementation of the MiCRM model [55,59]) to simulate the evolution of a microbial community composed of *p* species of consumers competing for *m* resources. Following the MiCRM model, the species and resources dynamics are defined by a system of non-linear equations


$$\begin{array}{cc} \frac{dW_{i}}{dt} & =gW_{i}[\sum_{\alpha }(1-l)\omega \sigma (c_{i\alpha }R_{\alpha })-\eta ],\;\forall i\in \{1,...,p\} \end{array}$$ (1)

$$\begin{array}{cc} \frac{dR_{\alpha }}{dt} & =h(R_{\alpha })-\sum_{j}W_{j}\sigma (c_{j\alpha }R_{\alpha })+\sum_{j\beta }W_{j}\sigma (c_{j\beta }R_{\beta })\left[D_{\alpha \beta }l\right],\;\forall \alpha \in \{1,...,m\} \end{array}$$ (2)

where *g* is the conversion from energy flux to growth rate, *l* is the fraction of input energy leaked back to the environment, ω is the energy content of the resources, σ is the input mass flux, η is the basal maintenance cost and *h* is the external supply of resources. A detailed list of the parameter values used in the simulations is available in S1 File, and for a detailed explanation of Eq (1–2) we invite the reader to consult the original papers [55,58]. Of particular importance are the matrices $𝐂\in {\mathbb{R}}^{p\times m}$ and $𝐃\in {\mathbb{R}}^{m\times m}$. In matrix **C** each element $c_{i\alpha }$ determines if species *i* feeds off of resource α, while matrix **D** describes how the resources are converted by species metabolism, e.g., $D_{\alpha \beta }$ denotes the fraction of resource $R_{\beta }$ that is converted to $R_{\alpha }$ with $\sum D_{\cdot \beta }=1$. Thus, the two matrices encode the interaction network between species in the community, that is if $c_{i,\alpha }\neq 0,D_{\alpha \beta }\neq 0,c_{j,\beta }\neq 0$ then species *j* is beneficial to species *i*, while, on the contrary, if $c_{i,\gamma }\neq 0,c_{j,\gamma }\neq 0$ the two species will compete for the same resource $R_{\gamma }$. In N2SIMBA, the **C** and **D** matrices are generated univocally, following a user-defined interaction network (for details, we invite the reader to consult the original paper [57]). The user can provide the interaction network as an adjacency matrix or it can be generated using a graph generation algorithm. Since the interaction network topology of real communities is not known, we used 3 different graph generation algorithms: Erdős–Rényi (ER) model [60], Barabási–Albert (BA) model [61] and Watts-Strogatz (WS) model [62]. Each algorithm takes as input the number of nodes/species *p* in the graph and a parameter (here generically indicated as θ) that has different meanings depending on the algorithm, but ultimately determines the graph density ρ(𝒢) (for more details, refer to S1 File). As the density of real microbial ecological networks is not known and may vary across different ecological niches, we simulated datasets with various levels of interaction network density (from 2% up to 15%). The parameters used by the three algorithms to generate graphs with different densities are reported in Table A in S1 File.

Given the adjacency matrix **A**, N2SIMBA allows users to specify the negative interaction ratio $r_{neg}$; a set of edges $E_{neg}$, with $|E_{neg}|=|E|\cdot r_{neg}$, will be selected at random and the corresponding element in the adjacency matrix will be set as $A_{ij}=-1\;\forall (i,j)\in E_{neg}$. Negative interactions are intended as competition for the same resource and not predation, since MiCRM does not model predation. Once all the model parameters are initialised, the initial abundances of each species are randomly selected from a preset of real sequencing data [63]. The community is simulated until a steady state is reached. Then, from the steady-state community, *n* communities are derived by perturbing the steady-state resources in *n* different ways, simulating the different environmental conditions found between samples in real experiments. How each resource is perturbed and the magnitude of the perturbations allow us to generate datasets with different levels of diversity between samples, where for diversity we intend the well-known ecological concept of β-diversity, i.e., the relative differences in species composition between habitats [64]. In this study, we used three different perturbation strategies, varying the amount of available resources fed to the community’s environment (see S1 File), to obtain datasets with low ($\overset{―}{\beta }≅0.515$), medium ($\overset{―}{\beta }≅0.667$) and high ($\overset{―}{\beta }≅0.920$) median β-diversity between samples (see Fig E in S1 File). After the perturbation, the community is simulated until it reaches a new steady state, obtaining an abundance matrix $𝐖\in {\mathbb{R}}^{p\times n}$.

Finally, the steady-state abundances are sampled following a Multivariate Hypergeometric Model [63] to obtain the output 16S rDNA-seq simulated count matrix $𝐗\in {\mathbb{N}}^{p\times n}$:


$$\begin{array}{c} {𝐗}_{\cdot k}\sim MHG(L_{k},\overset{{𝐘}_{\cdot k}}{\overbrace{[Y_{1k},...,Y_{pk}{]}^{T}}}) \end{array}$$ (3)

where $L_{k}$ is the number of extractions performed, i.e., the library size of sample *k*, and ${𝐘}_{\cdot k}$ are the rescaled steady-state abundances ${𝐘}_{\cdot k}=\frac{{𝐖}_{\cdot k}}{\sum {𝐖}_{\cdot k}}\cdot const$. Rescaling to a fixed sample count simulates the selective amplification of the 16S rRNA gene performed during library preparation in a real experiment. To obtain the library size $L_{k}$ the simulator rescales the original library sizes *L* from the preset [63] by a scale factor composed of two elements


$$\begin{array}{c} L_{k}=L\cdot \frac{\sum_{i}^{p}W_{i}^{0}}{W_{tot}}\cdot K \end{array}$$ (4)

where $W_{i}^{0}$ are the initial abundances of the species (at *t* = 0), $W_{tot}$ is the sum of all the species abundances in the preset and *K* is a user-defined scale factor. By reducing *K* < 100% the number of extractions will be smaller, mimicking reduced library sizes, and thus the output 16S rDNA-seq table will have stronger compositional effects and contain more zeros as rare species are not sampled.

### Simulated scenarios

Given the limited information in the literature on biological network characteristics, and consequently on how to set the corresponding simulation parameters, we conducted extensive simulations under different parameter configurations to generate a wide range of scenarios. This approach also allowed us to assess how changes in individual parameters affect network inference performance. Specifically, we evaluated the impact of the following factors on method performance:

- the number of samples (*n*) and library sizes (*K*), which are the only parameters directly controllable in real experiments and represent the primary cost drivers of 16S rDNA-seq studies. In practice, these parameters are often linked through sample multiplexing strategies, where increased multiplexing (i.e., higher number of samples) leads to reduced per-sample library sizes, resulting in sparser count matrices and stronger compositional effects.
- the number of species in the community (*p*) and the diversity of their composition in the samples ($\overset{―}{\beta }$), two characteristics that can vary substantially between ecological niches and experimental settings.
- the underlying interaction network model (ℳ), as well as more specific network characteristics such as the ratio of competitive interactions ($r_{neg}$) and the network density ρ(𝒢). Notably, low network density is a key assumption underlying most inference methods.

To distinguish the effect of each parameter on the simulated scenarios and consequently on the performance of the methods, we defined default values for most parameters and varied one parameter at a time while keeping the others fixed. This strategy resulted in a total of 189 distinct scenarios (parameter configurations). Two parameters were treated differently: the graph generation model (ℳ= ER, BA, WS) and the median β-diversity between samples ($\overset{―}{\beta }=$low, medium, high). For these parameters, no default values were defined; instead, all possible combinations were considered when varying the remaining parameters.

A detailed list of parameter values and defaults is provided in Table 1. For each of the 189 parameter configurations, we generated 50 independent simulations, resulting in a total of 9450 simulated 16S rDNA-seq count tables.

**Table 1:** Simulation parameters.

Parameter	Symbol	Values tested
Graph generation model	ℳ	ER, BA, WS
Diversity between samples	$\overset{―}{\beta }$	low, medium, high
Network densities	ρ(𝒢)	2%, **5%**, 8%, 10%, 15%
Number of samples	*n*	50, 75, **100**, 200, 300, 500, 1000
Number of species	*p*	**50**, 100, 150, 200
Negative interaction rate	$r_{neg}$	0, **0.05**, 0.1, 0.2
Library size fraction	*K*	**100%**, 10%, 2%, 1%, 0.5%

**Bold** entries are the default values used when varying other parameters (e.g., when generating datasets with different number of samples *n* = 50, 75, 100, 200, 300, 500, 1000, the other parameters are set to ρ(𝒢)=5%, *p* = 50, $r_{neg}=0.05$, *K* = 100%). All combinations of ℳ and $\overset{―}{\beta }$ were always considered.

To assess whether the simulated data reproduce key characteristics of real microbiome data, we compared them with six representative empirical datasets (see Table D in S1 File) examining: species abundance curves, mean-variance curves and mean-prevalence curves. The complete comparative evaluation is provided in Section 3 of S1 File. Overall, the simulations showed qualitative agreement with selected macroecological patterns observed in the empirical datasets, while also showing discrepancies in prevalence and representation of rare taxa. Simulations with low beta diversity, and some scenarios with medium beta diversity, tended to under-represent low-abundance, highly sparse taxa. However, the extreme zero inflation observed for some taxa in real datasets is often reduced in practice through standard prevalence filtering before network inference. When retained, such taxa can pose a substantial challenge for inference methods; accordingly, these simulated datasets may represent a somewhat simplified input for network inference benchmarking.

### Tested network inference methods

All standalone microbial associations inference methods present in previous comparative studies [48–52,56] were included in the benchmark, except for REBACCA [65] due to persistent runtime errors, including newly introduced methods, up to 13 inference tools. We divided the tools into two broad classes: unconditional dependence methods and conditional dependence methods. This classification will often be used to group the methods in the following sections. All methods were executed using their default parameter settings, except for the filtering parameters, where applicable. These filtering parameters were adjusted in a method-specific manner to prevent any filtering of the input data (i.e., the removal of columns or rows), thereby ensuring that all methods analysed exactly the same data (all custom values of the filtering parameters are highlighted in S2 File). Although this takes the methods out of their intended pipeline and may affect performance, it was necessary to ensure that the results remained comparable. We also conducted a preliminary sensitivity analysis to explore the effects of the parameters for some of the methods (Section 3 in S2 File), which did not highlight strong effects on the methods’ performance and was thus not explored further. In addition to the selected tools, we tested 5 basic metrics, often used to measure microbial associations, as a performance baseline, for a total of 18 inference techniques. For each method, we collected information on their relevance, i.e., the number of citations on Scopus and Web of Science, their publication year, the availability and maintenance of the software and the inference strategy implemented (Table 2).

**Table 2:** Network inference methods included in this benchmarking study.

Base methods				
**Method**	**Citations**	**Approach**	**Year**	**Software implementation**
Pearson		Linear pairwise metric		stats R package
Spearman		Monotonic pairwise metric		stats R package
Bray-Curtis		Non-linear pairwise metric		vegan R package available on CRAN
Partial Pearson		Conditional dependence		ppcor R package available on CRAN
Partial Spearman		Conditional dependence		ppcor R package available on CRAN
**Unconditional dependence methods**				
**Method**	**Citations**	**Approach**	**Year**	**Software implementation**
Proportionality [37]	193^‡^ - 205^†^	Compositional pairwise metric	2015	propr R package available at https://github.com/tpq/propr
SparCC [38]	1982^‡^ - 2015^†^	Compositional pairwise metric	2012	Part of the SpiecEasi R package available at https://github.com/zdk123/SpiecEasi
CCLasso [39]	138^‡^ - 150^†^	Lasso covariance estimation	2015	R code available at https://github.com/huayingfang/CCLasso
SECOM [46]	106^‡^ - 111^†^	Bias-aware linear metric	2022	Part of the ANCOMBC R package available at https://github.com/FrederickHuangLin/ANCOMBC
		Bias-aware non-linear metric		
**Conditional dependence methods**				
**Method**	**Citations**	**Approach**	**Year**	**Software implementation**
SPIEC-EASI [40]	1245^‡^ - 1243^†^	Neighbourhood selection (MB)	2015	SpiecEasi R package available at https://github.com/zdk123/SpiecEasi
		Precision matrix selection (Glasso)		
SPRING [41]	91^‡^ - 93^†^	Neighbourhood selection	2019	SPRING R package available at https://github.com/IrinaStatsLab/SPRING
gCoda [42]	40^‡^ - 42^†^	Precision matrix selection	2017	R code available at https://github.com/huayingfang/gCoda
FlashWeave [43]	114^‡^ - 112^†^	Partial correlation (PR)	2019	FlashWeave Julia package available at https://github.com/meringlab/FlashWeave.jl
		Mutual information (MI)		
HARMONIES [44]	25^‡^ - 27^†^	Zero Inflated	2020	HARMONIES R package available at https://github.com/shuangj00/HARMONIES
COZINE [45]	22^‡^ - 24^†^	Zero Inflated	2020	COZINE R package available at https://github.com/MinJinHa/COZINE

Citation numbers were obtained from ‡ Web of Science, † Scopus in April 2026.

#### Base methods.

We used Pearson correlation, Spearman correlation, Bray-Curtis dissimilarity, partial Pearson correlation and partial Spearman correlation to measure pairwise associations between species $\xi_{ij}$ in the 16S rDNA-seq count table. To implement these association scores on constrained data, a log-ratio transformation was applied to the simulated 16S rDNA-seq table (except for Bray-Curtis dissimilarity). A modified centred log-ratio clr_m_ was used to avoid the division by zero without adding a pseudocount; this transformation considers only the non-zero elements of each sample


$$\begin{array}{c} clr_{m}({𝐗}_{\cdot k})=\left\{\frac{X_{ik}}{g({𝐗}_{\cdot k})}\;|\;i\in I\right\}\cup \{X_{ik}\;|\;i\notin I\}\;with\;I=\{i\in \{1,...,p\}\;|\;X_{ik}\neq 0\} \end{array}$$

Note that applying the centred log-ratio transformation leads to a singular covariance matrix Σ [66]; thus, to obtain its inverse, the precision matrix Ω, to calculate partial Pearson correlation and partial Spearman correlation, we used the Moore–Penrose pseudo-inverse [67]. To construct the association network, we determined statistically significant associations using an empirical permutation test. We randomised matrix **X** obtaining 2000 matrices ${𝐗}_{rand}$ in which any statistical association is due to random noise. We then re-calculated the pairwise metric $\xi_{ij}^{rand}$ on the randomised matrices. The empirical p-value was calculated as the fraction of times the random association magnitude was greater than the one measured on the 16S rDNA-seq table


$$\begin{array}{c} p_{value}=\frac{\#\{|\xi_{ij}^{rand}|\geq |\xi_{ij}|\}}{2000+1} \end{array}$$

After the p-values were corrected for multiple testing using the Benjamini-Hochberg procedure, the pairwise association $\xi_{ij}$ was determined to be significant if $p_{value}\leq 2\alpha$ (with α=0.025). The corresponding adjacency matrix value was then set to one $A_{ij}=1$, obtaining an unweighted and undirected graph.

#### Unconditional dependence methods.

The first methods developed to infer microbial interactions measure the dependence between species pairs without considering the effect of other species. We refer to them as unconditional dependence methods since they don’t condition the measured dependence metric on the effect of other variables. In this class of methods, (for a detailed explanation of the methods consult S2 File) we find: Proportionality [37], SparCC [68], CCLasso [39] and a more recent method SECOM [46], which corrects for biases introduced by the 16S rDNA-seq pipeline (SECOM uses two metrics to measure linear and non-linear associations: Spearman correlation coefficient and distance correlation coefficient [69], respectively). No preprocessing, such as log-ratio transformations, was performed for any of the methods, as they are methods developed specifically for metagenomics data and all implementations include some form of preprocessing. For Proportionality, we followed the same empirical permutation test described above to determine statistically significant associations and construct the inferred association network. The other methods output the correlation matrix $𝐑\in {\mathbb{R}}^{p\times p}$ and a p-value for each correlation coefficient. To obtain the adjacency matrix **A**, we corrected the p-values for multiple testing using the Benjamini-Hochberg procedure, and set $A_{ij}=1$ if $p_{value}<2\alpha$ with α=0.025.

#### Conditional dependence methods.

When relying on unconditional dependence between variables, the resulting inferred network may contain spurious edges. By spurious edge we mean an association between two species determined by the interaction with a third species (or a set of species), that by simultaneously influencing both species introduces an indirect dependence between the two (Fig 1). To avoid this, more recent inference methods use the concept of conditional independence to infer species associations.

**Fig 1:**
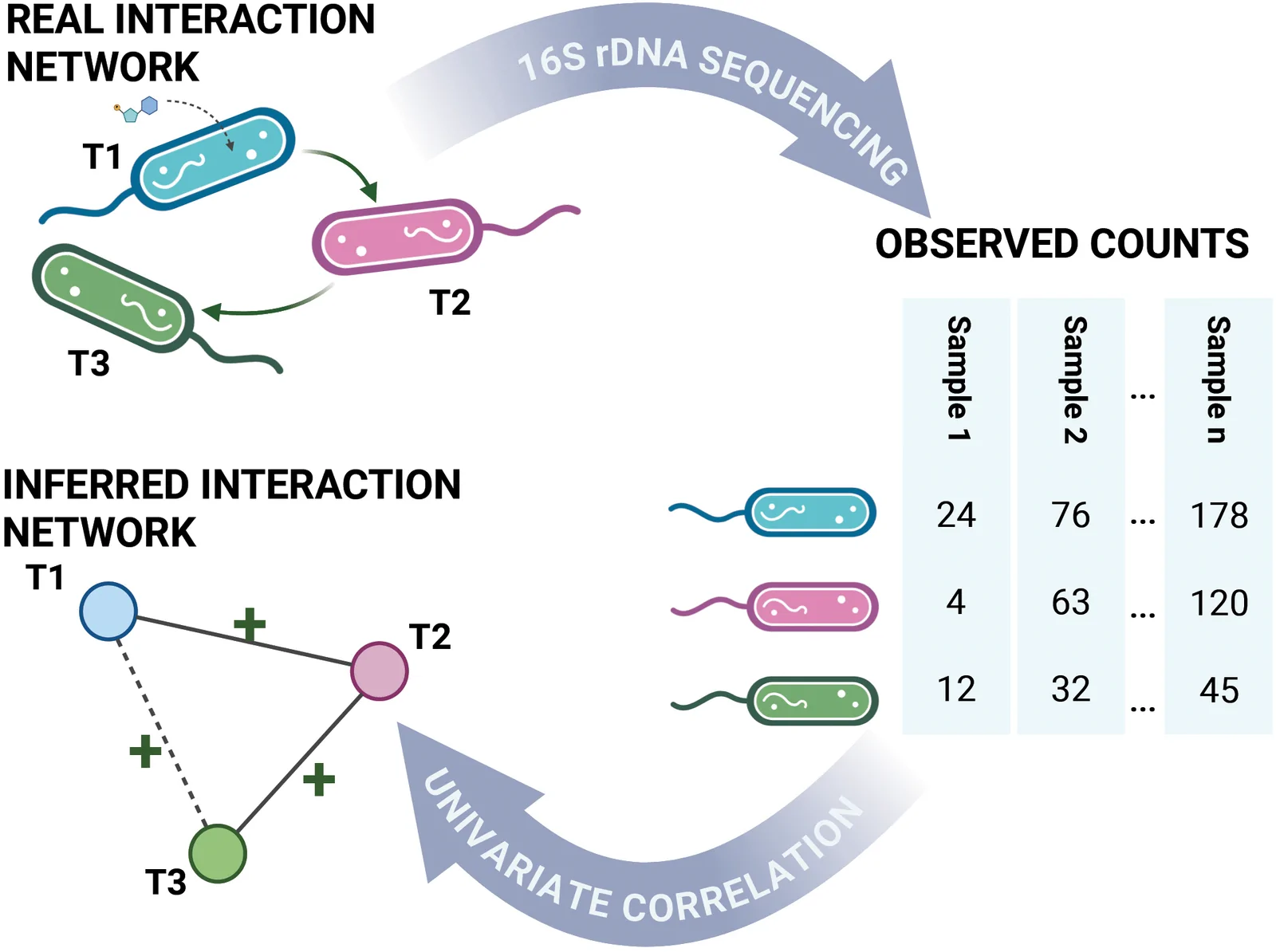
Measuring unconditional associations leads to spurious edges. A depiction of a toy example of a microbial community with 3 species. In the real interaction network, species T1 has a positive interaction with species T2 and T2 has a positive interaction with species T3. When measuring associations using unconditional metrics, like classical correlation, we would infer a positive interaction between all three species, since all grow in counts because of mutualistic interaction (given no significant external disturbance). In reality, the variability in T3 abundance can be explained considering only T2, as there is no real interaction between T1 and T3. Thus the use of conditional association metrics, which measure association conditioned on the effect of other variables, can avoid the inference of spurious edges. Created in BioRender. MARIOTTO, P. (2026) https://BioRender.com/l15o298.

Given a set of *p* random variables $𝒳=\{x_{1},...,x_{p}\}$, with P={1,2,...,p} the index set, two random variables $x_{i},x_{j}\in 𝒳$ are conditionally independent $x_{i}⟂x_{j}\;|\;{𝒳}_{P\setminus \{i,j\}}$ if $p(x_{i}\;|\;x_{j},{𝒳}_{P\setminus \{i,j\}})=p(x_{i}\;|\;{𝒳}_{P\setminus \{i,j\}})$, with ${𝒳}_{P\setminus \{i,j\}}$ all the variables in the set except $x_{i}$ and $x_{j}$ (from now on abbreviated to ${𝒳}_{\neg \{i,j\}}$), meaning that $x_{j}$ gives no additional information about $x_{i}$ that was not already known from the other variables in the set [70]. This concept can be used to obtain the association network from the conditional independence graph of the *p* species, which encodes the conditional dependence structure of the variables (defined as a graphical model), where (*i*,*j*) is not an edge in the graph if and only if $x_{i}⟂x_{j}\;|\;{𝒳}_{\neg \{i,j\}}$ [70]. If the *p* random variables follow a multivariate Gaussian distribution 𝒳~𝒩(μ,Σ) then two random variables $x_{i},x_{j}\in 𝒳$ are conditionally independent $x_{i}⟂x_{j}\;|\;{𝒳}_{\neg \{i,j\}}$ if and only if $\Omega_{ij}=0$ where $\Omega =\Sigma^{-1}$ [70]. Thus, under the assumption of multivariate normality, the estimation of the precision matrix Ω, given *n* independent and identically distributed observations, is sufficient to construct the independence graph of the *p* species.

The methods belonging to this class are: SPIEC-EASI [40] (which implements two approaches: Meinshausen-Bühlman method [71] and Glasso [68]), SPRING [41], gCoda [42], FlashWeave [43] (which measures association using two metrics: partial correlation and partial mutual information), and two methods HARMONIES [44] and COZINE [45] that also address the zero-inflation of 16S rDNA-seq data (for a detailed explanation of the methods, consult S2 File). As for unconditional methods, no external preprocessing was performed as this step is managed internally. These conditional dependence methods give as output a sparse association matrix, where the non-significant associations have been set to zero. For this reason, the adjacency matrix **A** was obtained by binarizing the output association matrix.

### Evaluation of methods’ performance

To evaluate methods’ performance, we compared the ground-truth network used to generate the simulated data with the inferred association network. Some microbial interactions may be intrinsically unidentifiable from count data alone, in both real and simulated settings, and experimental or sequencing biases can introduce additional artefacts. Nevertheless, because network inference methods aim to reconstruct the underlying community interaction network, benchmarking against the original adjacency matrix **A** provides the most direct and unambiguous measure of performance. This comparison is also straightforward, as the evaluated methods return either an inferred network or a corresponding list of edges. We used various metrics that can be grouped into three categories: edge-wise metrics, node-wise metrics, and graph-wise metrics. In addition, we assessed the computational burden of the tested methods by measuring both runtime and memory usage.

Runs in which a method failed were excluded from the evaluation of that method’s performance. Only successful runs were considered, and performance for a given simulation scenario was reported only when at least 10 of the 50 iterations were completed successfully. In our tests, these failures did not appear to be specific to our computing environment, as they were also observed on other systems. Moreover, the use of software containers reduces the likelihood that the failures were caused by system-specific software configurations.

#### Edge-wise metrics.

The first category of metrics concerns the evaluation of the methods in estimating each edge (edge-wise metrics). Since the output of the methods is an estimated adjacency matrix $\hat{𝐀}$ of ones and zeros (${\hat{A}}_{ij}=1$ indicates an inferred edge between node *i* and node *j*), we can use classical confusion matrix metrics to measure the performance of the methods. Considering the inferred adjacency matrix $\hat{𝐀}$ and the ground truth adjacency matrix **A**, each element of the matrix (and thus each inferred edge) can be classified as


$$\begin{array}{c} TP\;if\;{\hat{A}}_{ij}=1\;\wedge \;A_{ij}=1\;\;\;TN\;if\;{\hat{A}}_{ij}=0\;\wedge \;A_{ij}=0\\ FP\;if\;{\hat{A}}_{ij}=1\;\wedge \;A_{ij}=0\;\;\;FN\;if\;{\hat{A}}_{ij}=0\;\wedge \;A_{ij}=1 \end{array}$$

Given this definition, the performances of the methods can be evaluated using basic rates for confusion matrices like


$$\begin{array}{c} Precision=\frac{TP}{TP+FP}\;\;\;Recall=\frac{TP}{TP+FN} \end{array}$$

and summary statistics like accuracy, F1 score and MCC. In this study, we use MCC, as it is one of the most complete summary statistics for binary classification [72], as high MCC values ensure good results in all four categories of confusion matrix.


$$\begin{array}{c} MCC=\frac{TP\times TN-FP\times FN}{\sqrt{\left(TP+FP)(TP+FN)(TN+FP)(TN+FN\right)}} \end{array}$$

MCC has the advantage of being an unbiased metric for unbalanced labels, and in the case of binary classification, it corresponds to Pearson’s correlation coefficient [73], giving it a straightforward interpretation.

#### Node-wise metrics.

The second class of metrics used comes from a network science approach and can generally be called centrality measures. These centrality measures are calculated for each node (hereafter node-wise metrics) and are used to identify important nodes in the graph. We thus compared the centrality measured in the inferred graph against the centrality of the nodes in the true graph. The rationale behind this comparison is to see if the methods correctly identify key species in the network, even if they make errors in estimating some interactions between species.

Given a node i∈V, the nodes connected to *i* define the neighbourhood of the node, and the degree of a node $k_{i}$ is defined as the number of edges connected to that node (or the cardinality of the neighbourhood of the node). Given a pair of nodes (*i*,*j*), we define as $P_{ij}^{1},P_{ij}^{2},...,P_{ij}^{s}$ the *s* paths connecting the two nodes and $\left|P_{ij}^{1}|,|P_{ij}^{2}|,...,|P_{ij}^{s}\right|$ the paths’ lengths. The distance between two nodes $d_{ij}$ is defined as $\min_{s}(|P_{ij}^{s}|)$ (often called the geodesic) and the eccentricity of a node is the maximum distance between node *i* and any other node in the graph $ecc_{i}=\max_{j}d_{ij}$.

We considered various centrality metrics present in the literature and implemented in the Networkx [74] Python library:

- *Betweenness centrality* [75]: expresses the centrality of a node as the extent to which it falls on the shortest path between pairs of other vertices.
$$\begin{array}{c} {𝒞}_{B}(i)=\sum_{u,v\in V}\frac{\left|G(u,v|i)\right|}{\left|G(u,v)\right|} \end{array}$$
  With *G*(*u*,*v*) the geodesic paths between two nodes *u* and *v*, |*G*(*u*,*v*)| the number of geodesic paths and *G*(*u*,*v*|*i*) the geodesic paths between *u* and *v* that contain node *i*.
- *Eigenvector centrality* [76]: computes the centrality of a node by adding the centrality of its neighbours; thus, a node is more central if it is connected to other important nodes. The centrality for node *i* is the *i*-th element of the right eigenvector associated with the positive eigenvalue λ of maximum modulus
$$\begin{array}{c} {𝒞}_{E}(i)=\sum_{j|(i,j)\in V}\frac{x_{j}}{\lambda } \end{array}$$
- *Closeness centrality* [77]: refers to the measure of the inverse average distance between a node and every other node in a network. It indicates if a node has a short distance from all the other nodes, with higher scores indicating a more central and important position in the network.
$$\begin{array}{c} {𝒞}_{C}(i)=\sum_{j\in V}\frac{p-1}{d_{ij}} \end{array}$$

As a summary statistic for all node-wise metrics, we computed the coefficient of determination *R*^2^ between the centrality of the node in the true graph 𝒞 and the one calculated on the same node but on the inferred graph $\hat{𝒞}$


$$\begin{array}{c} R^{2}=1-\frac{\sum_{i}({𝒞}_{i}-{\hat{𝒞}}_{i}{)}^{2}}{\sum_{i}({𝒞}_{i}-\overset{―}{𝒞}{)}^{2}} \end{array}$$

for ease of representation, we transformed negative values of *R*^2^ by mapping them into [−1,0] according to $R^{2}=\frac{R^{2}}{1-R^{2}}\;\forall R^{2}<0$.

#### Graph-wise metrics.

Finally, the third class of metrics consists of graph-wise metrics that aim to capture the topological characteristics of the graph at a high level. As for the centrality measures, we confronted the inferred and true graph metrics to see if the methods were able to maintain the true graph macroscale structure, which is an important factor in determining microbial communities’ functionality. The metrics considered (implemented in the Networkx [74] and SciPy [78] Python libraries) are:

- *Two-sample Kolmogorov–Smirnov statistic* [79]: expresses the maximum difference between two empirical cumulative distribution functions. Here we applied it to the degree distribution, estimated by a histogram of the nodes’ degrees, to compare the true degree distribution *p*(*k*) to the inferred one $\hat{p}(k)$
$$\begin{array}{c} \delta_{KS}=\max\{|P(k)-\hat{P}(k)|\} \end{array}$$
  where *P*(*K*) is the cumulative degree distribution. Thus, $\delta_{KS}\in [0,1]$ and if $\delta_{KS}≅0$, the two empirical distributions are similar, while if $\delta_{KS}≅1$, they are dissimilar.
- *Density*: the ratio between the number of edges in a graph and the number of possible edges in the same graph. In a complete undirected graph, the maximum number of edges is $|E{|}_{max}=\frac{p(p-1)}{2}$ thus
$$\begin{array}{c} \rho (𝒢)=\frac{\left|E\right|}{|E{|}_{max}}=\frac{2|E|}{p(p-1)} \end{array}$$
- *Diameter*: defined as the maximum node eccentricity in the graph. For disconnected graphs, we used the maximum of the diameters ${𝒟}^{\prime }$ calculated on the connected components of the graphs.
$$\begin{array}{cc} 𝒟 & =\max_{i\in V}(ecc_{i})\;if\;𝒢\;is\;connected\\ 𝒟 & =\max({𝒟}^{\prime })\;\;otherwise \end{array}$$
- *Radius*: defined as the minimum node eccentricity in the graph. For disconnected graphs, we used the minimum of the radiuses ${ℛ}^{\prime }$ calculated on the connected components of the graphs.
$$\begin{array}{cc} ℛ & =\min_{i\in V}(ecc_{i})\;if\;𝒢\;is\;connected\\ ℛ & =\min({ℛ}^{\prime })\;\;otherwise \end{array}$$
  Notably, if a graph presents a disconnected vertex, the radius of the graph is undefined. In this work, we considered ℛ=0 if the graph had isolated nodes.
- *Average distance (Average shortest path)*: is the average of the distance across all nodes
$$\begin{array}{c} \overset{―}{d}=\sum_{i,j\in V}\frac{d_{ij}}{p(p-1)} \end{array}$$
  We set $\overset{―}{d}=\infty$ if 𝒢 is not connected.
- *Average clustering coefficient*: the clustering coefficient $C_{i}$ is a measure that shows whether a node has the tendency to form clusters or tightly connected communities. It is defined as the number of edges between the *i* node neighbours divided by the number of possible connections between these neighbours. Then, by averaging across vertices, we obtain the average clustering coefficient, which shows the tendency of the network to form clusters
$$\begin{array}{c} C_{i}=\frac{2e_{i}}{k_{i}(k_{i}-1)}\;\overset{―}{C}=\sum_{i\in V}\frac{C_{i}}{p} \end{array}$$
  with $k_{i}$ the degree of node *i*, i.e., the number of neighbours, and $e_{i}$ the number of edges between the neighbours of *i*.

Since we are interested in the difference between the true graph-wise metric *M* and the one calculated on the inferred graph $\hat{M}$, in the results we report the delta between the two metrics normalised by the true value


$$\begin{array}{c} \Delta M=\frac{M-\hat{M}}{M} \end{array}$$

Note that when *M* or $\hat{M}$ are ∞ (as can happen for $\overset{―}{d}$), or *M* = 0 (as can happen for ℛ), then the computed delta can be ΔM=±∞.

#### Computational burden.

Another important aspect of the methods’ performance, alongside their ability to make correct inferences, is the amount of computational resources required to make such inference. Consequently, we quantified the computational burden of each method by measuring the average run time and peak memory usage for a subset of the simulated scenarios (S6 Dataset), as preliminary testing confirmed that this subset captured the full variability of the methods. For each scenario considered, computational burden metrics were aggregated over the 50 simulated datasets. We ran each method in serial mode on the 50 datasets, using a Linux server equipped with 4 Intel Xeon Gold 6252N and 3 TB RAM. The Slurm workload manager [80] was used to schedule computation and collect resource usage statistics. Collected statistics excluded post-processing and data storage operations; only method-specific data preprocessing (when required) and the execution of the network inference methods were included. The average run time was computed by dividing the reported job elapsed time by 50 or by the number of successful runs in cases where errors occurred.

## Results and discussion

### Graph generation model and sample diversity exert consistent and substantial effects on performance

First, we generated 16S datasets using the default simulator parameters and we focused on the effect of the graph generation model ℳ and the level of β−diversity between samples $\overset{―}{\beta }$ (first two rows of Table 1) on the performance of the methods. We considered 3 generation models ℳ=(ER, BA, WS) and 3 levels of β−diversity (low, medium and high) for a total of 9 scenarios. All other parameters were set to their default values (bold values in rows 3–7 of Table 1) and 50 datasets were generated for each scenario.

In Fig 2 we can see the MCC for each method as the graph model and β-diversity change. We observed substantial differences both among methods and within individual methods across different graph generation model and β−diversity levels.

**Fig 2:**
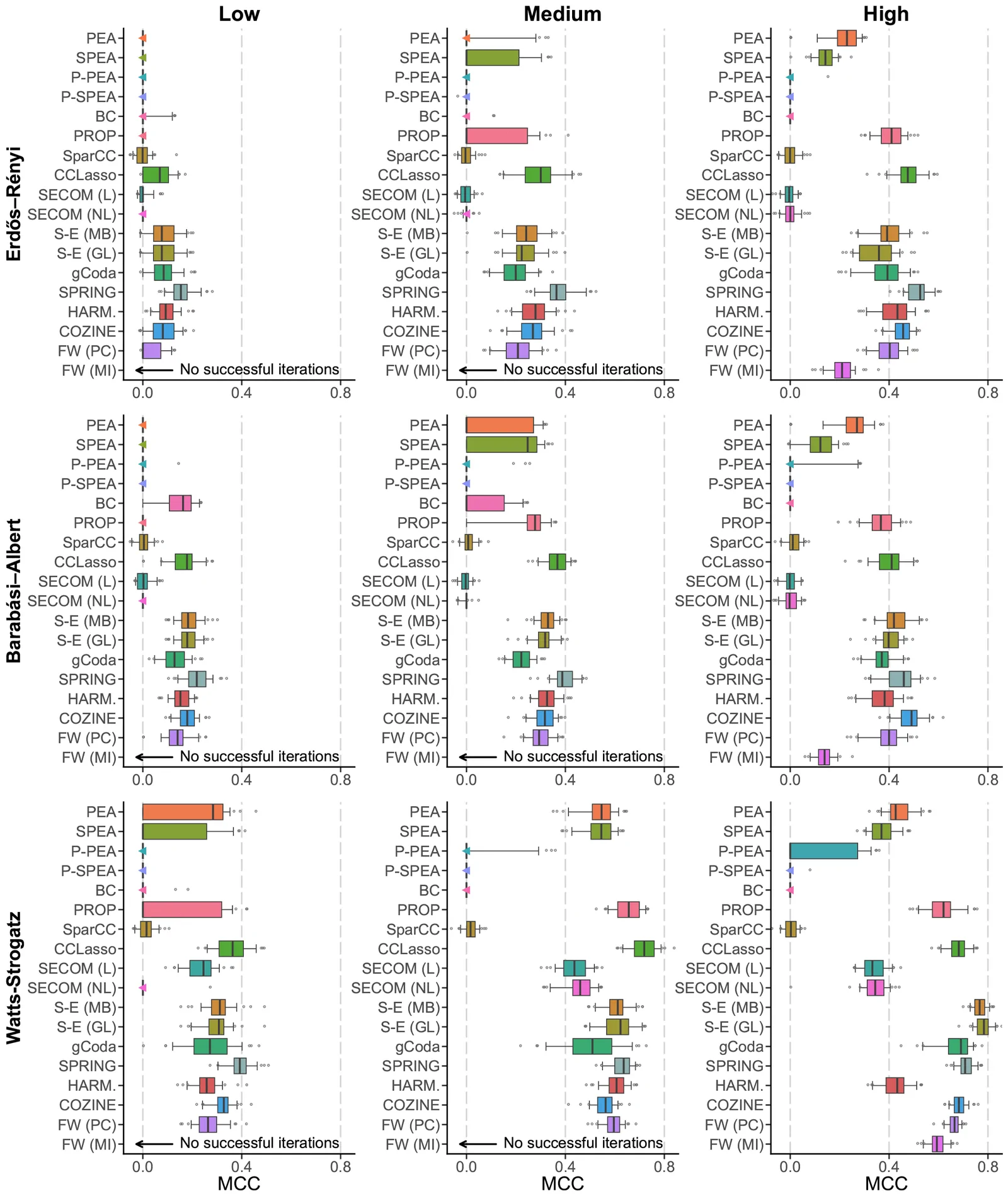
Methods’ performance for different graph model and β-diversity. Figure shows the MCC values for each method for different graph generation models ℳ= ER, BA, WS (on the rows) and different levels of β−diversity between samples (on the columns). As reported in the figure, FlashWeave (MI) had no successful runs over the 50 iterations for low and medium β−diversity.

In general, the methods showed moderate performance, with median MCC values $\overset{―}{MCC}\leq 0.4$, except for the WS graph model, in particular for medium and high β−diversity, where $\overset{―}{MCC}$ reached high values for some methods ($\overset{―}{MCC}=0.77$ for SPIEC-EASI (MB), $\overset{―}{MCC}=0.78$ for SPIEC-EASI (Glasso) and $\overset{―}{MCC}=0.71$ for SPRING). We found that the major factor hindering the methods’ performance is that they infer a usually small subset of the edges, leading to a large number of false negatives, whereas the called edges are generally correct. This is reflected in the precision and recall values (Figs C and D in S3 File), as we consistently observe mean precision values $\overset{―}{Precision}≅0.8$ especially for conditional dependence methods, while the observed mean recall values were considerably lower, $\overset{―}{Recall}=0.17$ considering all methods and with best values of $\overset{―}{Recall}=0.35$ for SPRING and CCLasso. Overall, conditional dependence methods were consistently the best-performing methods (with SPRING almost always yielding the highest $\overset{―}{MCC}$ value). Among the other methods, CCLasso was the only one that consistently matched the performance of conditional dependence methods. These changes were also observed for the other node-wise (Figs E–G in S3 File) and graph-wise (Figs H–M in S3 File) metrics, although in some cases the pattern was less evident.

Looking at the results, we can clearly identify some trends. The WS graph model was always the most favourable for the methods, followed by BA and ER. This could be a result of the regular graph topology resulting from the WS model. As a result, the degree distribution is nearly uniform, and there are no high-degree nodes, for which the effect of a single interaction on the species counts is greatly confounded by the other interactions, making the inference challenging.

For the β−diversity level, we clearly see a general increase in methods’ performance as the β−diversity increases, with $\overset{―}{\Delta MCC}(high,medium)=0.06$ and $\overset{―}{\Delta MCC}(high,low)=0.15$ for the BA model. We observed that high levels of β−diversity correspond to perturbations with a high number of turned-off external resources supply (Fig D in S1 File), meaning that the resource is no longer provided by the environment but can only be present in the community as result of metabolism by-product. This seems to suggest that in rich environments with lots of resources supplied to the community (like the human gut), the co-occurrence-based inference of interactions is more difficult compared to poorer ones (like the soil), as the dependence on metabolite by-products from other species is less severe and thus has a smaller impact on species counts.

Unlike edge-wise, node-wise and graph-wise metrics, we did not find considerable differences in the computational cost of the methods when considering different topologies or different levels of β-diversity.

Since these trends were consistent across all scenarios and metrics tested, we selected the BA graph generation model and a medium β-diversity level as representative settings to illustrate the methods’ performance in the following sections. The complete sets of results, with all combinations of topology, β-diversity level and parameter values, are provided as Supplementary Data (S1, S2, S3, S4, S5 Datasets). In S3 File are available additional figures, Figs N–AT display the results for all topologies with a level of medium β-diversity, and are referenced throughout the manuscript where particularly relevant.

### Increasing sample size improves network inference performance

One of the few parameters that can be truly controlled in a real-world experiment is the number of samples collected. This is also one of the main factors in determining the experiment cost, especially in a clinical setting where sample collection is not just a matter of economic considerations but also an ethical one, as it often causes discomfort to patients. Thus, evaluating the cost-effectiveness of increasing the number of samples is of great importance. To this end, we simulated communities of 50 taxa (*p* = 50) varying the sample size n∈[50,75,100,200,300,500,1000] and keeping all other parameters at the default value (i.e., $\rho (𝒢)=5\%,\;r_{neg}=0.05,\;K=100\%$). The complete results are reported in S1 Dataset; figures for selected scenarios of interest are available in S3 File (Figs N–S).

As we can see in Fig 3A, the general trend is that methods’ performance increases with the number of samples. Starting at *n* = 50 = *p* all methods have poor performance ($\overset{―}{MCC}\leq 0.3$). As the number of samples increases to *n* = 1000, some methods can reach peaks of $\overset{―}{MCC}≅0.6$, and $\overset{―}{MCC}≅0.8$ for the WS model (Fig P in S3 File), and most methods reach a stable $\overset{―}{MCC}>0.4$ for *n* > 300. After the 300 sample mark, for most methods the increase in performance was marginal across all graph models, indicating a saturation effect (Figs P–R in S3 File).

**Fig 3:**
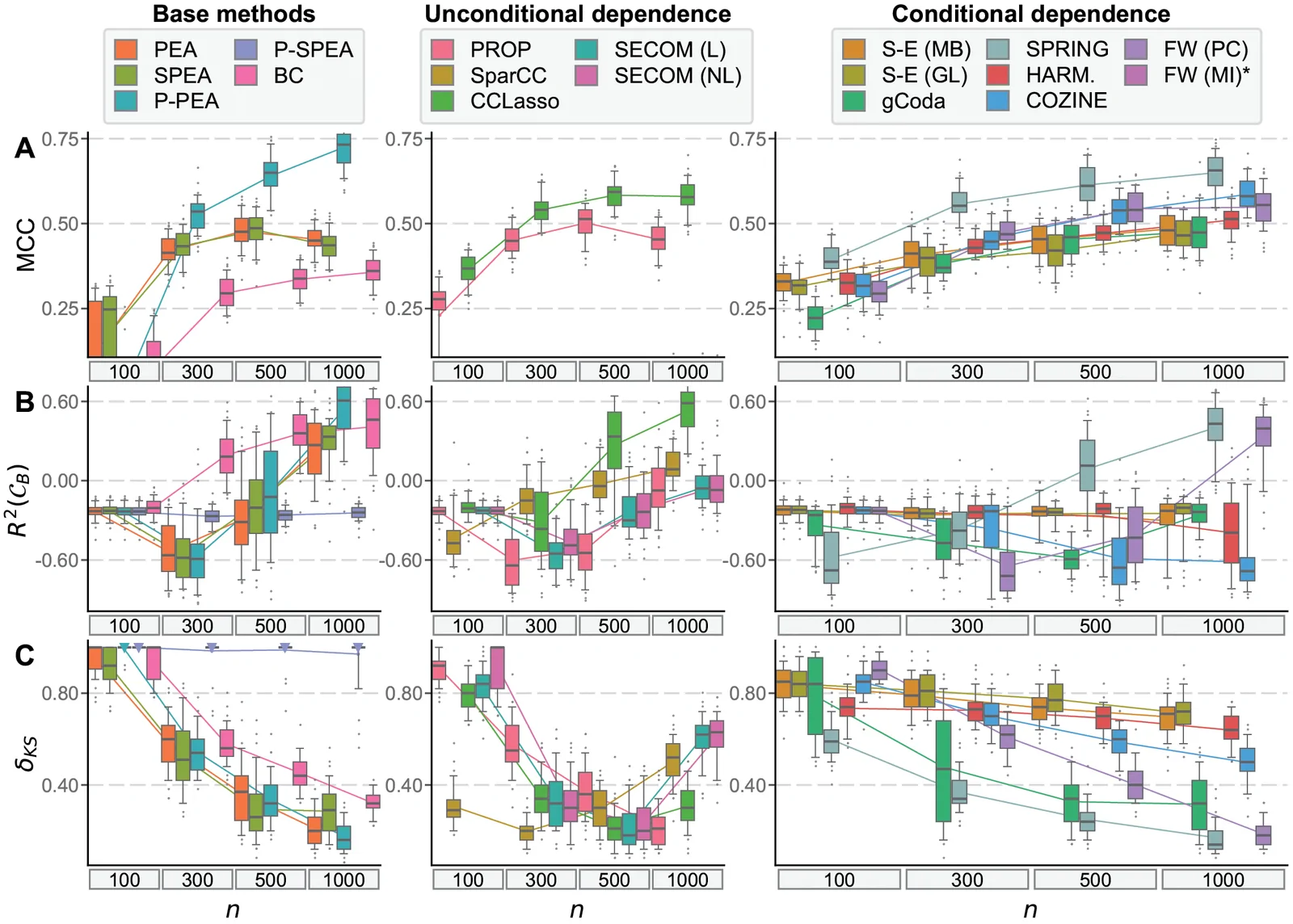
Methods’ performance at increasing number of sample size. Here we show the results for the BA model with medium β−diversity between samples (see Figs N–S in S3 File for the other results). Data were simulated keeping all other parameters fixed at the default values $\rho (𝒢)=5\%,\;p=50,\;r_{neg}=0.05,\;K=100\%$. Methods are grouped based on the implementation strategy, and each column refers to one class of methods. Here we show 3 of the evaluation metrics: **A)** MCC, **B)** *R*^2^ for betweenness centrality and **C)** the Kolmogorov–Smirnov statistic $\delta_{KS}$. Methods marked with an asterisk had no successful runs over the 50 iterations for any of the scenarios in the figure.

Interestingly, some base methods show similar performance compared to other more complex methods, designed ad-hoc for this application. In particular, partial Pearson correlation is the best-performing of all methods for *n* > 500 (while for low samples sizes it has poor performance). Pearson and Spearman’s correlation have results comparable to the other methods, albeit slightly poorer (Fig 3A). These findings were also highlighted by other studies [50].

In general, increasing the number of samples improved the resemblance of the inferred graph compared to the true one, although some considerable differences are also present at high sample sizes. Looking at node-wise metrics (Figs N, P and R in S3 File), the coefficient of determination *R*^2^ between the true betweenness centrality and the inferred one increased for all methods as the sample size increased. The methods that showed the strongest improvement were Pearson partial correlation (*R*^2^ = 0.61 at *n* = 1000), CCLasso (*R*^2^ = 0.59 at *n* = 1000), Bray-Curtis dissimilarity (*R*^2^ = 0.46 at *n* = 1000) and SPRING (*R*^2^ = 0.43 at *n* = 1000), while for the other methods, the increase in *R*^2^ was more modest (Fig 3B). As centrality metrics give a quantification of the importance of a node in the graph, we wanted to see if the methods could identify high centrality nodes. Restricting the comparison to the 5 highest centrality nodes, we saw a general reduction in the *R*^2^ value; for the best performing Pearson partial correlation, CCLasso, Bray-Curtis dissimilarity and SPRING, the values were respectively $R_{TOP}^{2}=0.42,\;R_{TOP}^{2}=0.11$, $R_{TOP}^{2}=0.25$ and $R_{TOP}^{2}=0.32$ (Fig T in S3 File). For the other centrality metrics, there were no significant gains, as the median $\overset{―}{R^{2}}≅0$ for all sample sizes tested (Figs N, P and R in S3 File).

In terms of graph-wise metrics, we observed some improvements in the resemblance of the inferred graph compared to the true one (Figs O, Q and S in S3 File). In particular, the Kolmogorov–Smirnov statistic $\delta_{KS}$ measures the similarity in the degree distributions of the two graphs. As in Fig 3C, $\delta_{KS}$ tends to decrease, meaning the degree distributions are more similar for almost all methods as the number of samples increases, with the best result obtained by SPRING ($\delta_{KS}=0.14$). The only methods that show a different trend are SparCC, SECOM (linear), and SECOM (non-linear), for which $\delta_{KS}$ has a V-shaped trajectory, but since these methods have $\overset{―}{MCC}≅0$, this is likely an artefact caused by spurious inferred edges. Regarding the other network metrics, we see a general improvement as the sample size increases, with unconditional dependence methods generally outperforming conditional dependence methods across all graph-wise metrics (Figs O, Q and S in S3 File). This is likely caused by the fact that conditional dependence methods tend to infer sparser but more precise graphs, leading to different graph-wise metrics.

In terms of computational burden (Fig 4), increasing the number of samples leads to higher run times and memory usage for all base methods and Proportionality. The remaining methods exhibit only minor variations in execution time and memory consumption, with the exception of SECOM (NL) and HARMONIES, which show an increase in run time of ~6min (5340% relative increase) and ~45min (4500% relative increase), respectively. The increased sample size allowed us to better appreciate the difference in computational burden between the methods. In terms of run time at *n* = 500, the best performers were gCoda and FlashWeave (both implementations), both with a run time of ~1s. In contrast, HARMONIES showed the worst performance with a run time of ~46min. In terms of memory, at *n* = 500, the best performer was CCLasso (197MB required), while the worst performers were HARMONIES and SPIEC-EASI (GL), with 1.8GB and 1.5GB required, respectively.

**Fig 4:**
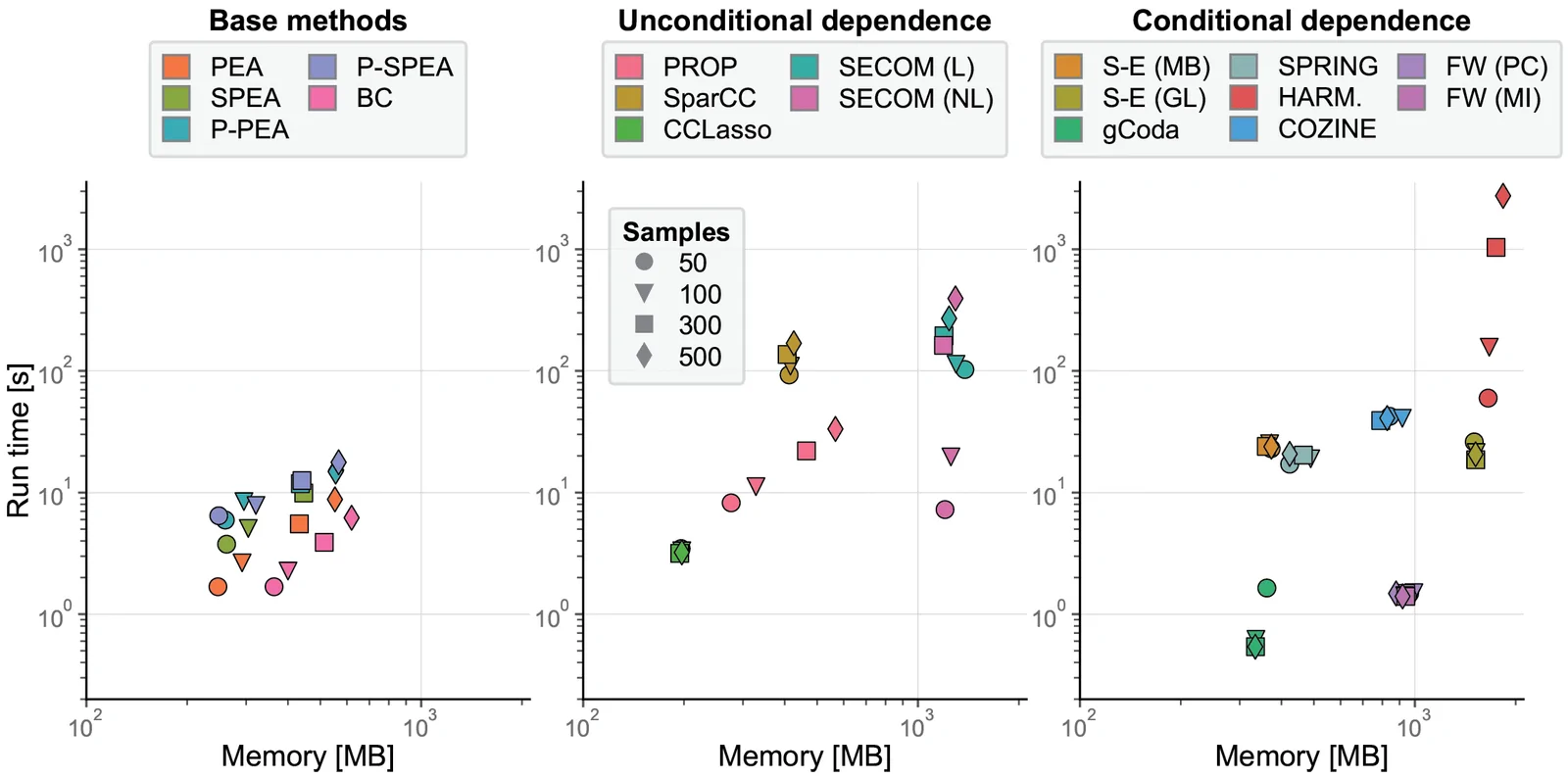
Methods’ resource utilisation at increasing number of samples. Scatter plot, with axis in log-scale, of maximum memory and average run time required by each method to infer a single network. Measurements were collected using SLURM workload manager accounting tools. We measured the run time and peak memory usage for a subset of the sample sizes used in this study, namely n∈[50,100,300,500], all the other simulation parameters were set to: BA graph model, medium β-diversity, $\rho (𝒢)=5\%,\;p=50,\;r_{neg}=0.05$ and *K* = 100%.

As expected, increasing the sample size considerably improves the performance of microbial co-occurrence methods. However, we observed a distinct saturation effect beyond certain sample size thresholds, indicating that indiscriminately increasing the number of samples eventually yields diminishing returns. In addition, larger datasets naturally increase the memory footprint and computational load of these algorithms, making it essential to carefully balance and select an appropriate sample size.

### Community richness affects computational burden and overall accuracy

As real bacterial communities generally comprise hundreds if not thousands of species, we simulated communities with up to *p* = 200 taxa to observe the effect on the methods’ performance. As before, we kept all other simulation parameters fixed ($n=100,\;\rho (G)=5\%,\;r_{neg}=0.05,\;K=100\%$) and simulated communities with p∈[50,100,150,200]. The complete results are reported in S3 Dataset; figures for selected scenarios of interest are available in S3 File (Figs U–Z).

For the methods with an $\overset{―}{MCC}\neq 0$ at *p* = 50, we observed a general decline in performance (Fig 5A), to the point where at *p* = 200 the $\overset{―}{MCC}$ is equal to 0, with the only exception being SPRING with the WS model ($\overset{―}{MCC}=0.25$ see Fig W in S3 File). As in the previous results, the methods show the best performance with the WS model, while the ER and BA models show a similar, lower performance (see Figs W–Y in S3 File). The major factor limiting methods’ *MCC* is the extremely low recall, with median recall always lower than 0.15 (except for SPRING, for which $\overset{―}{Recall}=0.25$ at *p* = 50) and $\overset{―}{Recall}≅0$ for *p* > 100 (Fig 5C). The decline in performance is due to a loss in precision from $\overset{―}{Precision}≅0.9$ at *p* = 50 to $\overset{―}{Precision}≅0.35$ considering the best performing methods SPIEC-EASI (MB), SPIEC-EASI (Glasso), SPRING and COZINE (Fig 5B).

**Fig 5:**
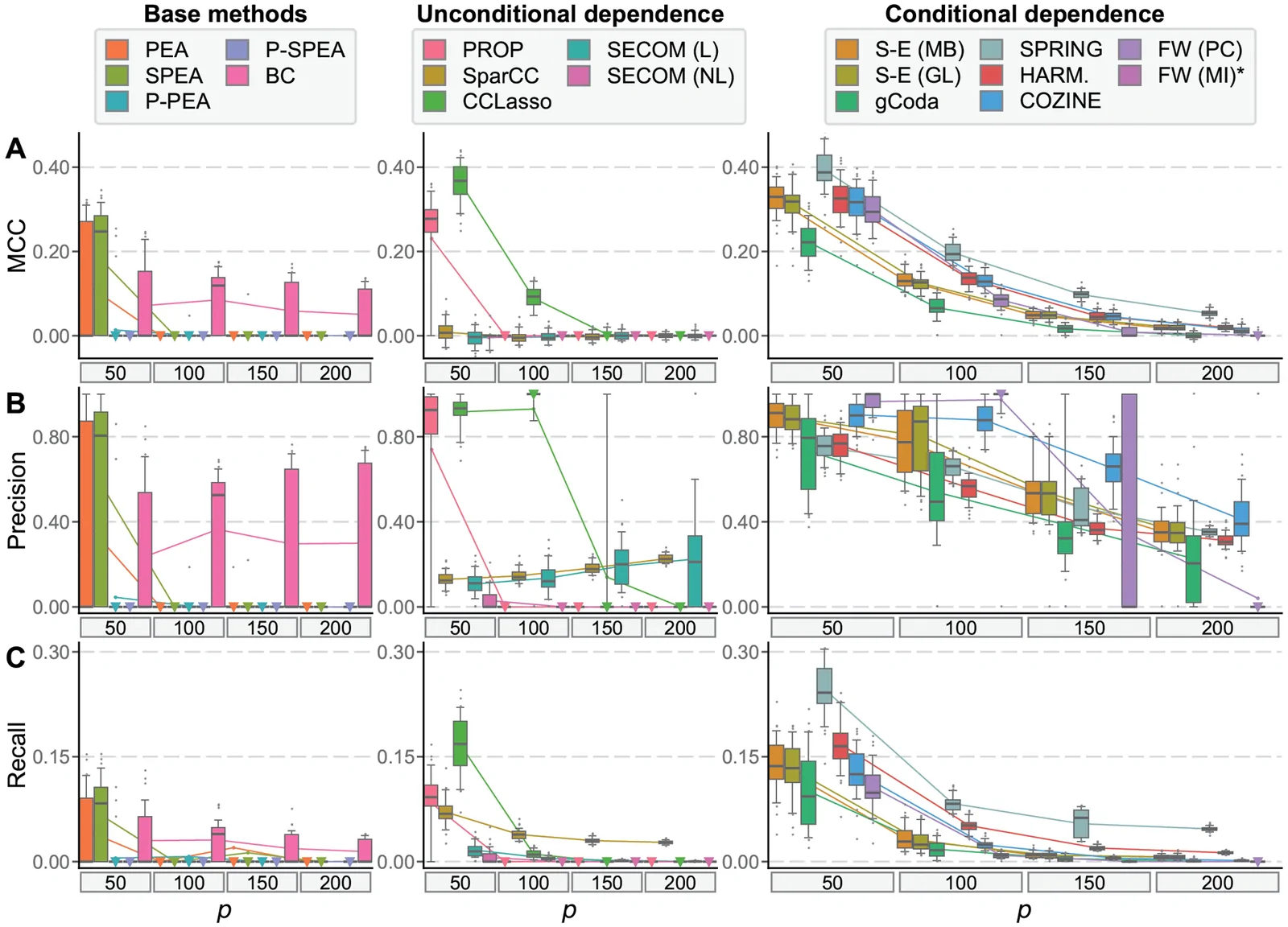
Methods’ performance at increasing number of species. Here we show the results for the BA model with medium β−diversity between samples (see Figs U–Z in S3 File for the other results). Data were simulated keeping all other parameters fixed at the default values $n=100,\;\rho (𝒢)=5\%,\;r_{neg}=0.05,\;K=100\%$. Methods are grouped based on the implementation strategy, and each column refers to one class of methods. Here we show 3 of the evaluation metrics: **A)** MCC, **B)** Precision and **C)** Recall. Methods marked with an asterisk had no successful runs over the 50 iterations for any of the scenarios in the figure.

In terms of node-wise (Figs U, W and Y in S3 File) and graph-wise (Figs V, X and Z in S3 File) metrics, we did not identify any particular trend. The inferred graphs do not resemble the true one in any of the metrics we calculated, except for the diameter (best $\overset{―}{\Delta 𝒟}/𝒟=0.17$ for COZINE). Inferred graphs tend to be disconnected ($\Delta \overset{―}{d}/\overset{―}{d}=\infty$) and present isolated nodes ($\hat{ℛ}=0$). The only exceptions are SparCC, which has $\overset{―}{MCC}=0$, and SPRING.

The increase in community size also had a significant effect on the computing resources needed by the methods. In Fig 6 we see that, in general, all methods require more resources as the number of species increases. Ranging from 50 to 200 species, we found that Pearson correlation and SECOM (linear) showed the highest increase in run time, ~34min (77878% relative increase) and ~31min (1603% relative increase), respectively, while the least impacted method was FlashWeave. In addition, the maximum memory required was affected by the increase in species richness, with an average increase of 2382MB (315% mean relative increase). The most impacted methods were SPIEC-EASI (GL) and HARMONIES, with a respective increase of 18GB (1209% relative increase) and 9.7GB (598% relative increase), confirming them as the most memory intensive methods, while the least impacted ones were SECOM (non-linear), CCLasso and FlashWeave (MI).

These findings emphasise the constraints associated with co-occurrence methods. As we progressively test more realistic simulation parameters by increasing the number of species while maintaining the number of samples constant (*n* = 100), the efficacy of these methods declines significantly and the computational resources needed increase considerably.

**Fig 6:**
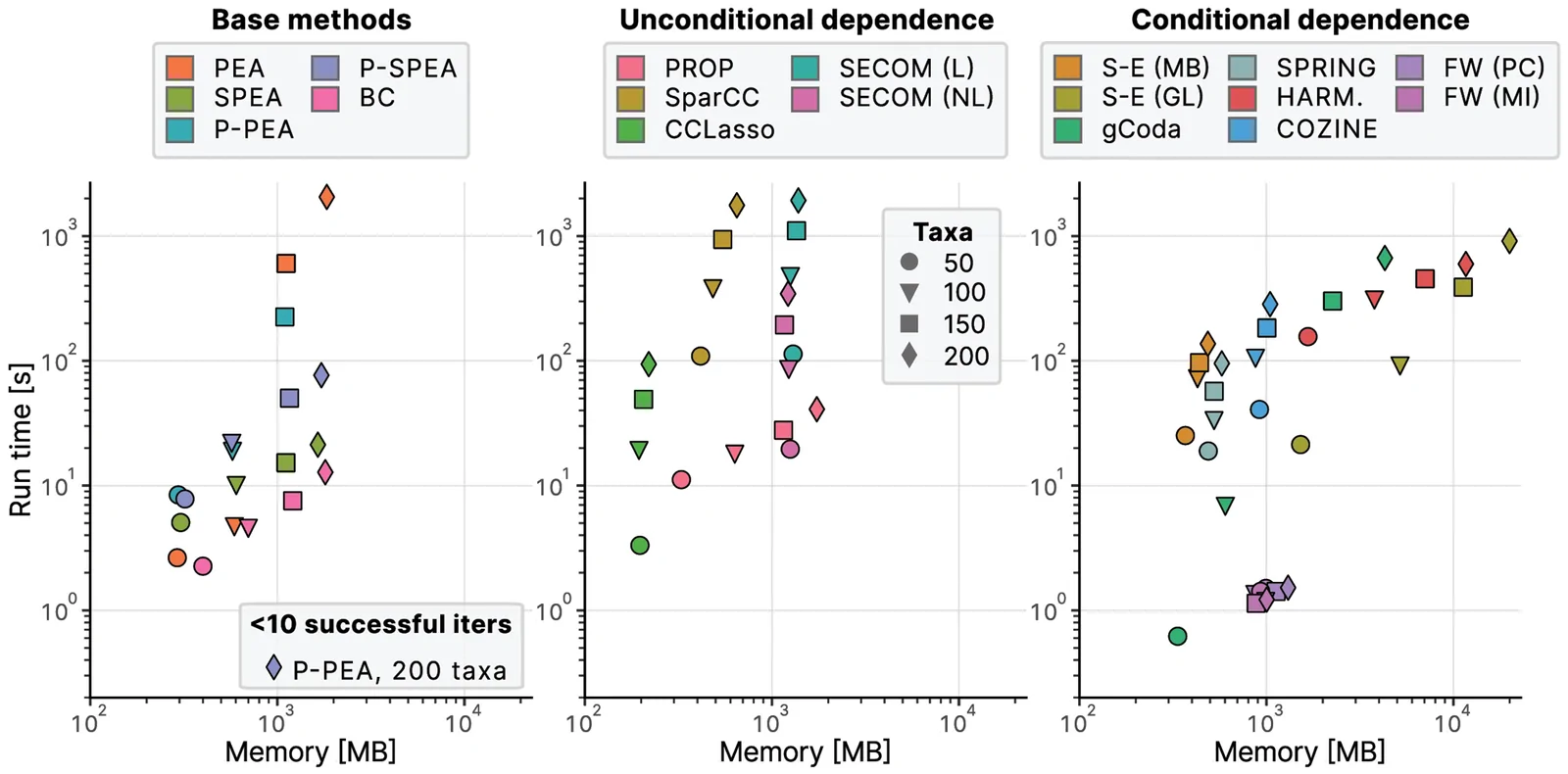
Methods’ resource utilization at increasing number of species. Scatter plot, with axis in log-scale, of maximum memory and average run time required by each method to infer a single network. Measurements were performed using SLURM workload manager accounting tools. The datasets used as input for the methods were generated with varying number of species p∈[50,100,150,200], BA graph model, medium β-diversity and keeping all other parameters fixed ($\rho (𝒢)=5\%,\;n=100,\;r_{neg}=0.05,\;K=100\%$).

### Network density considerably affects methods’ performance

The network density was the main parameter that affected the performance of the inference methods. We simulated communities of 50 taxa (*p* = 50) generated at different density levels ρ(𝒢)∈[2%,5%,8%,10%,15%] and all other parameters were set at the default value ($n=100,\;r_{neg}=0.05,\;K=100\%$). The complete results are reported in S2 Dataset; figures for selected scenarios of interest are available in S3 File (Figs AA–AF).

Examining Fig 7A, we observe that MCC ranges from 0.25 to 0.5 for ρ(𝒢)=2%, followed by a marked and steady decline at increasing density. For all methods, as the density reaches ρ(𝒢)=15% the $\overset{―}{MCC}≅0$, meaning that the methods have the same power as a random estimator. This is mainly because the methods infer networks that have a constant level of density and struggle to adapt to the increase in density. We can see in Fig 7B that the density of the inferred graph $\rho (\hat{𝒢})$ remains constant or even decreases, as the true density ρ(𝒢) increases.

**Fig 7:**
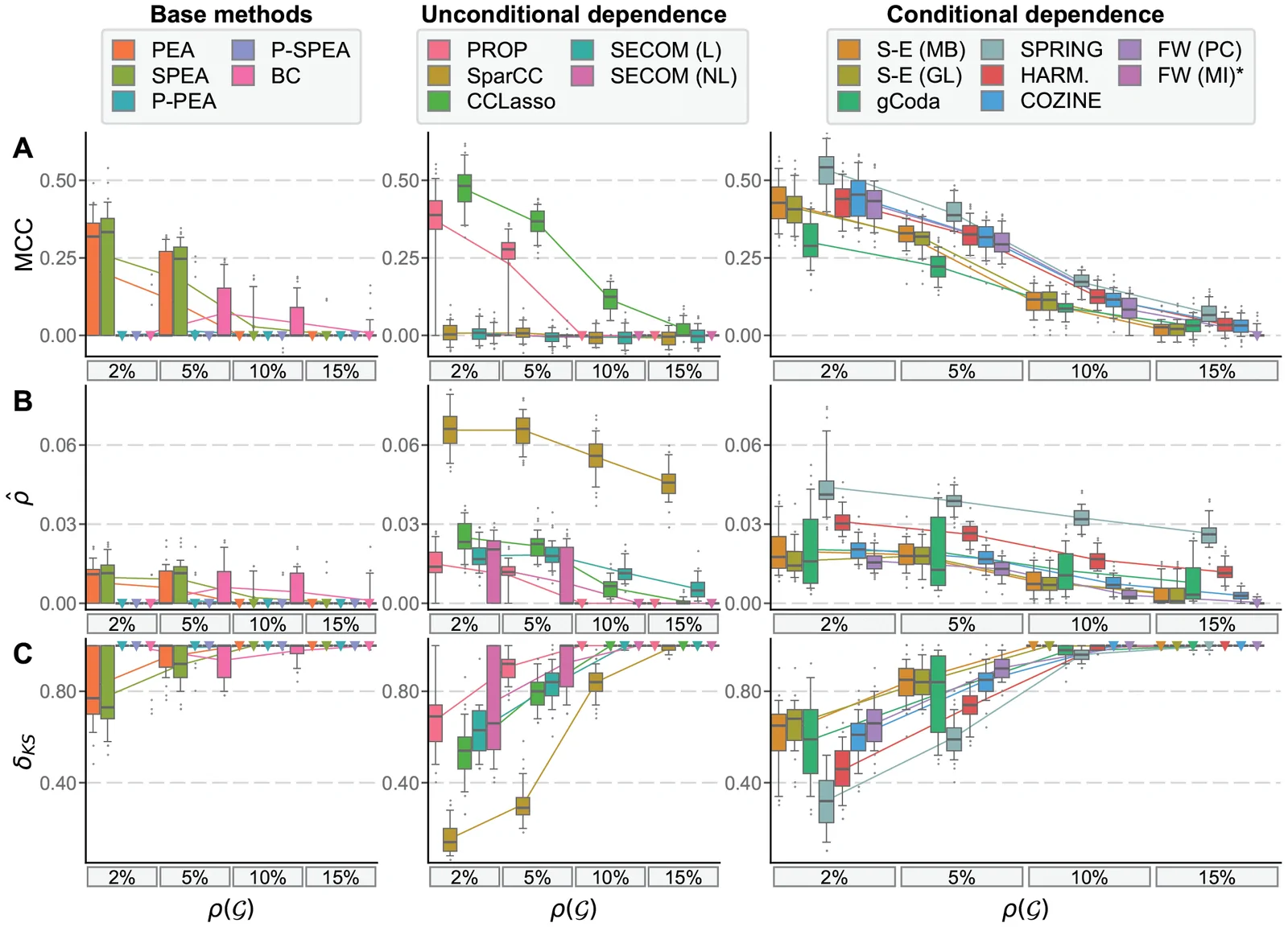
Methods’ performance at increasing density levels. Here we show the results for the BA model with medium β−diversity between samples (see Figs AA–AF in S3 File for the other results). Data were simulated keeping all other parameters fixed at the default values $n=100,\;p=50,\;r_{neg}=0.05,\;K=100\%$. Methods are grouped based on the implementation strategy, and each column refers to one class of methods. Here we show 3 of the evaluation metrics: **A)** MCC, **B)** inferred density $\rho (\hat{𝒢})$ and **C)** the Kolmogorov–Smirnov statistic $\delta_{KS}$. Methods marked with an asterisk had no successful runs over the 50 iterations for any of the scenarios in the figure.

In Fig O (S3 File) we observe that as the number of samples increases, the methods are able to infer denser networks; this suggests that the ability of co-occurrence-based methods to infer dense networks may be determined by the number of samples. It should be noted that in Fig O (S3 File), methods are able to infer networks with similar density to the real one (in this case ρ(𝒢)=2%) only with *n* = 500 samples, ten times the number of taxa (*p* = 50).

As for previous results, we observe that the methods tend to have better performance with the WS graph model (Figs AC and AD in S3 File). The decrease in methods’ performance is mainly due to a loss in recall which starts as soon as the density increases (see Figs AA, AC and AE in S3 File). Taking the mean loss in recall across methods, we obtain $\overset{―}{\Delta Recall}=-0.14$, but this is skewed by methods that have $\overset{―}{Recall}=0$ to begin with. Looking at the best performing methods, we see losses in recall of up to $\Delta \overset{―}{Recall}=-0.4$. The precision remains more stable for the conditional dependence methods up to ρ(𝒢)=10% ($\overset{―}{\Delta Precision}=-0.08$), while for the other methods we observe major declines in precision even at lower density levels. The loss in recall even at low density levels can be explained by the fact that the methods tend to estimate a certain number of edges independently of the density of the true network, reflected in a constant level of inferred density $\rho (\hat{𝒢})$ (Fig 7B). Therefore, with an increase in the number of true edges, there is a corresponding rise in false negatives, dropping the recall while maintaining a more consistent precision.

Concerning node-wise (Figs AA, AC and AE in S3 File) and graph-wise (Figs AB, AD and AF in S3 File) metrics, at increasing network density we observed no improvement in methods performance. The *R*^2^ of all centrality metrics is almost zero at ρ(𝒢)=2%, and remained flat at higher density levels. As density increases, we observed a stark increase in $\delta_{KS}$ (Fig 7C) to the point that for ρ(𝒢)>10%, all methods have $\delta_{KS}≅1$. The methods severely underestimated the radius (see Figs AB, AD and AF in S3 File), inferring $\hat{ℛ}=0$ despite the increase in the real radius. This indicates that the methods maintained isolated nodes in the inferred network even if there were none; this is also reflected in the estimated average shortest path $\hat{d}=\infty$. The only significant exception was SPRING in the WS model, but only for ρ(G)≤5%. All considered, the methods do not seem to capture the true characteristics of denser networks.

The true network topology, the β-diversity between samples, and the network density did not have any major impacts on the computational burden of the methods, with run time and memory usage unaffected by different levels of ρ(𝒢). Overall, these results show that violating the sparsity assumption, made by all methods, has a significant impact on their performance, as also shown in previous studies [50,51]. This assumption is necessary to obtain credible estimates when the number of samples is considerably lower than the number of variables we want to estimate.

### Negative interactions increase the difficulty of network inference

We then tested whether the ratio of negative interactions had an effect on the methods’ inference ability. In this work, a negative interaction indicates competition for resources, as predator-pray interactions are not implemented in the simulator. To this end, we ran simulations with n=100, ρ(𝒢)=5%, p=50, K=100% and varied the ratio of negative interaction $r_{neg}\in [0,0.05,0.1,0.2]$. The complete results are reported in S4 Dataset; figures for selected scenarios of interest are available in S3 File (Figs AG–AL).

As we can see in Fig 8A, methods’ *MCC* declines as the negative ratio increases, with the largest drop observed for CCLasso, which goes from $\overset{―}{MCC}=0.52$ at $r_{neg}=0$ to $\overset{―}{MCC}=0.13$ at $r_{neg}=0.2$. Conditional dependence methods also experience a significant drop in *MCC*, with the average loss of $\overset{―}{\Delta MCC}=-0.31$. Bray-Curtis dissimilarity is the only method that increases in performance $\Delta \overset{―}{MCC}=0.11$, but we observe a high variance in the *MCC*, making it difficult to interpret the observed difference as a robust indication of improvement.

**Fig 8:**
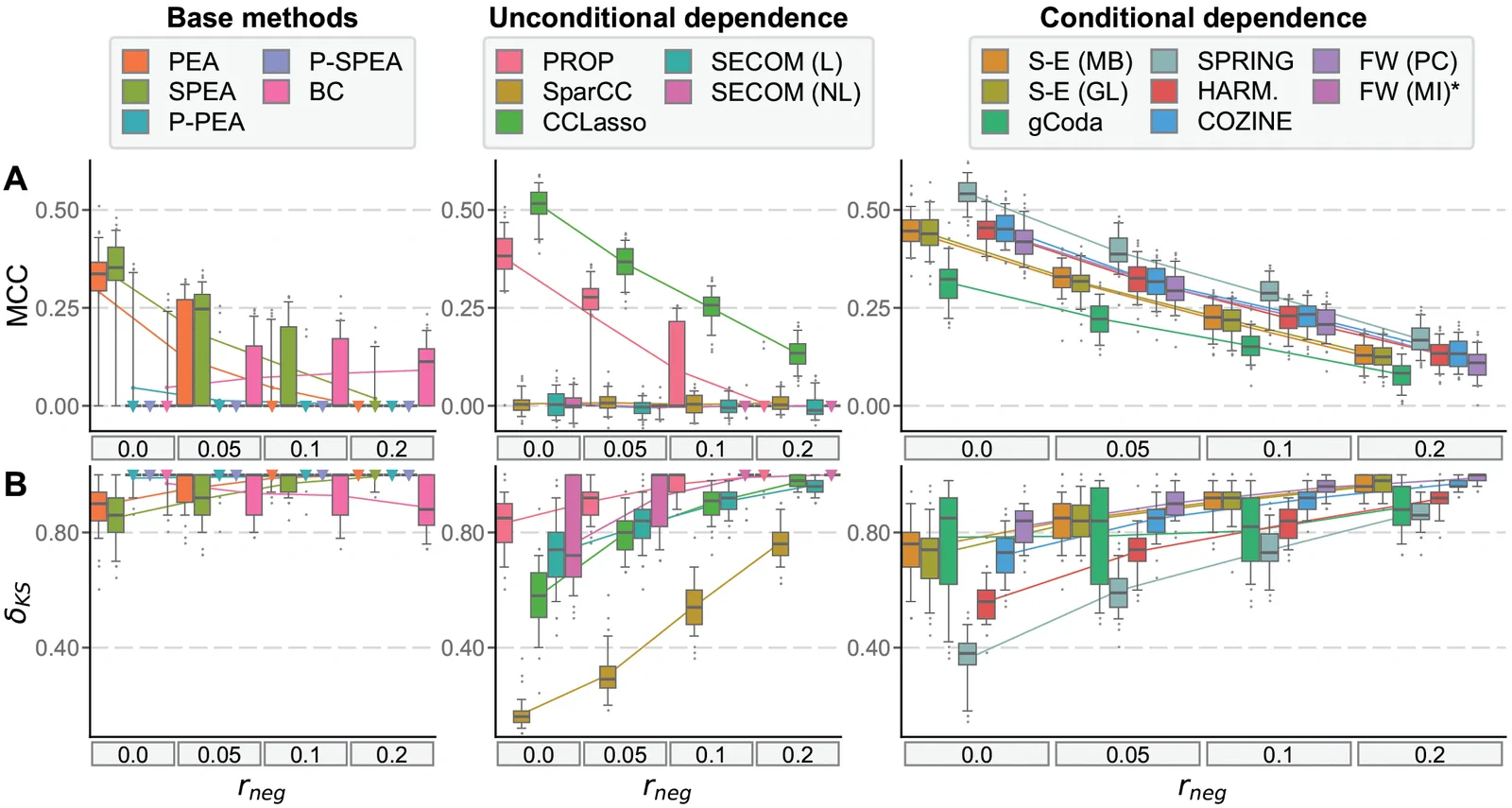
Methods’ performance as the negative interaction ratio increases. Here we show the results for the BA model with medium β−diversity between samples (see Figs AG–AL in S3 File for the other results). Data were simulated keeping all other parameters fixed at the default values ρ(𝒢)=5%, *p* = 50, *K* = 100%, *n* = 100. Methods are grouped based on the implementation strategy, and each column refers to one class of methods. Here we show 2 of the evaluation metrics: **A)** MCC and **B)** the Kolmogorov–Smirnov statistic $\delta_{KS}$. Methods marked with an asterisk had no successful runs over the 50 iterations for any of the scenarios in the figure.

In terms of node-wise (Figs AG, AI and AK in S3 File) and graph-wise metrics (Figs AH, AJ and AL in S3 File), the performance of the methods worsen with the increase in $r_{neg}$, suggesting that the inferred networks are structurally different from the true ones. As a representative in Fig 8, we can see that the degree distributions are considerably different, with $\overset{―}{\delta_{KS}}≅1$ for $r_{neg}=0.2$ for all methods.

The increase in negative interactions probably determines more complex population dynamics and causes interactions with opposite signs, incident to the same node, to cancel each other out and have little effect on the species count. Consider a 3-species network where species A has a mutualistic interaction with species B and a competitive interaction with species C. If the two interactions have the same magnitude, the net effect on the abundance of A will be null.

We found that the methods’ performance markedly decreased as the ratio of competitive interaction increased. This highlights the danger of relying solely on species abundance to infer interaction networks, as done by all co-occurrence inference methods, since the lack of an association between species’ abundances is no unequivocal evidence of the absence of direct interactions [81].

### Reduced library size degrades network inference performance

Due to the compositional nature of sequencing count data, the role of library size has been widely recognised as a crucial constraint in the analysis of sequencing count data, such as 16S rDNA-seq datasets [31], as well as for its impact on data sparsity. To investigate how reduced library size impacts the performance of co-occurrence-based methods, we simulated datasets with decreasing library size, meaning that we decreased the number of extractions (i.e., reads) by multiplying a factor K∈[100%,10%,2%,1%,0.5%] to the original library size obtained from a preset of real data [63]. As in previous sections, all other simulation parameters were kept fixed at their default value ($n=100,\;\rho (𝒢)=5\%,\;p=50,\;r_{neg}=0.05$). The complete results are reported in S5 Dataset; figures for selected scenarios of interest are available in S3 File (Figs AM–AT).

In Fig 9A, a noticeable reduction in $\overset{―}{MCC}$ is observable as the library size decreases, with a change from $\overset{―}{MCC}=0.18$ at *K* = 100% to $\overset{―}{MCC}=0.05$ at *K* = 1%. Interestingly, simulations conducted with high β-diversity among samples demonstrate a smaller reduction in *MCC* (refer to Fig AS in S3 File). Furthermore, both node-level (Figs AM, AO and AQ in S3 File) and graph-level (Figs AN, AP and AR in S3 File) performance declined as the number of reads decreased (i.e., decreasing value of *K*), indicating that the inferred networks deviate structurally from the true networks. As a representative in Fig 9B, we can see that the degree distributions are considerably different, with $\overset{―}{\delta_{KS}}≅1$ for *K* = 1% for all methods, with the only exceptions being SparCC ($\overset{―}{\delta_{KS}}=0.74$), which has $\overset{―}{MCC}=0$, and SPRING ($\overset{―}{\delta_{KS}}=0.7$).

**Fig 9:**
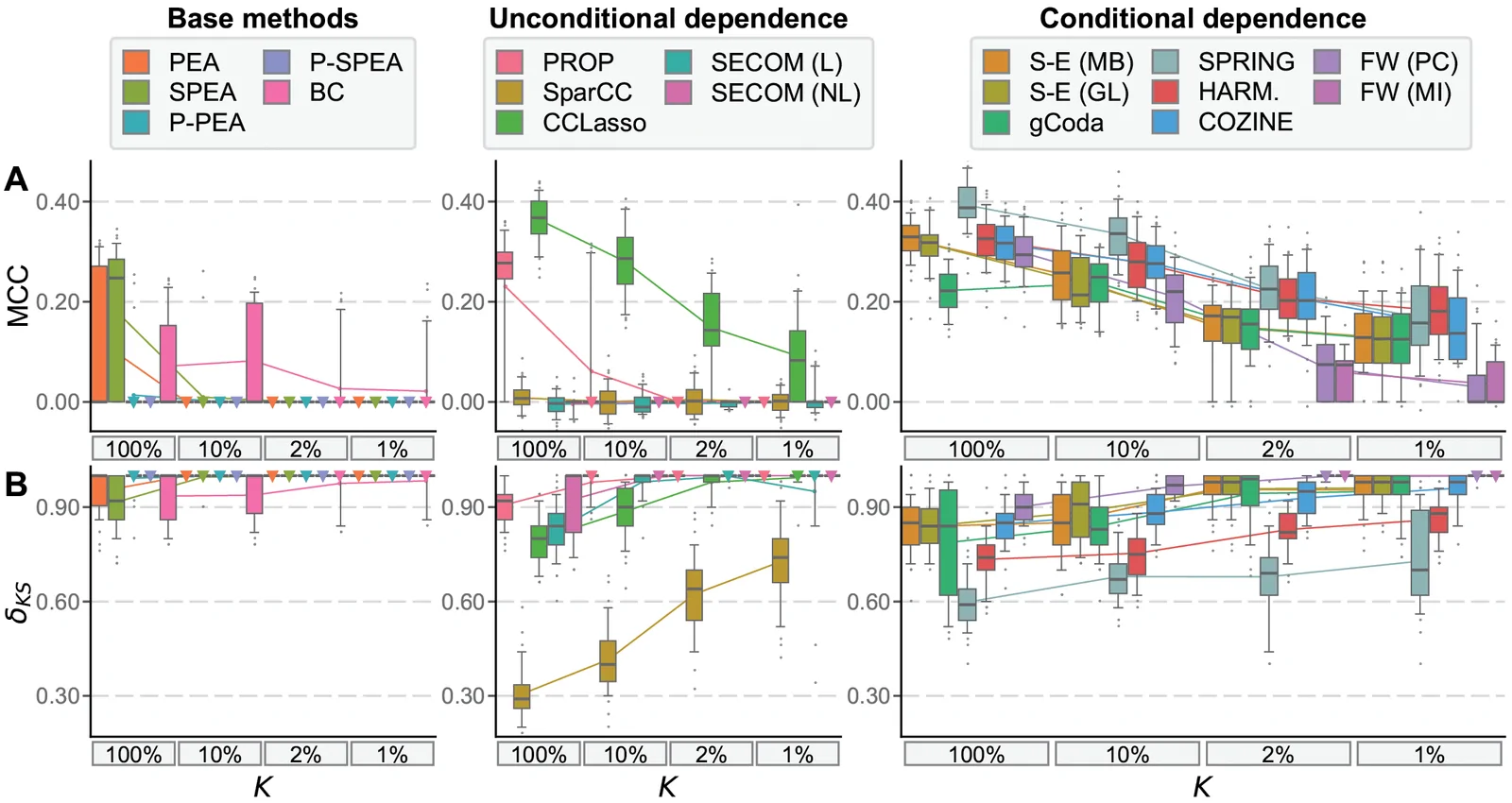
Methods’ performance as the library size decreases. Here we show the results for the BA model with medium β−diversity between samples (see Figs AM–AT in S3 File for the other results). Data were simulated keeping all other parameters fixed at the default values ρ(𝒢)=5%, *p* = 50, $r_{neg}=0.05$, *n* = 100. Methods are grouped based on the implementation strategy, and each column refers to one class of methods. Here we show 2 of the evaluation metrics: **A)** MCC and **B)** the Kolmogorov–Smirnov statistic $\delta_{KS}$.

Ultimately, these findings indicate that smaller library sizes substantially compromise the robustness of network reconstruction, causing both reduced predictive performance and structural deviations from the true underlying network. Consequently, the sampling depth emerges as a key design parameter in real-world studies aiming to achieve more reliable outcomes in microbial network inference.

## Conclusion

The study aimed to evaluate the performance of different co-occurrence inference methods to assess their applicability in real metagenomics studies to identify species associations. Considering previous valuable studies [48–52,56], we expanded the evaluation of inference methods to 18 different approaches from the literature, applied them to simulated datasets generated using a novel microbial community simulator [57] and thoroughly measured their performance by edge-, node-, and graph-wise metrics, as well as their computational burden. The use of simulated data allowed us to compare the methods’ inferred networks with the true network and to probe the impact of various parameters on the methods’ performance. We tested the methods in a variety of scenarios, comprising different graph generation models, level of diversity between samples, number of samples, number of species, network density, ratio of negative interactions, and library size.

Consistent with previous studies [48–52], the results show the limitations of co-occurrence methods in inferring microbial associations, with moderate to low performances observed in the majority of the simulated scenarios. The main limitation of the methods is their extremely low recall, meaning that they are able to detect only a small part of the species interactions. This recommends caution when using co-occurrence methods as exploratory tools to identify potential associations to then investigate further, as key interactions may be missing from the inferred network.

Different network topologies, generated with different graph generation models, have a significant impact on the methods’ performance. The methods obtained the best results with the Watts-Strogatz model (Fig 2), as found by other benchmarks [50,51]. For more complex and disordered topologies, derived from the Erdős–Rényi and Barabási–Albert models, the performance was consistently inferior. The specific challenge of recovering scale-free (Barabási–Albert) network structures, in particular, is consistently observed in many previous assessments [49–51].

The β−diversity between samples also had an impact on the inferred co-occurrence network. Higher β−diversity between samples improved methods’ performance, a finding that deviates from what was previously reported [51]. This trend is likely due to resource perturbations mimicking environments with limited resources, thereby increasing dependency on metabolite by-products, thus strengthening the interaction effect on species co-occurrence.

Concerning specific simulation parameters, the methods showed an increase in performance as the number of samples increased (Fig 3), as expected and in agreement with previous studies [48,50,51,56]. Furthermore, we observed a drop in the methods’ performance when the number of species in the community increased (Fig 5), a trend less evident in previous studies where the number of species did not considerably affect performance [50,51]. However, rather than the absolute increase or decrease of these two parameters individually, their ratio appears to play a more important role: for most methods, performance tends to improve as the ratio of the number of samples to the number of features increases (Fig AU in S3 File). Thus, in a real-world setting, where the number of species ranges in the hundreds or thousands, the number of samples required is likely larger than the one usually collected by metagenomics experiments.

The results show that the main parameter that affects the performance of the methods is the true density of the network (Fig 7), as highlighted in previous works [50,51]. As density ρ(𝒢) increased, a sharp decrease in all metrics considered was observed for all methods to the point that for ρ(𝒢)=15% all methods lost any estimation power (MCC≅0 for all methods). This result is expected, as most co-occurrence methods rely on the assumption of network sparsity, and violating this assumption places the methods outside their theoretical range of applicability. However, it is important to note that sparsity is assumed primarily for computational convenience, rather than as a reflection of any known biological property of microbial interaction networks. Thus, to determine the applicability of these methods in real scenarios, further studies are needed to assess the true level of sparsity in real microbiota interaction networks.

Increasing the percentage of negative interactions, and thus the level of competition, significantly reduced the methods’ performance (Fig 8). Our results align with [52] regarding the limitations of inferring competitive versus mutualistic interactions, but contrast with [50], which reported better performance in competitive communities. In a competitive scheme, the lack of associations between species counts is no evidence of the absence of direct interaction. This constraint may account for the decreased performance of co-occurrence methods, and the results obtained under these conditions should therefore be viewed and interpreted carefully.

We observed a considerable decline in all metrics values for all methods as the library size decreased. We advise adopting all possible measures to prevent these effects: do not use too extreme sample multiplexing strategies, try to maximise the per sample library size, and apply data transformations (like Aitchison log-ratio transformations) to consider the compositional nature of the data and to mitigate the effect of the constant sum constraints.

In general, conditional dependence methods have higher and more consistent performance, with SPRING achieving the best results (consistent with the findings of the only previous study that included this tool [49]), across both the various scenarios considered (Fig 10A) and the different evaluation metrics (Fig 10B). Although it is true that in some occasions base methods, coming from classical correlation statistics, have comparable results with ad-hoc tools (as highlighted by previous studies [48,50,56]), we find that the scenarios where this happens are generally obtained with unrealistic simulation parameters. Another method that showed strong and stable performance was CCLasso, which ranked second in overall performance (Fig 10B) and was among the best performing methods in [50]. This is particularly noteworthy given that the other methods in the unconditional dependence category performed substantially worse and the fact that CCLasso had the lowest overall computational burden. It is also important to note that some methods can have good performance according to some metrics but low performance according to other metrics, showing the importance of using different evaluation metrics. This is particularly clear in the case of SparCC, which ranks highest according to graph-level metrics but performs worst on edge-level metrics (Fig 10B). This indicates that, although the global topology of the inferred network closely matched that of the true network, the specific edges recovered were largely incorrect.

**Fig 10:**
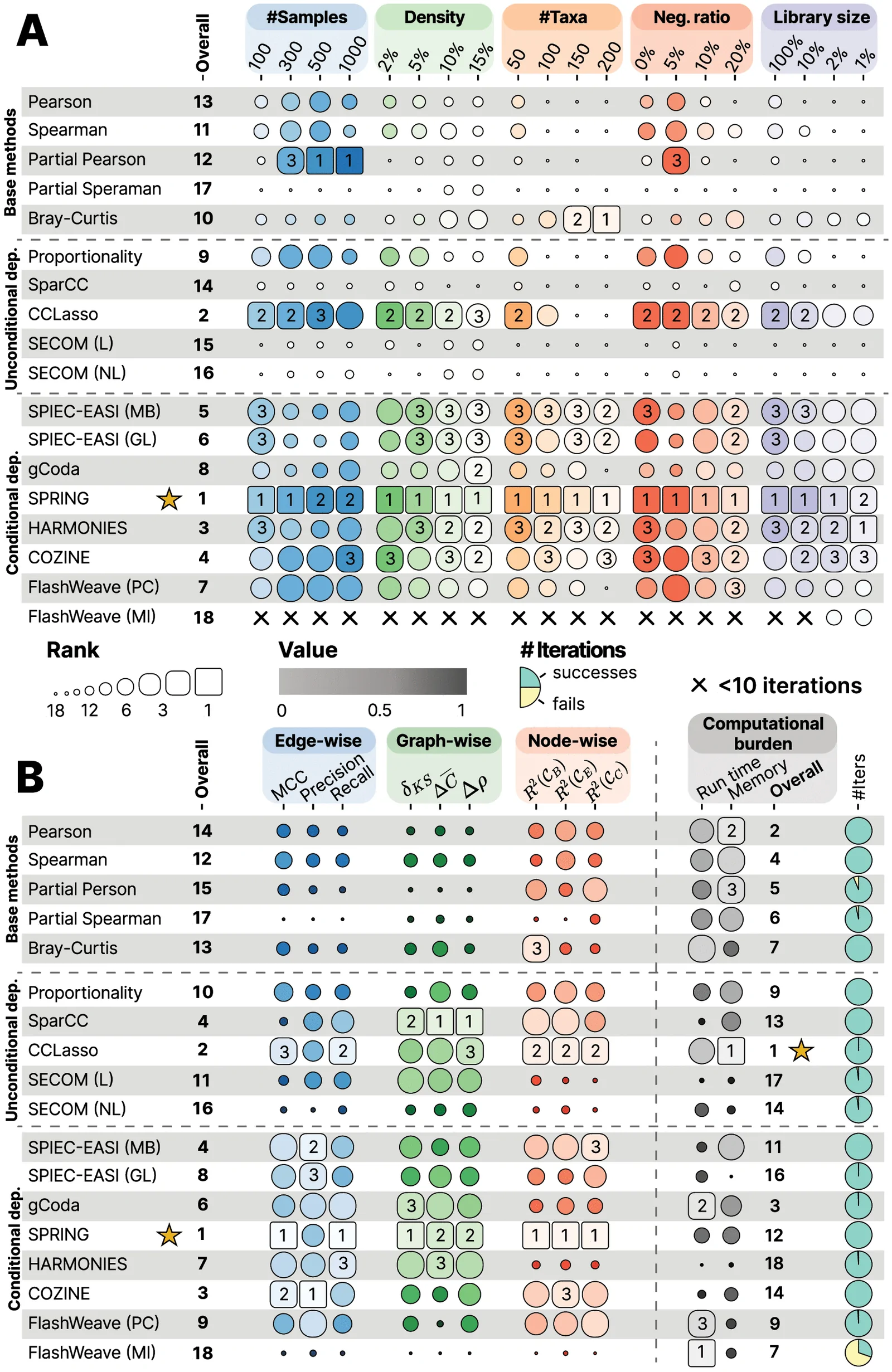
Overall performance of each inference method. A summary of the methods’ performances at various simulation parameters and according to the different metrics considered in the study. **A)** Mean MCC value of each method for different simulated scenarios, each column is a different scenario and the column name indicates the parameter that is different from the default ones (see Table 1). The mean was computed over the successful runs out of the 50 simulations with the same parameter values. The size of the dots represents the rank of the method within each column, with a numeric overlay for the top 3 ranks (rank 1 indicates the best performer), the colour intensity represents the absolute value of the mean MCC. The overall rank column was computed by averaging the ranks across each row. **B)** The overall rank of the inference methods according to different metric classes: edge-wise, graph-wise, node-wise and computational burden. For each metric we computed the methods’ ranks in each of the 189 simulated scenarios and then obtained the final rank for that metric as the average of all the ranks. The overall rank column was obtained by averaging the ranks for each method across the edge-wise, graph-wise and node-wise metrics. Similarly, for the computational burden we computed the methods’ ranks (in this case lower values have higher rank) in each of the 18 scenarios where the computational burden was measured, and then obtained the final rank for that metric as the mean of all the ranks. The overall rank in terms of computational burden was computed by averaging the ranks for each method across the computational burden metrics. Both the dots size and colour intensity represent the rank of the method within each column, with a numeric overlay for the top 3 ranks (rank 1 indicates the best performer). The last column displays the number of successful iterations over all the 9450 datasets.

Among the methods previously included in other benchmarking studies, SparCC, SPIEC-EASI and CCLasso were evaluated in [50], while SPRING, gCoda, SparCC and SPIEC-EASI were evaluated in [49]. Our edge-wise results are consistent with their findings: SparCC generally exhibits lower performance than SPRING, CCLasso and SPIEC-EASI, which are consistently among the top performing methods when included in the assessment. The remaining methods (SECOM, HARMONIES, COZINE, and FlashWeave) were not included in previous benchmarking studies. In our study, COZINE ranks third and HARMONIES falls within the top half of the rankings. These results suggest that more recent methods, developed specifically for microbial network inference, have achieved substantial advances. An exception was SECOM, a method that models 16S rDNA-seq biases. It should be noted that the performance we observe may stem either from the lack of filtering of input data, a step present in SECOM but which was turned off in this study to ensure comparable results with the other methods, or from the fact that some of the tool’s parameters should be tuned to the specific application context, which was not done in this study for any of the methods. In summary, when comparing our edge-wise results with previously published studies [49,50] that relied on alternative simulation strategies, we observed substantial agreement in the partial ranking of the methods (see Section 1 and Fig A in S3 File). However, we emphasise that this provides only indirect evidence and does not serve as a complete validation of our overall 18-method ranking, and that method ranking may vary across data-generation frameworks. While several main trends identified in our analysis, such as the effects of specific data characteristics [50–52] and the comparability of base methods with ad-hoc tools [48,50,56], were largely consistent with previous findings, further investigations that systematically assess how simulation frameworks influence inference results remain an important direction for future research.

Although this benchmark offers an extensive assessment of existing microbial network inference tools, additional investigation may still be necessary. For example, the use of simulated data could be rightfully questioned. While synthetic datasets are indispensable for providing a known ground truth against which to measure performance, they inherently involve a degree of oversimplification. However, it is important to note that this study addresses some of these gaps by employing a significantly more sophisticated simulation framework than those used in previous benchmarks. Furthermore, even if simulated data may not capture the full complexity of real datasets, we argue that these results provide an optimistic assessment of method performance under the controlled simulation settings used here. Another critical factor in network inference is the impact of data preprocessing, particularly normalisation, filtering, and imputation. In other areas of high-throughput “omics” analysis, it is well established that data preprocessing can strongly influence downstream findings. However, its precise impact on inferred microbial networks was only partially investigated by a valuable study [52], and additional investigation is required. Future studies should investigate the impact of data preprocessing, and in particular imputation and filtering, on the performance of network inference methods. Finally, the impact of parameter tuning represents a significant and non-trivial variable in the performance of inference methods. Preliminary tests conducted during this study, which involved standardising “similar” parameters across different methods, revealed only minor performance changes. An exhaustive exploration of the parameter space, which is beyond the current scope, is complicated by the fact that different algorithms expose different parameters often with different meaning. Future research into automated hyperparameter optimisation specifically tailored for microbial networks would be a valuable asset to the community.

In conclusion, we found that co-occurrence inference methods achieve acceptable results only in a narrow field of application: when the number of samples is large (considerably higher than the number of species) with reasonable library size, low network density, and a limited proportion of negative interactions. Unfortunately, conditions commonly encountered in empirical microbiome studies typically fall outside this field of applicability, for example, with the number of species in the network often being orders of magnitude higher than the number of samples available. Arguably, co-occurrence methods may obtain more reliable results when applied to an environment in which metabolite exchange between species is the principal factor determining species abundance (which in turn increases sample diversity). In contrast, in nutrient-rich environments where externally supplied resources constitute a major confounding factor, i.e., interaction patterns arising from microbial production–consumption processes are masked by the high availability of external resources, co-occurrence methods are substantially less effective. These results suggest that co-occurrence matrices are probably not sufficient to infer species interactions at a satisfactory level and underscore the fact that the problem of recovering the ecological network of a community is a non-trivial task. This does not undermine the importance of the considered methods, but highlights the need for careful investigation prior to real-world applications, the use of current methods in their narrow field of application, and the need for further methodological and computational developments in the ecological interaction network inference field.

## Supporting information

S1 File Simulation details. A comprehensive description of the procedure used to simulate the dataset. We provide all the parameters used in the simulation and their explanation. We also include a comparison of the simulated data with real datasets.
(PDF)

S2 File Inference methods details. A detailed list of the inference methods tested. We provide a brief description of each method and the mathematical assumption made to infer the species association network. We also include a limited sensitivity analysis on the effect of the methods parameters.
(PDF)

S3 File Additional results and figures. A comparison with previous benchmarking studies. Additional figures and results of the benchmarking study.
(PDF)

S1 Dataset Methods’ performance at the varying of the number of samples. (XLSX)

S2 Dataset Methods’ performance at the varying of the network density. (XLSX)

S3 Dataset Methods’ performance at the varying of the richness. (XLSX)

S4 Dataset Methods’ performance at the varying of the competitive interactions ratio. (XLSX)

S5 Dataset Methods’ performance at the varying of the library size. (XLSX)

S6 Dataset Computational burden profiling results. (XLSX)
