# Supplementary material for: From 16S rDNA-seq count data to microbial interactions: A comprehensive benchmark of co-occurrence network inference methods
Source: PLoS Comput Biol. 2026 Oct 29;22(10):e1014898. doi: 10.1371/journal.pcbi.1014898 (PMC13750663; doi:10.1371/journal.pcbi.1014898)
Supplement: Supplementary file 1 [file pcbi.1014898.s001.pdf]

Simulation details

Piero Mariotto[1], Matteo Baldan[1], Barbara Di Camillo[1,2,3], and Giacomo Baruzzo[1,*]

[1]Department of Information Engineering, University of Padova, Padova (PD), Italy.
[2]Department of Comparative Biomedicine and Food Science, University of Padova, Legnaro (PD), Italy.
[3]Padua Center of Network Medicine, University of Padova, Padova (PD), Italy.
[*]Corresponding author (giacomo.baruzzo@unipd.it)

1 Simulation framework

In this document, we provide a full explanation of the simulations performed to obtain the data used in the main article. We will go over each step of the N2SIMBA simulator as presented in the original paper (**Baldan et al. 2026**) and we will mention any modification made for this work, see **Figure A** for a diagram of the simulation pipeline. The software used is freely available in containerised form at `https://gitlab.com/sysbiobig/net_inf_bench` and `doi.org/10.5281/zenodo.18482664`.

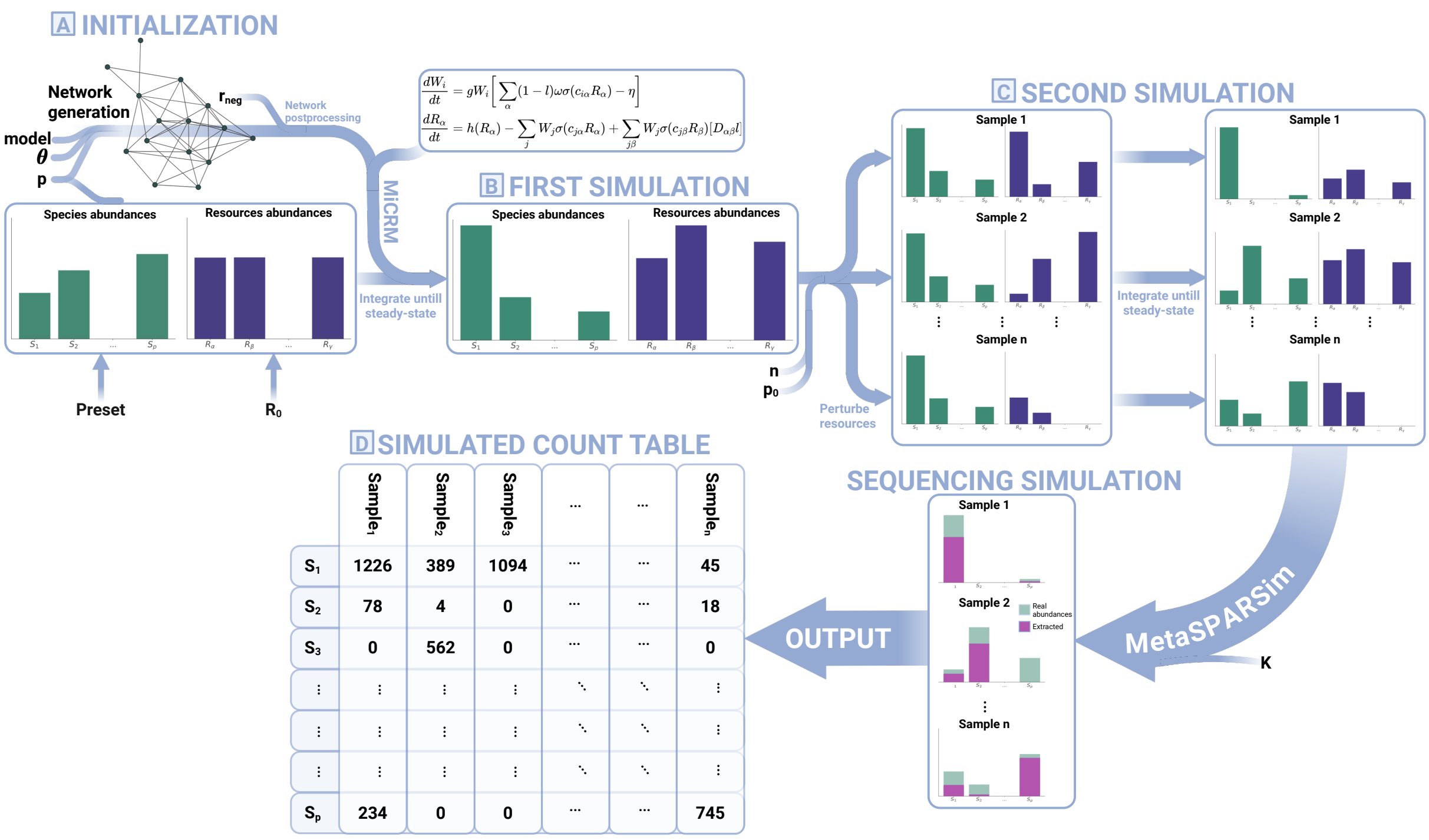

Fig A – Simulation pipeline. *A schematic representation of the main simulation steps. A) First, the community is initialized from a preset of real sequencing data and the interaction network is generated. B) The community is then propagated over time until a steady state is reached. C) To obtain different biological samples, the resources supply rates are perturbed randomly, and each sample is propagated in time to a new steady state. D) Finally to simulate sequencing, a multivariate hypergeometric model is applied to the simulated abundances obtaining a compositional count matrix as output. Image created in BioRender. MARIOTTO, P. (2026)* `https://BioRender.com/l42j184`

Each simulated dataset used in the main article is reproducible using the same simulation parameters and a numeric identifier (used to set `random.seed` and `np.random.seed`). By default, the identifier is generated using the Unix epoch timestamp (using `time.time`), but can also be provided by the user. Each simulated dataset stores the identifier used to generate the data, which can be provided in the input to the simulator to reproduce the results.

The first step in the simulation pipeline is the definition of a graph encoding the interaction pattern between the p species of the community. Since the interaction network topology of real communities is not known, we used 3 different graph generation algorithms (**Figure B**):

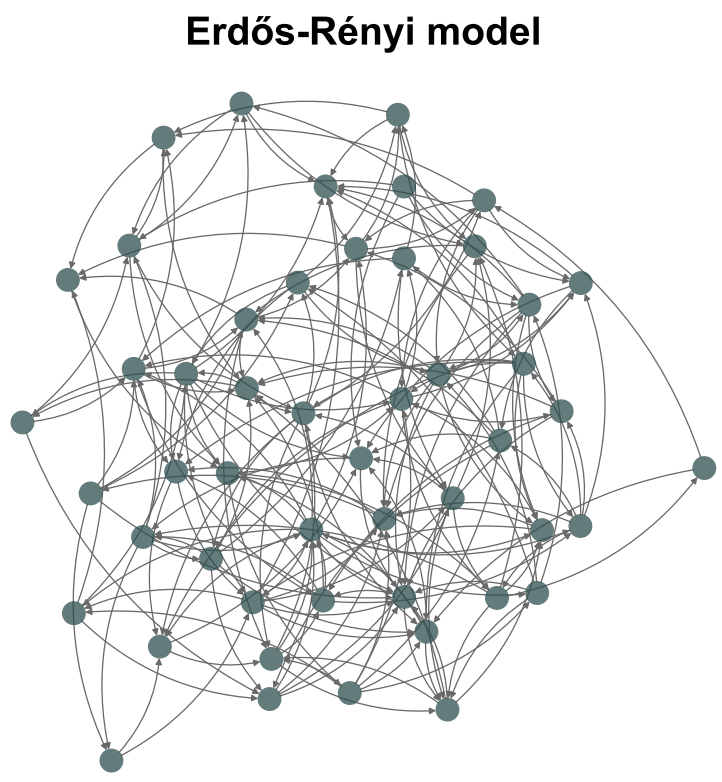

Fig B – Graph generation models. *An example of the three different generation algorithms. Each graph has the same number of nodes $p = 50$ and the same density $\rho(\mathcal{G}) = 8\%$ and was plotted using the same node positioning algorithm kamada_kawai_layout() from the* NetworkX *(**Hagberg et al. 2008**) python package. The topological differences are clear, with the Erdős–Rényi model resulting in a random disposition of edges, the Barabási–Albert model having central hubs with high degree and the Watts-Strogatz model shows local neighbourhoods connected by "bridge-like" edges.*

- Erdős–Rényi (ER) model (**Erdös and Rényi 1959**): generates a graph without a regular structure, often referred to as a random graph. Given a number of nodes p and a probability $\phi \in [0, 1]$ consider for each of the $p(p-1)$ ordered pairs of nodes (i, j) a realisation X_n of the random variable $X \sim U(0, 1)$ if $X_n < \phi$ add the directed edge $(i \to j)$ to the graph. It is straightforward that the parameter ϕ determines the density of the generated graph $\rho(\mathcal{G}^d_{ER})$, which is defined as the number of edges $|E|$ divided by the number of edges in the fully connected graph $|E_{max}|$. Thus

$$\mathbb{E}[\rho(\mathcal{G}^d_{ER})] = \mathbb{E}\left[\frac{|E^d_{ER}|}{|E_{max}|}\right] = \frac{\mathbb{E}[|E^d_{ER}|]}{p(p-1)} = \frac{\sum \mathbb{E}[X_n \leq \phi]}{p(p-1)} = \frac{\phi p(p-1)}{p(p-1)} = \phi$$

  Note that since ϕ is a real number in $[0, 1]$ we can generate a directed graph with any desired density.

- Barabási–Albert (BA) model (**Barabási and Albert 1999**): uses the preferential attachment principle to generate a graph with a power law degree distribution $P(k) \sim k^{-\gamma}$, often denoted as scale-free networks. Given an integer $m \in \mathbb{N}^+ > 1$ start the generation from an undirected star graph $\mathcal{G}_{init}$ of m nodes and $m-1$ edges. Then at each time step $t_{i=m+1,\dots,p}$ add a new node i with m edges that link the new node to the nodes already present in the graph. The probability ϕ_j that the new node is connected to node j is proportional to the degree k_j of the node $\phi_j = \frac{k_j}{\sum_{v \in N} k_v}$, thus nodes of a high degree tend to acquire more connections as new nodes are added. Thus, the number of edges in the generated undirected graph is equal to $|E^u_{BA}| = |E_{init}| + m(p-m)$. As the BA model generates an undirected graph $\mathcal{G}^u_{BA}$ we then converted it to a directed graph. To this end, consider $|E^u_{BA}| - |E_{init}|$ i.i.d. random variables $X_n \in \{1, 2\}$ $\forall n \in \{|E_{init}|, \dots, |E^u_{BA}|\}$ with probability $P(X_n = 1) = \frac{2}{3}$ and $P(X_n = 2) = \frac{1}{3}$. Then each edge in the initial star graph is converted to bidirectional directed edges (for a total of $2(m-1)$ directed edges), while the remaining edges in the undirected graph $(i, j) \in E^u_{BA}$ are converted as follows:

- $(i \to j) \in E^d_{BA}$ if $Y = 0 \mid X_n = 1 \; \forall n \in \{|E_{init}|, \ldots, |E^u_{BA}|\}$
- $(j \to i) \in E^d_{BA}$ if $Y = 1 \mid X_n = 1 \; \forall n \in \{|E_{init}|, \ldots, |E^u_{BA}|\}$
- $(i \to j) \in E^d_{BA}$ and $(j \to i) \in E^d_{BA}$ if $X_n = 2 \; \forall n \in \{|E_{init}|, \ldots, |E^u_{BA}|\}$

With $Y \sim \text{Br}\left(\frac{1}{2}\right)$ independent of X_n, thus the conditioned probability $P(Y = 0 \mid X_n = 1) = P(Y = 0) \cdot P(X_n = 1) = \frac{1}{3}$ and similarly $P(Y = 1 \mid X_n = 1) = \frac{1}{3}$. Thus, the number of edges in $\mathcal{G}^d_{BA}$ is a random variable defined as

$$|E^d_{BA}| = \overbrace{2(m-1)}^{|E_{init}|} + \sum_{n=|E_{init}|}^{|E^u|} X_n = 2(m-1) + \sum_{v=m}^{p} \sum_{u=1}^{m} X_n$$

with expectation

$$\begin{aligned} \mathbb{E}[|E^d_{BA}|] &= \mathbb{E}\left[2(m-1) + \sum_{v=m}^{p} \sum_{u=1}^{m} X_n\right] = 2(m-1) + \sum_{v=m}^{p} \sum_{u=1}^{m} \overbrace{\mathbb{E}[X_n]}^{=\frac{4}{3}} \\ &= 2(m-1) + \frac{4}{3} m(p-m) \end{aligned}$$

Thus, the density of the generated directed graph is a random variable with expectation

$$\begin{aligned} \mathbb{E}[\rho(\mathcal{G}^d_{BA})] &= \mathbb{E}\left[\frac{|E^d_{BA}|}{|E_{max}|}\right] = \frac{\mathbb{E}[|E^d_{BA}|]}{p(p-1)} = \frac{2m - 2 + \frac{4}{3}m(p-m)}{p(p-1)} \\ &= \frac{4}{3}\frac{mp}{p(p-1)} - \underbrace{\frac{4}{3}\frac{m^2}{p(p-1)}}_{\approx 0} + \underbrace{\frac{2m}{p(p-1)}}_{\approx 0} - \underbrace{\frac{2}{p(p-1)}}_{\approx 0} \qquad \textit{with } m \ll p \\ &\cong \frac{4}{3}\frac{m}{p-1} \end{aligned}$$

As can be seen, the density of the generated graph depends on the parameter m and the number of nodes p. As $m \in \mathbb{N}^+$ it is not possible to obtain an arbitrary value for the graph density, unlike in the Erdős–Rényi model.

- Watts-Strogatz (WS) model[1] (**Watts and Strogatz 1998**): generates a graph that is highly clustered, like regular lattices, yet has small characteristic path lengths, like random graphs, a property often defined as "small-world". The generation algorithm starts from an undirected ring lattice $\mathcal{G}_{rl}$ of p nodes each connected to its $k \in \mathbb{N}^+$ nearest neighbours. Thus, assuming that k is an even number, the number of edges in the ring lattice is $|E_{rl}| = p\frac{k}{2}$. The undirected ring lattice is then converted to a directed graph with the same procedure used for the BA model, thus the expected number of edges in the directed graph is

$$\mathbb{E}[|E^d_{WS}|] = \mathbb{E}\left[\sum_{n=1}^{p\frac{k}{2}} X_n\right] = \sum_{n=1}^{p\frac{k}{2}} \mathbb{E}[X_n] = p\frac{k}{2}\frac{4}{3} = pk\frac{2}{3}$$

The expected density is thus

$$\mathbb{E}[\rho(\mathcal{G}^d_{WS})] = \mathbb{E}\left[\frac{|E^d_{WS}|}{|E_{max}|}\right] = \frac{\mathbb{E}[|E^d_{WS}|]}{p(p-1)} = \frac{2}{3}\frac{k}{p-1}$$

[1] Not implemented in the released version of N2SIMBA

Then given a probability of rewiring ζ (in our simulations fixed at $\zeta = 0.05$), consider a random variable $X_n \sim U(0,1)$ for each edge $(i \to j) \in E^d_{WS}$, if $X_n < \zeta$ rewire the edge to a random node in the graph. The rewiring decreases the characteristic path length of the graph, granting the small-world property, and since we don't change the number of edges in the graph it doesn't affect the expected graph density.

As written above, each algorithm can generate graphs at different density levels depending on the value of the input parameter. As real microbial ecological network densities are unknown, we simulated datasets with various levels of interaction network density. Considering the approximate value of the expected density for the BA model, we can easily find the parameters of the generation algorithm ϕ, m and k to obtain equal densities

$$\begin{aligned}&\mathbb{E}[\rho(\mathcal{G}^d_{ER})] = \mathbb{E}[\tilde{\rho}(\mathcal{G}^d_{BA})] = \mathbb{E}[\rho(\mathcal{G}^d_{WS})]\\ \implies &\phi = \frac{4}{3}\frac{m}{p-1} = \frac{2}{3}\frac{k}{p-1}\\ \implies &\frac{3}{4}(p-1)\phi = m = \frac{k}{2}\end{aligned}$$

Thus, given m we would set $k = 2m$ and then calculate ϕ which being a real number doesn't pose many issues. In reality, the expected density of the BA model is

$$\mathbb{E}[\rho(\mathcal{G}^d_{BA})] = \mathbb{E}[\tilde{\rho}(\mathcal{G}^d_{BA})] - \varepsilon \quad with \quad \varepsilon = \frac{4}{3}\frac{m^2}{p(p-1)} - \frac{2m}{p(p-1)} + \frac{2}{p(p-1)} > 0 \quad \forall m \in \mathbb{R}$$

Thus, given m and $k = 2m$ the density of the graph generated with the BA model will be slightly lower than that of the graph generated by WS. To obtain graphs with density levels as close as possible, we set

$$\phi = \frac{\mathbb{E}[\rho(\mathcal{G}^d_{BA})] + \mathbb{E}[\rho(\mathcal{G}^d_{WS})]}{2}$$

In **Table A** the generation parameters used for the three algorithms are listed to generate graphs with equal density levels (up to a small constant δ).

As part of graph generation, the user can specify the ratio of competitive interaction in the network r_{neg}. Given $|E|$ the number of edges $|E_{neg}| = |E| \cdot r_{neg}$ will be selected at random and the corresponding value in the adjacency matrix $A_{ij} = -1$. We generate graphs with different levels of competition $r_{neg} \in \{0, 0.05, 0.1, 0.2\}$.
After the graph is generated, a check is performed to ensure that each node has at least one positive inward edge $k_i^{in+} > 0,\ \forall i \in V$, to ensure the survivability of each species also in sparse interaction networks. Each edge is assigned a different resource and the interaction matrices $\mathbf{C}$ and $\mathbf{D}$ are constructed according to the generated interaction graph. The matrix $\mathbf{C}$ is a binary matrix where $c_{i\alpha} = 1$ if species i feeds on the resource α and zero otherwise. For matrix $\mathbf{D}$, given its binary form in which $D^{bin}_{\alpha\beta} = 1$ indicates that resource β is converted into resource α, each element is obtained by column sum normalisation $D_{\alpha\beta} = D^{bin}_{\alpha\beta} / \sum \mathbf{D}^{bin}_{\cdot\beta}$ (meaning that each resource β is converted equally to non zero resources in $\mathbf{D}^{bin}_{\cdot\beta}$).
The initial state of the system is obtained by sampling the initial abundance of the p species from a preset, containing the 16S-rDNA-seq counts of real experiments, and by setting the initial abundance of the m resources to $R_0 = 1000$. Then the dynamics are obtained with the Community Simulator (**Marsland et al. 2020a**) function `MakeConsumerDynamics()`

$$\frac{dW_i}{dt} = gW_i\left[\sum_\alpha (1-l)\omega\sigma(c_{i\alpha}R_\alpha) - \eta\right] \tag{1}$$

$$\frac{dR_\alpha}{dt} = h(R_\alpha) - \sum_j W_j\sigma(c_{j\alpha}R_\alpha) + \sum_{j\beta} W_j\sigma(c_{j\beta}R_\beta)\left[D_{\alpha\beta}l\right] \tag{2}$$

Table A – *Graph generation parameters used for the three generation algorithm to obtain graphs with different topologies but the same edge density (up to a small error δ). The values reported refer to graphs with $p = 50$ nodes.*

		Density $\rho(\mathcal{G}) \pm \delta$				
		**2%**	**5%**	**8%**	**10%**	**15%**
**Erdős–Rényi**	Parameter$=\phi$	0.0269	0.0537	0.080	0.1057	0.1555
	$\rho(\mathcal{G}) = \phi = \frac{\rho(\mathcal{G}_{BA})+\rho(\mathcal{G}_{WS})}{2}$	2.69%	5.37%	8.00%	10.57%	15.55%
**Barabási–Albert**	Parameter$=m$	1	2	3	4	6
	$\rho(\mathcal{G}) = \frac{2m-2+\frac{4}{3}m(p-m)}{p(p-1)}$	2.66%	5.31%	7.83%	10.26%	14.77%
**Watts-Strogatz**	Parameter$=k$	2	4	6	8	12
	$\rho(\mathcal{G}) = \frac{2}{3}\frac{k}{p-1}$	2.72%	5.44%	8.16%	10.88%	16.32%

The values reported are truncated at 2 decimal points for readability, thus there may be some discrepancy, due to rounding error, between the reported value of ϕ and the one obtained as the mean of the reported 2 decimal digits $\rho(\mathcal{G}_{BA})$ and $\rho(\mathcal{G}_{WS})$. As we can see the difference between the desired density and the actual one is modest, especially for the first 4 levels of density with $\delta < 1\%$, with $\max(\delta) = 1.32\%$ for the WS model at the 15% density level.

the parameters used for the simulations are available in **Table B**.

Table B – **Community Simulator parameters**. *A list of the model parameters and their values used in the simulations.*

Symbol	Description	Value [Units]
g	Conversion factor from energy uptake to growth rate	1 [1/energy]
l	Leakage fraction, fraction of inward resources released in the environment in the form of metabolism by-product	0.2 [unitless]
ω	Conversion factor form mass flux to energy flux	1 [energy/mass]
R_0	Initial abundance of resources	1000 [mass]
R_α^{eq}	Intrinsic equilibrium abundance of resource α	1000 [mass/volume]
τ	Timescale for externally supplied resource turnover	1 [time]
η	Minimal energy uptake for maintenance of species	1 [energy/time]
f_p	Percentage of perturbed resources	1 [unitless]
n	Hill coefficient for functional response	2 [unitless]
σ_{max}	Maximum input flux	5 [mass/time]

For all simulations, we set an external supply of resources $h(R_\alpha) = (R_\alpha^{eq} - R_\alpha)/\tau$ for all resources. For the single value function $\sigma(x)$, which encodes the relationship between resource availability and uptake rates, we used the Hill function

$$\sigma_{Hill}(x) = \frac{x^n}{1 + x^n/\sigma_{max}}$$

Given the initial condition and the dynamics of the model, the state of the system is propagated forward in time until a steady state is reached. At this point, the steady-state

resources were perturbed n times using different perturbation strategies (see **next section**). The idea is that the n perturbations represent the different biological conditions found in a real experiment when we obtain samples from various sites. The n perturbed communities are then propagated over time until a new steady state is reached. These abundances form the $p \times n$ matrix $\mathbf{W}$ that contains the final abundances of the species in the simulated samples n. To simulate the sequencing step, and thus obtain compositional data, N2SIMBA uses a Multivariate-Hypergeometric model as in **Patuzzi et al. 2019**. First, each column of the matrix $\mathbf{W}$ is linearly scaled to a constant sum, obtaining the matrix $\mathbf{Y}$. An equal sum across samples represents the standardised input material of each sequencing run (set by sequencing protocols) after targeted amplification of the 16S rRNA gene during library preparation. Each column/sample of the final 16S rDNA-seq count matrix $\mathbf{X}$ is obtained following a Multivariate-Hypergeometric model

$$\mathbf{X}_{\cdot k} \sim MHG(\hat{L}_k, \overbrace{[Y_{1k}, \dots, Y_{pk}]^T}^{\mathbf{Y}_{\cdot k}})$$

where $\hat{L}_k$ is the library size of each sample k derived from the preset library sizes as

$$L_k = L \cdot \frac{\sum_i^p W_i^0}{W_{tot}} \cdot K \tag{3}$$

where L are the original library sizes from the preset, W_i^0 are the initial abundances of the species (at $t = 0$), W_{tot} is the sum of all the abundances of species in the preset and K is a user-defined scale factor between 0 and 1, setting $K < 100\%$ the number of extractions will be smaller and thus the output count table will have stronger compositional effects and contain more zeros since rare species are not sampled (**Figure C**).

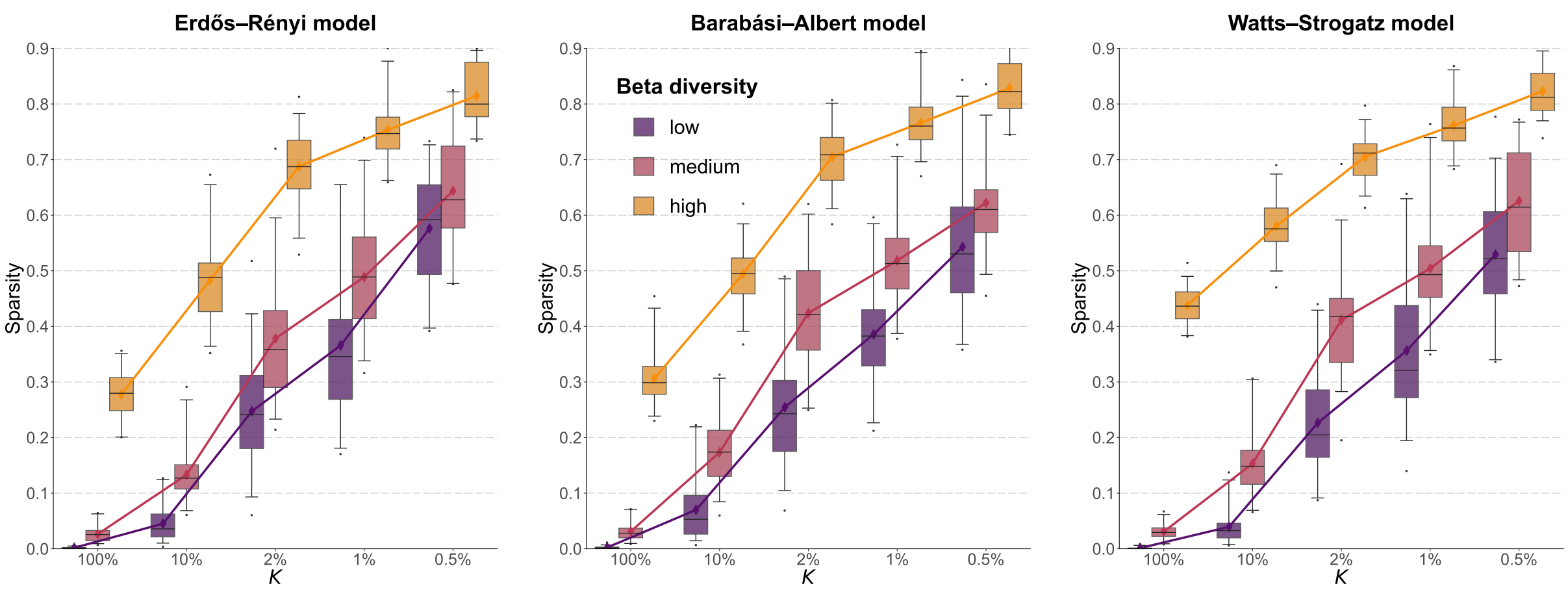

Fig C – Sparsity in the simulated datasets. *We can see the sparsity levels increase as the number of extractions diminishes, computed as the number of zeros divided by the total number of entries in the simulated 16S rDNA-seq count table. The three panels refer to the three different graph generation models and the different colours to different values of $\beta-$diversity between samples (see* **next section***). The other simulation parameters were set to the default values ($\rho(\mathcal{G}) = 5\%,\ n = 100,\ p = 50,\ r_{neg} = 0.05$).*

Following this procedure, we simulated 189 different scenarios with different combinations of parameters (see **Table C** for the different values used). We repeated the simulation for each scenario 50 times as there are some random processes in the model (like the preset sampling or resource perturbation), obtaining 9450 datasets. When simulating a scenario and changing one of the parameters, all other parameters were set to a default value (**bold** in **Table C**), this was done to later investigate the impact of that parameter on the inference methods' performance.

Table C – N2SIMBA parameters. **Highlighted** *values are the default parameters used when varying other parameters (i.e. when generating datasets with different levels of interaction network density we kept fixed* $r_{neg} = 0.05$*,* $K = 100\%$*,* $p = 50$*,* $n = 100$*)*

Parameter	Symbol	Values tested
Graph generation model	$\mathcal{M}$	ER, BA, WS
Diversity between samples	$\overline{\beta}$	low, medium, high
Network densities	$\rho(\mathcal{G})$	2%, **5%**, 8%, 10%, 15%
Number of samples	n	50, 75, **100**, 200, 300, 500, 1000
Number of species	p	**50**, 100, 150, 200
Negative interaction rate	r_{neg}	0, **0.05**, 0.1, 0.2
Library size fraction	K	**100%**, 10%, 2%, 1%, 0.5%

2 Perturbation strategies

As stated above to obtain n samples, steady-state resources are perturbed to obtain different biological replicates of the community. To perturb the community, we multiply the intrinsic equilibrium abundance vector of the resources $\boldsymbol{R^{eq}} = [R_1^{eq}, \dots, R_\alpha^{eq}, \dots, R_m^{eq}]$ by a random vector $\boldsymbol{r}_k \in \mathbb{R}^{m\times 1}\ \forall k \in [0, \dots, n]$.
For simplicity, we will illustrate the procedure to generate one random perturbation vector $\boldsymbol{r}$, in the simulations, the vector is generated n times for each sample. First, m values are drawn from a user-defined range $[\frac{1}{x}, \frac{1}{x-1}, \dots, \frac{1}{2}, 0, 1, 2, \dots, x]$, in our case $x = 10 \implies r_\alpha \in [\frac{1}{10}, 10]\ \forall \alpha \in [0, \dots, m]$, but the values within the range are not selected with equal probability. The value 0 has a user defined probability of occurring p_0 while the other values have probability $p_i = \frac{1-p_0}{2x-1}\ \forall i \in [\frac{1}{x}, \dots, \frac{1}{2}, 1, \dots, x]$. Then the intrinsic equilibrium abundance of each resource is multiplied by the corresponding element of vector $\boldsymbol{r}$ obtaining $\boldsymbol{R_k^{eq}} = \boldsymbol{R^{eq}} \cdot \boldsymbol{r}^T$. As p_0 increases, we get more and more zeros in $\boldsymbol{r}$, meaning that a large fraction of resources will have intrinsic equilibrium $R_{k,\alpha}^{eq} = 0$ and therefore will no longer be supplied externally to the community. This means that if $R_{k,\alpha}^{eq} = 0$ any resource α present in the community was supplied to the community during the initial simulation of the community or is the by-product of cell metabolism of some species present in the community.
Varying p_0 has major effects on community evolution. With low values of p_0, the majority of the resources required by the community are externally supplied, resulting in even growth for all species. In contrast, for higher values of p_0, some species die off, as the required resources are not supplied, leading to even more species extinction as more gaps are introduced in the metabolic paths required to produce certain resources. In this context, cross-feeding becomes fundamental for species survival, and we are likely to see stronger correlations between species abundances. We quantified the impact of p_0 on the simulated samples by measuring the relative differences in the composition of the species between the samples (often called β diversity), measured using the Bray-Curtis dissimilarity index (**Bray and Curtis 1957**).

$$\beta_{kj} = 1 - \frac{2\sum_{i=1}^{p} \min(X_{ik}, X_{ij})}{\sum_{i=1}^{p}(X_{ik} + X_{ij})}$$

and obtained median values of $\overline{\beta} \in [0.5, 1]$ with higher values of p_0 corresponding to higher values of β-diversity and thus stronger differences between samples (**Figure D**). We chose three values of $p_0 = [0.1, 0.5, 0.9]$ corresponding to low ($\overline{\beta} = 0.515$), intermediate ($\overline{\beta} = 0.667$) and high ($\overline{\beta} = 0.920$) median values of β-diversity (**Figure E**) and run all the simulation parameters configuration at the 3 levels of diversity.

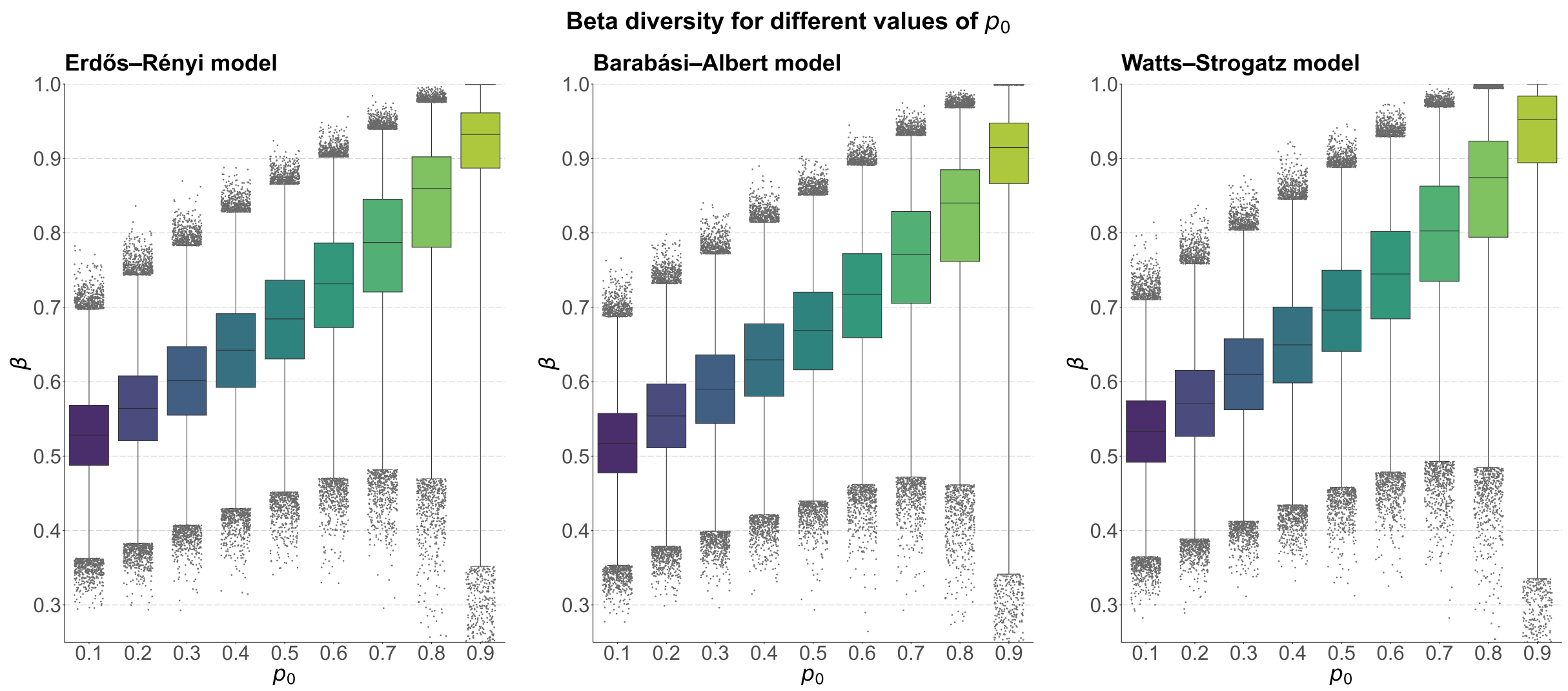

Fig D – Boxplots of β-diversity between samples, calculated using Bray-Curtis dissimilarity, at various levels of p_0. *As we can see the diversity increases with p_0 for all graph generation models. For each p_0 value we simulated 50 datasets with equal parameters ($p = 50$, $n = 50$, $\rho(\mathcal{G}) = 5\%$, $r_{neg} = 0.05$ and $K = 100\%$)*

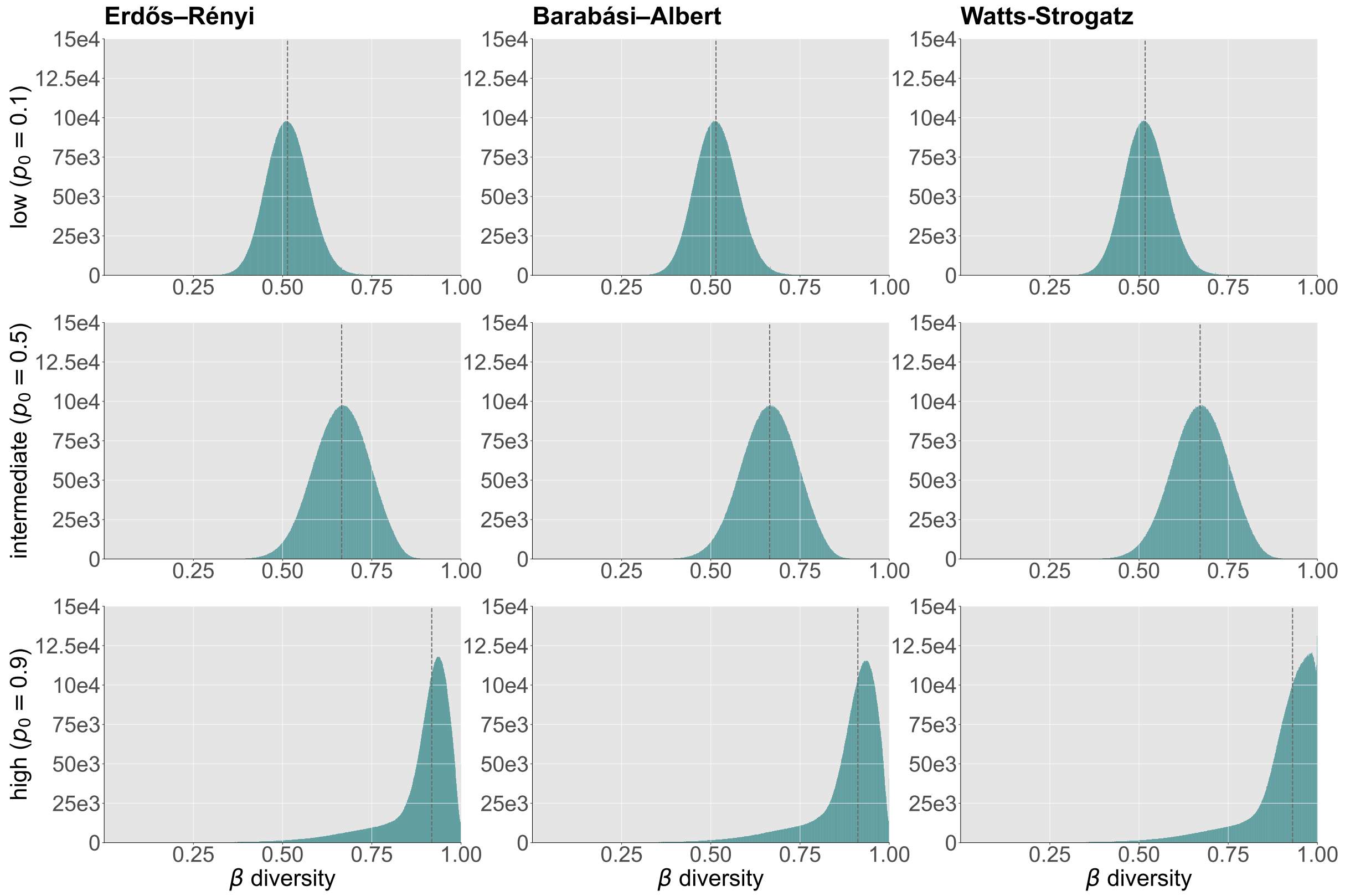

Fig E – Histograms of the β-diversity between samples, calculated using Bray-Curtis dissimilarity. *Each column referees to a different graph generation model and each row to a different value of p_0. The values were calculated on all simulated datasets.*

3 Comparison of simulated count tables with representative real datasets

An important aspect in benchmarking studies that use simulated data is the resemblance of the simulated data to experimental real world data on which the assessed methods are usually applied. Therefore, we assessed whether the simulated datasets reproduce several

well-characterised properties of real microbiome data and qualitatively compared the resulting trends with those observed in six empirical datasets (Table D). Based on characteristics previously investigated in the literature (**Marsland et al. 2020b**; **Grilli 2020**; **Ho et al. 2022**; **López et al. 2026**), we evaluated properties related to sparsity/prevalence, variability, and abundance, as well as the relationships among these features.

Table D – Real datasets. *Experimental microbial datasets used for comparing against data simulated using N2SIMBA. For each dataset is provided the number of taxa* ($\mathbf{p}$)*, the number of samples* ($\mathbf{n}$)*, the reference to the original publication presenting the data, a link to the resource used to download the dataset and an indication of filtering performed on the downloaded data.*

Name	p	n	Reference	Source	Rank	Filtering
MOMS-PI	90	224	**Osborne et al. 2022**	link	OTU	No filtering
GUT	128	195	**Yooseph et al. 2015**	link	Genus	No filtering
AMGUT1	127	289	**Kurtz et al. 2015**	link	OTU	No filtering
AMGUT2	138	294	**Kurtz et al. 2015**	link	OTU	No filtering
HMP1 Stool	7115	112	**HMP Consortium 2012**	link	OTU	No filtering
HMP2	959	178	**Lloyd-Price et al. 2019**	link	OTU	No filtering

In particular, we analyse:

- **Rank versus abundance** (Fig F), also known as species abundance curves, plots the number of species (y-axis) observed at specific abundance levels (x-axis). These curves are generally well fit by a Fisher log-series (**Fisher et al. 1943**; **Magurran 2005**), reflecting the inflation of rare species in these ecosystems. Comparing empirical and simulated counts using these curves helps assess the realism of the simulated count distributions (**Ho et al. 2022**; **López et al. 2026**).

- **Variability versus abundance** curves (Fig G) plot the mean abundance of each taxa against its variance. It has been shown that this curve follows Taylor's Law (**Grilli 2020**) meaning that the variance of a species scales as a power-law function of the mean population.

- **Prevalence versus abundance** curves (Fig H) display the taxa prevalence (i.e., the fraction of samples in which they are present) against their abundance. This relationship is known to be nonlinear, with more abundant taxa generally exhibiting higher prevalence (**Wang et al. 2024**), and it has been used in previous studies to compare simulated data with real one (**López et al. 2026**; **Ho et al. 2022**), and helps to assess if the sparsity and zero inflation structure of real datasets is captured by the simulation.

We examined these properties using 5 randomly selected datasets (using `np.random.choice(range(50), size=5, replace=False)` after setting a fixed seed `np.random.seed(42)`) out of the 50 generated under the default simulation settings: graph generation models ER, BA, and WS; network density $\rho = 5\%$; negative interaction rate $r_{neg} = 0.05$; β-diversity levels of low, medium, and high; sample size $n = 100$; and number of species $p = 50$. We evaluated several values of the library size fraction $K \in [100\%, 10\%, 2\%, 1\%]$ in our comparison, as it is known to directly affect sparsity and abundance.
Even before considering the simulated data, the empirical datasets showed substantial variability between the characteristics analysed (Fig F-H). All followed expected trends in rank–abundance and abundance–variability relationships, while prevalence patterns differed considerably between datasets.

The simulated datasets covered abundance ranges comparable to those observed empirically and reproduced the expected decreasing rank–abundance relationship (Fig F). However, it is important to note that simulations with low β-iversity deviated from the expected Fisher log-series at low abundances, suggesting that rare taxa were under-represented in that configuration.
The positive relationship between mean abundance and variance was also preserved in the simulated data (Fig G). Compared with the empirical datasets, the dynamic range of the mean abundance was covered considering the different value of the library size fraction. However, we note that the inference methods evaluated are invariant to the scale and range of mean abundance, as these characteristics are typically standardised through internal normalisation and transformation procedures.
The prevalence–abundance relationship was the most variable characteristic in both empirical and simulated datasets (Fig H). Simulations with low β-diversity, and some scenarios with medium β-diversity, tended to under-represent low-prevalence, highly sparse taxa, whereas high β-diversity simulations better reproduced the empirical prevalence range, although extremely sparse taxa remained under-represented.
A numerical comparison of the curves in terms of Mean Absolute Error (MAE) is reported in Figure I. Briefly, for each pairwise combination of real and simulated datasets, we first log-transformed the data to evaluate specific relationships (logseries, mean-variance, and mean-prevalence). We then fitted a smoothing spline using the `scipy.interpolate.make_smoothing_spline()` function. Finally, we evaluated the interpolated values on a regular grid of 100 points spanning the shared x-domain of both datasets and computed the error as

$$\text{MAE} = \frac{1}{\#points} \sum |y_1 - y_2|$$

Overall, this analysis suggests that some simulated datasets may not fully capture scenarios dominated by extremely low-abundance taxa with many zero observations. In practice, such taxa are often removed during preprocessing; when retained, however, they are likely to pose a greater challenge for network inference methods. In this respect, these simulated datasets may represent a somewhat easier inference setting than real microbiome data because they under-represent such challenging taxa.

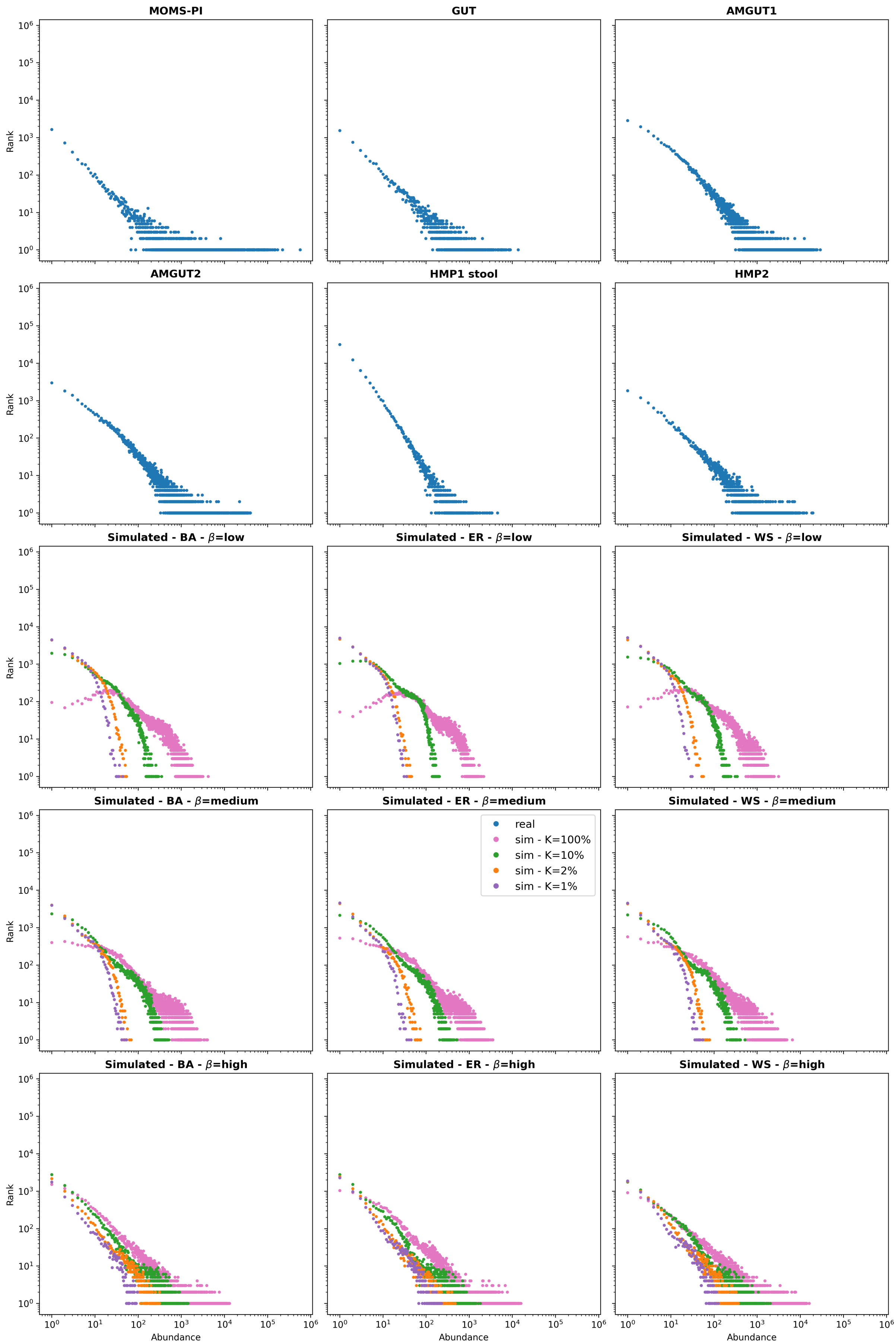

Fig F – Rank vs Abundance curves. *First 6 panels (with blue dots) show the real datasets. The bottom 9 panels are the simulated data in the BA, ER and WS topologies (1st, 2nd and 3rd columns respectively) with low, medium and high β-diversity values (3rd, 4th and 5th rows respectively). For the simulated data the different values of library size scale factor are shown with different colours: $K = 100\%$ in pink, $K = 10\%$ in green, $K = 2\%$ in orange and $K = 1\%$ in purple.*

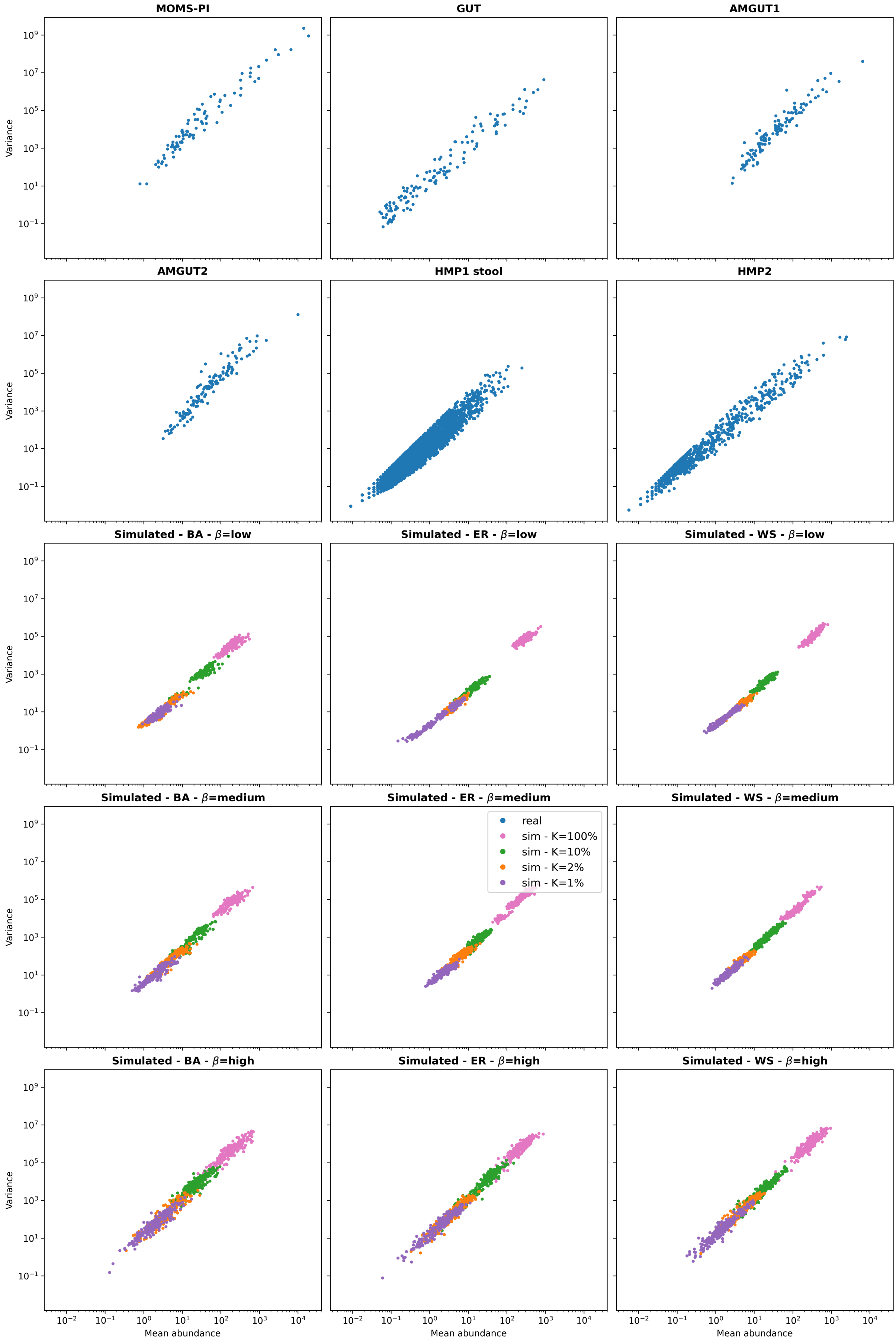

Fig G – Mean vs Variance curves. *First 6 panels (with blue dots) show the real datasets. The bottom 9 panels are the simulated data in the BA, ER and WS topologies (1st, 2nd and 3rd columns respectively) with low, medium and high β-diversity values (3rd, 4th and 5th rows respectively). For the simulated data the different values of library size scale factor are shown with different colours: $K = 100\%$ in pink, $K = 10\%$ in green, $K = 2\%$ in orange and $K = 1\%$ in purple.*

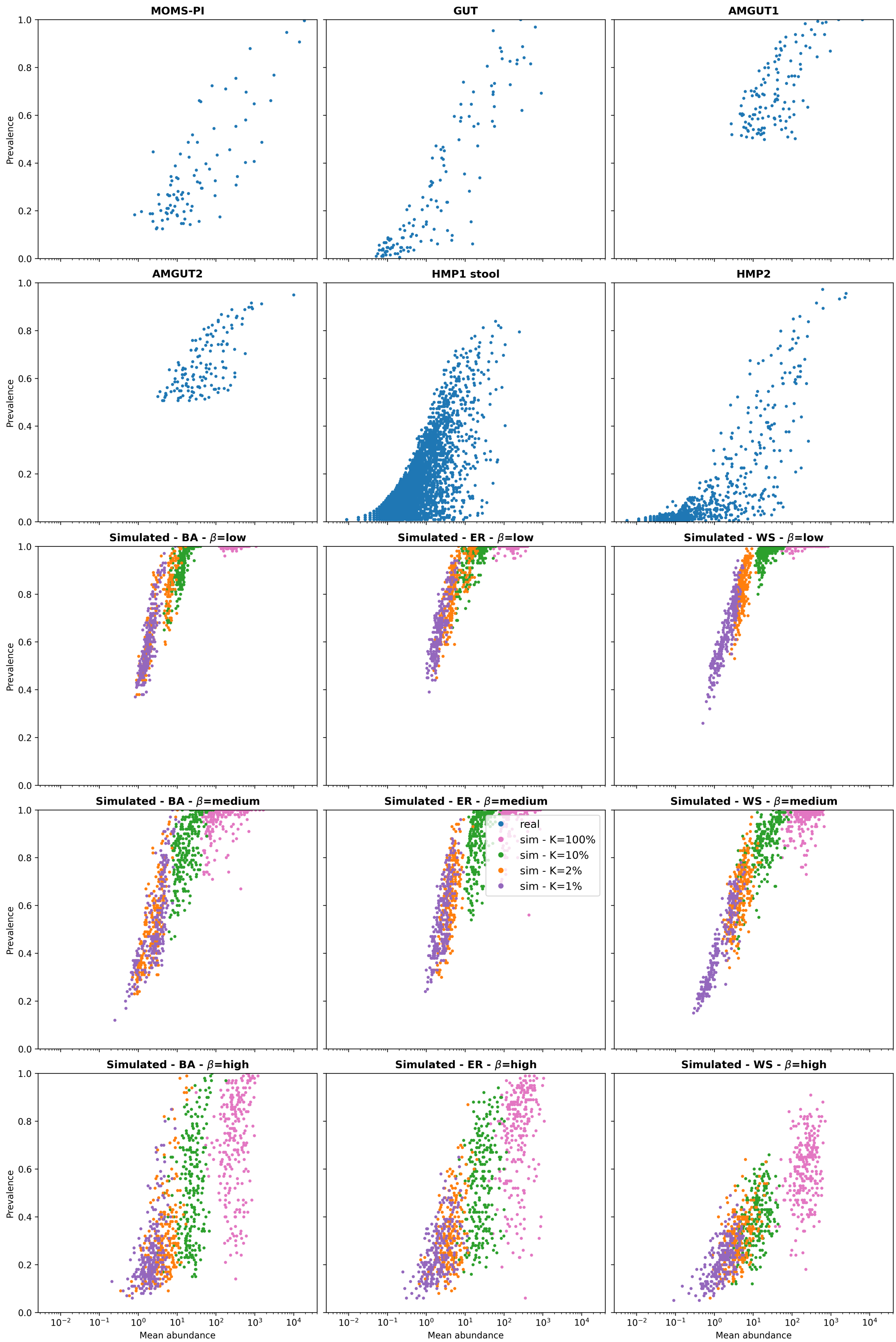

Fig H – Prevalence vs Mean curves. *First 6 panels (with blue dots) show the real datasets. The bottom 9 panels are the simulated data in the BA, ER and WS topologies (1st, 2nd and 3rd columns respectively) with low, medium and high β-diversity values (3rd, 4th and 5th rows respectively). For the simulated data the different values of library size scale factor are shown with different colours: $K = 100\%$ in pink, $K = 10\%$ in green, $K = 2\%$ in orange and $K = 1\%$ in purple.*

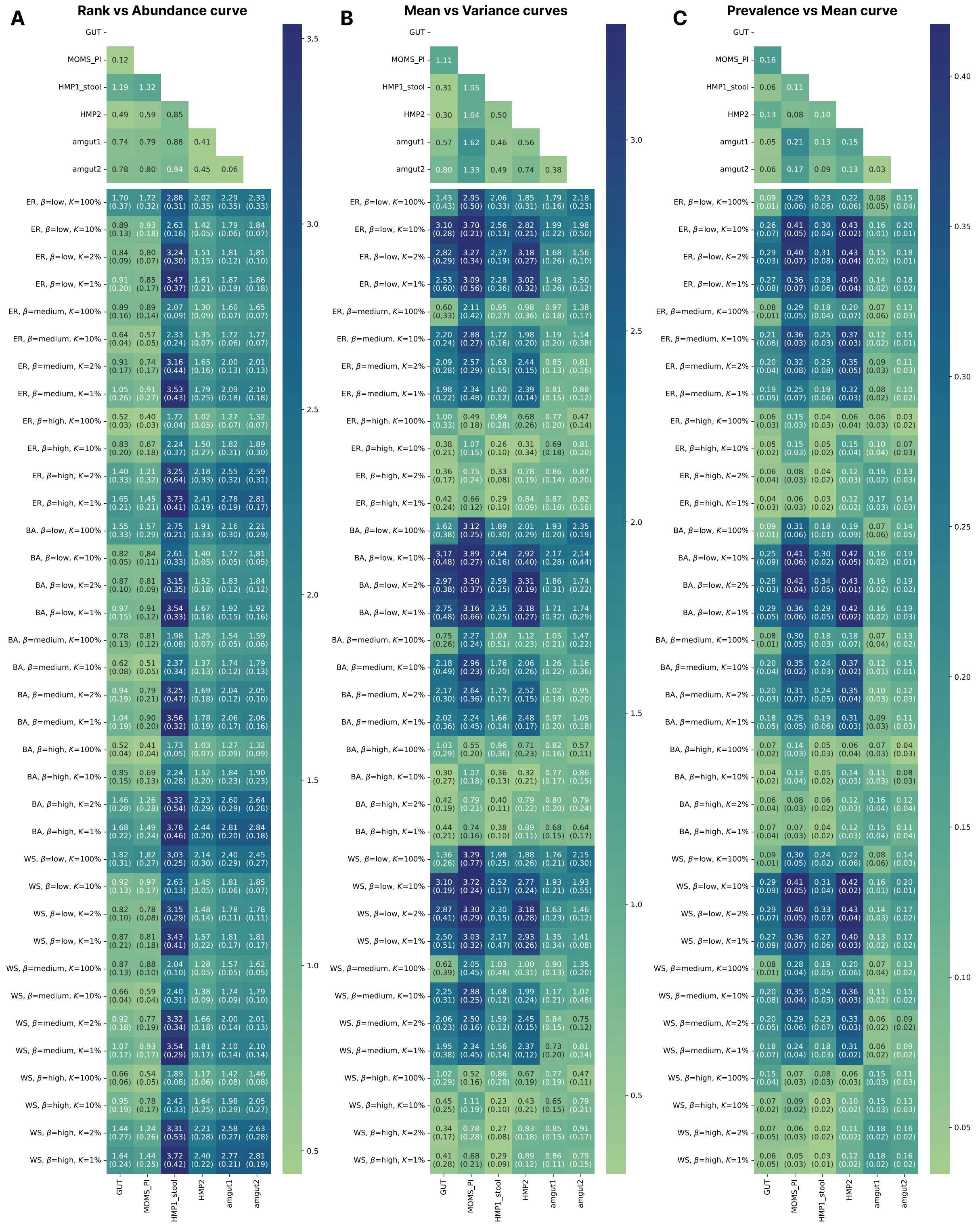

Fig I – MAE Heatmaps. *In each panel, the lower-triangular heatmap at the top shows the MAE values for all pairs of real datasets, serving as a reference baseline. The lower rectangular heatmap summarizes the MAE between each of the 6 real datasets and simulated datasets, grouped by graph model and β-diversity and library size scale factor, and reports the median together with the IQR (Inter-Quantile Range between the 25% and 75% quantiles). MAE values were computed on simulations with $p = 50$, $n = 100$, $\rho = 5\%$, $r_{neg} = 0.05$, and $K \in [100\%, 10\%, 2\%, 1\%]$, using all 50 iterations per parameter setting. Panel A presents MAE results for Rank vs. Abundance curves, panel B for Mean vs. Variance curves, and panel C for Prevalence vs. Mean curves.*

References

M. Baldan, G. Baruzzo, P. Mariotto, A. Rossato, M. Cappellato, and B. Di Camillo. N2SIMBA: from Network topology to SIMulation of interactions and BActerial abundance, using microbial consumer resource model. *Frontiers in Bioinformatics*, 6, 2026. ISSN 2673-7647. doi: 10.3389/fbinf.2026.1783447. URL https://www.frontiersin.org/journals/bioinformatics/articles/10.3389/fbinf.2026.1783447.

A.-L. Barabási and R. Albert. Emergence of Scaling in Random Networks. *Science*, 286(5439): 509–512, 1999. doi: 10.1126/science.286.5439.509. URL https://www.science.org/doi/abs/10.1126/science.286.5439.509.

J. R. Bray and J. T. Curtis. An Ordination of the Upland Forest Communities of Southern Wisconsin. *Ecological Monographs*, 27(4):325–349, 1957. doi: https://doi.org/10.2307/1942268. URL https://esajournals.onlinelibrary.wiley.com/doi/abs/10.2307/1942268.

P. Erdös and A. Rényi. On Random Graphs I. *Publicationes Mathematicae Debrecen*, 6: 290–297, 1959.

R. A. Fisher, A. S. Corbet, and C. B. Williams. The Relation Between the Number of Species and the Number of Individuals in a Random Sample of an Animal Population. *Journal of Animal Ecology*, 12(1):42–58, 1943. URL http://www.jstor.org/stable/1411.

J. Grilli. Macroecological laws describe variation and diversity in microbial communities. *Nature Communications*, 11(1), 2020. doi: 10.1038/s41467-020-18529-y. URL http://dx.doi.org/10.1038/s41467-020-18529-y.

A. A. Hagberg, D. A. Schult, and P. J. Swart. Exploring network structure, dynamics, and function using NetworkX. In G. Varoquaux, T. Vaught, and J. Millman, editors, *Proceedings of the 7th Python in Science Conference (SciPy2008)*, pages 11–15, Pasadena, CA USA, Aug 2008.

HMP Consortium. Structure, function and diversity of the healthy human microbiome. *Nature*, 486:207–214, 2012. doi: 10.1038/nature11234.

P.-Y. Ho, B. H. Good, and K. C. Huang. Competition for fluctuating resources reproduces statistics of species abundance over time across wide-ranging microbiotas. *eLife*, 11:e75168, 2022. doi: 10.7554/eLife.75168. URL https://doi.org/10.7554/eLife.75168.

Z. D. Kurtz, C. L. Müller, E. R. Miraldi, D. R. Littman, M. J. Blaser, and R. A. Bonneau. Sparse and Compositionally Robust Inference of Microbial Ecological Networks. *PLOS Computational Biology*, 11(5):1–25, 2015. doi: 10.1371/journal.pcbi.1004226. URL https://doi.org/10.1371/journal.pcbi.1004226.

J. Lloyd-Price, C. Arze, A. N. Ananthakrishnan, M. Schirmer, J. Avila-Pacheco, T. W. Poon, E. Andrews, N. J. Ajami, K. S. Bonham, C. J. Brislawn, D. Casero, H. Courtney, A. Gonzalez, T. G. Graeber, A. B. Hall, K. Lake, C. J. Landers, H. Mallick, D. R. Plichta, M. Prasad, G. Rahnavard, J. Sauk, D. Shungin, Y. Vázquez-Baeza, R. A. White, 3rd, IBDMDB Investigators, J. Braun, L. A. Denson, J. K. Jansson, R. Knight, S. Kugathasan, D. P. B. McGovern, J. F. Petrosino, T. S. Stappenbeck, H. S. Winter, C. B. Clish, E. A. Franzosa, H. Vlamakis, R. J. Xavier, and C. Huttenhower. Multi-omics of the gut microbial ecosystem in inflammatory bowel diseases. *Nature*, 569(7758):655–662, 2019.

R. C. López, J. A. Bonachela, M. G. Dominguez-Bello, M. Manhart, S. A. Levin, M. J. Blaser, and M. A. Muñoz. Imbalance in gut microbial interactions as a marker of health and disease. *Science*, 391(6788):890–895, 2026. doi: 10.1126/science.ady1729. URL https://www.science.org/doi/abs/10.1126/science.ady1729.

A. E. Magurran. Species abundance distributions: pattern or process? *Functional Ecology*, 19(1):177–181, 2005. doi: https://doi.org/10.1111/j.0269-8463.2005.00930.x. URL https://besjournals.onlinelibrary.wiley.com/doi/abs/10.1111/j.0269-8463.2005.00930.x.

R. Marsland, W. Cui, J. Goldford, and P. Mehta. The Community Simulator: A Python package for microbial ecology. *PLOS ONE*, 15:1–18, 2020a. doi: 10.1371/journal.pone.0230430. URL https://doi.org/10.1371/journal.pone.0230430.

R. Marsland, W. Cui, and P. Mehta. A minimal model for microbial biodiversity can reproduce experimentally observed ecological patterns. *Scientific Reports*, 10, 2020b. doi: 10.1038/s41598-020-60130-2.

N. Osborne, C. B. Peterson, and M. Vannucci. Latent Network Estimation and Variable Selection for Compositional Data Via Variational EM. *Journal of Computational and Graphical Statistics*, 31(1):163–175, 2022. doi: 10.1080/10618600.2021.1935971. URL https://doi.org/10.1080/10618600.2021.1935971.

I. Patuzzi, G. Baruzzo, C. Losasso, A. Ricci, and B. Di Camillo. metaSPARSim: a 16S rRNA gene sequencing count data simulator. *BMC Bioinformatics*, 20:416, 2019. doi: 10.1186/s12859-019-2882-6. URL https://doi.org/10.1186/s12859-019-2882-6.

T. Wang, A. Weiss, and L. You. A generic approach to infer community-level fitness of microbial genes. *Proceedings of the National Academy of Sciences*, 121(17):e2318380121, 2024. doi: 10.1073/pnas.2318380121. URL https://www.pnas.org/doi/abs/10.1073/pnas.2318380121.

D. J. Watts and S. H. Strogatz. Collective dynamics of 'small-world'networks. *nature*, 393 (6684):440–442, 1998.

S. Yooseph, E. F. Kirkness, T. M. Tran, D. M. Harkins, M. B. Jones, M. G. Torralba, E. O'Connell, T. B. Nutman, S. Doumbo, O. K. Doumbo, B. Traore, P. D. Crompton, and K. E. Nelson. Stool microbiota composition is associated with the prospective risk of plasmodium falciparum infection. *BMC Genomics*, 16(1):631, 2015.
