# Supplementary material for: From 16S rDNA-seq count data to microbial interactions: A comprehensive benchmark of co-occurrence network inference methods
Source: PLoS Comput Biol. 2026 Oct 29;22(10):e1014898. doi: 10.1371/journal.pcbi.1014898 (PMC13750663; doi:10.1371/journal.pcbi.1014898)
Supplement: Supplementary file 2 [file pcbi.1014898.s002.pdf]

Inference methods details

Piero Mariotto[1], Matteo Baldan[1], Barbara Di Camillo[1,2,3], and Giacomo Baruzzo[1,*]

[1]Department of Information Engineering, University of Padova, Padova (PD), Italy.
[2]Department of Comparative Biomedicine and Food Science, University of Padova, Legnaro (PD), Italy.
[3]Padua Center of Network Medicine, University of Padova, Padova (PD), Italy.
[*]Corresponding author (giacomo.baruzzo@unipd.it)

In this benchmark study, we evaluate some of the most relevant interaction network inference methods present in the literature. We selected 10 methods, for a total of 13 different implementations (as 3 methods: SPIEC-EASI, SECOM and FlashWeave offer 2 implementations each).
In this supplementary file, we give a brief description of the methods and of their mathematical assumptions. We describe the meaning of the parameters and report the values we used in this study. All the scripts used are available at https://gitlab.com/sysbiobig/net_inf_bench.

1 Unconditional dependence methods

Proportionality (Lovell et al. 2015) is a metric specifically developed to measure the dependence between compositional variables, based on the log-ratio variance $Var(\log x_i/x_j)$ proposed in Aitchison 1982. Here we used the proportionality correlation coefficient

$$\rho_p(\log x_i, \log x_j) = \frac{2Cov(\log x_i, \log x_j)}{Var(\log x_i) + Var(\log x_j)} = -\frac{Var(\log(x_i/x_j))}{Var(\log x_i) + Var(\log x_j)}$$

SparCC (Friedman and Alm 2012) was the first compositionally aware method developed to infer the interaction network of a microbial community. First, SparCC estimates the true relative abundance from the observed count matrix $\boldsymbol{X}$. Assuming unbiased sampling in the sequencing procedure, and a uniform prior, the posterior joint distribution of the true relative abundance random vector $\boldsymbol{v}$ is the Dirichlet distribution

$$p(\boldsymbol{v} \mid \boldsymbol{x}) = \text{Dir}(\boldsymbol{x} + 1)$$

The estimated fractions are set as a random sample from this posterior distribution. The method exploits the link between the variance of the log-ratio of components, named Aitchison variance $t_{ij} = Var\left(\log \frac{v_i}{v_j}\right)$, and the variance/covariance of the log-transformed basis $Var(\log w_i) = \sigma_i^2$, $Var(\log w_j) = \sigma_j^2$ and $Cov(\log w_i, \log w_j) = \rho_{ij}\sigma_i\sigma_j$

$$t_{ik} = \sigma_i^2 + \sigma_j^2 - 2\rho_{ij}\sigma_i\sigma_j$$

Under the assumption that the number of species is high and that the true correlation network is sparse $\hat{\rho}_i \cong 0$ SparCC estimates the basis variances $\hat{\sigma}_i^2$ which are then used to calculate the correlation coefficient as

$$\hat{\rho}_{ij} = \frac{\hat{\sigma}_i^2 + \hat{\sigma}_j^2 - t_{ij}}{2\hat{\sigma}_i\hat{\sigma}_j}$$

The method can be refined using an iterative procedure in which at each of the I_i iterations correlations are estimated as above, the components whose correlation exceeds a threshold are excluded (because they violate the assumption of average zero correlation) and the procedure

is repeated on the remaining components. To account for sampling noise, the inference procedure is repeated I_o times, each time with true relative abundance values $\boldsymbol{V}$ drawn randomly from their posterior distribution, generating a distribution of each pairwise correlation. The median value of each pairwise correlation distribution is taken as its estimated value. In the implementation used, the p-values of the correlation coefficients are obtained with a bootstrap procedure. SparCC is applied to B count matrices obtained by bootstrapping the original matrix and to B matrices obtained through permutation. The p-value is calculated as the fraction of times the correlation coefficient calculated on the bootstrapped matrices is more extreme than the median correlation coefficient calculated on the permutations. A list of the used parameters is available in **Table A**.

Table A – SparCC parameters

Parameters	Code	Description	Default
Outer iterations (I_o)	`iter`	SparCC whole procedure is repeated I_o times and median values are taken for correlation	20
Inner iterations (I_i)	`inner_iter`	Number of times the correlation coefficient are calculated, at each iteration species having correlation above th are excluded	10
Correlation threshold (th)	`th`	Correlation threshold for inner iterations	0.1
Number of bootstraps (B)	`R`	Number of bootstraps to calculate p-values	100

CCLasso (**Fang et al. 2015**) aims to estimate the log-basis vector covariance matrix $\boldsymbol{\Sigma}_{\log \boldsymbol{w}}$ from the covariance matrix $\boldsymbol{\Sigma}_{\log \boldsymbol{x}}$ obtained as the Pearson's sample covariance estimator applied to the log-transformed observed count matrix $\boldsymbol{X}$. Under the assumption that the observed counts are an equivalent composition of the real abundances, the authors use the relationship

$$\boldsymbol{F}_{clr}\boldsymbol{\Sigma}_{\log \boldsymbol{w}}\boldsymbol{F}_{clr}^T = \boldsymbol{F}\hat{\boldsymbol{\Sigma}}_{\log X}\boldsymbol{F}^T \quad (1)$$

As **Equation 1** is a rank deficient system (since $Rank(\boldsymbol{F}_{clr}) = p-1$ and $\boldsymbol{\Sigma}_{\log \boldsymbol{w}}$ is a $p \times p$ positive definite matrix) the authors estimate $\boldsymbol{\Sigma}_{\log W}$ by minimizing an inverse variance weighted loss function with an ℓ_1 penalty

$$\hat{\boldsymbol{\Sigma}}_{\boldsymbol{w}} = \underset{\boldsymbol{\Sigma}_{\boldsymbol{w}}>0}{\operatorname{argmin}} \frac{1}{2}\operatorname{tr}((\boldsymbol{F}_{clr}(\boldsymbol{\Sigma}_{\boldsymbol{w}} - \hat{\boldsymbol{\Sigma}}_X)\boldsymbol{F}_{clr}^T)(\boldsymbol{F}_{clr}\hat{\boldsymbol{\Sigma}}_X\boldsymbol{F}_{clr}^T)^{-1}(\boldsymbol{F}_{clr}(\boldsymbol{\Sigma}_{\boldsymbol{w}} - \hat{\boldsymbol{\Sigma}}_X)\boldsymbol{F}_{clr}^T) + \lambda\|\boldsymbol{\Sigma}_{\boldsymbol{w}}\|_1 \quad (2)$$

The optimisation problem in **Equation 2** is solved by an efficient algorithm based on the alternating direction method. To select the optimal λ value, CCLasso uses k-fold cross-validation. Briefly, for each λ value considered, one fold is used as a test set, on which the covariance matrix is estimated using the Pearson sample estimator $\boldsymbol{S}^{test}$, and the others as training. In the training folds $\boldsymbol{\Sigma}_{\log \boldsymbol{w}}$ is estimated using CCLasso, and the cross-validation error is calculated as $e_k^{CV} = \|\hat{\boldsymbol{\Sigma}}_{\log \boldsymbol{w}} - \boldsymbol{S}^{test}\|$. The final error is calculated as the cumulative cross-validation error in all folds $e_\lambda^{CV} = \sum_k e_k^{CV}$. The selection procedure starts with two arbitrary values provided by the user $[\lambda_{min}, \lambda_{max}]$ which are iteratively updated as in **Figure A** until a maximum number of iterations I_{max} are reached or the relative cumulative cross-validation error drops below a tolerance threshold $e_{\lambda_1,\lambda_2}^{CV} < 1e-4$. To calculate the p-values CCLasso repeats the estimation on B bootstrapped matrices, after correcting the mean correlation across bootstraps for random associations, the p-values are obtained with a Student t-test. A list of the parameters used is available in **Table B**.

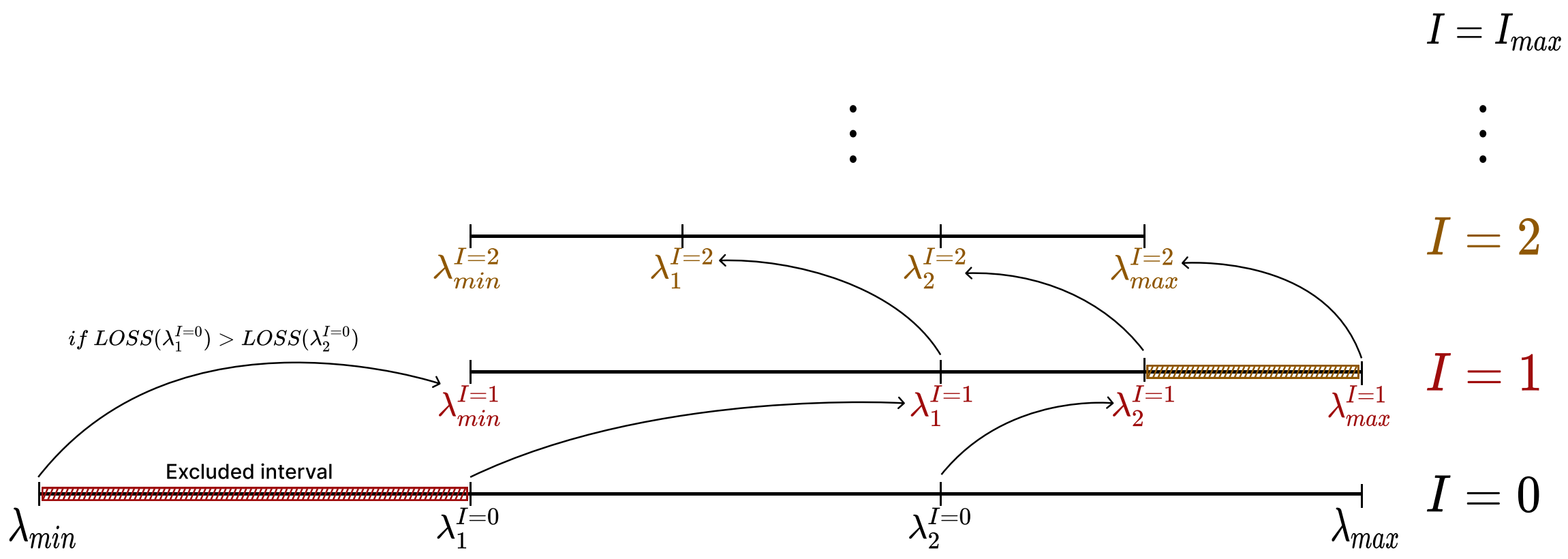

Fig A – CCLasso λ selection scheme. Illustration depicting the iterative procedure to select the optimal λ value.

Table B – CCLasso parameters

Parameters	Code	Description	Default
Maximum number of iterations (I_{max})	`k_max`	Maximum number of iterations to select the optimal λ value	20
Number of bootstraps (B)	`n_boot`	Number of bootstraps to calculate p-value	20
Lambda path $[\lambda_{min}, \lambda_{max}]$	`lam_int`	Correlation threshold for inner iterations	$[1e-4, 1]$

SECOM (Lin et al. 2022) is a recently developed method based on estimating the dependences between species after the count matrix $\boldsymbol{X}$ has been corrected for sample- and taxa-specific biases. It is known that the 16S rDNA-seq pipeline introduces several biases, like PCR saturation (Suzuki and Giovannoni 1996) and different primer affinity (Sipos et al. 2007) to name some, making the count matrix $\boldsymbol{X}$ a composition of the distorted real abundances and not an equivalent composition of the real abundances $\boldsymbol{W}$. SECOM assumes that biases are multiplicative $W_{ik} = s_k \cdot t_i \cdot X_{ik}$ and after correcting for the multiplicative factors $\hat{s}_k, \hat{t}_i$, measures the dependence between variables using two metrics: correlation coefficient (in this work we used Spearman correlation) and distance correlation coefficient (Székely et al. 2007). For each of the two metrics, SECOM gives in output an association matrix $\boldsymbol{R}$, a sparse association matrix $\boldsymbol{R}^S$ obtained by hard thresholding

$$R_{ij}^s = \begin{cases} R_{ij} & \text{if } R_{ij} > th \\ 0 & \text{if } R_{ij} \le th \end{cases}$$

and a confidence score for each association coefficient; for correlation, the p-value is calculated with a Student t-test, while for the distance correlation coefficient, the p-value is obtained through a permutation test. A list of the parameters used is available in Table C.

2 Conditional dependence methods

First, we would like to introduce some concepts that are widely used by conditional dependence-based methods. As stated in the main papers under the assumption of multivariate Gaussianity, two random variables are conditionally independent $x_i \perp\!\!\!\perp x_j \iff \Omega_{ij} = 0$ thus the estimation of

Table C – SECOM parameters

Parameters	Code	Description	Default
Correlation threshold (th)	`thresh_hard`	Correlation values $R_{ij} < th$ will be set to 0 in $\boldsymbol{R}^S$	0.3
Number of permutations (B)	`R`	Number of permutations to calculate p-value for distance correlation	1000
Correlation threshold choice	`soft`	If `soft=FALSE` use user-defined threshold th, if `soft=TRUE` use cross-validation to determine the threshold	FALSE
Prevalence threshold	`prv_cut`	Numerical threshold for filtering species with prevalences less than `prv_cut`	**0***
Library size threshold	`lib_cut`	Numerical threshold for filtering samples with library sizes less than `lib_cut`	**0***

*Filtering parameters set to custom defaults to ensure no filtering of the input data, actual defaults are `prv_cut=0.5` and `lib_cut=1000`

the precision matrix $\boldsymbol{\Omega}$, given n i.i.d.[1] observation, is sufficient to construct the independence graph of the p species. To estimate $\boldsymbol{\Omega}$ a Maximum Likelihood Estimation (MLE) can be used. The likelihood function, which expresses the likelihood that the given observations come from a Gaussian distribution with parameters $\boldsymbol{\theta} = \{\boldsymbol{\mu}, \boldsymbol{\Omega}\}$, can be written as:

$$\begin{aligned}
\mathcal{L}(\boldsymbol{\theta}|\boldsymbol{X}_{\cdot 1}, \dots, \boldsymbol{X}_{\cdot n}) &= \prod_{k=1}^{n} p(\boldsymbol{X}_{\cdot k}|\boldsymbol{\theta}) \\
&= \prod_{j=1}^{n} \left(\frac{1}{2\pi}\right)^{\frac{p}{2}} (\det \boldsymbol{\Omega})^{\frac{1}{2}} e^{-\frac{1}{2}(\boldsymbol{X}_{\cdot k}-\boldsymbol{\mu})^T \boldsymbol{\Omega} (\boldsymbol{X}_{\cdot k}-\boldsymbol{\mu})} \\
&= \left(\frac{1}{2\pi}\right)^{\frac{np}{2}} (\det \boldsymbol{\Omega})^{\frac{n}{2}} e^{-\frac{1}{2}\sum_{k=1}^{n}(\boldsymbol{X}_{\cdot k}-\boldsymbol{\mu})^T \boldsymbol{\Omega} (\boldsymbol{X}_{\cdot k}-\boldsymbol{\mu})}
\end{aligned}$$

To obtain a more tractable problem, we can take the logarithm and multiply by -1 to obtain the negative log-likelihood function. To estimate a sparse graph structure we can minimise the negative log-likelihood function adding an ℓ_1 penalty term

$$\begin{aligned}
\hat{\boldsymbol{\Omega}} &= \underset{\boldsymbol{\Omega}>0}{\operatorname{argmin}} \ \ell_{neg}(\boldsymbol{\theta}|\boldsymbol{X}_{\cdot 1}, \dots, \boldsymbol{X}_{\cdot n}) + \lambda\|\boldsymbol{\Omega}\|_1 \\
&= \underset{\boldsymbol{\Omega}>0}{\operatorname{argmin}} \ -\log(\det \boldsymbol{\Omega}) + \operatorname{tr}(\boldsymbol{S}\boldsymbol{\Omega}) + \lambda\|\boldsymbol{\Omega}\|_1 \qquad (3)
\end{aligned}$$

This convex optimisation problem can be solved efficiently using an algorithm proposed by **Friedman et al. 2007** called graphical lasso or Glasso.

Another method used to estimate the precision matrix of a multivariate normal distribution is the neighbourhood selection method introduced by **Meinshausen and Bühlmann 2006**, commonly known as the MB method. The method aims to individually estimate the neighbourhood[2] $N(i)$ of each node by solving an ℓ_1-regularized linear regression problem

$$\hat{\boldsymbol{\theta}}^i = \underset{\boldsymbol{\theta}\in\mathbb{R}^{p-1}}{\operatorname{argmin}} \|\boldsymbol{X}_{i\cdot} - \boldsymbol{X}_{\neg\{i\}\cdot}\boldsymbol{\theta}\|^2 + \lambda\|\boldsymbol{\theta}\|_1 \qquad (4)$$

determining the local neighbourhood of the node as $N(i) = \{j \subset \{1, \dots, p\} \mid \hat{\theta}^i_j \neq 0\}$. Once the regression coefficients have been calculated for all nodes, an edge is drawn between nodes

[1] independent and identically distributed

[2] as neighbourhood of a node i we intend the smallest subset of nodes so that given all the variables in the neighbourhood i is conditionally independent of all remaining variables.

i and j if $i \in N(j) \vee j \in N(i)$.
To select the optimal regularisation parameter λ, the majority methods use a stability-based method named StARS (**Liu et al. 2010**). StARS aims to use the least amount of regularisation that simultaneously makes a graph sparse and replicable under random sampling. In particular, StARS selects λ so that, with high probability, the real graph is contained in the estimated graph $\mathcal{G} \subseteq \hat{\mathcal{G}}$. The method works by drawing B subsamples $S_1, \ldots, S_B$ from the n observations. The graph is estimated in each of the subsamples along a lambda path $\Lambda = \{\lambda_{max}, \ldots, \lambda_0\}$. With $\psi^{\lambda}_{i,j}(S_\alpha) = 1$ indicating whether the graph estimation, based on subsample S_α, places an edge between nodes i and j. For each pair of nodes (i,j) we can estimate the stability $\hat{\tau}_{i,j}(\lambda)$ and the instability $\hat{\varphi}_{i,j}(\lambda)$ across subsamples as follows.

$$\hat{\tau}_{i,j}(\lambda) = \frac{1}{B}\sum_{b=1}^{B}\psi^{\lambda}_{i,j}(S_b)$$
$$\hat{\varphi}_{i,j}(\lambda) = 2\hat{\tau}_{i,j}(\lambda)(1-\hat{\tau}_{i,j}(\lambda))$$

The stability $\hat{\tau}_{i,j}(\lambda) \in [0,1]$ expresses the frequency edge (i,j) is estimated in the graphs B. In contrast, the instability $\hat{\varphi}_{i,j}(\lambda) \in [0,\frac{1}{2}]$ is the fraction among the B graphs that disagrees on the edge (i,j). For example if $\hat{\tau}_{i,j}(\lambda) = 0$ (all B graphs don't have an edge between i and j) then $\hat{\varphi}_{i,j}(\lambda) = 2\cdot 0(1-0) = 0$ meaning that 0% of the graphs disagree on the edge (i,j), on the contrary, if $\hat{\tau}_{i,j}(\lambda) = \frac{1}{2}$ (meaning that 50% for graphs have an edge between i and j while the other half doesn't) the instability is maximum $\hat{\varphi}_{i,j}(\lambda) = 2\cdot\frac{1}{2}(1-\frac{1}{2}) = \frac{1}{2}$ as we have a fifty-fifty split. Starting Λ from the value that ensures an empty graph λ_{max} the mean instability $\hat{\Phi}(\lambda) = \frac{1}{E}\sum_{i,j}\varphi_{i,j}(\lambda)$ will be equal to $\hat{\Phi}(\lambda_{max}) = 0$ because all the B graphs will agree on all edges (all empty). Then, as λ decreases, the estimated graphs will increase in density with an inevitable increase in instability until λ approaches zero (denoted as λ_0) for which all graphs will be fully connected and thus $\hat{\Phi}(\lambda_0) = 0$. Since the algorithm is interested in finding sparse graphs with low instability, the region of interest is the one near λ_{max}, thus $\hat{\Phi}(\lambda)$ is monotonized $\hat{\Phi}_{mon}(\lambda)$ as

$$\hat{\Phi}_{mon}(\lambda) = \begin{cases} \hat{\Phi}(\lambda) & \text{if } \lambda_{1/2} < \lambda < \lambda_{max} \\ \frac{1}{2} & \text{if } \lambda_0 < \lambda < \lambda_{1/2} \end{cases}$$

StARS chooses λ_{opt} as

$$\hat{\lambda}_{opt} = \inf\{\lambda : \hat{\Phi}_{mon}(\lambda) \leq \beta\}$$

So we select the smallest λ (corresponding to the denseest sparse graph) that ensures an average instability lower than a threshold β.

SPIEC-EASI (**Kurtz et al. 2015**) was the first method in the literature that used the concept of conditional dependence applied to metagenomics data. First, a log-ratio transformation is applied to the relative abundance matrix $\boldsymbol{X}$ to obtain the matrix $\boldsymbol{Z}$

$$\boldsymbol{Z} = \begin{bmatrix} 1-\frac{1}{p} & -\frac{1}{p} & \ldots & -\frac{1}{p} \\ -\frac{1}{p} & 1-\frac{1}{p} & \ldots & -\frac{1}{p} \\ \vdots & \vdots & \ddots & \vdots \\ -\frac{1}{p} & -\frac{1}{p} & \ldots & 1-\frac{1}{p} \end{bmatrix} \begin{bmatrix} X_{11} & X_{12} & \ldots & X_{1n} \\ X_{21} & X_{22} & \ldots & X_{2n} \\ \vdots & \vdots & \ddots & \vdots \\ X_{p1} & X_{p2} & \ldots & X_{pn} \end{bmatrix} = \boldsymbol{F}_{clr}\log(\boldsymbol{X})$$

The method is based on the relationship between the covariance matrix of the log-ratio transformed composition $\boldsymbol{\Sigma}_{\boldsymbol{z}}$ and the covariance matrix of the log-transformed basis $\boldsymbol{\Sigma}_{\log \boldsymbol{w}}$. Due

to the scale invariance of the clr transformation $\boldsymbol{z} = \boldsymbol{F}_{clr} \log(\boldsymbol{w})$, due to the properties of variance $Var(\boldsymbol{z}) = \boldsymbol{F}_{clr} Var(\log(\boldsymbol{w}))\boldsymbol{F}_{clr}^T$ and because the matrix $\boldsymbol{F}_{clr}$ is symmetric, we get

$$\boldsymbol{\Sigma}_{\boldsymbol{z}} = \boldsymbol{F}_{clr}\boldsymbol{\Sigma}_{\log \boldsymbol{w}}\boldsymbol{F}_{clr}$$

If the number of samples p is large, then the matrix $\boldsymbol{F}_{clr} \cong \boldsymbol{I}_p$ and thus a finite sample estimator of $\boldsymbol{\Sigma}_{\boldsymbol{z}}$ gives a good approximation of $\boldsymbol{\Sigma}_{\log \boldsymbol{w}}$ and of the true network. SPIEC-EASI implements both the MB method and Glasso (where in **Equation 3** we substitute to $\boldsymbol{S}$ the empirical covariance matrix $\hat{\boldsymbol{\Sigma}}_Z$) to estimate the precision matrix $\boldsymbol{\Omega}$, under the assumptions of Gaussianity ($\log(\boldsymbol{w}) \sim \mathcal{N}(\boldsymbol{\mu}, \boldsymbol{\Omega}^{-1})$) and that the network is sufficiently sparse. For both methods, the StARS model selection scheme is used to choose the best regularisation parameter λ. To determine the lambda path $\Lambda = [\lambda_{min}, \dots, \lambda_{max}]$ first λ_{max} is calculated as $\lambda_{max} = \max(|\boldsymbol{R}| - |diag(\boldsymbol{R})|)$ and $\lambda_{min} = \lambda_{max} \cdot r_\lambda$ with r_λ a user defined parameter. A list of the parameters used is available in **Table D**.

Table D – SPIEC-EASI parameters

Parameters	Code	Description	Default	Uniform
Subsamples (B)	`rep.num`	Number of subsamples used in StARS	20	50
Instability threshold (β)	`thresh`	Maximum instability allowed in StARS	0.05	0.1
Lambda ratio (r_λ)	`lambda.min.ratio`	Ratio between the maximum and minimum λ value tested	1e-3	1e-3
Length of Λ path (n_λ)	`nlambda`	Number of λ values $\in [\lambda_{min}, \lambda_{max}]$ evaluated to find λ_{opt}	20	30

SPRING (**Yoon et al. 2019**) as SPIEC-EASI uses the MB method to estimate the network, but uses a semi-parametric rank-based estimator for the correlation matrix, instead of the Pearson sample covariance matrix. First, the authors propose a modified centred log-ratio transformation

$$\text{mclr}_\varepsilon(\boldsymbol{x}) = \left[0, \dots, \frac{x_{q+1}}{\tilde{g}(\boldsymbol{x})} + \varepsilon, \dots, \frac{x_p}{\tilde{g}(\boldsymbol{x})} + \varepsilon\right]$$

where $\tilde{g}(\boldsymbol{x})$ is the geometric mean across the $p - q$ components different from zero and ε is the smallest constant for which all components of $\text{mclr}_\varepsilon(\boldsymbol{x}) \geq 0$.

To estimate the covariance matrix SPRING assumes that the *mclr* transformed data follow a truncated Gaussian copula model. Given a random vector $\boldsymbol{z} = \text{mclr}_\varepsilon(\boldsymbol{x})$ it satisfies the truncated Gaussian copula model if it exits a family of monotonically increasing functions $\boldsymbol{f} = \{f_1, \dots f_p\}$ such that for $\boldsymbol{f}(\boldsymbol{z}) > c$ it satisfies $[f(z_1), \dots, f(z_p)] \sim \mathcal{N}(\boldsymbol{0}, \boldsymbol{\Sigma})$ with $\sigma_{ii} = 1$ and for $\boldsymbol{f}(\boldsymbol{z}) \leq c$ we have that $\boldsymbol{f}(\boldsymbol{z}) = 0$.

For such truncated Gaussian copula models, there exists an explicit form of the bridge function F that links the sample Kendall's τ estimates to the elements of $\boldsymbol{\Sigma}$ defined as $E[\hat{\tau}_{ij}] = F(\sigma_{ij})$ where $\hat{\tau}_{ij}$ is sample Kendall's τ estimate which can be calculated from n samples of random vector $\boldsymbol{z}$ stored in the matrix $\boldsymbol{Z} = \boldsymbol{F}_{mclr} \log(\boldsymbol{X})$. From this we can estimate the semi-parametric rank-based covariance matrix $\tilde{\boldsymbol{\Sigma}}$ from $F^{-1}(\hat{\tau}_{ij})$ $\forall i, j = 1, \dots, p$. The matrix $\tilde{\boldsymbol{\Sigma}}$ is then used in the MB method 4 instead of the Pearson sample covariance matrix.

$$\hat{\boldsymbol{\theta}}^i = \underset{\boldsymbol{\theta} \in \mathbb{R}^{p-1}}{\operatorname{argmin}} \, \boldsymbol{\theta}^T \hat{\boldsymbol{\Sigma}} \boldsymbol{\theta} - 2\boldsymbol{\theta}^T \hat{\boldsymbol{\Sigma}}_{\cdot \boldsymbol{i}} + \lambda \|\boldsymbol{\theta}\|_1$$

SPRING uses the StARS model selection method to choose the best regularisation parameter λ. A list of the parameters used is available in **Table E**.

Table E – SPRING parameters

Parameters	Code	Description	Default	Uniform
Subsamples (B)	`rep.num`	Number of subsamples used in StARS	20	50
Instability threshold (β)	`thresh`	Maximum instability allowed in StARS	0.1	0.1
Lambda ratio (r_λ)	`lambda.min.ratio`	Ratio between the maximum and minimum λ value tested	1e-2	1e-3
Length of Λ path (n_λ)	`nlambda`	Number of λ values $\in [\lambda_{min}, \lambda_{max}]$ evaluated to find λ_{opt}	20	30
Lambda path (Λ)	`lambdaseq`	Vector of λ values evaluated to find λ_{opt}	■[1]	■[2]

[1] `lambdaseq = exp(seq(log(0.6), log(0.6*lambda.min.ratio), length.out = nlambda))`

[2] In the uniform parameters configuration $\lambda_{max} = \max(|\boldsymbol{R}| - |diag(\boldsymbol{R})|)$ and then n_λ values are generated to be equally spaced on a logarithmic scale in the interval $[\lambda_{max} \cdot r_\lambda, \lambda_{max}]$

gCoda (Fang et al. 2017) is also based on the minimisation of a negative log-likelihood function with an ℓ_1 regularisation term, but the parametric assumptions made by the authors are different. The authors assume that the real abundances $\boldsymbol{w}$ follow a log-normal distribution $\log(\boldsymbol{w}) \sim \mathcal{N}(\boldsymbol{\mu}, \boldsymbol{\Omega}^{-1})$. Under this assumption, the composition of $\boldsymbol{w}$ obtained from the closure operation $\boldsymbol{x} = \mathcal{C}(\boldsymbol{w})$ follows a Logistic-Normal distribution $\boldsymbol{x} \sim \mathscr{L}(\boldsymbol{F}\boldsymbol{\mu}, \boldsymbol{F}\boldsymbol{\Omega}^{-1}\boldsymbol{F}^T)$ (Aitchison and Shen 1980) with $\boldsymbol{F}$ the clr transformation matrix. Under these assumptions, the optimisation problem is

$$\hat{\boldsymbol{\Omega}} = \underset{\boldsymbol{\Omega} \succ 0}{\operatorname{argmin}} \; -\log\left(\frac{\det \boldsymbol{\Omega}}{\mathbf{1}_p^T \boldsymbol{\Omega} \mathbf{1}_p}\right) + \operatorname{tr}\left(\hat{\boldsymbol{\Sigma}}_{\log \boldsymbol{X}}\left(\boldsymbol{\Omega} - \frac{\boldsymbol{\Omega}\mathbf{1}_p\mathbf{1}_p^T\boldsymbol{\Omega}}{\mathbf{1}_p^T\boldsymbol{\Omega}\mathbf{1}_p}\right)\right) + \lambda\|\boldsymbol{\Omega}\|_1$$

with $\mathbf{1}_p$ a $p \times 1$ vector of ones. To solve the minimization problem gCoda implements a Majorization-Minimization algorithm and selects the regularization parameter λ by minimizing the Extended Bayesian Information Criteria

$$\text{EBIC}(\lambda) = n \; \ell_{neg}(\hat{\boldsymbol{\Omega}}) + \#Edges \cdot (\log n + 4\gamma \log p)$$

In the finite sample case (Foygel and Drton 2010) shows that for low values of γ, we are more likely to obtain a good (high) positive selection rate. For higher values of γ, we are more likely to obtain a good (low) false discovery rate. A list of the parameters used is available in Table F.

Table F – gCoda parameters

Parameters	Code	Description	Default	Uniform
Length of Λ path (n_λ)	`nlambda`	Number of λ values $\in [\lambda_{min}, \lambda_{max}]$ evaluated to find λ_{opt}	15	30
Lambda ratio (r_λ)	`lambda.min.ratio`	Ratio between the maximum and minimum λ value tested	1e-4	1e-3
EBIC γ parameter	`ebic.gamma`	Extended Bayesian Inforamtion Criterion parameter	0.5	0.5

FlashWeave (Tackmann et al. 2019) is based on the local-to-global learning (LGL) approach (Aliferis et al. 2010) where the directly associated neighbours are inferred for each

variable x_i then the individual neighbourhoods are connected to form a global network using an `OR` combination rule (edge (i, j) is included in the final network if $x_i \in N(x_j)$ or $x_j \in N(x_i)$). FlashWeave starts by computing for each variable x_i its unconditioned association to all other variables. All significant unconditioned associations become candidates for inclusion in $N(x_i)$, the variable with the strongest unconditioned association is automatically added to the tentative neighbourhood of x_i, and then all other candidates are tested for inclusion in the tentative neighbourhood by probing the association between x_i conditioned on all sub-sets (conditioning sets) of the members already included in the tentative neighbourhood. The final neighbourhood is determined by performing the test for conditional independence that where skipped in the previous step (for example, the first variable in the tentative neighbourhood is added with no conditional dependence test). The results of the statistical test are corrected for multiple tests using the Benjamini-Hochberg procedure, and a variable is added to the neighbourhood if the test p-value$< \alpha$. FlashWeave introduces a number of heuristics to accelerate the inference procedure:

- *feedforward*: the computation of the neighbourhood of the hub nodes x_h is computationally costly (since we need to test the conditional dependence with all subsets in the large tentative neighbourhood). Now, for the edge $(h \to i)$ to be included in the final global network using the `OR` combinator rule, it is sufficient that the considerably cheaper reverse link $(i \to h)$ is proven. The heuristic exploits this property by prioritising the computation of variables expected to have smaller neighbourhoods (as approximated by their unconditioned neighbourhood size).

- *fast-elimination*: even if a variable x_j is discarded during the final determination of the neighbourhood, it will still be included in future conditioning sets, thus inflating the number of conducted conditional independence tests. The heuristic addresses this computational hurdle by not considering a removed variable x_j for any subsequent conditioning sets.

A list of the parameters used is available in **Table G**.

Table G – FlashWeave parameters

Parameters	Code	Description	Default	Uniform
Max-k	`max_k`	Maximum number of variables in the conditioning set, see (**Aliferis et al. 2010**)	3	-
Heuristics	`feed_forward`	Use *feedfoward* heuristic	True	-
Heuristics	`fast_elim`	Use *fast-elimination* heuristic	True	-
P-value threshold (α)	`alpha`		0.01	-

HARMONIES (**Jiang et al. 2020**) is a recently developed zero-inflated aware method based on conditional dependence. HARMONIES first fits a zero-inflated negative binomial (ZINB) model with the Dirichlet Process Prior (DPP) to normalise the data and then estimates the precision matrix $\boldsymbol{\Omega}$ using the glasso algorithm and selects the regularisation parameter λ using StARS. In this work, we used a modified version of the original package to uniform the input parameters with the other methods, without modification to the package functionality. The modified version is available at https://github.com/mariottopier0/HARMONIES. A list of the parameters used is available in **Table H**.

Table H – HARMONIES parameters

Parameters	Code	Description	Default	Uniform
Subsamples (B)	`n.rep`	Number of subsamples used in StARS	20	50
Instability threshold (β)	`beta.stars`	Maximum instability allowed in StARS	0.05	0.1
Lambda ratio (r_λ)	`lambda.min.ratio`	Ratio between the maximum and minimum λ value tested	1e-2	1e-3
Length of Λ path (n_λ)	`nlambda`	Number of λ values $\in [\lambda_{min}, \lambda_{max}]$ evaluated to find λ_{opt}	■[1]	30
Lambda step (λ_{step})	`lambda.step`	Vector of λ values evaluated to find λ_{opt}	-0.01	■[2]
Sparsity threshold	`sparsity.cutoff`	Numerical threshold for filtering species with sparsity larger `sparsity.cutoff`	**1***	

[1]In the default implementation n_λ is not explicit. With `lambda.max=max(cor.mat)` Λ is determined using λ_{step} as: `seq(lambda.max, lambda.max / 100, by = lambda.step)`

[2]With the uniform parameters configuration λ_{step} is determined based on n_λ as for the other methods `lambda.step = - (lambda.max - lambda.min.ratio * lambda.max) / nlambda`

*Filtering parameters set to custom defaults to ensure no filtering of the input data, actual defaults are `sparsity.cutoff=0.5`

COZINE (Ha et al. 2020) relies on the multivariate Hurdle model to infer a sparse set of conditional dependencies which reflect not only relationships among continuous values but also between binary indicators of presence or absence and between binary and continuous representations of the data. Given the compositional vector $\boldsymbol{x}$ we can obtain $\boldsymbol{z} = clr_0(\boldsymbol{x})$ (with clr_0 indicating the centred log-ratio transformation applied to non-zero values) and the vector $\boldsymbol{i}$ obtained by applying to each element the indicator function $I(\boldsymbol{x} \neq 0) = 1$.
COZINE assumes that

- The random vector $\boldsymbol{i}$ follows an Ising model with joint probability

$$p(\boldsymbol{i}) \propto e^{\boldsymbol{i}^T \boldsymbol{G} \boldsymbol{i}}$$

- given a realisation of $\boldsymbol{i}$ the random vector $\boldsymbol{z}$ follows a normal distribution

$$(\boldsymbol{z}|\boldsymbol{i} = \boldsymbol{i}_k) \sim \mathcal{N}(\boldsymbol{\mu}_{\boldsymbol{i}}, \boldsymbol{\Sigma}_{\boldsymbol{i}}^{-1})$$

- the mean vector

$$\boldsymbol{\mu}_{\boldsymbol{i}} = (diag(\boldsymbol{i}_k)\boldsymbol{\Sigma}_{\boldsymbol{i}} diag(\boldsymbol{i}_k))^{-} \boldsymbol{H}^T \boldsymbol{i}$$

  were $diag(\boldsymbol{i}_k)$ indicates a $p \times p$ diagonal matrix with $\boldsymbol{i}_k$ on the diagonal.

By combining these assumptions, the joint distribution of $\boldsymbol{z}$ and $\boldsymbol{i}$ follows the multivariate Hurdle model. The joint density is in an exponential family with three interaction matrices $\boldsymbol{G}, \boldsymbol{\Sigma}^{-1}$ and $\boldsymbol{H}$ as natural parameters (Ha et al. 2020). In order to estimate its parameters, COZINE solves a node-wise penalised log-likelihood estimation problem.
The elements of the tree $p \times p$ matrices $\boldsymbol{G}$, $\boldsymbol{\Sigma}^{-1}$ and $\boldsymbol{H}$ encode different types of interaction between two different nodes:

- Binary-Binary: if $g_{ij} = 0$ then the random variables i_i and i_j are conditionally independent, i.e. the presence/absence of one taxon doesn't affect the other.
- Continuous-Continuous: $\sigma_{ij}^{(-)}$ encodes conditional dependencies in the abundance of the two taxa when observed.
- Binary-Continuous: h_{ij} quantifies the mean level of abundance of taxa i when taxa j is present.

In the inferred graph an edge E_{ij} is missing between nodes (i.e. taxa) i and j if all 3 values are equal to zero $g_{ij} = \sigma_{ij}^{(-)} = h_{ij} = 0$. Thus if at least one of the 3 interaction terms is different from zero and edge is drawn between the two nodes. In this work, we used a modified version of the original package to uniform the input parameters with the other methods, with no modification to the package functionality. The modified version is available at https://github.com/mariottopier0/COZINE. A list of the used parameters is available in **Table I**.

Table I – COZINE parameters

Parameters	Code	Description	Default	Uniform
Length of Λ path (n_λ)	`nlambda`	Number of λ values $\in [\lambda_{min}, \lambda_{max}]$ evaluated to find λ_{opt}	50	30
Lambda ratio (r_λ)	`lambda.min.ratio`	Ratio between the maximum and minimum λ value tested	0.5	1e-3

3 Sensitivity analysis

Conditional dependence methods share several input parameters, many of which have the same interpretation across methods but differ in their default values. Although typical usage involves using default settings, as in our benchmarking, and a systematic assessment of parameter tuning lies outside the scope of this study, we conducted preliminary analyses (BA graph model, medium β-diversity and default simulation parameters $\rho(\mathcal{G}) = 5\%, n = 100, p = 50, r_{neg} = 0.05, K = 100\%$), comparing method performance under default parameter settings against the performance obtained using a common value for corresponding parameters across all methods (hereafter referred to as the "uniform" configuration). The purpose of these analyses was to assess whether differences in performance are primarily driven by methodological choices rather than by discrepancies in default parameter settings.
In particular, SPIEC-EASI, SPRING, gCoda, HARMONIES, and COZINE use an ℓ_1-penalized maximum likelihood estimator and thus need to select the optimal λ value in an arbitrary Λ path. In the uniform parameter configuration, we set for all methods the length of the Λ path at 30 (i.e., the number of λ values to test) and the extremes of the Λ path as $(\lambda_{min} = \lambda_{max} \cdot 1e-3, \lambda_{max} = \max(|\mathbf{R}|))$. SPIEC-EASI, SPRING and HARMONIES use StARS to select the optimal λ value. For these three methods, we set, in the uniform configuration, the parameter $\beta = 0.1$ and $B = 50$.
These preliminary analyses using MCC (**Figure B**) indicate that method performance is largely insensitive to these parameter modifications, and most methods achieve comparable results in both default and uniform configurations.

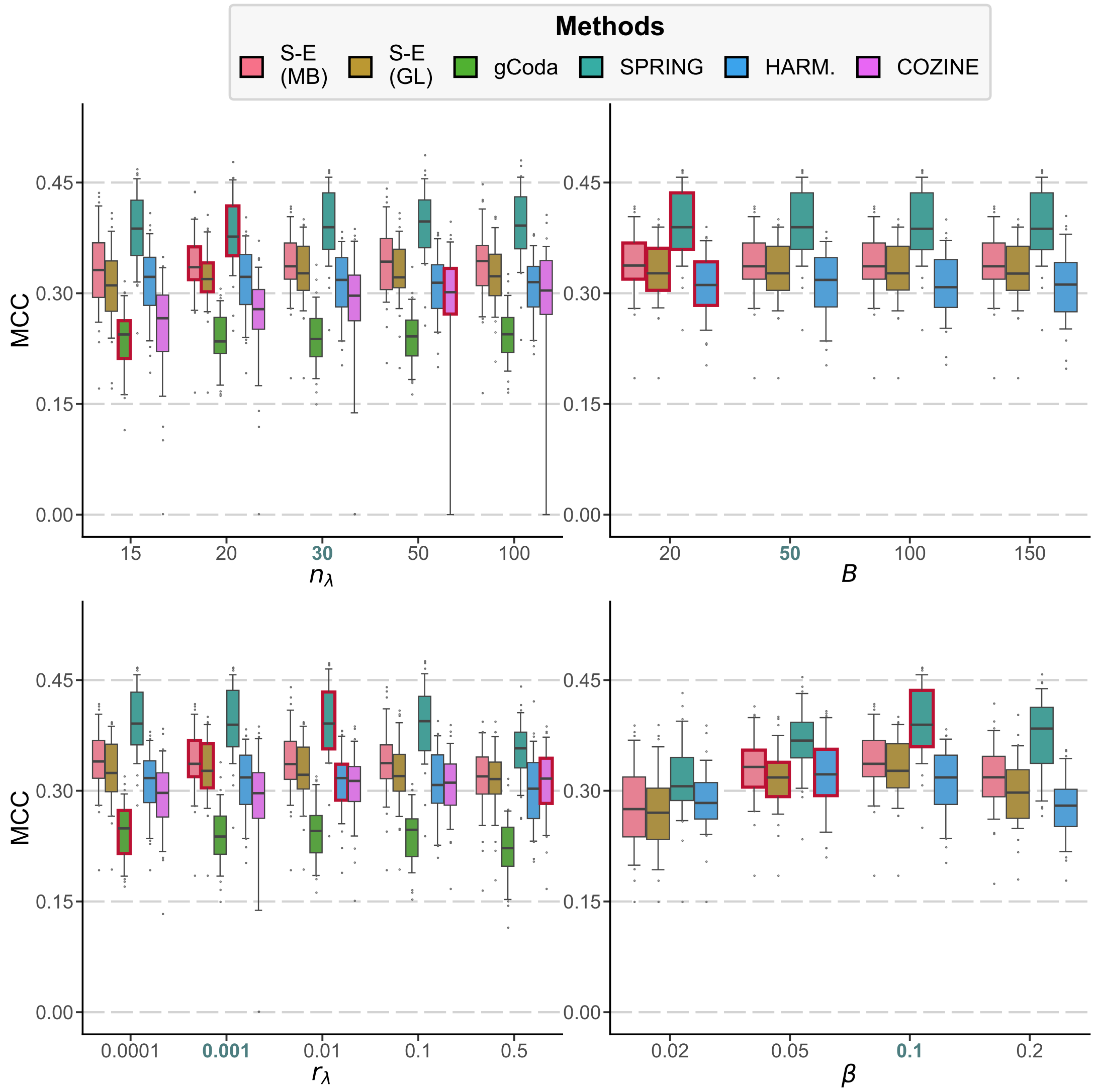

Fig B – Effect of input parameters on conditional dependence methods. MCC values for conditional dependence methods applied to 50 datasets generated with BA model, medium β-diversity and default simulation parameters ($\rho(\mathcal{G}) = 5\%, n = 100, p = 50, r_{neg} = 0.05, K = 100\%$). The 4 panels illustrate the performance of the methods as one of the methods' parameters is varied, in clock-wise order: the number of values in the Λ path n_λ, the number of subsamples B, the instability threshold β and the ratio between the Λ path extremes r_λ. Default parameters of the various methods are identified by a red outline while the values chosen as uniform input for all methods are reported in blue on the x-axis tick labels.

References

J. Aitchison. The statistical analysis of compositional data. *Journal of the Royal Statistical Society. Series B (Methodological)*, 44(2):139–177, 1982. URL http://www.jstor.org/stable/2345821.

J. Aitchison and S. M. Shen. Logistic-Normal Distributions: Some Properties and Uses. *Biometrika*, 67(2):261–272, 1980. doi: 10.2307/2335470. URL http://www.jstor.org/stable/2335470.

C. F. Aliferis, A. Statnikov, I. Tsamardinos, S. Mani, and X. D. Koutsoukos. Local Causal and Markov Blanket Induction for Causal Discovery and Feature Selection for Classification Part I: Algorithms and Empirical Evaluation. *Journal of Machine Learning Research*, 11(7): 171–234, 2010. URL http://jmlr.org/papers/v11/aliferis10a.html.

H. Fang, C. Huang, H. Zhao, and M. Deng. CCLasso: correlation inference for compositional data through Lasso. *Bioinformatics*, 31(19):3172–3180, 2015. doi: 10.1093/bioinformatics/btv349. URL https://doi.org/10.1093/bioinformatics/btv349.

H. Fang, C. Huang, H. Zhao, and M. Deng. gCoda: Conditional Dependence Network Inference for Compositional Data. *Journal of Computational Biology*, 24(7):699–708, 2017. doi: 10.1089/cmb.2017.0054. URL https://doi.org/10.1089/cmb.2017.0054.

R. Foygel and M. Drton. Extended Bayesian Information Criteria for Gaussian Graphical Models. In J. Lafferty, C. Williams, J. Shawe-Taylor, R. Zemel, and A. Culotta, editors, *Advances in Neural Information Processing Systems*, Vancouver, B.C., Canada, 2010. Curran Associates, Inc. URL https://proceedings.neurips.cc/paper_files/paper/2010/file/072b030ba126b2f4b2374f342be9ed44-Paper.pdf.

J. Friedman and E. J. Alm. Inferring Correlation Networks from Genomic Survey Data. *PLOS Computational Biology*, 8(9):1–11, 2012. doi: 10.1371/journal.pcbi.1002687. URL https://doi.org/10.1371/journal.pcbi.1002687.

J. Friedman, T. Hastie, and R. Tibshirani. Sparse inverse covariance estimation with the graphical lasso. *Biostatistics*, 9(3):432–441, 2007. doi: 10.1093/biostatistics/kxm045. URL https://doi.org/10.1093/biostatistics/kxm045.

M. J. Ha, J. Kim, J. Galloway-Peña, K.-A. Do, and C. B. Peterson. Compositional zero-inflated network estimation for microbiome data. *BMC Bioinformatics*, 21:581, 2020. doi: 10.1186/s12859-020-03911-w. URL https://doi.org/10.1186/s12859-020-03911-w.

S. Jiang, G. Xiao, A. Y. Koh, Y. Chen, B. Yao, Q. Li, and X. Zhan. HARMONIES: A Hybrid Approach for Microbiome Networks Inference via Exploiting Sparsity. *Frontiers in Genetics*, 11, 2020. doi: 10.3389/fgene.2020.00445. URL https://www.frontiersin.org/articles/10.3389/fgene.2020.00445.

Z. D. Kurtz, C. L. Müller, E. R. Miraldi, D. R. Littman, M. J. Blaser, and R. A. Bonneau. Sparse and Compositionally Robust Inference of Microbial Ecological Networks. *PLOS Computational Biology*, 11(5):1–25, 2015. doi: 10.1371/journal.pcbi.1004226. URL https://doi.org/10.1371/journal.pcbi.1004226.

H. Lin, M. Eggesbø, and S. D. Peddada. Linear and nonlinear correlation estimators unveil undescribed taxa interactions in microbiome data. *Nature Communications*, 13:4946, 2022. doi: 10.1038/s41467-022-32243-x. URL https://doi.org/10.1038/s41467-022-32243-x.

H. Liu, K. Roeder, and L. Wasserman. Stability Approach to Regularization Selection (StARS) for High Dimensional Graphical Models. In *Proceedings of the 23rd International Conference on Neural Information Processing Systems - Volume 2*, page 1432–1440, Red Hook, NY, USA, 2010. Curran Associates Inc.

D. Lovell, V. Pawlowsky-Glahn, J. J. Egozcue, S. Marguerat, and J. Bähler. Proportionality: A Valid Alternative to Correlation for Relative Data. *PLOS Computational Biology*, 11(3):1–12, 2015. doi: 10.1371/journal.pcbi.1004075. URL https://doi.org/10.1371/journal.pcbi.1004075.

N. Meinshausen and P. Bühlmann. High-dimensional graphs and variable selection with the Lasso. *The Annals of Statistics*, 34(3):1436 – 1462, 2006. doi: 10.1214/009053606000000281. URL https://doi.org/10.1214/009053606000000281.

R. Sipos, A. J. Székely, M. Palatinszky, S. Révész, K. Márialigeti, and M. Nikolausz. Effect of primer mismatch, annealing temperature and PCR cycle number on 16S rRNA gene-targetting bacterial community analysis. *FEMS Microbiology Ecology*, 60(2):341–350, 2007. doi: 10.1111/j.1574-6941.2007.00283.x. URL https://doi.org/10.1111/j.1574-6941.2007.00283.x.

M. T. Suzuki and S. J. Giovannoni. Bias caused by template annealing in the amplification of mixtures of 16S rRNA genes by PCR. *Applied and Environmental Microbiology*, 62 (2):625–630, 1996. doi: 10.1128/aem.62.2.625-630.1996. URL https://journals.asm.org/doi/abs/10.1128/aem.62.2.625-630.1996.

G. J. Székely, M. L. Rizzo, and N. K. Bakirov. Measuring and testing dependence by correlation of distances. *The Annals of Statistics*, 35(6):2769 – 2794, 2007. doi: 10.1214/009053607000000505. URL https://doi.org/10.1214/009053607000000505.

J. Tackmann, J. F. Matias Rodrigues, and C. von Mering. Rapid Inference of Direct Interactions in Large-Scale Ecological Networks from Heterogeneous Microbial Sequencing Data. *Cell Systems*, 9(3):286–296.e8, 2019. doi: https://doi.org/10.1016/j.cels.2019.08.002. URL https://www.sciencedirect.com/science/article/pii/S2405471219302716.

G. Yoon, I. Gaynanova, and C. L. Müller. Microbial Networks in SPRING - Semi-parametric Rank-Based Correlation and Partial Correlation Estimation for Quantitative Microbiome Data. *Frontiers in Genetics*, 10, 2019. doi: 10.3389/fgene.2019.00516. URL https://www.frontiersin.org/articles/10.3389/fgene.2019.00516.
