# Supplementary material for: From 16S rDNA-seq count data to microbial interactions: A comprehensive benchmark of co-occurrence network inference methods
Source: PLoS Comput Biol. 2026 Oct 29;22(10):e1014898. doi: 10.1371/journal.pcbi.1014898 (PMC13750663; doi:10.1371/journal.pcbi.1014898)
Supplement: Supplementary file 3 [file pcbi.1014898.s003.pdf]

Additional results and figures

Piero Mariotto[1], Matteo Baldan[1], Barbara Di Camillo[1,2,3], and Giacomo Baruzzo[1,*]

[1]Department of Information Engineering, University of Padova, Padova (PD), Italy.
[2]Department of Comparative Biomedicine and Food Science, University of Padova, Legnaro (PD), Italy.
[3]Padua Center of Network Medicine, University of Padova, Padova (PD), Italy.
[*]Corresponding author (giacomo.baruzzo@unipd.it)

In this document, we provide all the results produced during the study, which could not all be included in the main paper. The document is structured in the same manner as the result section of the main paper, the sections are presented in the same order and with the same title to give a quick and easy reference to the reader coming from the main paper.
Throughout the document and figures, we frequently use abbreviations for the methods names. Table A presents a map between the full name of the methods, their abbreviations, and the assigned colours used in the figures.

Table A – Map of the methods abbreviations and colours. *For each method we have its full name, the abbreviation used in the figures' legends and the colour used in the figures.*

Method	Abbreviation	Colour
Pearson correlation	PEA	
Spearman correlation	SPEA	
Partial Pearson correlation	P-PEA	
Partial Spearman correlation	P-SPEA	
Bray Curtis dissimilarity	BC	
Proportionality	PROP	
SparCC	SparCC	
CCLasso	CCLasso	
SECOM (linear)	SECOM (L)	
SECOM (non-linear)	SECOM (NL)	
SPIEC-EASI (Meinshausen-Bühlmann)	S-E (MB)	
SPIEC-EASI (graphical lasso)	S-E (GL)	
gCoda	gCoda	
SPRING	SPRING	
HARMONIES	HARM.	
COZINE	COZINE	
FlashWeave (partial correlation)	FW (PC)	
FlashWeave (mutual information)	FW (MI)	

1 Comparison with previous benchmark studies

We restricted our review to studies dedicated entirely, or predominantly, to comparing network inference methods for metagenomic data and offering a standalone, independent benchmarking analysis. We left out works where benchmarking was secondary or narrow in scope, such as papers presenting a new network inference approach or a metagenomic analysis pipeline. A high level overview of the previous benchmarks and the current work is available in Table B. For each study, we gathered information about the input data used to assess the inference methods, the number of methods examined, the evaluation metrics employed (organized as in the main paper into three categories: edge-, node-, and graph-wise), the parameters for which the impact on inference methods was explicitly evaluated, and any other notable aspects reported in the studies.

1.1 Methods' ranking

No previous benchmark reported an explicit and overall ranking of inference methods. Thus, to compare the robustness of our ranking to the one possibly obtained with other simulation frameworks, we derived from the studies, where possible, a partial ranking of the overlapping methods between this and their study.
To derive the ranking, we considered only the edge-wise metric values reported in the manuscripts, as these were the only metrics common to all studies (with the exception of Agyapong et al. 2025, which was therefore excluded from the ranking comparison). Here follows a detailed description of how the ranking was derived for each study or why such ranking was not possible to obtain:

- Berry and Widder 2014: The authors do not present an overall ranking of the tools in their study. We therefore ranked the tools by jointly considering the Recall and Specificity values shown in Figure 2B of the original manuscript. This figure was the only one that reported an evaluation metric stratified by inference method, rather than aggregating methods when assessing the impact of simulation parameters.

- Weiss et al. 2016: The authors do not present an overall ranking of the tools in their study. Therefore, we ordered the tools based on the F1 scores reported in Table 2 of the Supplementary Material and the Precision and Recall values shown in Figure 6 of the original manuscript. For Table 2, we produced a ranking for each Excel sheet after calculating the mean F1 across all simulations. For Figure 6, we visually determined a Precision ranking and a Recall ranking for each of the five datasets. The overall ranking was then calculated by taking the median rank for each method. Figure 2 in the original manuscript presents robustness metrics rather than method correctness, and therefore it was not taken into account. Figure 3 and 5 were not included in the ranking as they report fraction of inferred edges but not if the edges inferred are correct or not. Figure 4 was not used as it reports overlapping and irregular Precision-Recall curves but no AUC values and thus deriving a ranking was impossible.

- Hirano and Takemoto 2019: The authors do not present an overall ranking of the tools in their study. We therefore ranked the tools by considering the corrected AUPR values reported in Figure 1A-B and Figure 2A-B of the main manuscript and Figure S4A-B, Figure S5A-B, Figure S6, Figure S7, Figure S8, Figure S9A-B, Figure S10, Figure S12A, Figure S14A-B, Figure S15, Figure S17A-B and Figure S18 of the Supplementary Information. For each plot, we visually determined a ranking (for line plots, we used the y values corresponding to the midpoint along the x-axis) and the final ranking was obtained as the median rank for each method. Figure 3 and Figure 5 were not considered

because they do not report values for all methods and Figure 4 was not used because there was no way to obtain a consistent ranking.

- Brunner et al. 2024: The authors do not provide an overall ranking of the tools evaluated in their study. We therefore ranked the tools by combining the evidence from the R^2 and $AUROC$ values shown in Figure 4, Figure 5, and Figure 6. For each plot, we visually assigned ranks, and the final ordering for each method was determined by its median rank across plots. Figure 8 and Figure 9 report node- and graph-level metrics and were not included, since the ranking is based on edge-level metrics.

- Agyapong et al. 2025: In their original work, the authors do not evaluate the methods using any edge-wise metric. Instead, they report only Mean Squared Error (MSE) computed on held-out abundance data. Therefore, it was not feasible to produce an edge-wise metric based ranking for this study.

- Ghaeli et al. 2026: The authors do not provide an overall ranking of the tools assessed in their study. Consequently, we ranked the tools using the F1 scores shown in Figure 4: for each synthetic dataset, we visually inferred a ranking among the methods (the first 12 violin plots; the last 7 plots address reproducibility and were excluded). Each method's final position was then defined by its median rank across the considered plots. We did not use Figure 3 because its split-panel format did not allow a reliable, standardized visual comparison across all tools. The other figures and tables in the main manuscript focused on metrics related to the reproducibility of method inference and were therefore omitted. In the Supplementary Material, Figures S25, S26, and S27 shared the same layout as Figure 3, and the remaining figures reported either inference reproducibility or similarity between simulated and real data. For these reasons, the Supplementary Material was not used to derive the partial method ranking in this study.

A summary of the ranking comparisons is reported in Figure A. For the studies whose overlap included at least four methods (Weiss et al. 2016; Hirano and Takemoto 2019; Ghaeli et al. 2026), we calculated Spearman's and Kendall's correlations between the rankings of the methods in common. Overall, the comparison indicates that the ranking obtained in this work aligns well with the two most comprehensive studies: it perfectly matches Ghaeli et al. 2026 ($\rho = 1$ and $\tau = 1$) and shows a substential correlation with Hirano and Takemoto 2019 ($\rho = 0.6$ and $\tau = 0.45$). This is particularly noteworthy because these two studies rely on data simulation frameworks different from ours, gLV for Hirano and Takemoto 2019 and NORTA for Ghaeli et al. 2026. This may suggest that the relative ranking of inference methods is stable across data-generation frameworks; however, additional work is required to substantiate this hypothesis. Weiss et al. 2016 diverges more from the ranking reported in this study, particularly regarding the characterisation of SparCC (placed at the top of the derived ranking but at the bottom of the rankings of this work, Hirano and Takemoto 2019 and Ghaeli et al. 2026) and Bray-Curtis dissimilarity (ranked last in the derived ranking while appearing near the top in this work's ranking). The other two studies, Berry and Widder 2014 and Brunner et al. 2024, have limited method overlap (less than 3) and are focused on particular aspects of network inference (base methods in Berry and Widder 2014 and the impact of compositionality in Brunner et al. 2024), making a direct comparison of their rankings more difficult.
As a final remark, we emphasise that the partial ranking presented herein should neither be expanded nor interpreted as a validation of the complete ranking of 18 methods reported in this study. Moreover, the rankings reported were derived by the authors of the present manuscript and therefore should not be interpreted as representing the view of the original authors.

Table B – Studies overview. *For each study, we report the input data, the number of methods evaluated, the metrics applied, and the parameters whose impact on inference methods was explicitly assessed.*

		**This work**	Berry and Widder 2014	Weiss et al. 2016	Hirano and Takemoto 2019	Brunner et al. 2024	Agyapong et al. 2025	Ghaeli et al. 2026
**General**	Data	MiCRM	gLV	Parametric distributions and gLV	gLV	Parametric distributions	Real dataset[1]	NORTA[2] and real dataset[1]
	Number of methods	18	6[†]	8	9	6	4	6
**Evaluation metrics**	Edge-wise metrics	MCC, Precision, Recall	Recall, Specificity	Recall, Specificity, Precision	Precision, Recall, cAUPR[3]	R^2, AUROC	✗	Precision, Recall, F1 score
	Node-wise metrics	$\mathcal{C}_B$, $\mathcal{C}_C$, $\mathcal{C}_E$	$p(k)$	✗	✗	$\mathcal{C}_B$, $\mathcal{C}_D$, $\mathcal{C}_E$	✗	✗
	Graph-wise metrics	δ_{KS}, $\rho(\mathcal{G})$, $\mathcal{D}$, $\mathcal{R}$, $\overline{d}$, $\overline{C}$	$\overline{k}$, $\overline{d}$, $\overline{C}$, $\overline{\mathcal{C}_B}$, $\overline{\mathcal{C}_C}$, keystoness[4]	✗	✗	community detection	MSE[5]	✗
	Computational burden	✓	✗	✗	✗	✗	✗	✗
**Parameters explicitly tested**	Topologies	ER, BA, WS	ER, BA, WS, KE[6]	✗	ER, BA, WS	✗	✗	ER, BA, banded, clustered
	Number of species	50, 100, 150, 200	From 10 to 400*	✓	From 10 to 100*	✗	✗	✗
	Number of samples	50, 75, 100, 200, 300, 500, 1000	From ∼2 to 300*	✗	From ∼10 to 500*	75, 150, 300	From 10 to 85*	✗
	Network density	2%, 5%, 8%, 10%, 15%	From ∼0% to 60%*	✗	From $\overline{k}$=2 to $\overline{k}$=20[§]*	dense/ sparse*	✗	✗
	Interaction types	$r_{neg}=0$, 0.05, 0.1, 0.2	✗	✓	random, mutualism, competition, mixed, predator-pray	positively/ negatively biased*	✗	✗
	Library size	100%, 10%, 2%, 1%, 0.5%	✗	✗	✗	✗	✗	✗
	Other	Uniform configuration of common methods' parameters, β-diversity	Intra-sample evenness (α-diversity), Inter-sample heterogeneity (β-diversity)	Different normalisation approaches	✗	Inter-kingdom interactions	Cross-validation evaluation	Reproducibility assessment

[1]No ground-truth interaction network available.
[2]NORmal To Anything algorithm Nelsen 2006, used to generate synthetic data from an a priori covariance matrix and a real count matrix.
[3] Corrected area under the PR curve, calculated as ($AUPR_{obs}$–$AUPR_{rand}$) / (1–$AUPR_{rand}$), where $AUPR_{obs}$ and $AUPR_{rand}$ correspond to the observed AUPR value and the AUPR value obtained from random prediction.
[4]The number of lost species in the steady state community after the removal of a specie.
[5]Mean Square Error (MSE) between the counts and the predicted values of species' counts for the samples in the test set.
[6] Klemm-Eguiluz (KE) model: generates topologies with small world and scale-free properties as well as modularity.
[†]Only base metrics: Bray-Curtis dissimilarity, Jaccard index, Mutual Information, Kendall correlation, Pearson correlation, Spearman correlation.
[*] Values not explicitly stated in the main paper. Ranges were estimated visually from the paper figures.
[§]In the paper the authors express the level of connectivity using the average degree $\overline{k}$ which can be converted to density as $\rho(\mathcal{G}) = \overline{k}/(p-1)$.

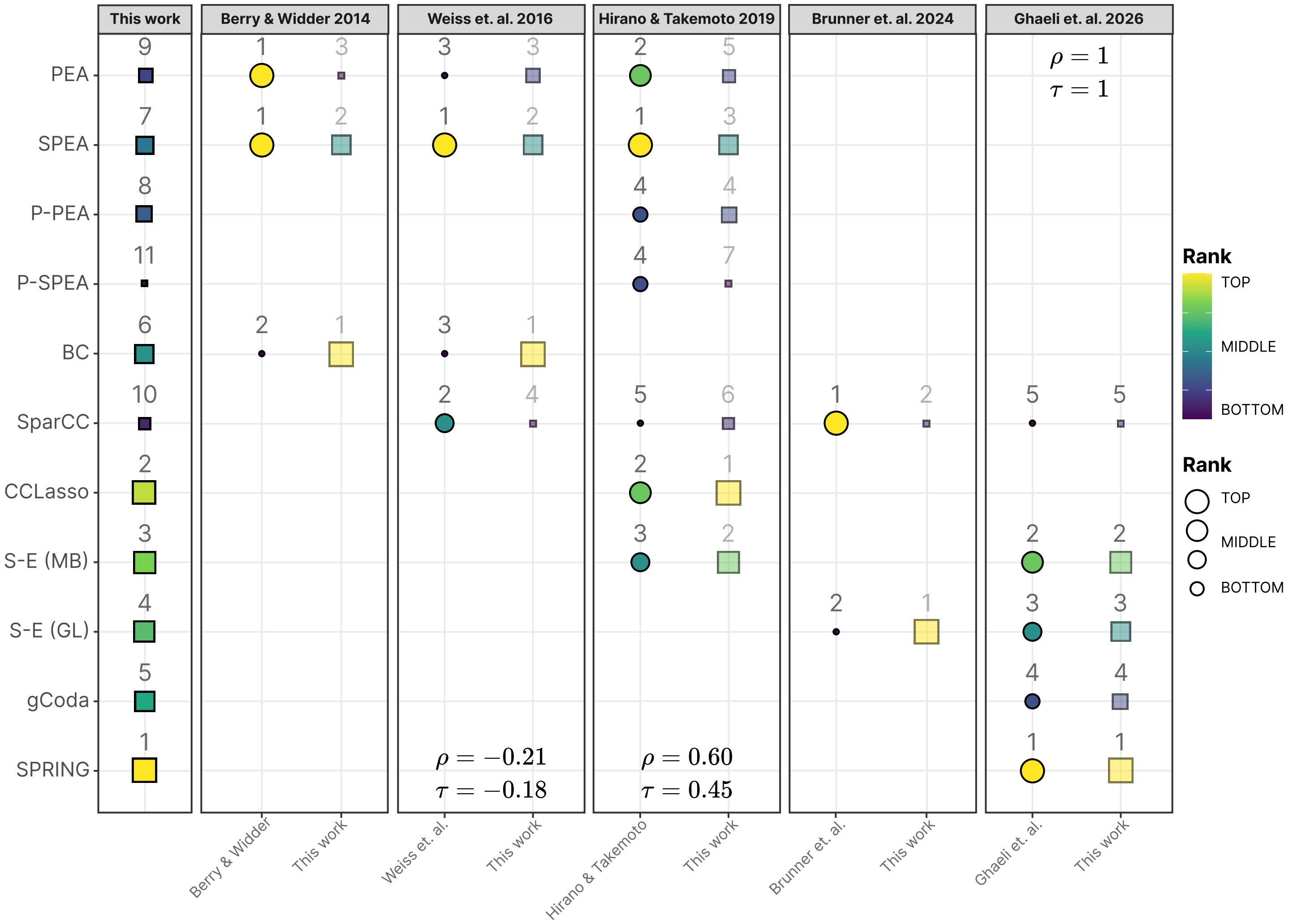

Fig A – Ranking comparison. *Each panel displays a partial ranking of network inference methods. For every study, this partial ranking includes the overlap between the methods examined in this work and those included in the earlier benchmark. The first panel ("This work") presents the ranking, according to this study, for the union of these overlaps (i.e., all methods that appeared in at least one previous study). Within the subsequent panels, circles indicate the ranking from the prior benchmarks, whereas the faded squares show how the same methods rank based on the results of this study. For studies for which the intersection had more or equal to 4 methods the corresponding panel reports the Spearman's ρ and Kendall's τ correlations between the ranking from this work and that from the corresponding previous benchmark, computed using only the methods shown in the panel.*

2 Graph model and diversity exert consistent and substantial effects on performance

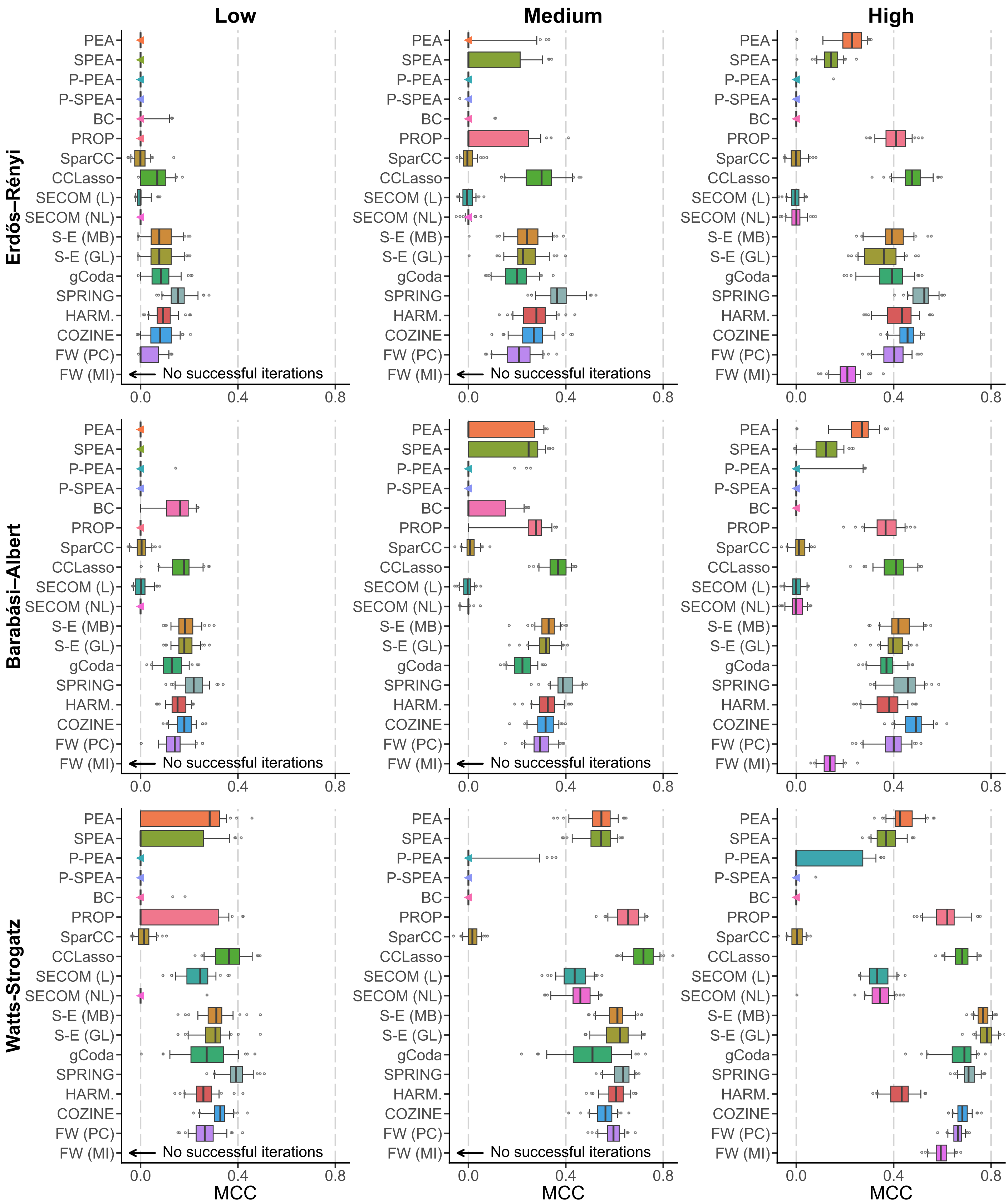

Fig B – MCC for different graph models and β-diversity. *16S rDNA-seq dataset were generated with different graph generation models $\mathcal{M}$ = ER, BA, WS (on the rows) and different levels of $\beta-$diversity between samples (on the columns). Other simulation parameters are fixed $n = 100, \rho = 5\%,\ p = 50,\ r_{neg} = 0.05, K = 100\%$. As reported in the figure, FlashWeave (MI) had no successful runs over the 50 iterations for low and medium $\beta-$diversity.*

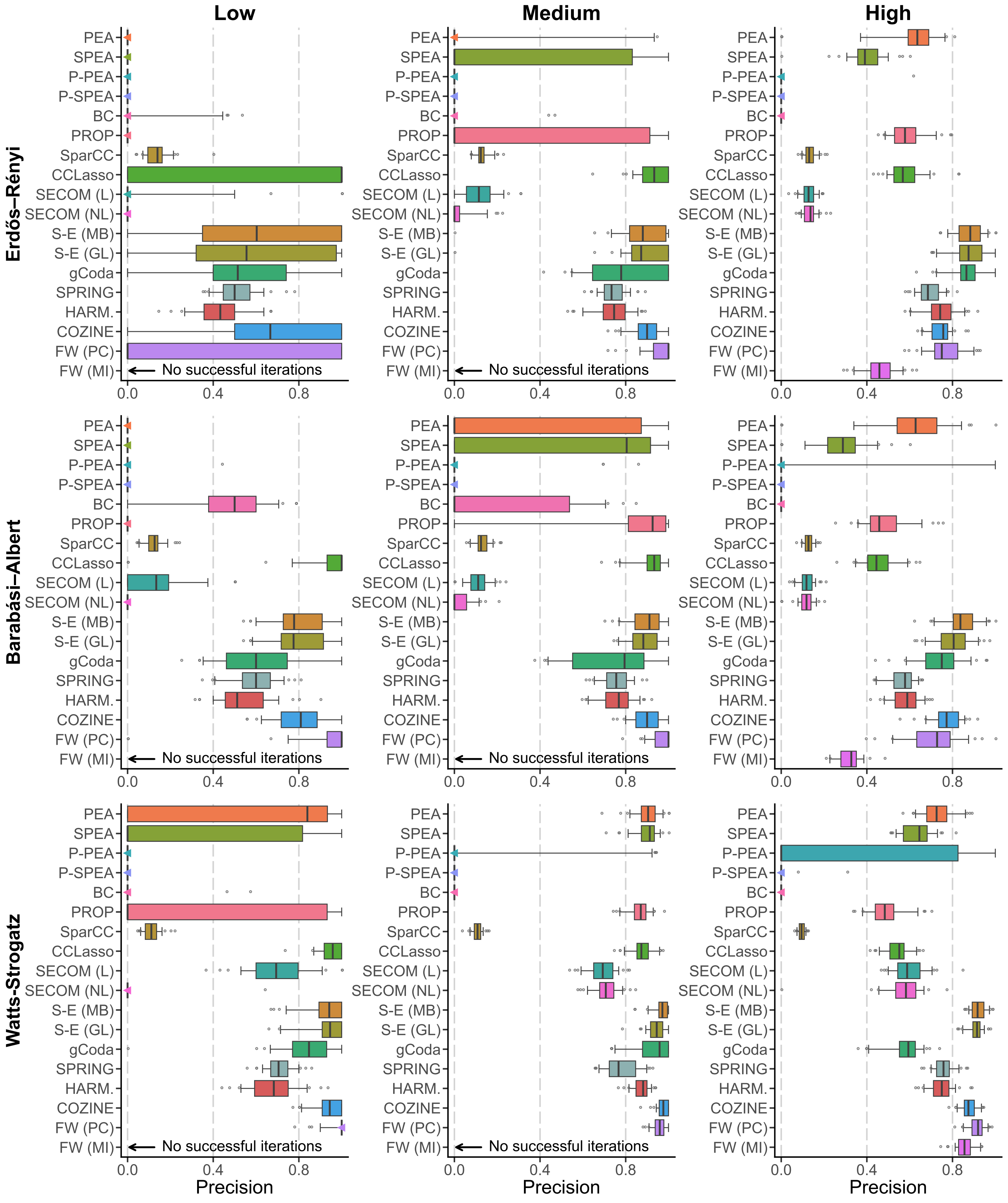

Fig C – Precision for different graph models and β-diversity. *16S rDNA-seq dataset were generated with different graph generation models $\mathcal{M}$ = ER, BA, WS (on the rows) and different levels of $\beta-$diversity between samples (on the columns). Other simulation parameters are fixed $n = 100, \rho(\mathcal{G}) = 5\%,\ p = 50,\ r_{neg} = 0.05, K = 100\%$. As reported in the figure, FlashWeave (MI) had no successful runs over the 50 iterations for low and medium $\beta-$diversity.*

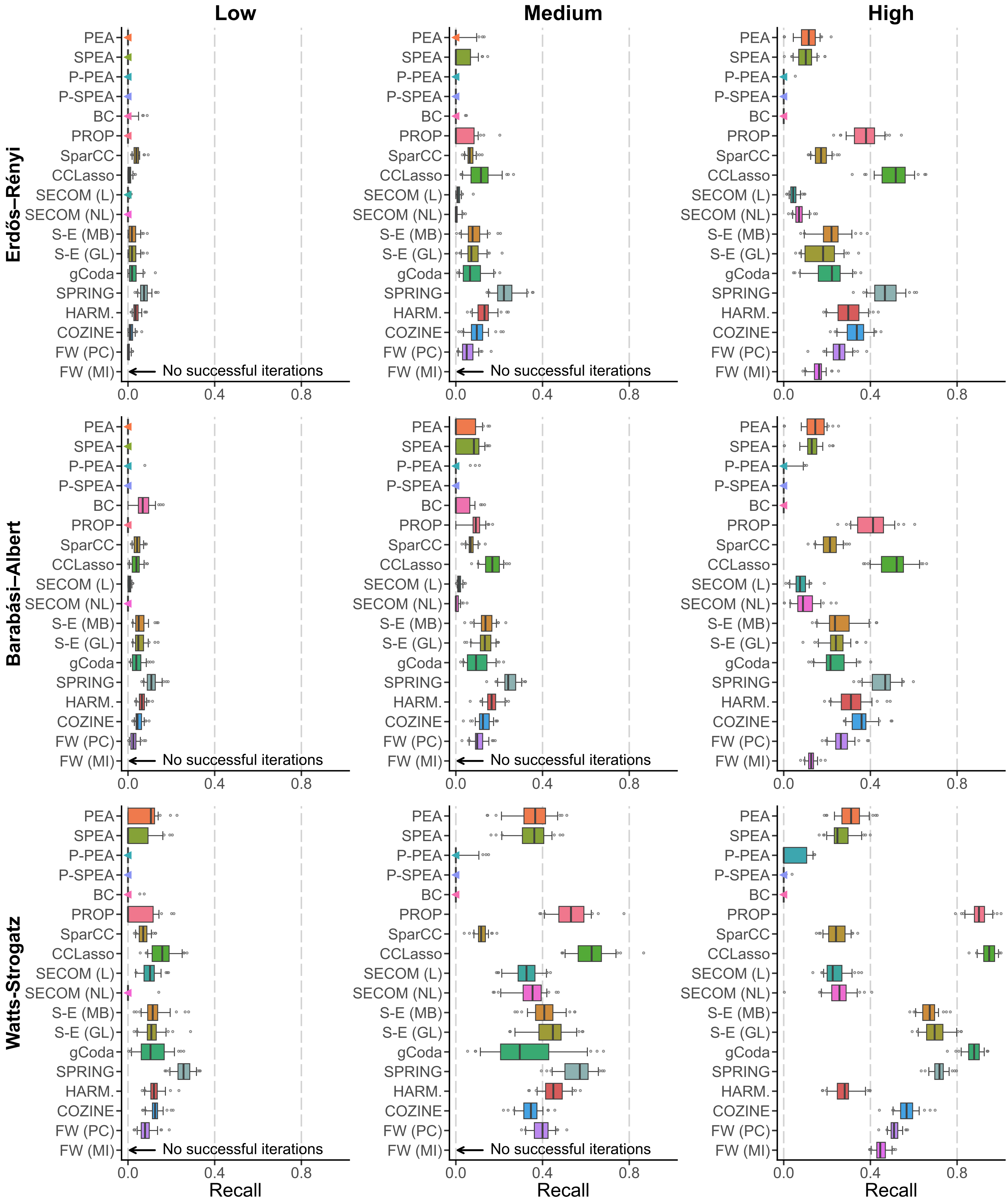

Fig D – Recall for different graph models and β-diversity. *16S rDNA-seq dataset were generated with different graph generation models $\mathcal{M}$ = ER, BA, WS (on the rows) and different levels of $\beta-$diversity between samples (on the columns). Other simulation parameters are fixed $n = 100, \rho(\mathcal{G}) = 5\%,\ p = 50,\ r_{neg} = 0.05, K = 100\%$. As reported in the figure, FlashWeave (MI) had no successful runs over the 50 iterations for low and medium $\beta-$diversity.*

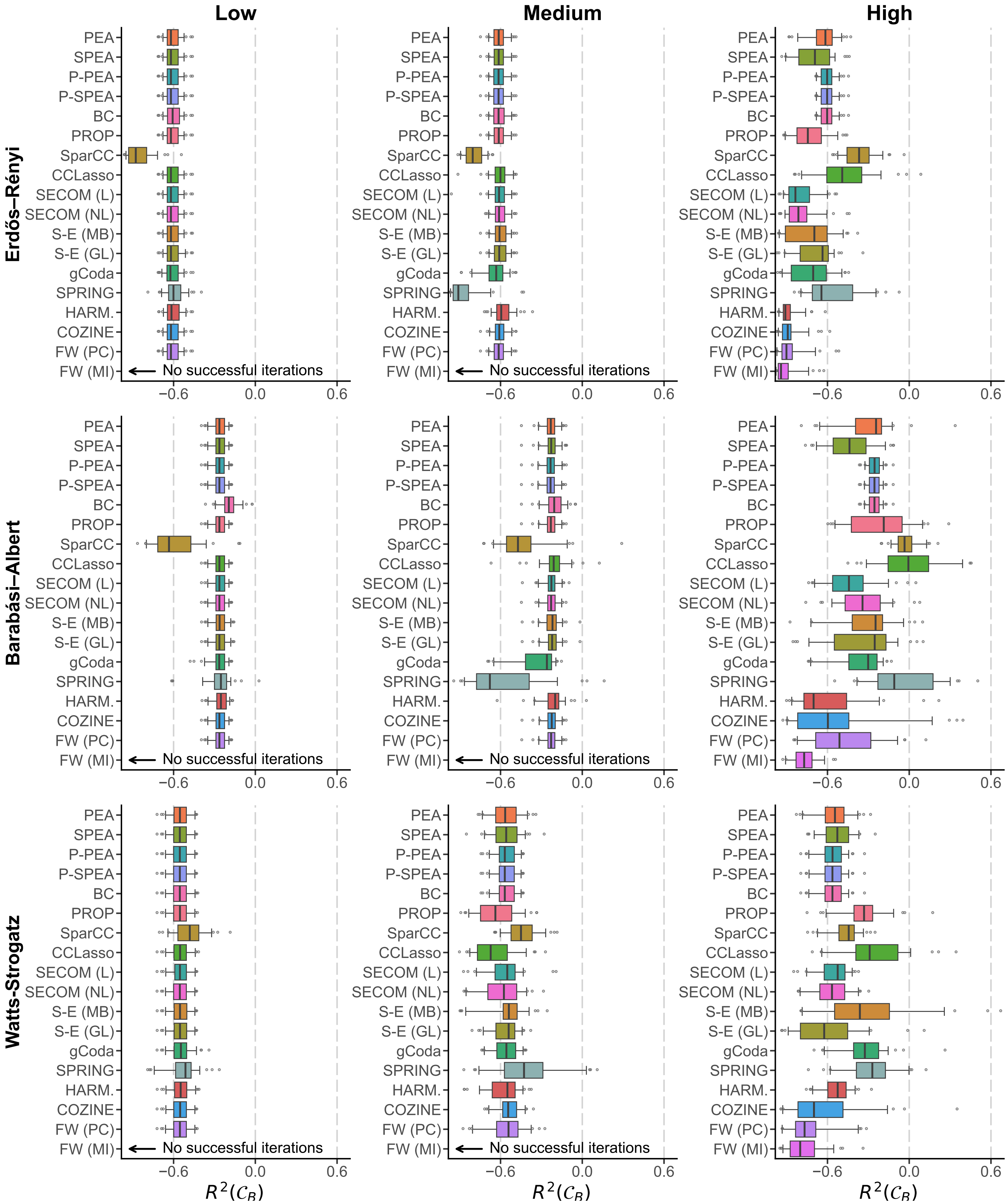

Fig E – **R^2 of betweenness centrality for different graph models and β-diversity**. *16S rDNA-seq dataset were generated with different graph generation models $\mathcal{M}$ = ER, BA, WS (on the rows) and different levels of $\beta-$diversity between samples (on the columns). Other parameters simulation are fixed $n = 100, \rho(\mathcal{G}) = 5\%,\ p = 50,\ r_{neg} = 0.05, K = 100\%$. As reported in the figure, FlashWeave (MI) had no successful runs over the 50 iterations for low and medium $\beta-$diversity.*

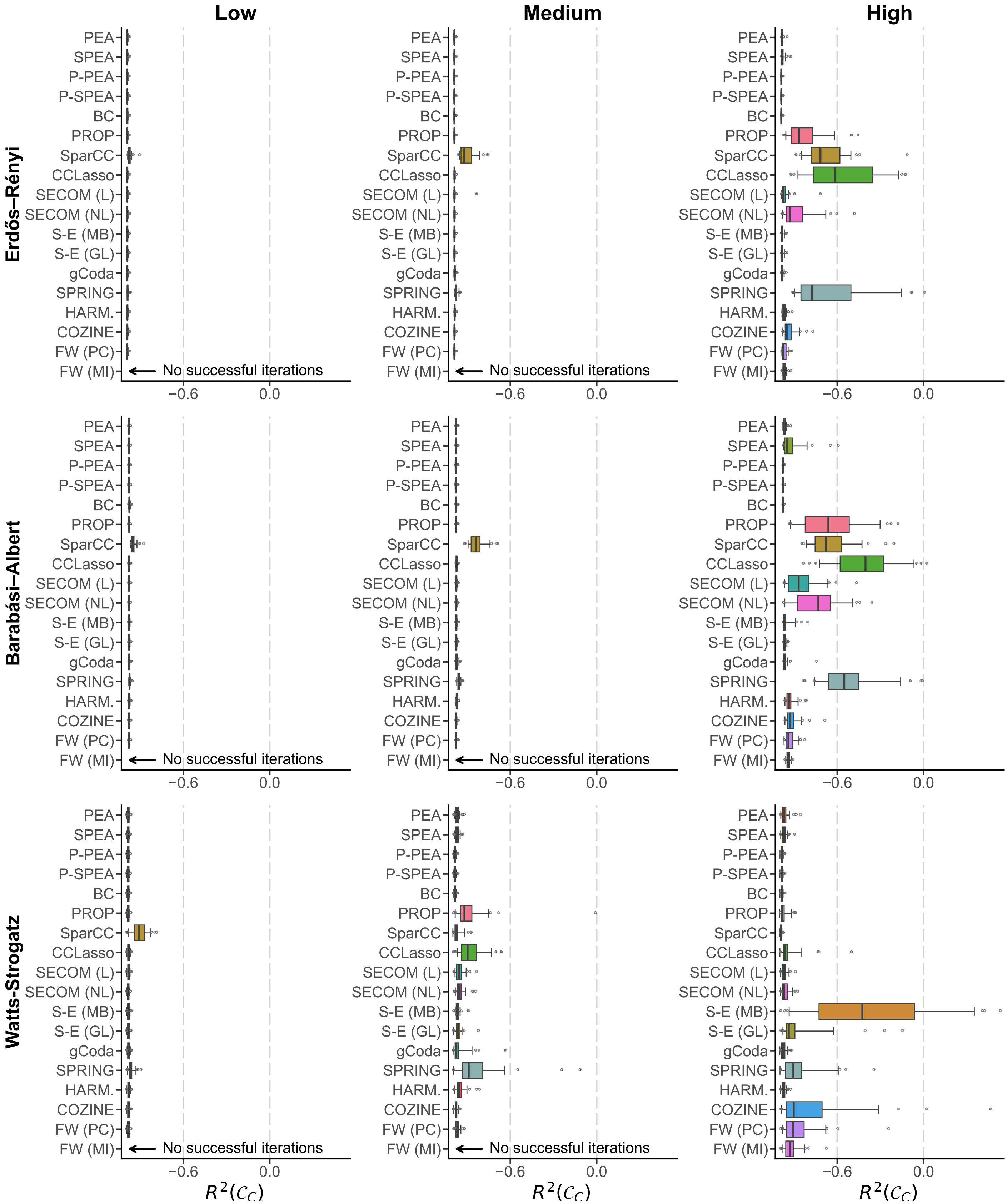

Fig F – R^2 of closeness centrality for different graph models and β-diversity. *16S rDNA-seq dataset were generated with different graph generation models $\mathcal{M}$ = ER, BA, WS (on the rows) and different levels of $\beta-$diversity between samples (on the columns). Other simulation parameters are fixed $n = 100, \rho(\mathcal{G}) = 5\%,\ p = 50,\ r_{neg} = 0.05, K = 100\%$. As reported in the figure, FlashWeave (MI) had no successful runs over the 50 iterations for low and medium $\beta-$diversity.*

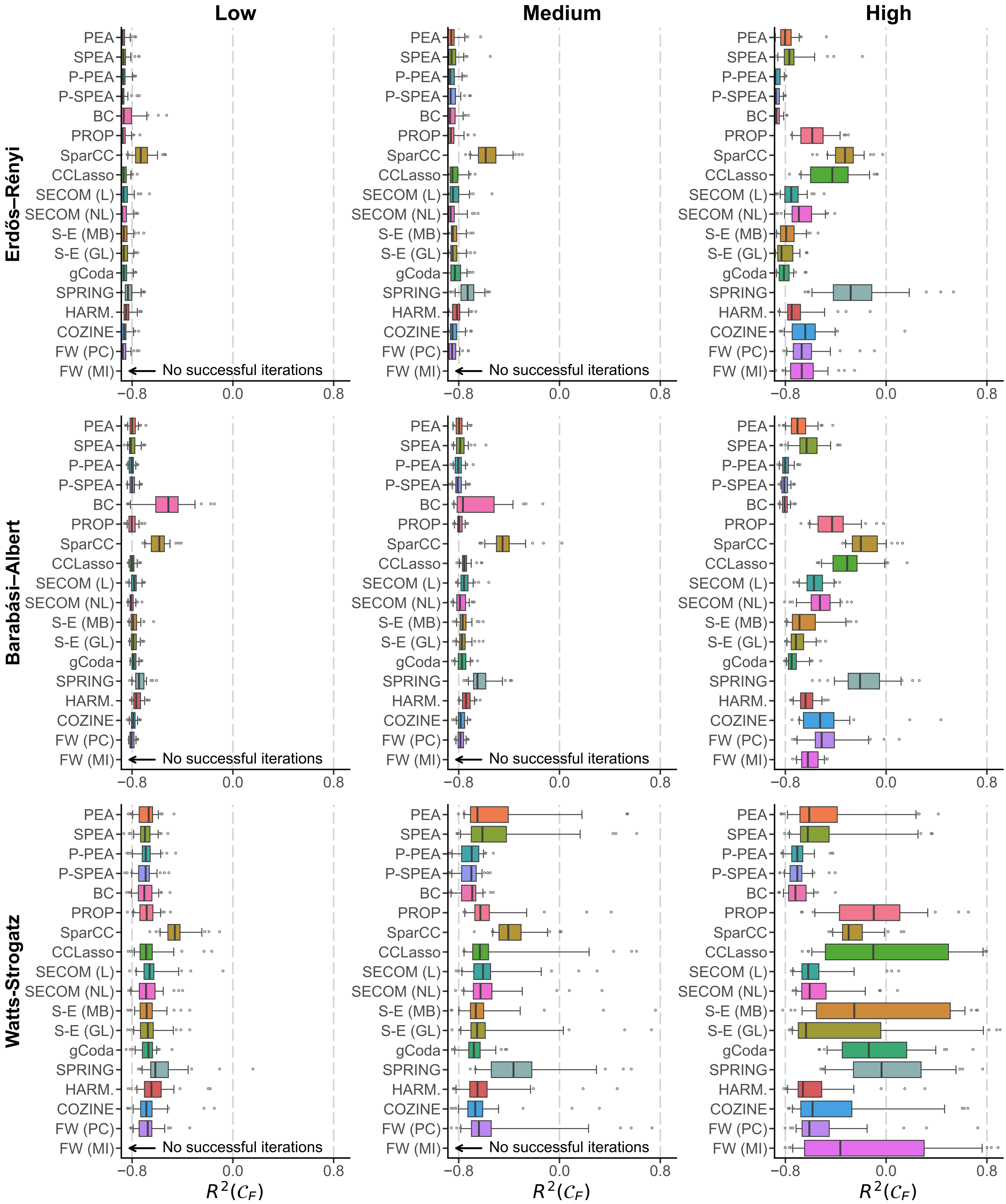

Fig G – R^2 of eigenvector centrality for different graph models and β-diversity. *16S rDNA-seq dataset were generated with different graph generation models $\mathcal{M}$ = ER, BA, WS (on the rows) and different levels of $\beta-$diversity between samples (on the columns). Other simulation parameters are fixed* $n = 100, \rho(\mathcal{G}) = 5\%,\ p = 50,\ r_{neg} = 0.05, K = 100\%$. *As reported in the figure, FlashWeave (MI) had no successful runs over the 50 iterations for low and medium $\beta-$diversity.*

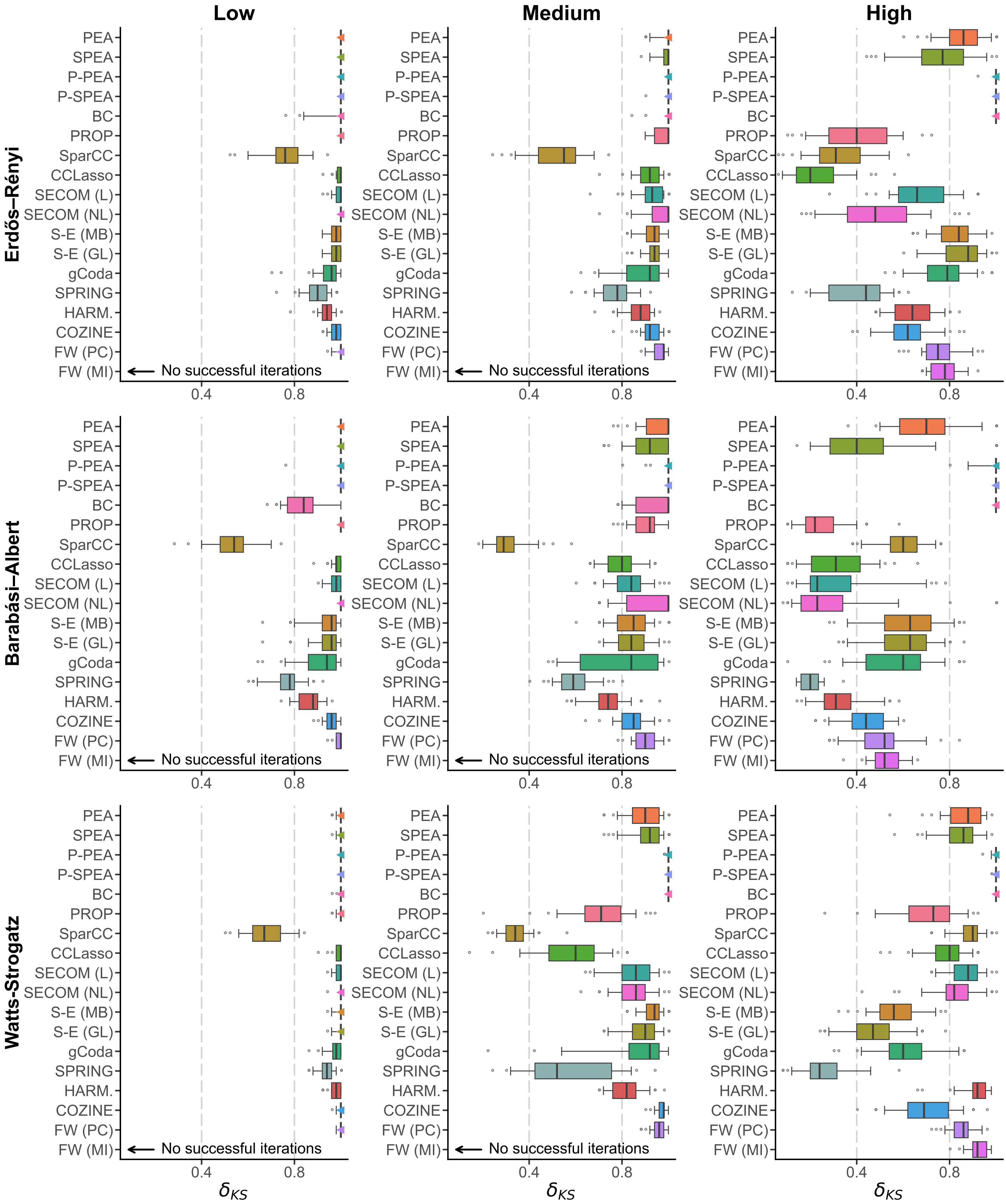

Fig H – Kolmogorov-Smirnov statisitics for different graph models and β-diversity. *16S rDNA-seq dataset were generated with different graph generation models $\mathcal{M}$ = ER, BA, WS (on the rows) and different levels of $\beta-$diversity between samples (on the columns). Other simulation parameters are fixed $n = 100, \rho(\mathcal{G}) = 5\%,\ p = 50,\ r_{neg} = 0.05, K = 100\%$. As reported in the figure, FlashWeave (MI) had no successful runs over the 50 iterations for low and medium $\beta-$diversity.*

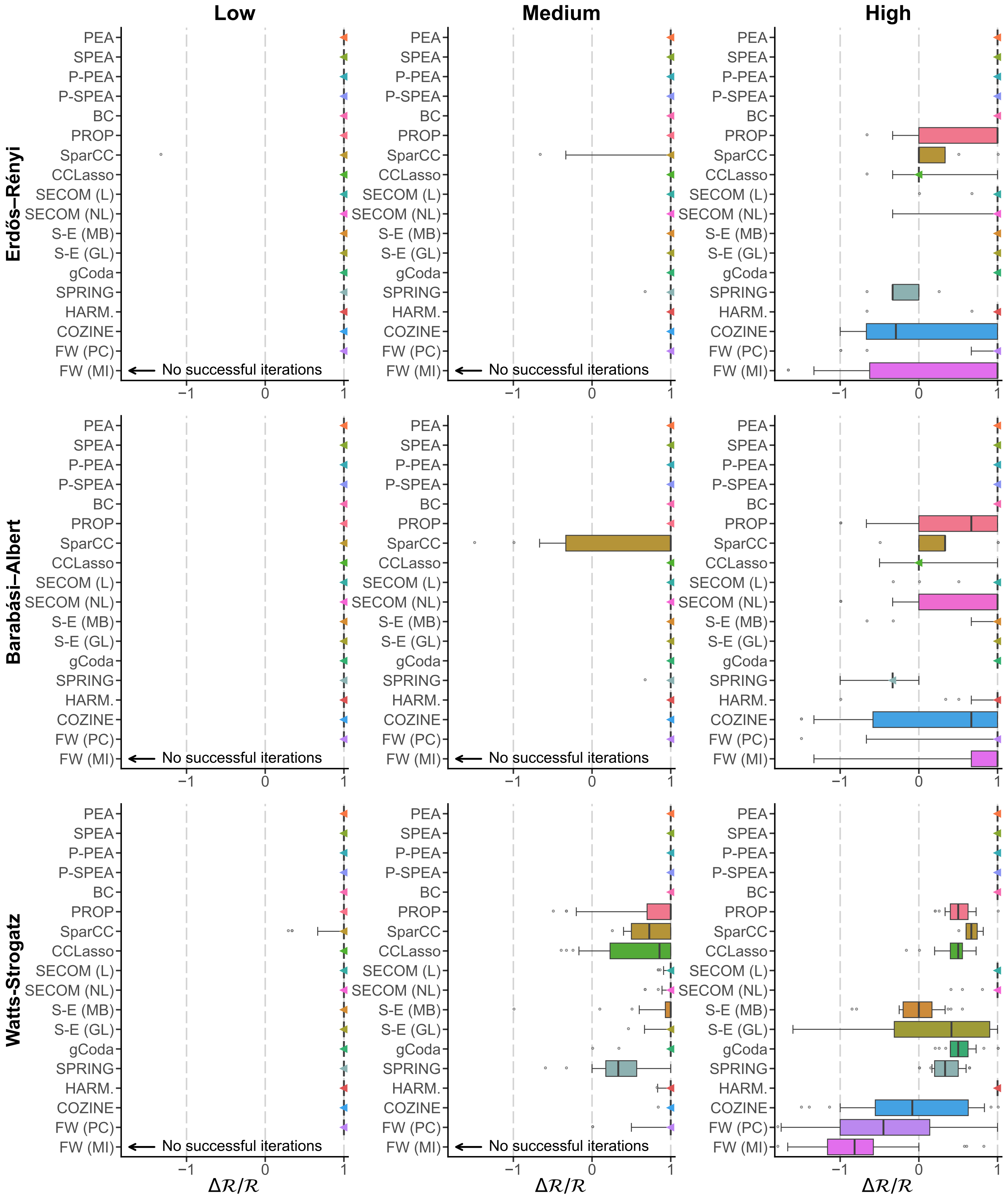

Fig I – Delta in radius for different graph models and β-diversity. *16S rDNA-seq dataset were generated with different graph generation models $\mathcal{M}$ = ER, BA, WS (on the rows) and different levels of $\beta-$diversity between samples (on the columns). Other simulation parameters are fixed* $n = 100, \rho(\mathcal{G}) = 5\%,\ p = 50,\ r_{neg} = 0.05, K = 100\%$. *As reported in the figure, FlashWeave (MI) had no successful runs over the 50 iterations for low and medium $\beta-$diversity.*

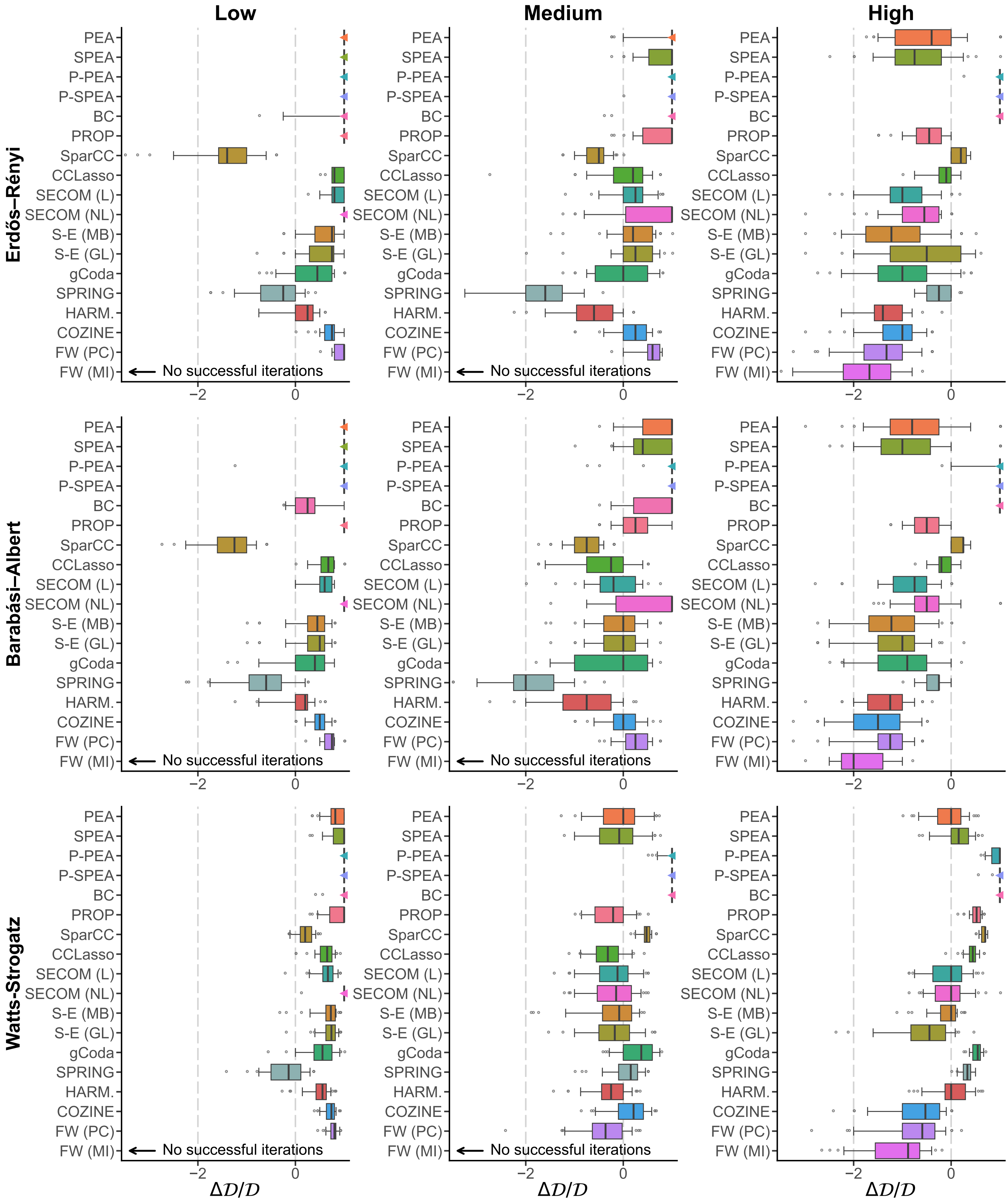

Fig J – Delta in diameter for different graph models and β-diversity. *16S rDNA-seq dataset were generated with different graph generation models $\mathcal{M}$ = ER, BA, WS (on the rows) and different levels of $\beta-$diversity between samples (on the columns). Other simulation parameters are fixed $n = 100, \rho(\mathcal{G}) = 5\%,\ p = 50,\ r_{neg} = 0.05, K = 100\%$. As reported in the figure, FlashWeave (MI) had no successful runs over the 50 iterations for low and medium $\beta-$diversity.*

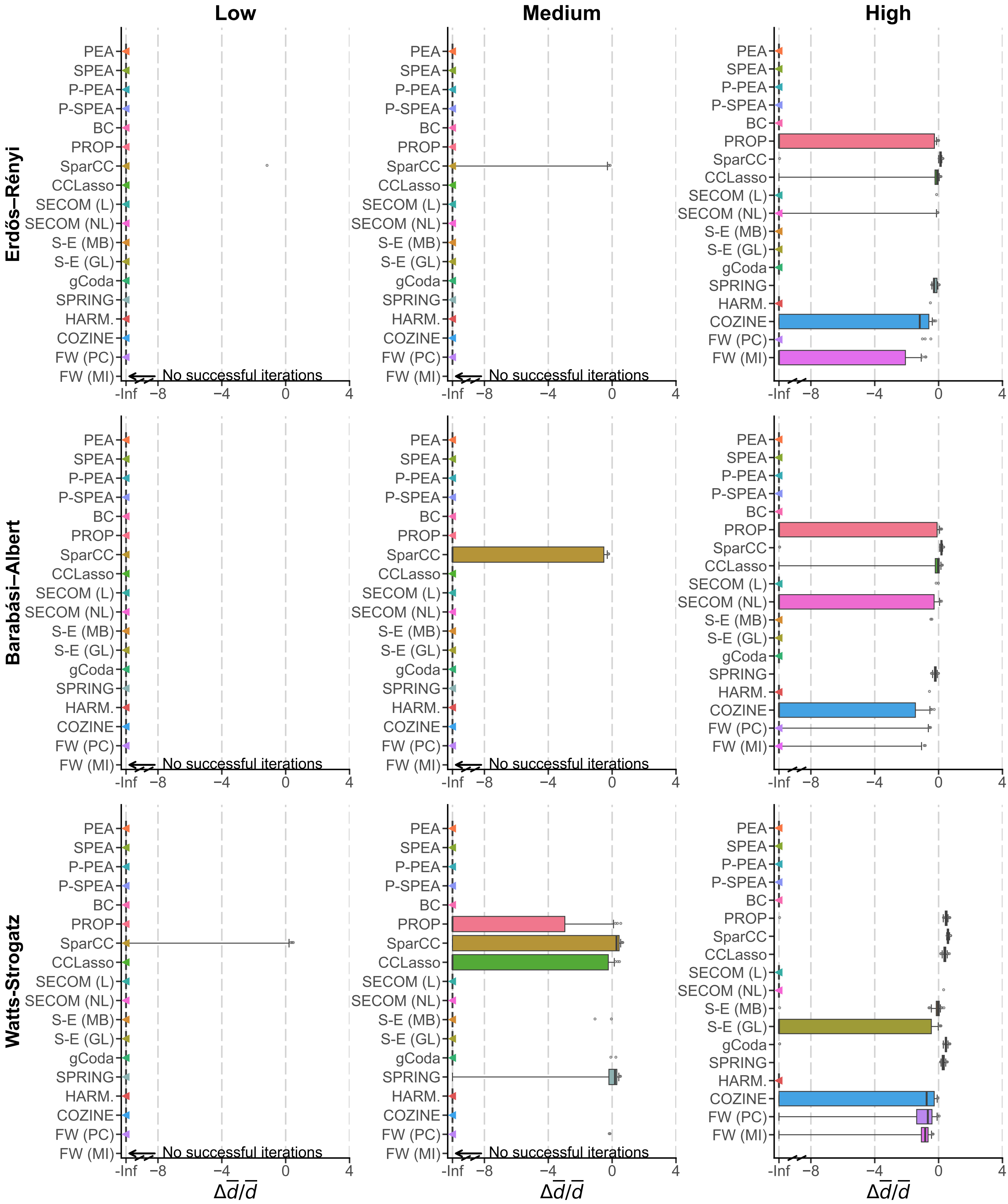

Fig K – Delta in average distance for different graph models and β-diversity. *16S rDNA-seq dataset were generated with different graph generation models $\mathcal{M}$ = ER, BA, WS (on the rows) and different levels of $\beta-$diversity between samples (on the columns). Other simulation parameters are fixed $n = 100, \rho(\mathcal{G}) = 5\%,\ p = 50,\ r_{neg} = 0.05, K = 100\%$. As reported in the figure, FlashWeave (MI) had no successful runs over the 50 iterations for low and medium $\beta-$diversity.*

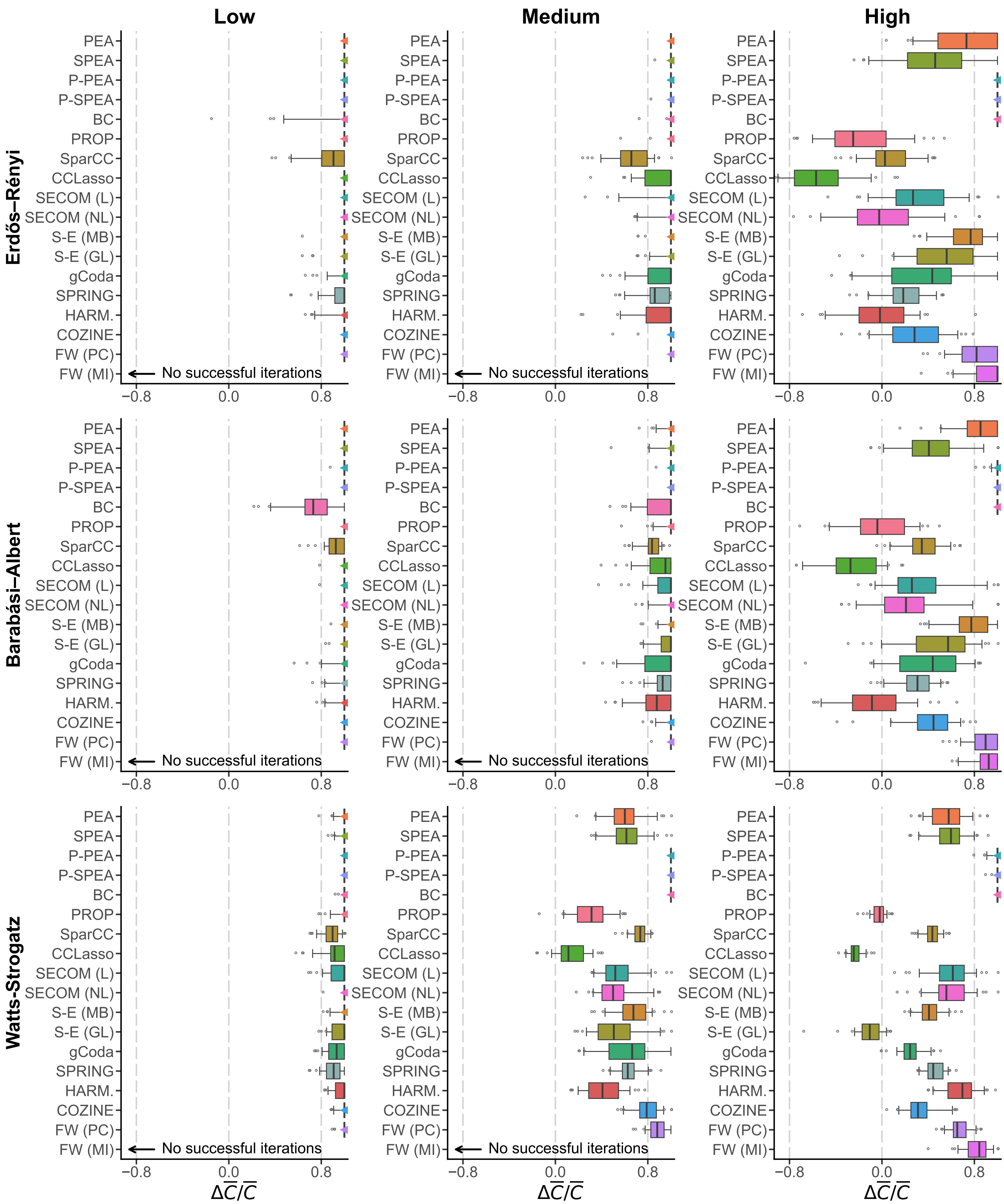

Fig L – Delta in average clustering coefficient for different graph models and β-diversity. *16S rDNA-seq dataset were generated with different graph generation models $\mathcal{M}$ = ER, BA, WS (on the rows) and different levels of $\beta-$diversity between samples (on the columns). Other simulation parameters are fixed $n = 100, \rho(\mathcal{G}) = 5\%,\ p = 50,\ r_{neg} = 0.05, K = 100\%$. As reported in the figure, FlashWeave (MI) had no successful runs over the 50 iterations for low and medium $\beta-$diversity.*

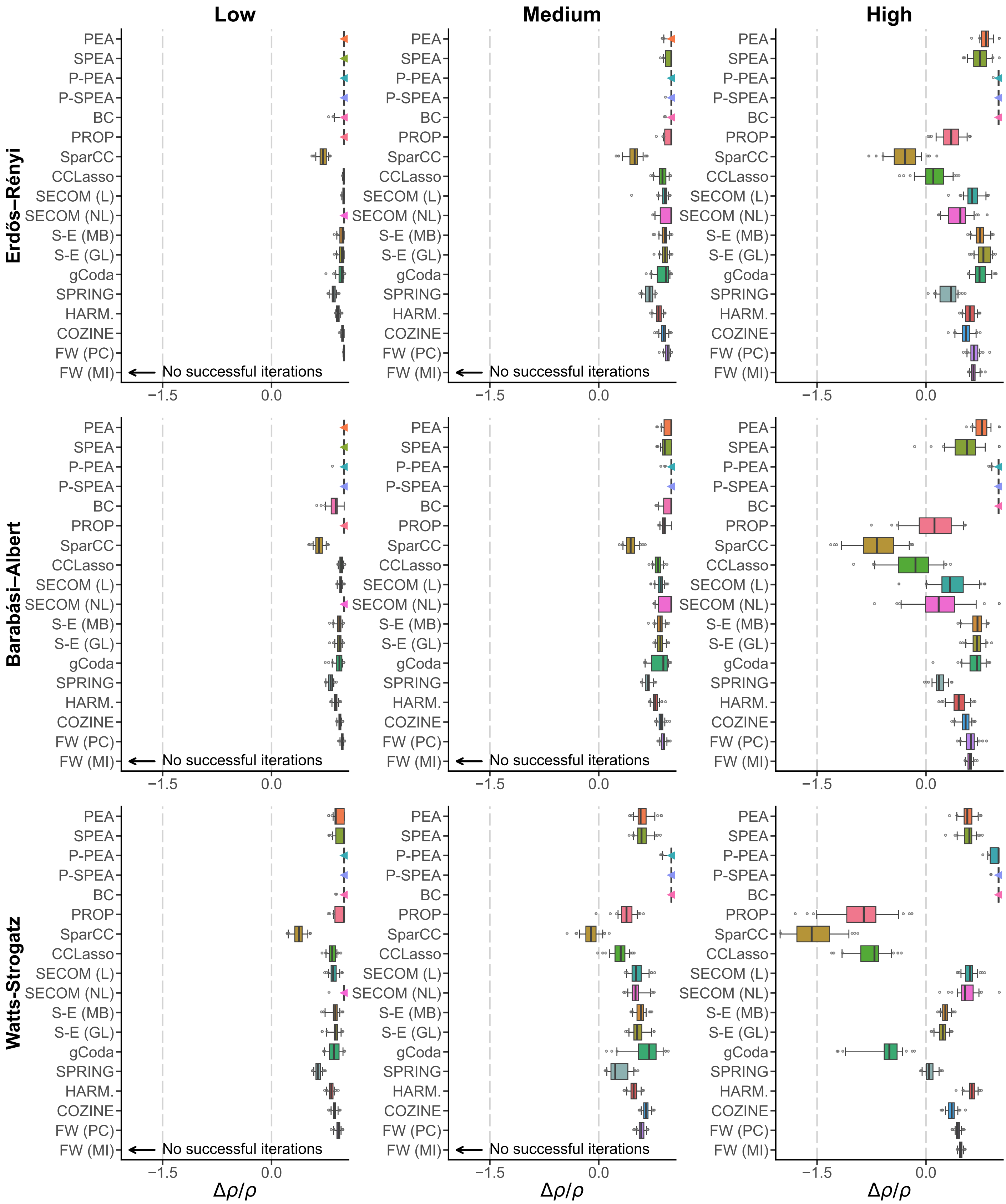

Fig M – Delta in density for different graph models and β-diversity. *16S rDNA-seq dataset were generated with different graph generation models $\mathcal{M}$ = ER, BA, WS (on the rows) and different levels of $\beta-$diversity between samples (on the columns). Other simulation parameters are fixed $n = 100, \rho(\mathcal{G}) = 5\%,\ p = 50,\ r_{neg} = 0.05, K = 100\%$. As reported in the figure, FlashWeave (MI) had no successful runs over the 50 iterations for low and medium $\beta-$diversity.*

3 Increasing sample size improves network inference performance

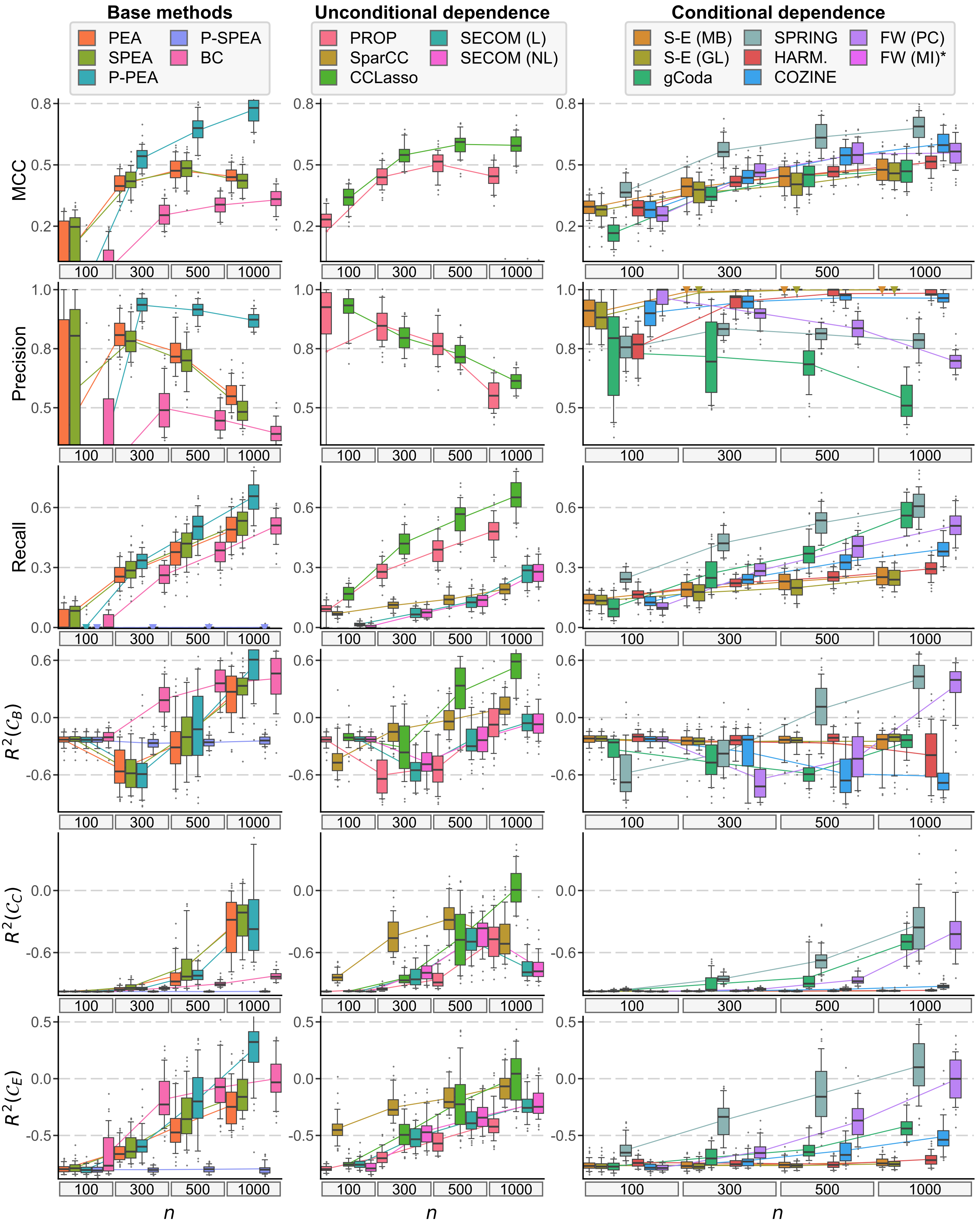

Fig N – Edge-wise and node-wise metrics for BA model with medium $\beta-$diversity at different sample sizes. *16S rDNA-seq dataset simulations were performed with different sample sizes $n \in [50, 75, 100, 200, 300, 500, 1000]$ (here we show a representative subset of the values). All other simulation parameters were set at their default value $\rho(\mathcal{G}) = 5\%$, $p = 50$, $r_{neg} = 0.05, K = 100\%$. Methods are grouped based on the implementation strategy (different columns). Note that it may happen that the displayed metric over the 50 iterations had the same value, meaning that boxplots collapse to a single line. In such cases, we plotted a coloured triangle corresponding to the median value of the metric over the 50 iterations.*

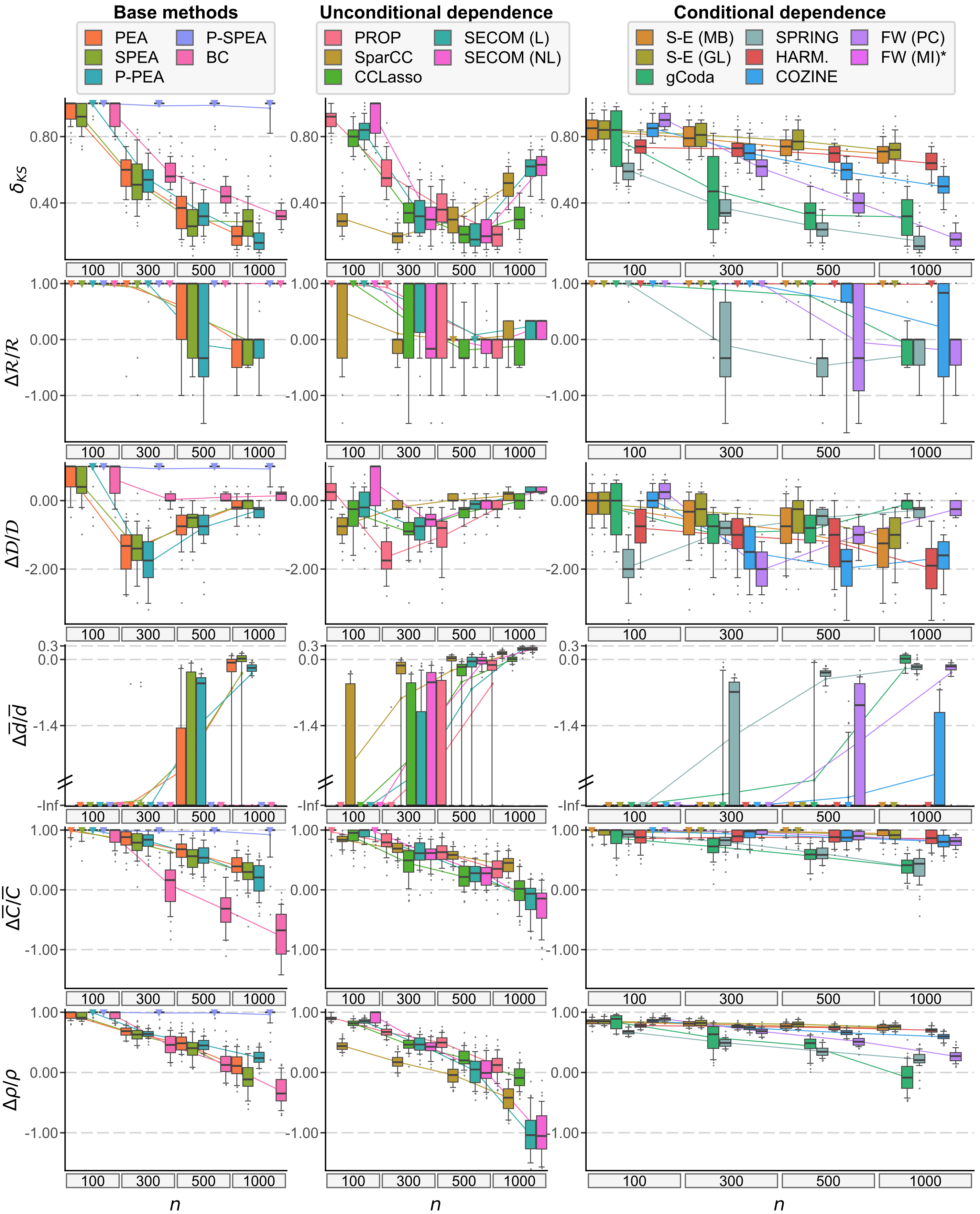

Fig O – Graph-wise metrics for BA model with medium β−diversity at different sample sizes. *16S rDNA-seq dataset simulations were performed with different sample sizes* $n \in [50, 75, 100, 200, 300, 500, 1000]$ *(here we show a representative subset of the values). All other simulation parameters were set at their default value* $\rho(\mathcal{G}) = 5\%,\ p = 50,\ r_{neg} = 0.05, K = 100\%$. *Methods are grouped based on the implementation strategy (different columns). Note that it may happen that the displayed metric over the 50 iterations had the same value, meaning that boxplots collapse to a single line. In such cases, we plotted a coloured triangle corresponding to the median value of the metric over the 50 iterations.*

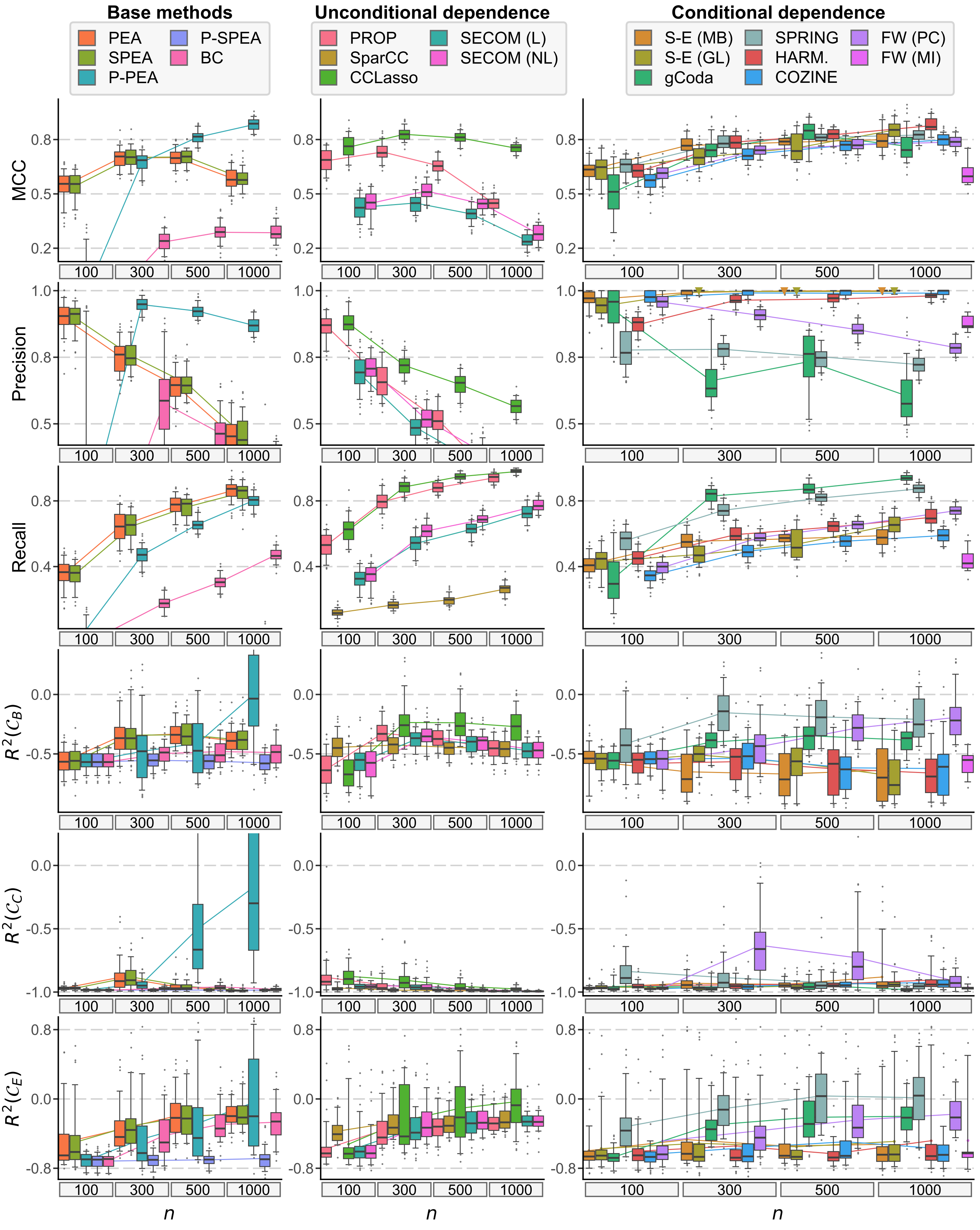

Fig P – Edge-wise and node-wise metrics for WS model with medium β−diversity at different sample sizes. *16S rDNA-seq dataset simulations were performed with different sample sizes $n \in [50, 75, 100, 200, 300, 500, 1000]$ (here we show a representative subset of the values). All other simulation parameters were set at their default value $\rho(\mathcal{G}) = 5\%$, $p = 50$, $r_{neg} = 0.05, K = 100\%$. Methods are grouped based on the implementation strategy (different columns). Note that it may happen that the displayed metric over the 50 iterations had the same value, meaning that boxplots collapse to a single line. In such cases, we plotted a coloured triangle corresponding to the median value of the metric over the 50 iterations.*

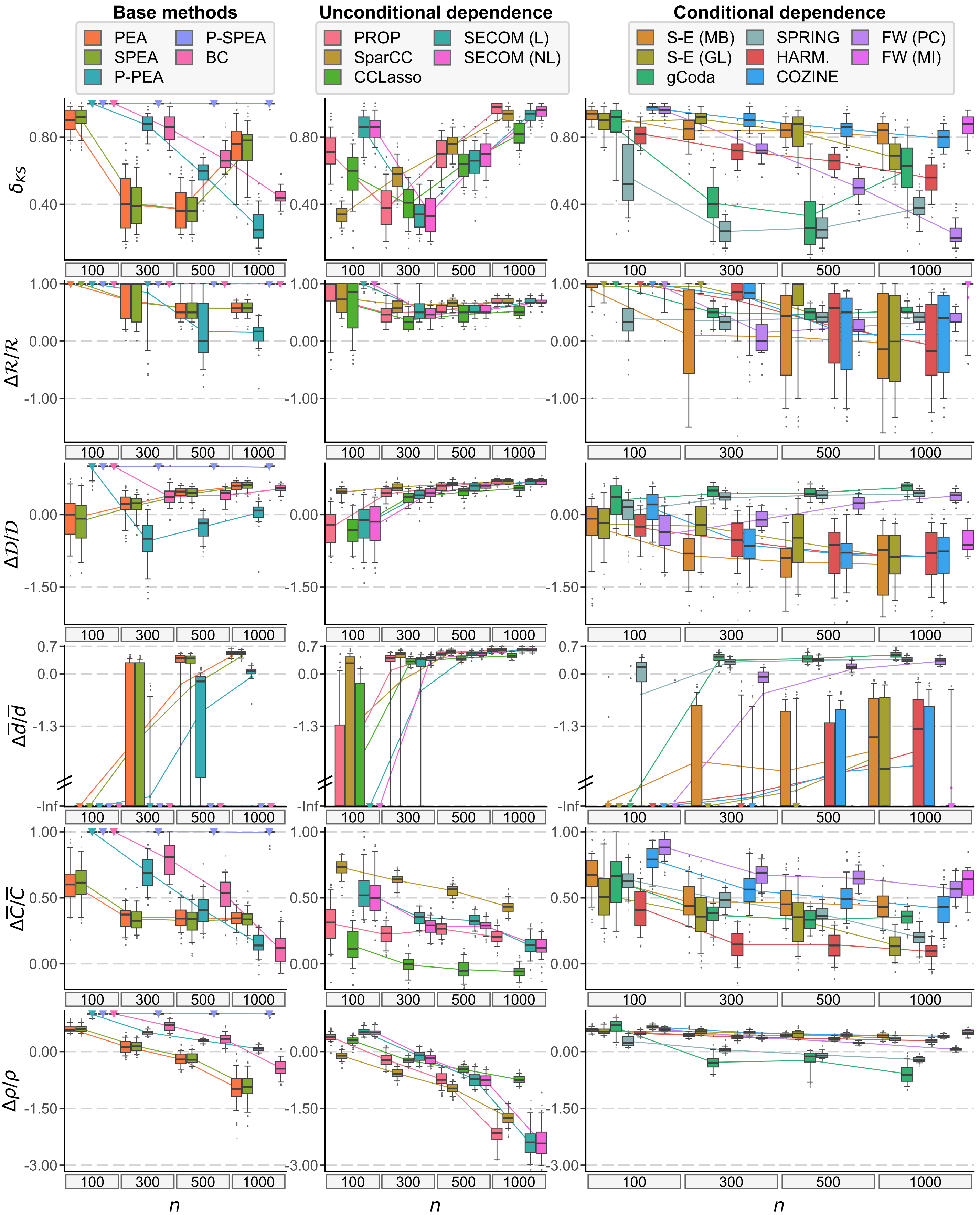

Fig Q – Graph-wise metrics for WS model with medium $\beta-$diversity at different sample sizes. *16S rDNA-seq dataset simulations were performed with different sample sizes $n \in [50, 75, 100, 200, 300, 500, 1000]$ (here we show a representative subset of the values). All other simulation parameters were set at their default value $\rho(\mathcal{G}) = 5\%,\ p = 50,\ r_{neg} = 0.05, K = 100\%$. Methods are grouped based on the implementation strategy (different columns). Note that it may happen that the displayed metric over the 50 iterations had the same value, meaning that boxplots collapse to a single line. In such cases, we plotted a coloured triangle corresponding to the median value of the metric over the 50 iterations.*

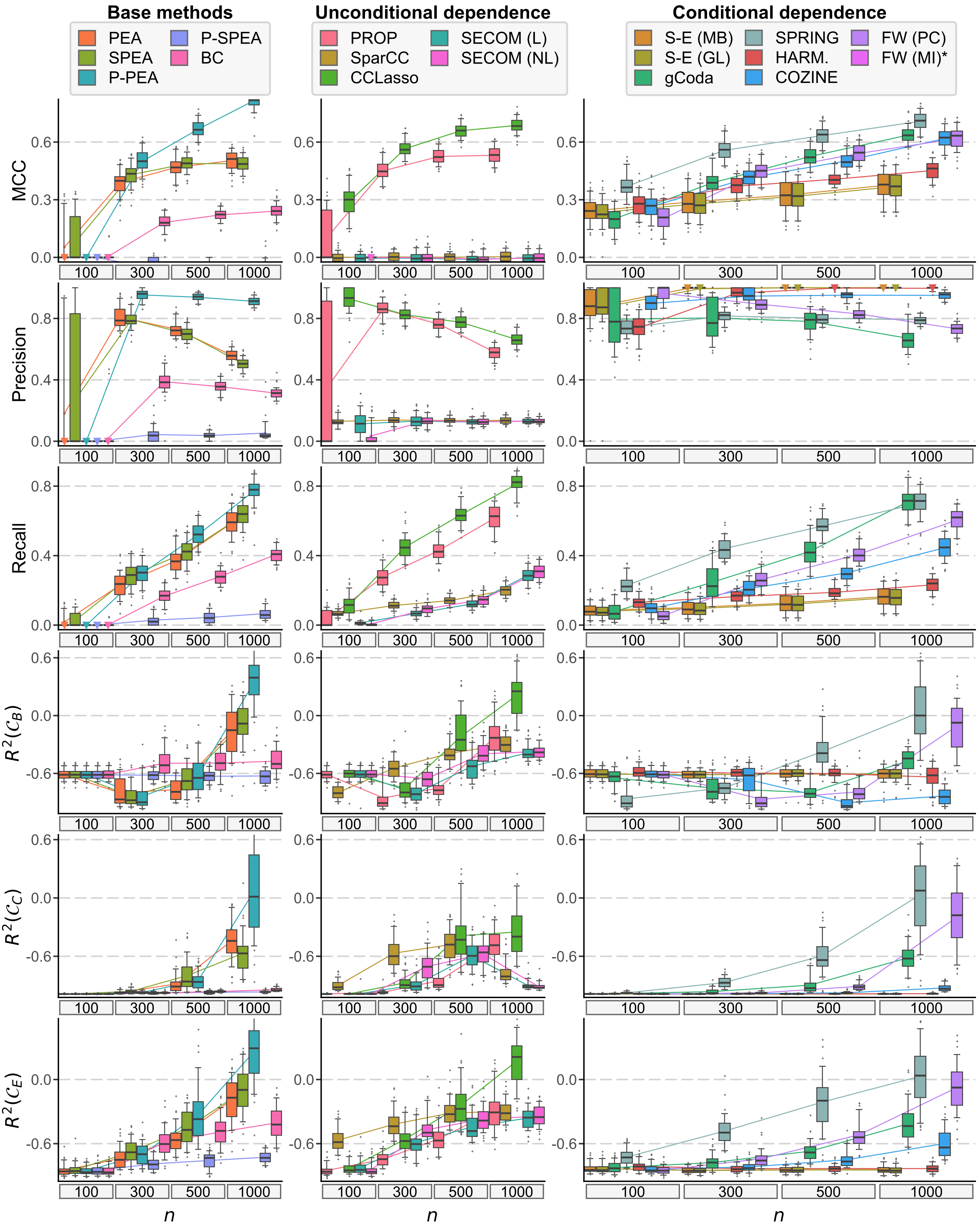

Fig R – Edge-wise and node-wise metrics for ER model with medium $\beta-$diversity at different sample sizes. *16S rDNA-seq dataset simulations were performed with different sample sizes $n \in [50, 75, 100, 200, 300, 500, 1000]$ (here we show a representative subset of the values). All other simulation parameters were set at their default value $\rho(\mathcal{G}) = 5\%$, $p = 50$, $r_{neg} = 0.05, K = 100\%$. Methods are grouped based on the implementation strategy (different columns). Note that it may happen that the displayed metric over the 50 iterations had the same value, meaning that boxplots collapse to a single line. In such cases, we plotted a coloured triangle corresponding to the median value of the metric over the 50 iterations.*

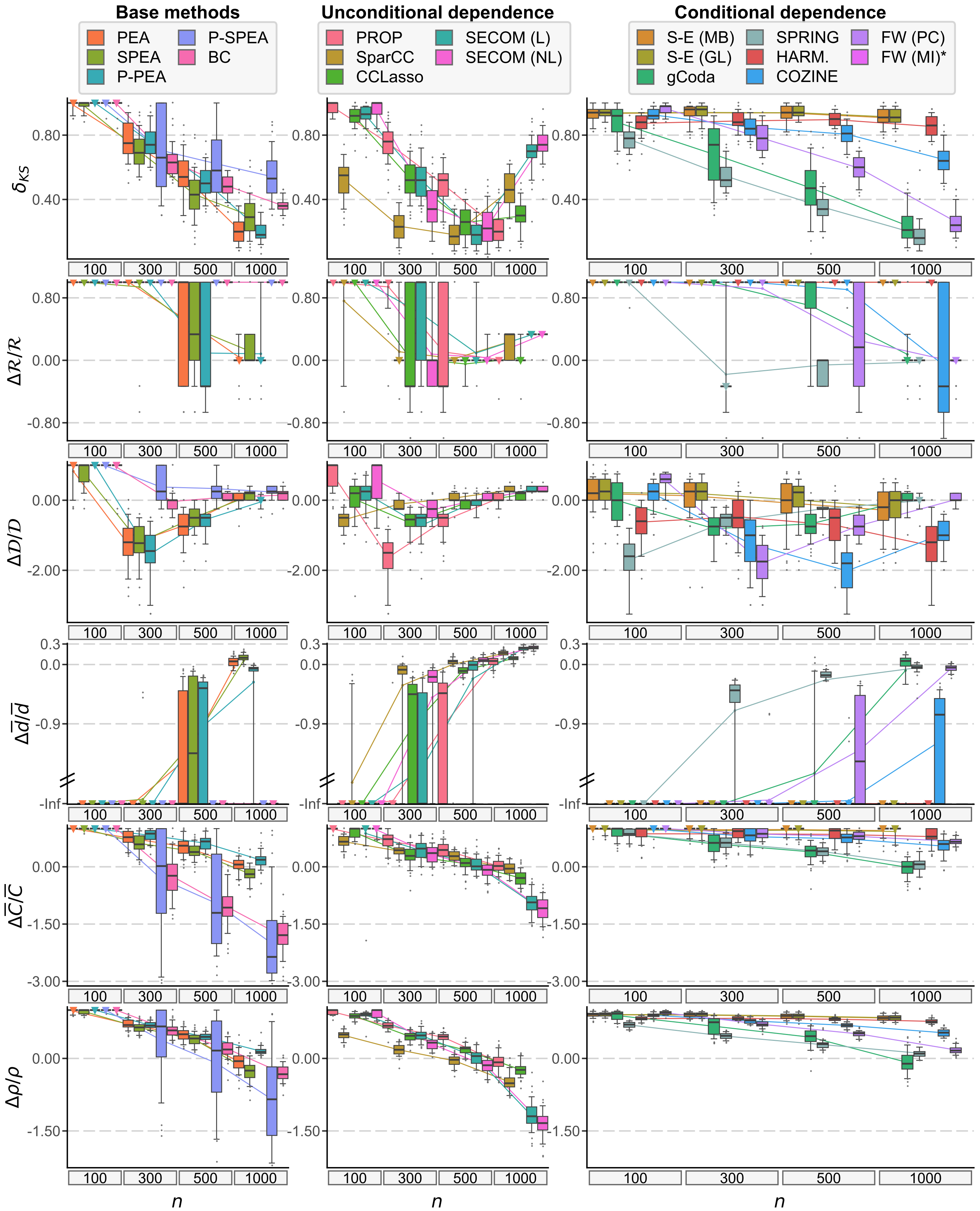

Fig S – Graph-wise metrics for ER model with medium β−diversity at different sample sizes. *16S rDNA-seq dataset simulations were performed with different sample sizes* $n \in [50, 75, 100, 200, 300, 500, 1000]$ *(here we show a representative subset of the values). All other simulation parameters were set at their default value* $\rho(\mathcal{G}) = 5\%,\ p = 50,\ r_{neg} = 0.05, K = 100\%$. *Methods are grouped based on the implementation strategy (different columns). Note that it may happen that the displayed metric over the 50 iterations had the same value, meaning that boxplots collapse to a single line. In such cases, we plotted a coloured triangle corresponding to the median value of the metric over the 50 iterations.*

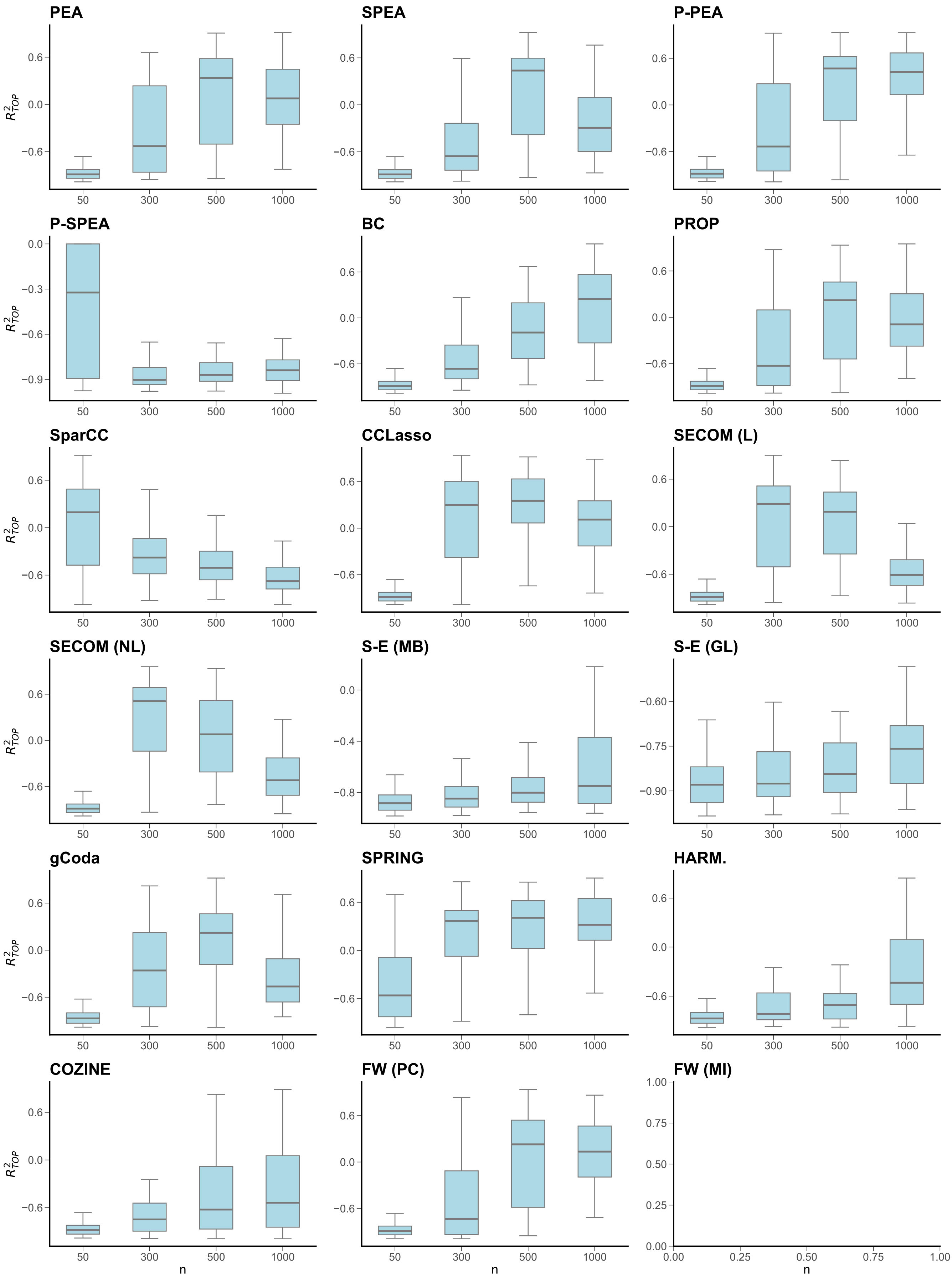

Fig T – $\mathbf{R^2_{TOP}}$ of betweenness centrality for BA model with medium β−diversity at different sample sizes. *16S rDNA-seq dataset simulations were performed with different sample sizes $n \in [50, 75, 100, 200, 300, 500, 1000]$ (here we show a representative subset of the values). All other simulation parameters were set at their default value $\rho(\mathcal{G}) = 5\%,\ p = 50,\ r_{neg} = 0.05, K = 100\%$. For each of the 50 iterations we identified the top 5 nodes with the highest betweenness centrality in the true graph and compared them with the values of betweenness centrality in the inferred graphs.*

4 Community richness affects computational burden and overall accuracy

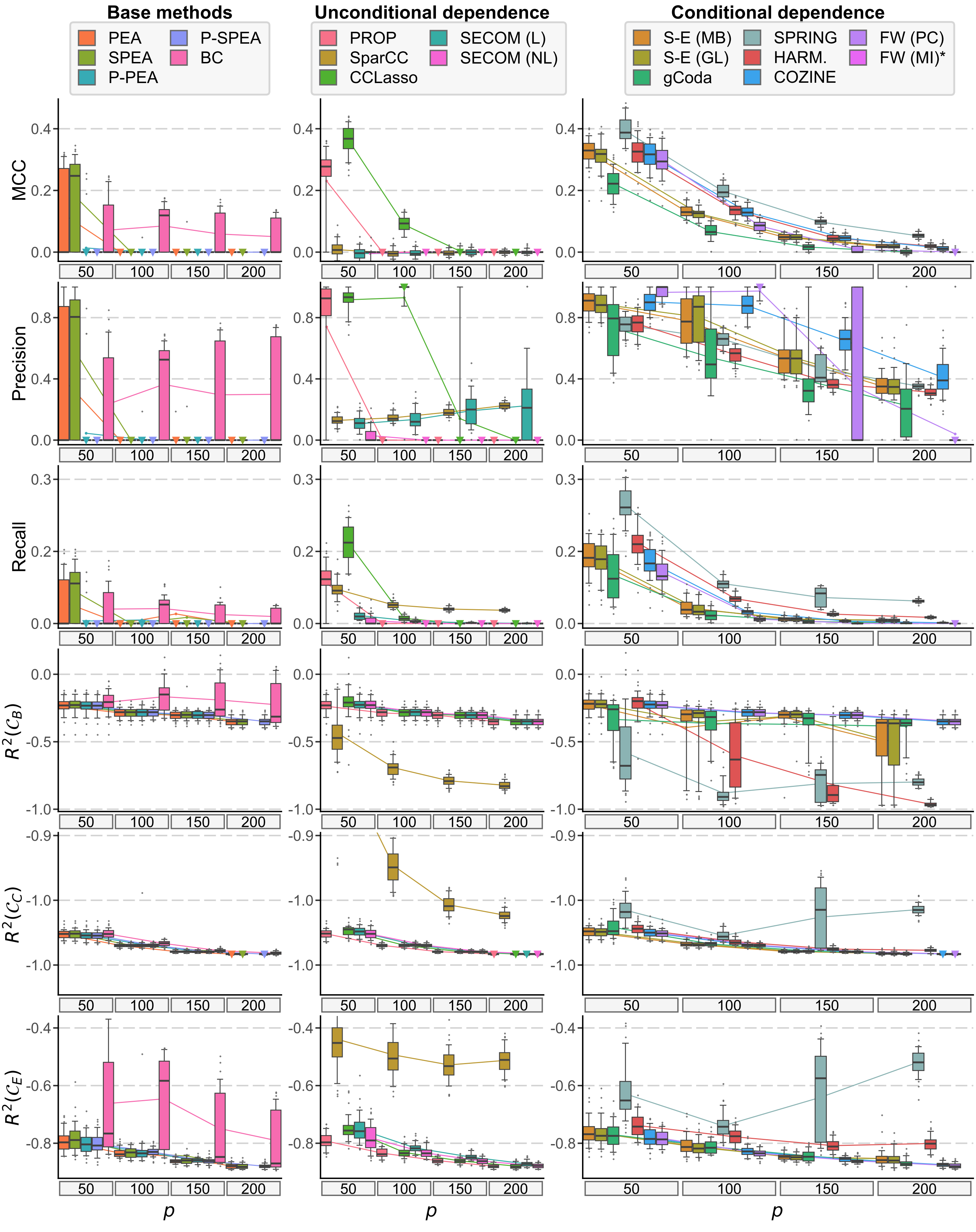

Fig U – Edge-wise and node-wise metrics for BA model with medium $\beta-$diversity for increasing number of species. *16S rDNA-seq dataset simulations were performed with different number of species* $p \in [50, 100, 150, 200]$*. All other simulation parameters were set at their default value* $n = 100$, $\rho(\mathcal{G}) = 5\%$, $r_{neg} = 0.05, K = 100\%$*. Methods are grouped based on the implementation strategy (different columns). Note that it may happen that the displayed metric over the 50 iterations had the same value, meaning that boxplots collapse to a single line. In such cases, we plotted a coloured triangle corresponding to the median value of the metric over the 50 iterations.*

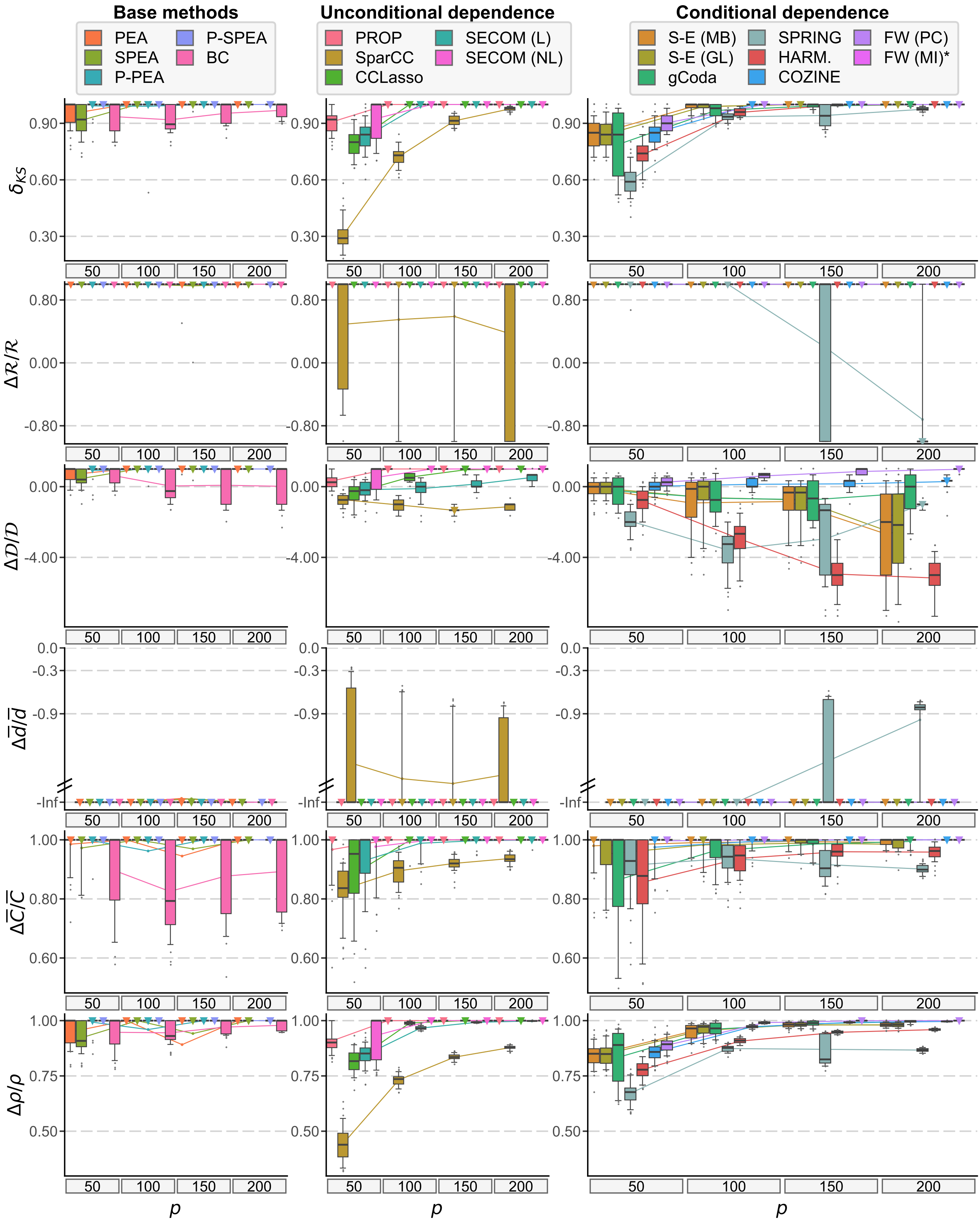

Fig V – Graph-wise metrics for BA model with medium $\beta-$diversity for increasing number of species. *16S rDNA-seq dataset simulations were performed with different number of species* $p \in [50, 100, 150, 200]$. *All other simulation parameters were set at their default value* $n = 100$, $\rho(\mathcal{G}) = 5\%$, $r_{neg} = 0.05, K = 100\%$. *Methods are grouped based on the implementation strategy (different columns). Note that it may happen that the displayed metric over the 50 iterations had the same value, meaning that boxplots collapse to a single line. In such cases, we plotted a coloured triangle corresponding to the median value of the metric over the 50 iterations.*

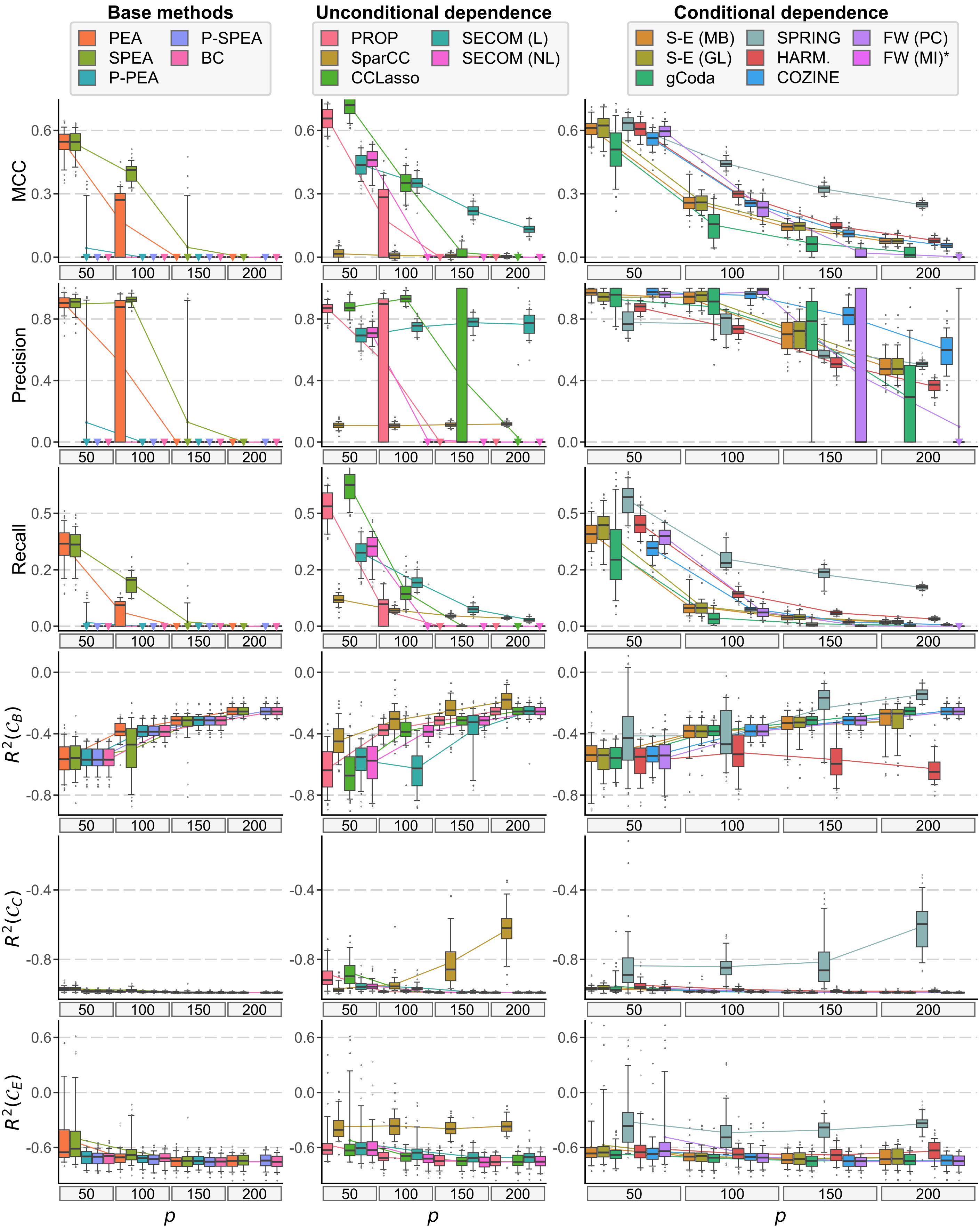

Fig W – Edge-wise and node-wise metrics for WS model with medium $\beta-$diversity for increasing number of species. *16S rDNA-seq dataset simulations were performed with different number of species* $p \in [50, 100, 150, 200]$. *All other simulation parameters were set at their default value* $n = 100$, $\rho(\mathcal{G}) = 5\%$, $r_{neg} = 0.05, K = 100\%$. *Methods are grouped based on the implementation strategy (different columns). Note that it may happen that the displayed metric over the 50 iterations had the same value, meaning that boxplots collapse to a single line. In such cases, we plotted a coloured triangle corresponding to the median value of the metric over the 50 iterations.*

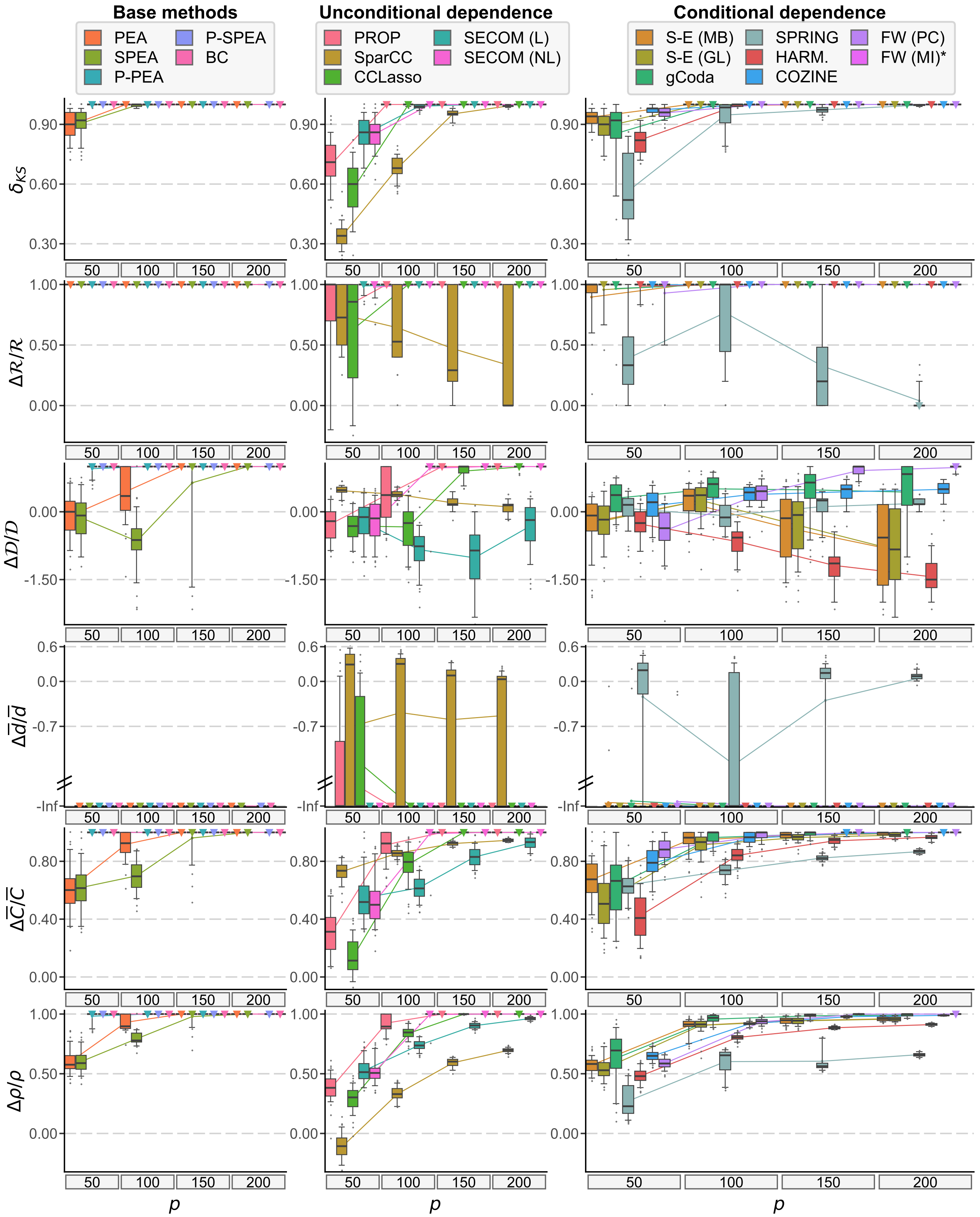

Fig X – Graph-wise metrics for WS model with medium β−diversity for increasing number of species. *16S rDNA-seq dataset simulations were performed with different number of species $p \in [50, 100, 150, 200]$. All other simulation parameters were set at their default value $n = 100$, $\rho(\mathcal{G}) = 5\%$, $r_{neg} = 0.05, K = 100\%$. Methods are grouped based on the implementation strategy (different columns). Note that it may happen that the displayed metric over the 50 iterations had the same value, meaning that boxplots collapse to a single line. In such cases, we plotted a coloured triangle corresponding to the median value of the metric over the 50 iterations.*

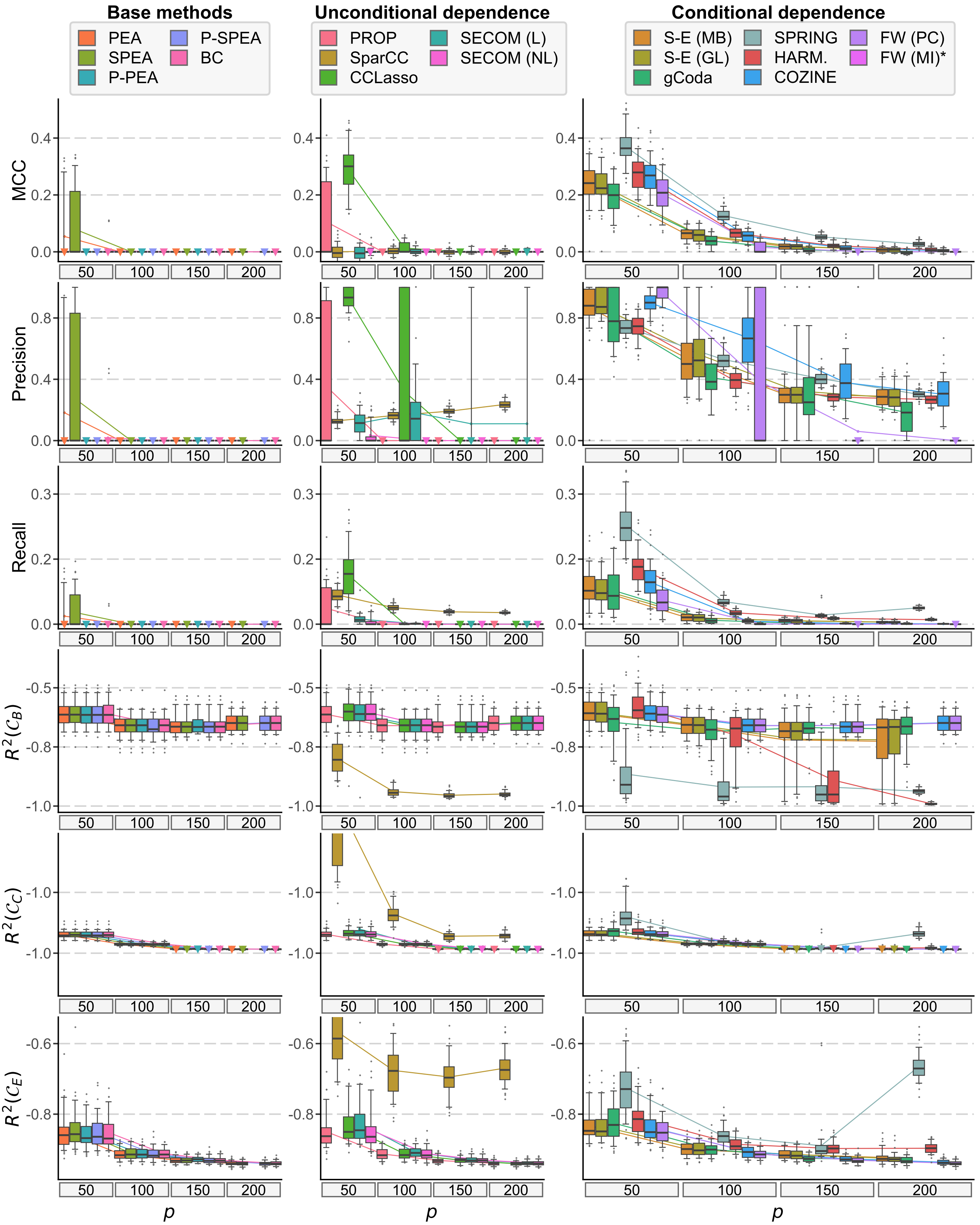

Fig Y – Edge-wise and node-wise metrics for ER model with medium $\beta-$diversity for increasing number of species. *16S rDNA-seq dataset simulations were performed with different number of species* $p \in [50, 100, 150, 200]$. *All other simulation parameters were set at their default value* $n = 100$, $\rho(\mathcal{G}) = 5\%$, $r_{neg} = 0.05, K = 100\%$. *Methods are grouped based on the implementation strategy (different columns). Note that it may happen that the displayed metric over the 50 iterations had the same value, meaning that boxplots collapse to a single line. In such cases, we plotted a coloured triangle corresponding to the median value of the metric over the 50 iterations.*

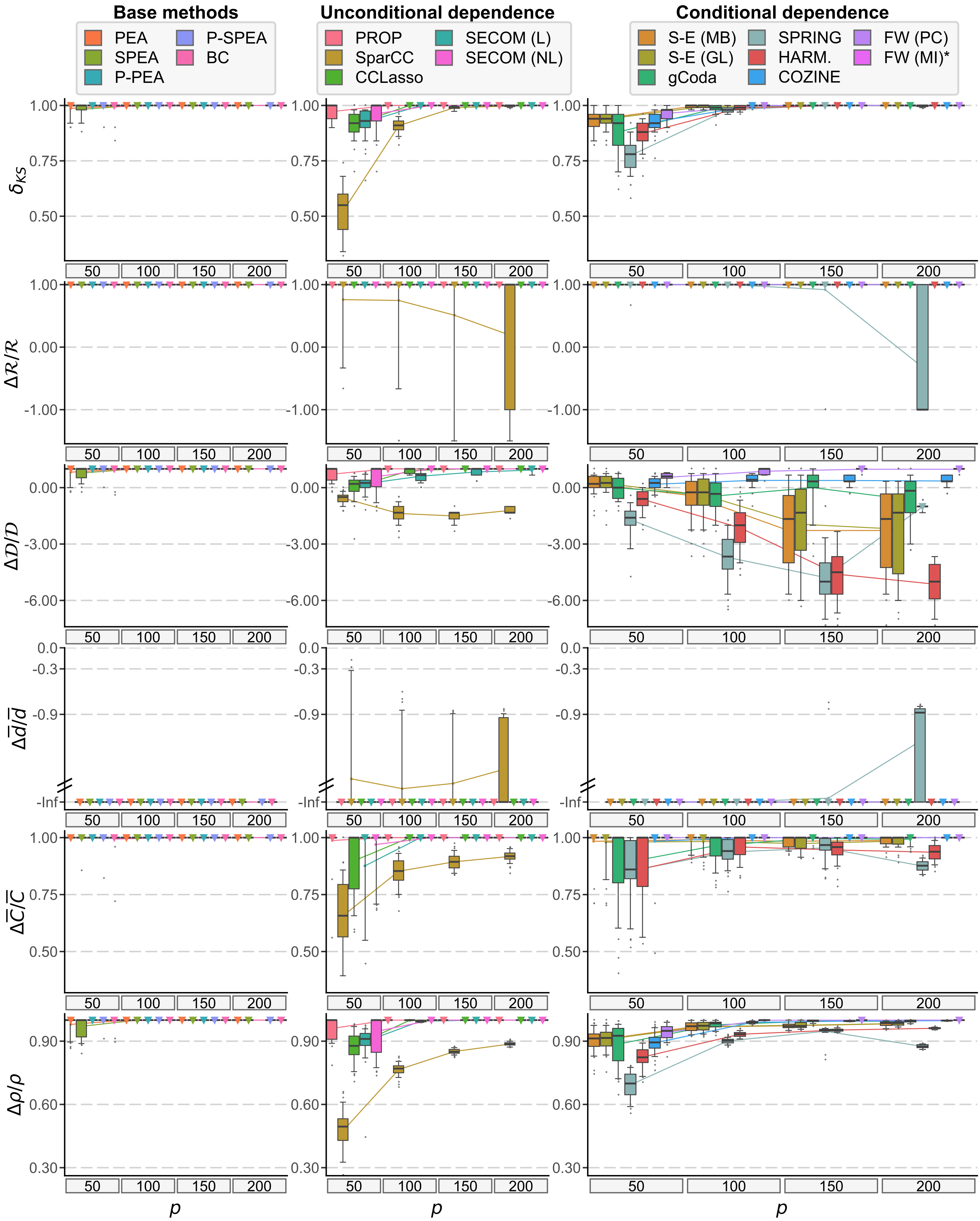

Fig Z – Graph-wise metrics for ER model with medium $\beta-$diversity for increasing number of species. *16S rDNA-seq dataset simulations were performed with different number of species $p \in [50, 100, 150, 200]$. All other simulation parameters were set at their default value $n = 100$, $\rho(\mathcal{G}) = 5\%$, $r_{neg} = 0.05, K = 100\%$. Methods are grouped based on the implementation strategy (different columns). Note that it may happen that the displayed metric over the 50 iterations had the same value, meaning that boxplots collapse to a single line. In such cases, we plotted a coloured triangle corresponding to the median value of the metric over the 50 iterations.*

5 Network density considerably affects methods' performance

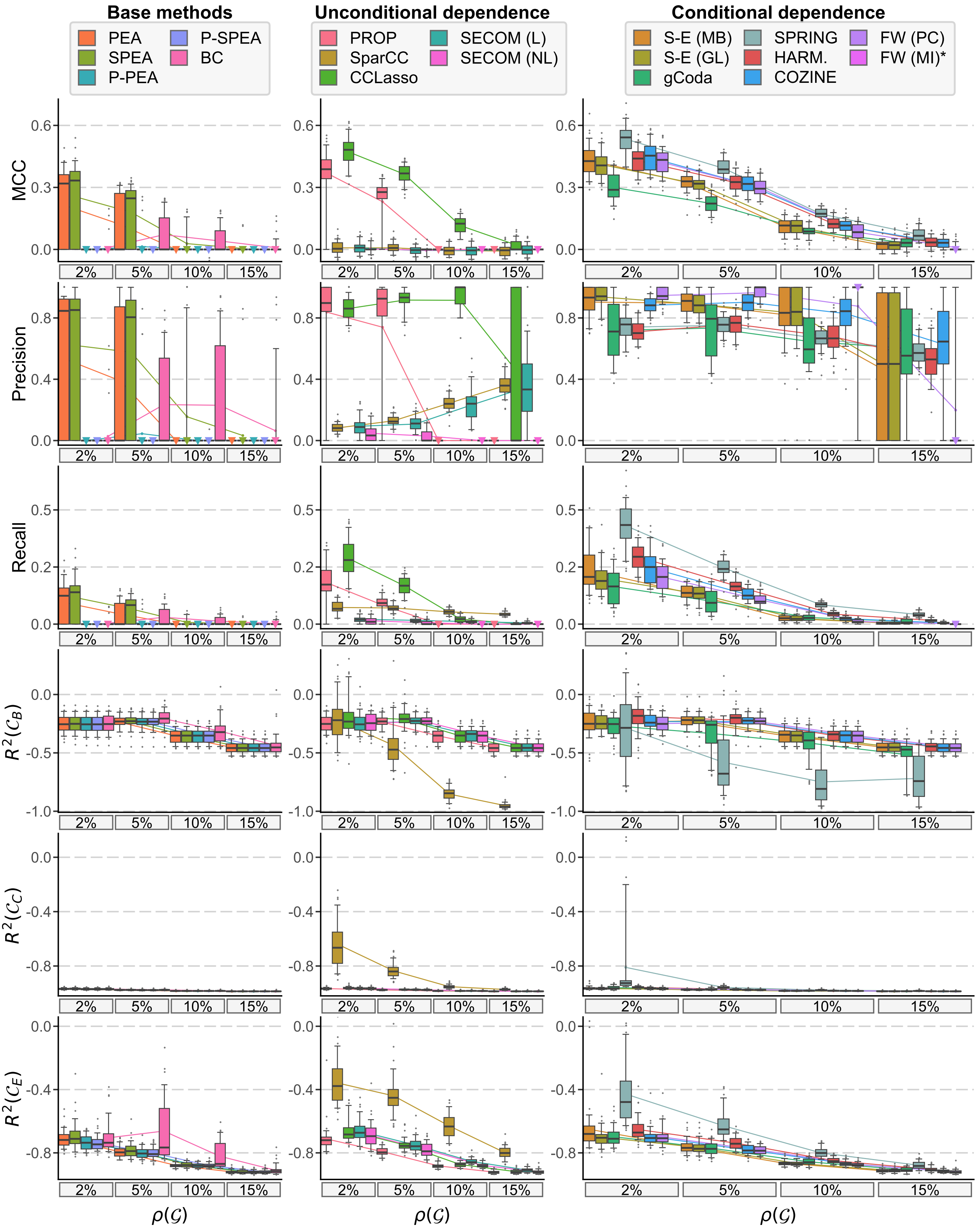

Fig AA – Edge-wise and node-wise metrics for BA model with medium $\beta-$diversity at different network densities. *16S rDNA-seq dataset simulations were performed with different network densities $\rho(\mathcal{G}) \in [2\%, 5\%, 8\%, 10\%, 15\%]$ (here we show a representative subset of the values). All other simulation parameters were set at their default value $n = 100,\ p = 50,\ r_{neg} = 0.05, K = 100\%$. Methods are grouped based on the implementation strategy (different columns). Note that it may happen that the displayed metric over the 50 iterations had the same value, meaning that boxplots collapse to a single line. In such cases, we plotted a coloured triangle corresponding to the median value of the metric over the 50 iterations.*

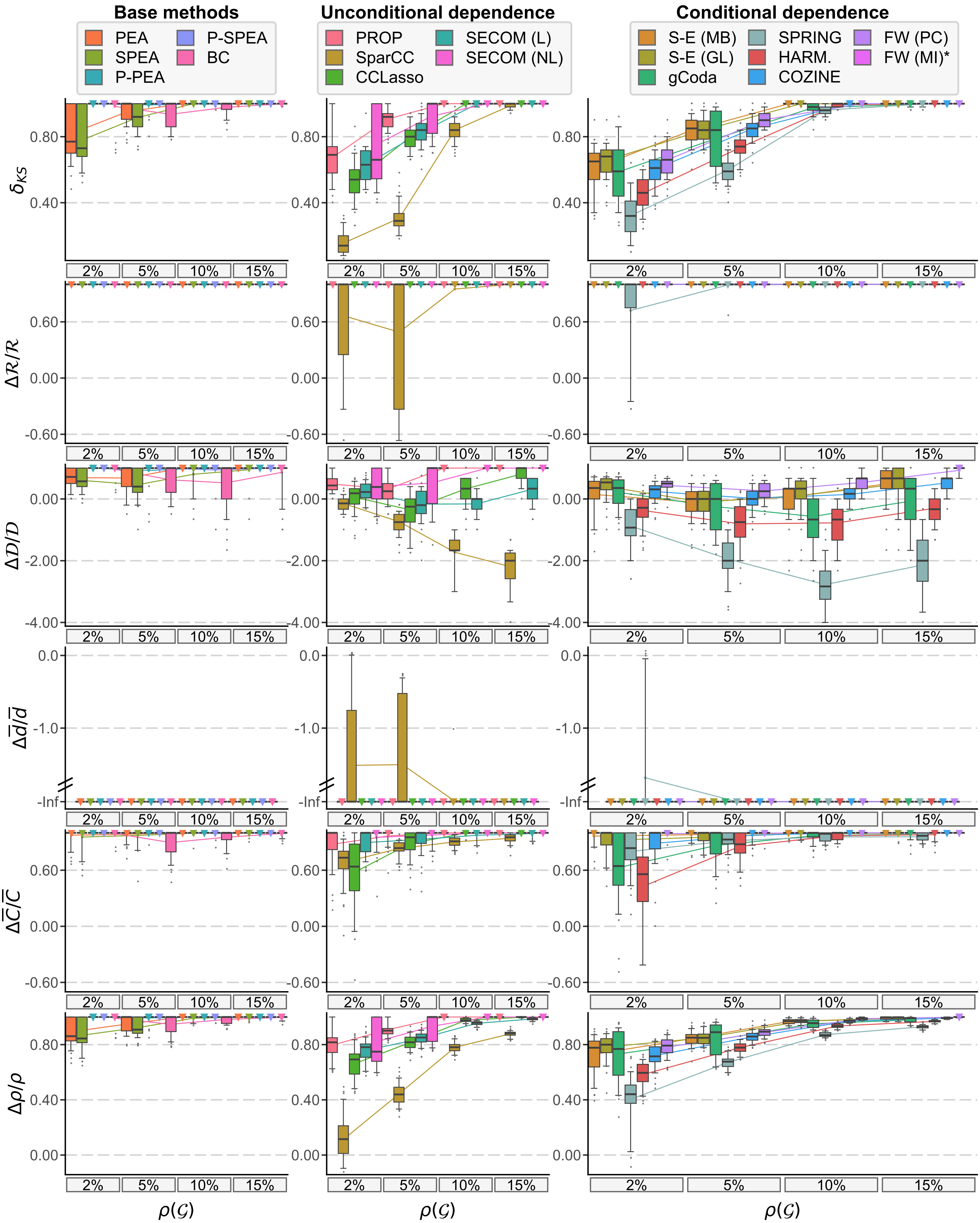

Fig AB – Graph-wise metrics for BA model with medium $\beta-$diversity at different network densities. *16S rDNA-seq dataset simulations were performed with different network densities $\rho(\mathcal{G}) \in [2\%, 5\%, 8\%, 10\%, 15\%]$ (here we show a representative subset of the values). All other simulation parameters were set at their default value $n = 100$, $p = 50$, $r_{neg} = 0.05, K = 100\%$. Methods are grouped based on the implementation strategy (different columns). Note that it may happen that the displayed metric over the 50 iterations had the same value, meaning that boxplots collapse to a single line. In such cases, we plotted a coloured triangle corresponding to the median value of the metric over the 50 iterations.*

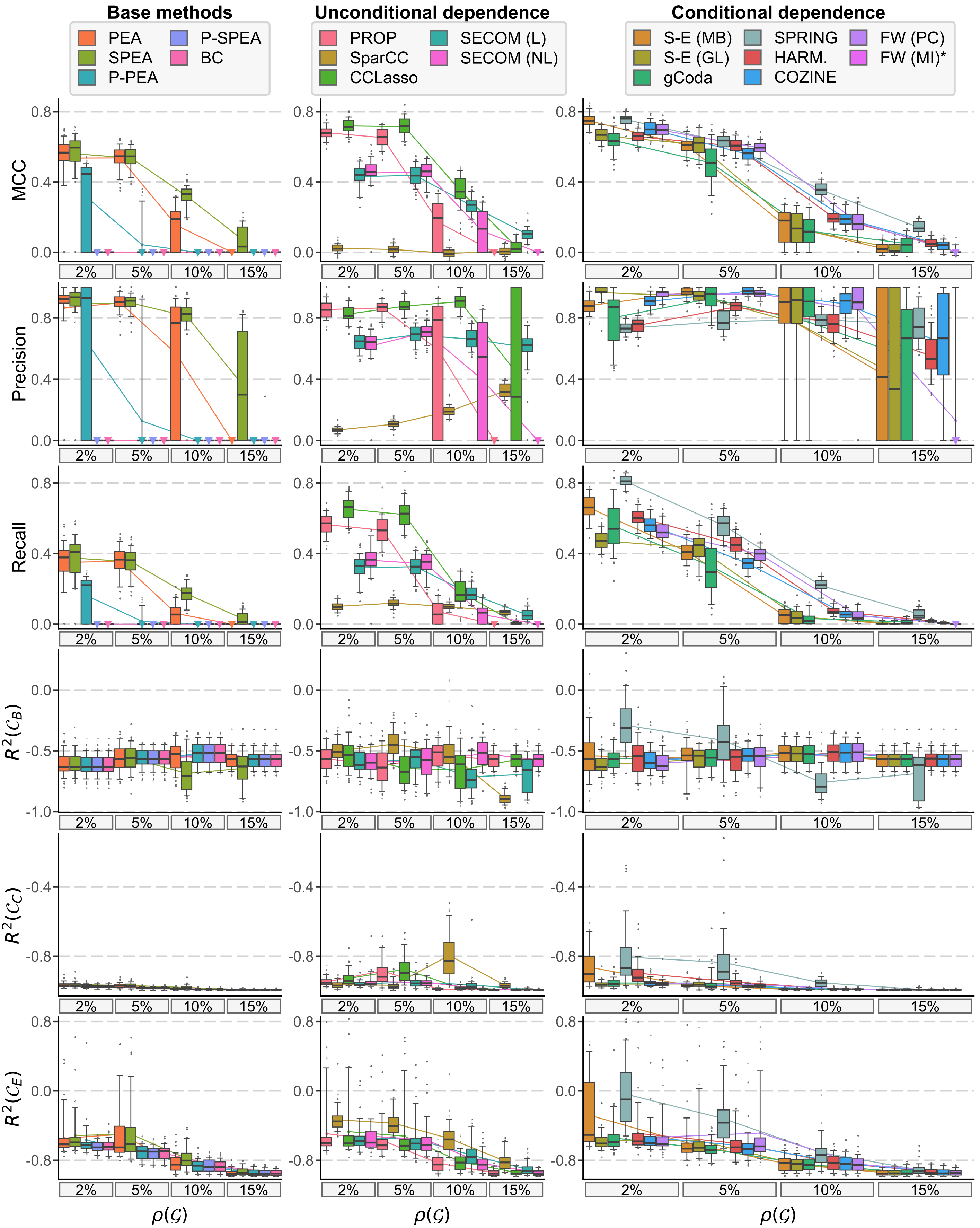

Fig AC – Edge-wise and node-wise metrics for WS model with medium $\beta-$diversity at different network densities. *16S rDNA-seq dataset simulations were performed with different network densities $\rho(\mathcal{G}) \in [2\%, 5\%, 8\%, 10\%, 15\%]$ (here we show a representative subset of the values). All other simulation parameters were set at their default value $n = 100$, $p = 50$, $r_{neg} = 0.05, K = 100\%$. Methods are grouped based on the implementation strategy (different columns). Note that it may happen that the displayed metric over the 50 iterations had the same value, meaning that boxplots collapse to a single line. In such cases, we plotted a coloured triangle corresponding to the median value of the metric over the 50 iterations.*

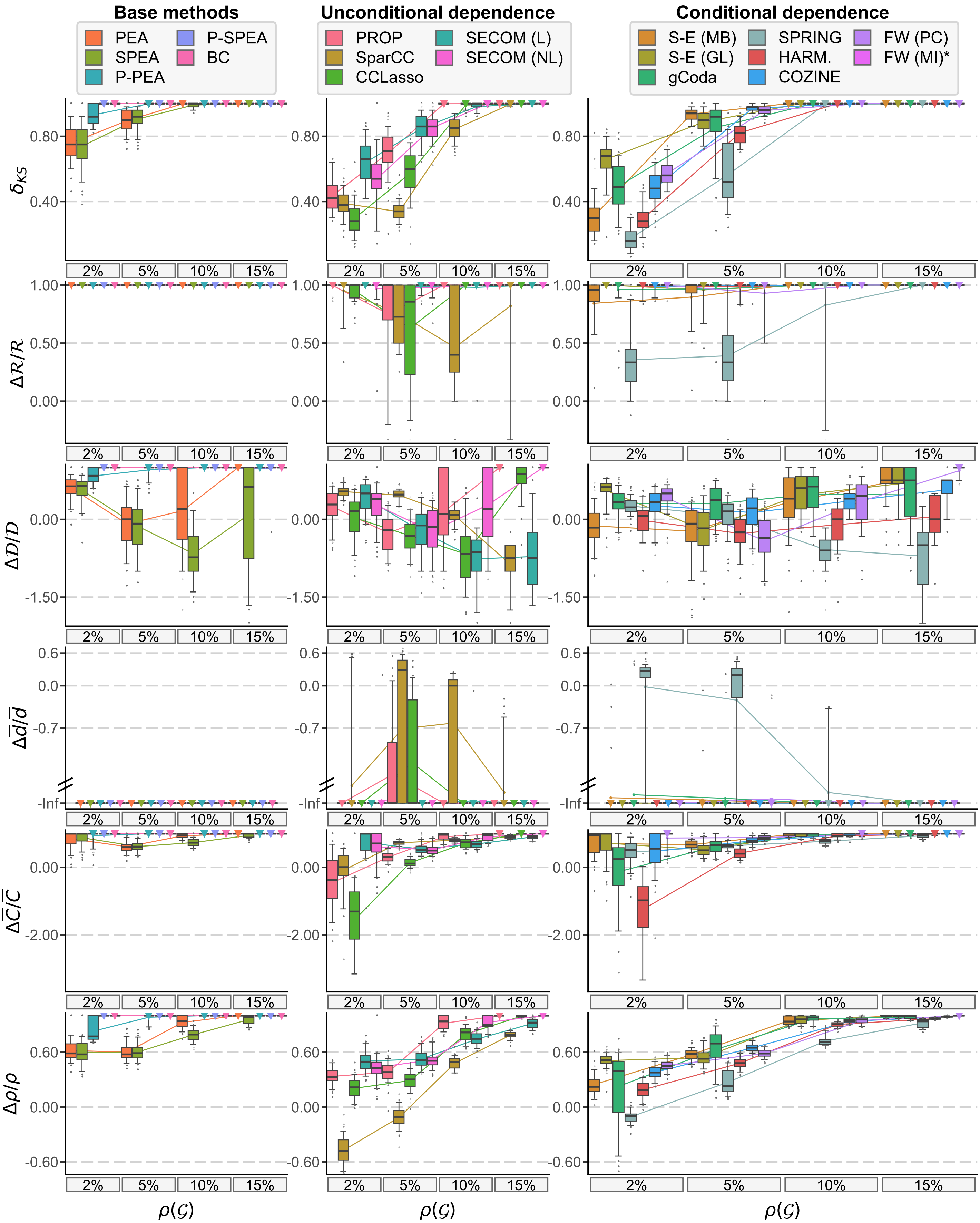

Fig AD – Graph-wise metrics for WS model with medium β−diversity at different network densities. *16S rDNA-seq dataset simulations were performed with different network densities $\rho(\mathcal{G}) \in [2\%, 5\%, 8\%, 10\%, 15\%]$ (here we show a representative subset of the values). All other simulation parameters were set at their default value $n = 100$, $p = 50$, $r_{neg} = 0.05, K = 100\%$. Methods are grouped based on the implementation strategy (different columns). Note that it may happen that the displayed metric over the 50 iterations had the same value, meaning that boxplots collapse to a single line. In such cases, we plotted a coloured triangle corresponding to the median value of the metric over the 50 iterations.*

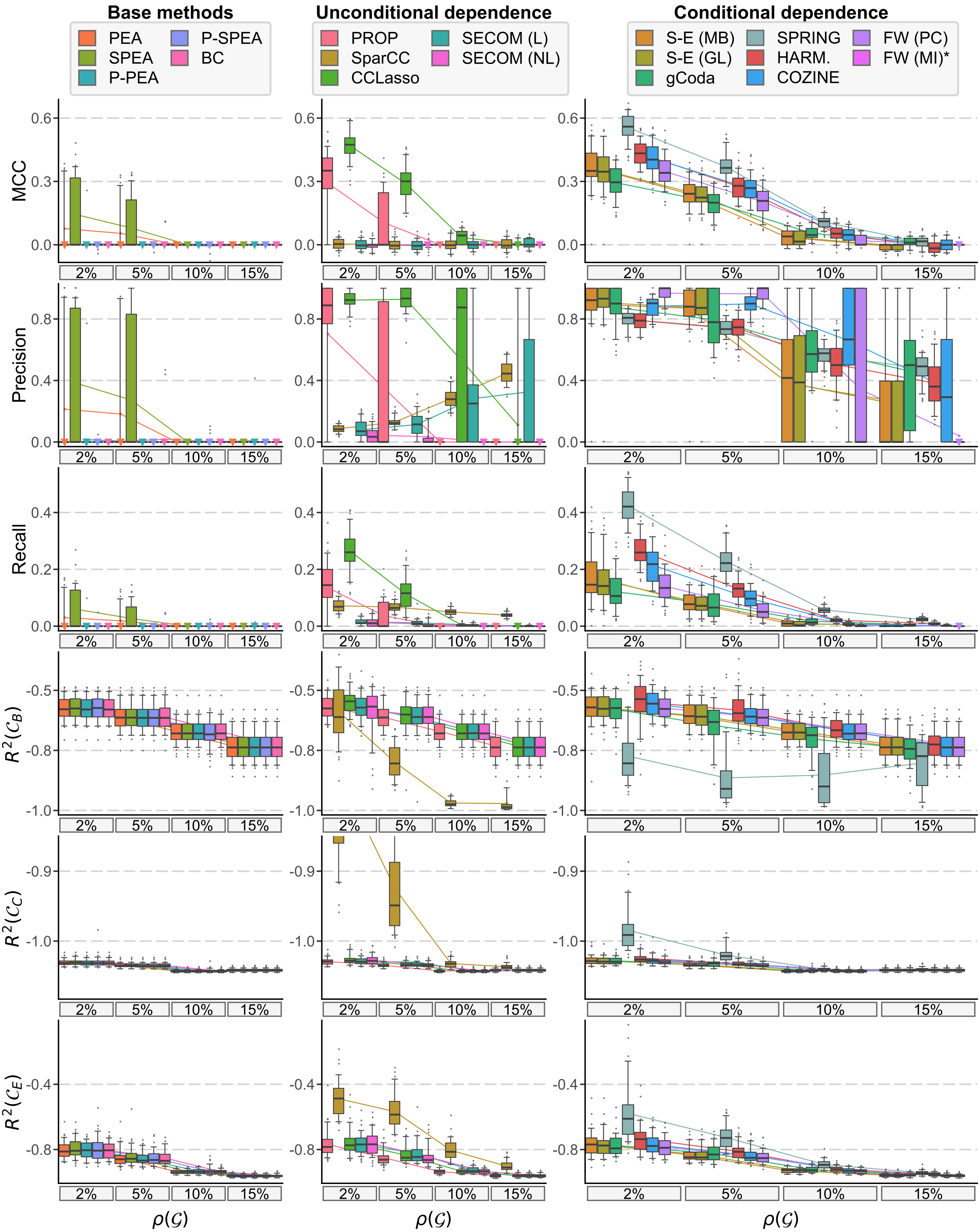

Fig AE – Edge-wise and node-wise metrics for ER model with medium $\beta-$diversity at different network densities. *16S rDNA-seq dataset simulations were performed with different network densities $\rho(\mathcal{G}) \in [2\%, 5\%, 8\%, 10\%, 15\%]$ (here we show a representative subset of the values). All other simulation parameters were set at their default value $n = 100$, $p = 50$, $r_{neg} = 0.05, K = 100\%$. Methods are grouped based on the implementation strategy (different columns). Note that it may happen that the displayed metric over the 50 iterations had the same value, meaning that boxplots collapse to a single line. In such cases, we plotted a coloured triangle corresponding to the median value of the metric over the 50 iterations.*

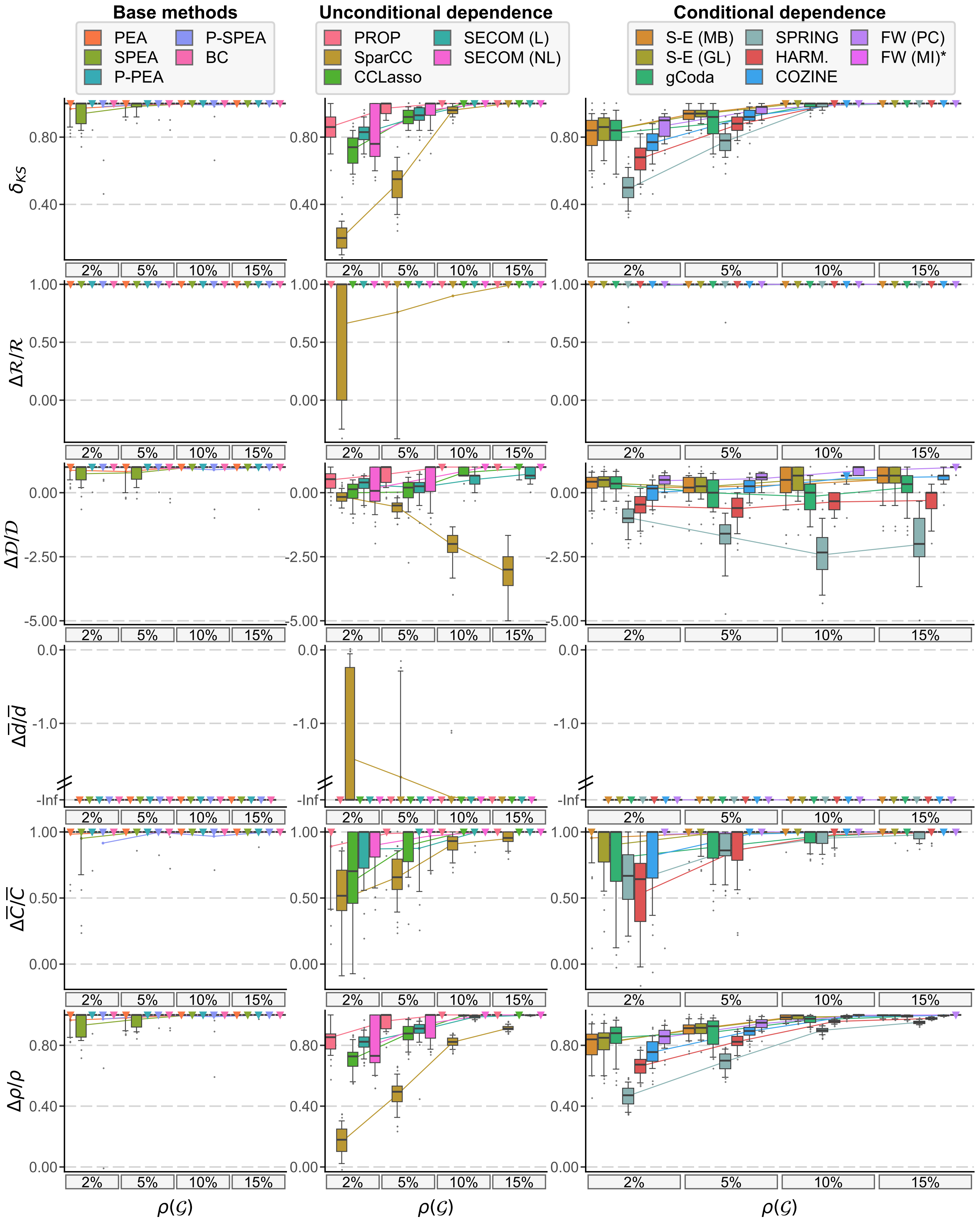

Fig AF – Graph-wise metrics for ER model with medium $\beta-$diversity at different network densities. *16S rDNA-seq dataset simulations were performed with different network densities $\rho(\mathcal{G}) \in [2\%, 5\%, 8\%, 10\%, 15\%]$ (here we show a representative subset of the values). All other simulation parameters were set at their default value $n = 100$, $p = 50$, $r_{neg} = 0.05, K = 100\%$. Methods are grouped based on the implementation strategy (different columns). Note that it may happen that the displayed metric over the 50 iterations had the same value, meaning that boxplots collapse to a single line. In such cases, we plotted a coloured triangle corresponding to the median value of the metric over the 50 iterations.*

6 Negative interactions increase the difficulty of network inference

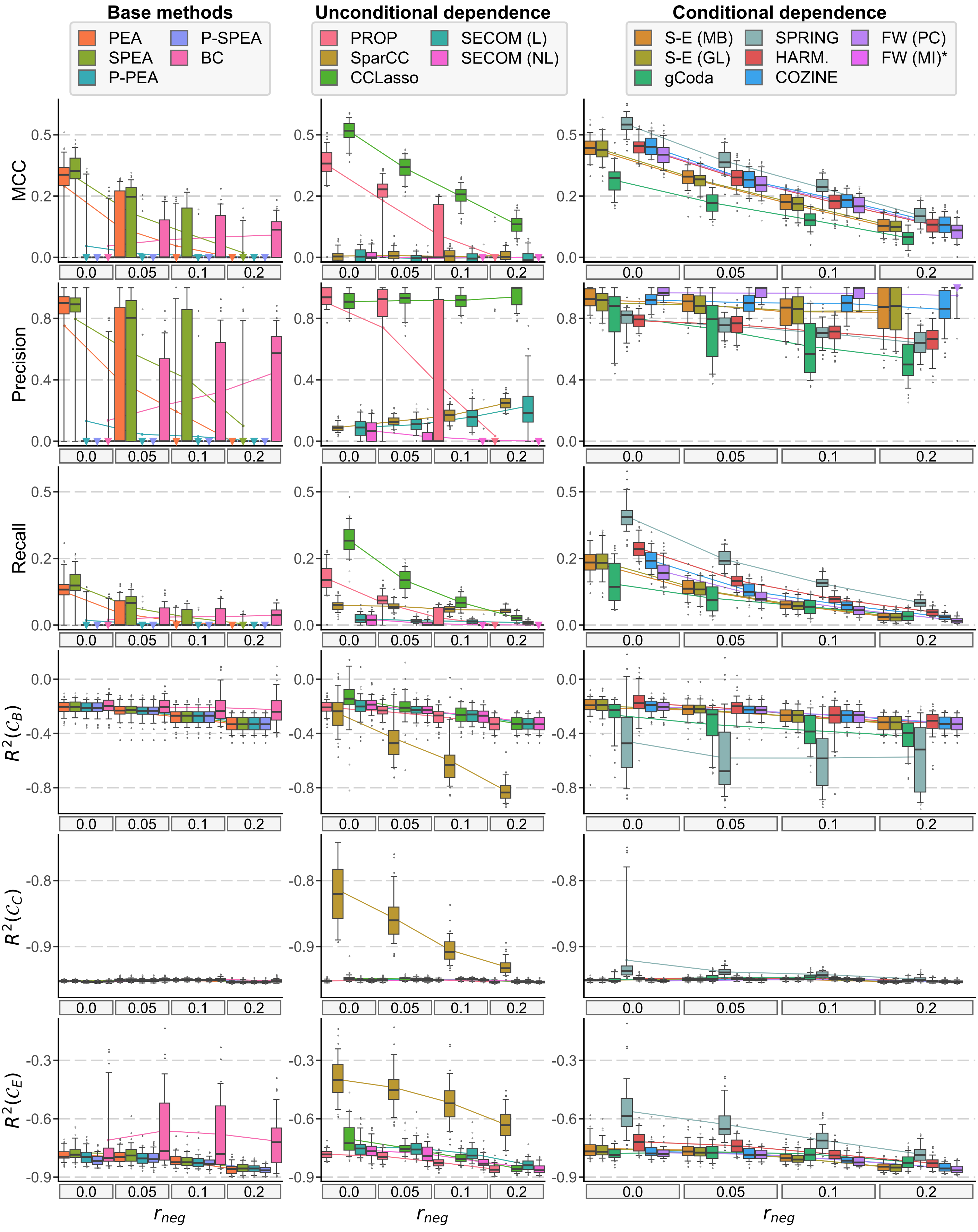

Fig AG – Edge-wise and node-wise metrics for BA model with medium β−diversity for increasing negative interaction ratio. *16S rDNA-seq dataset simulations were performed with different levels of negative interaction ratio* $r_{neg} \in [0, 0.05, 0.1, 0.2]$. *All other simulation parameters were set at their default value* $n = 100,\ \rho(\mathcal{G}) = 5\%,\ K = 100\%, p = 50$. *Methods are grouped based on the implementation strategy (different columns). Note that it may happen that the displayed metric over the 50 iterations had the same value, meaning that boxplots collapse to a single line. In such cases, we plotted a coloured triangle corresponding to the median value of the metric over the 50 iterations.*

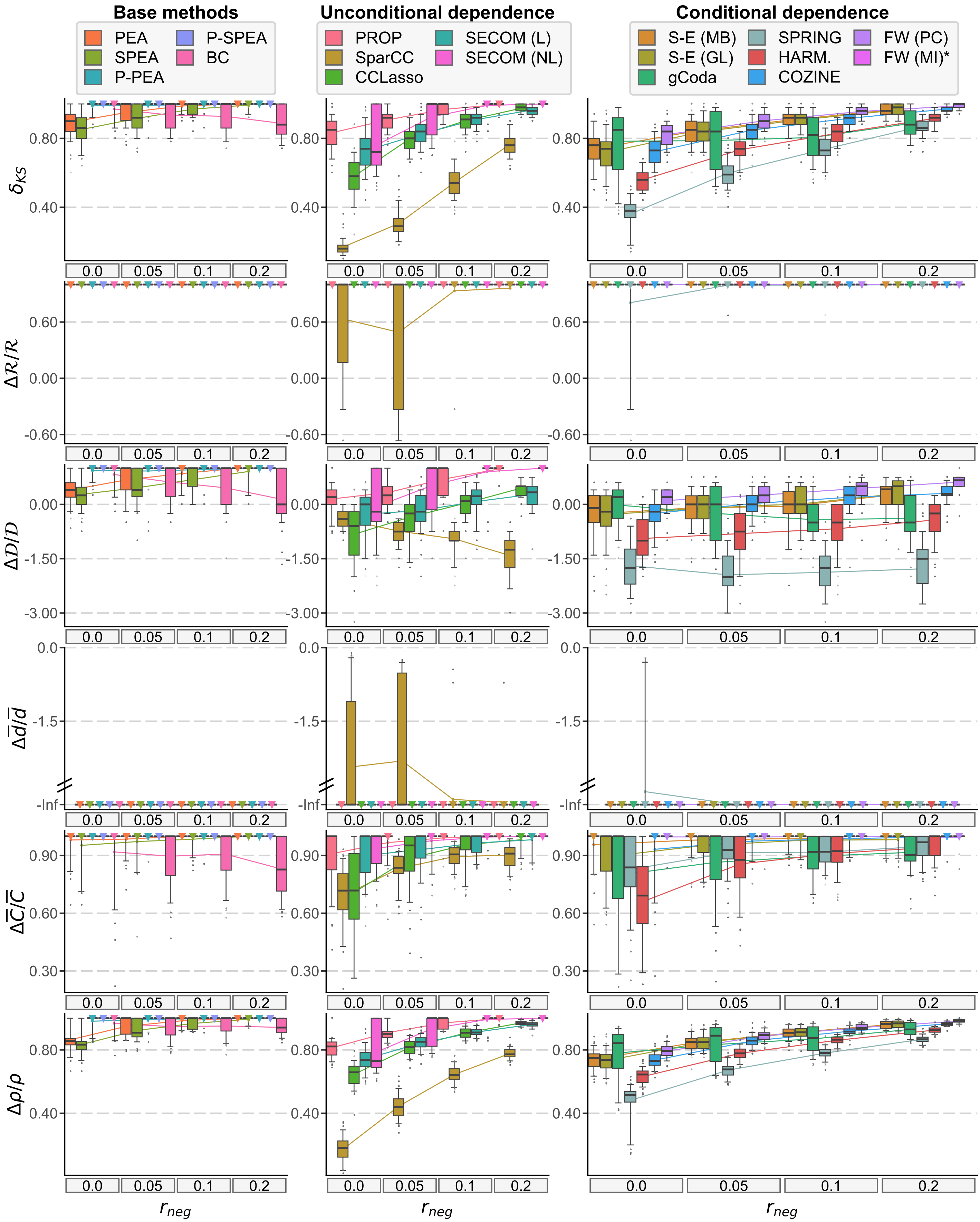

Fig AH – Graph-wise metrics for BA model with medium β–diversity for increasing negative interaction ratio. *16S rDNA-seq dataset simulations were performed with different levels of negative interaction ratio $r_{neg} \in [0, 0.05, 0.1, 0.2]$. All other simulation parameters were set at their default value $n = 100$, $\rho(\mathcal{G}) = 5\%$, $K = 100\%, p = 50$. Methods are grouped based on the implementation strategy (different columns). Note that it may happen that the displayed metric over the 50 iterations had the same value, meaning that boxplots collapse to a single line. In such cases, we plotted a coloured triangle corresponding to the median value of the metric over the 50 iterations.*

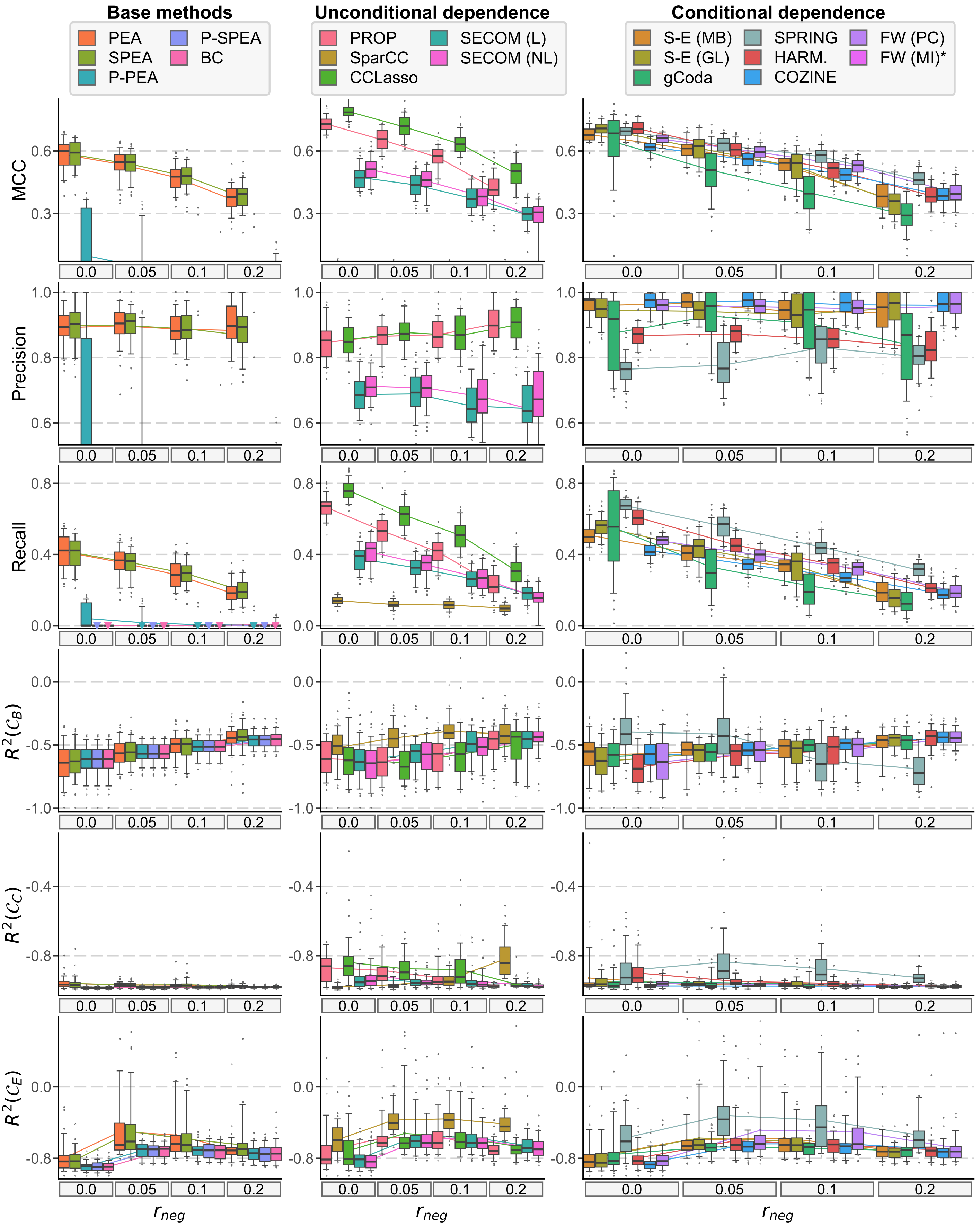

Fig AI – Edge-wise and node-wise metrics for WS model with medium β−diversity for increasing negative interaction ratio. *16S rDNA-seq dataset simulations were performed with different levels of negative interaction ratio* $r_{neg} \in [0, 0.05, 0.1, 0.2]$. *All other simulation parameters were set at their default value* $n = 100$, $\rho(\mathcal{G}) = 5\%$, $K = 100\%, p = 50$. *Methods are grouped based on the implementation strategy (different columns). Note that it may happen that the displayed metric over the 50 iterations had the same value, meaning that boxplots collapse to a single line. In such cases, we plotted a coloured triangle corresponding to the median value of the metric over the 50 iterations.*

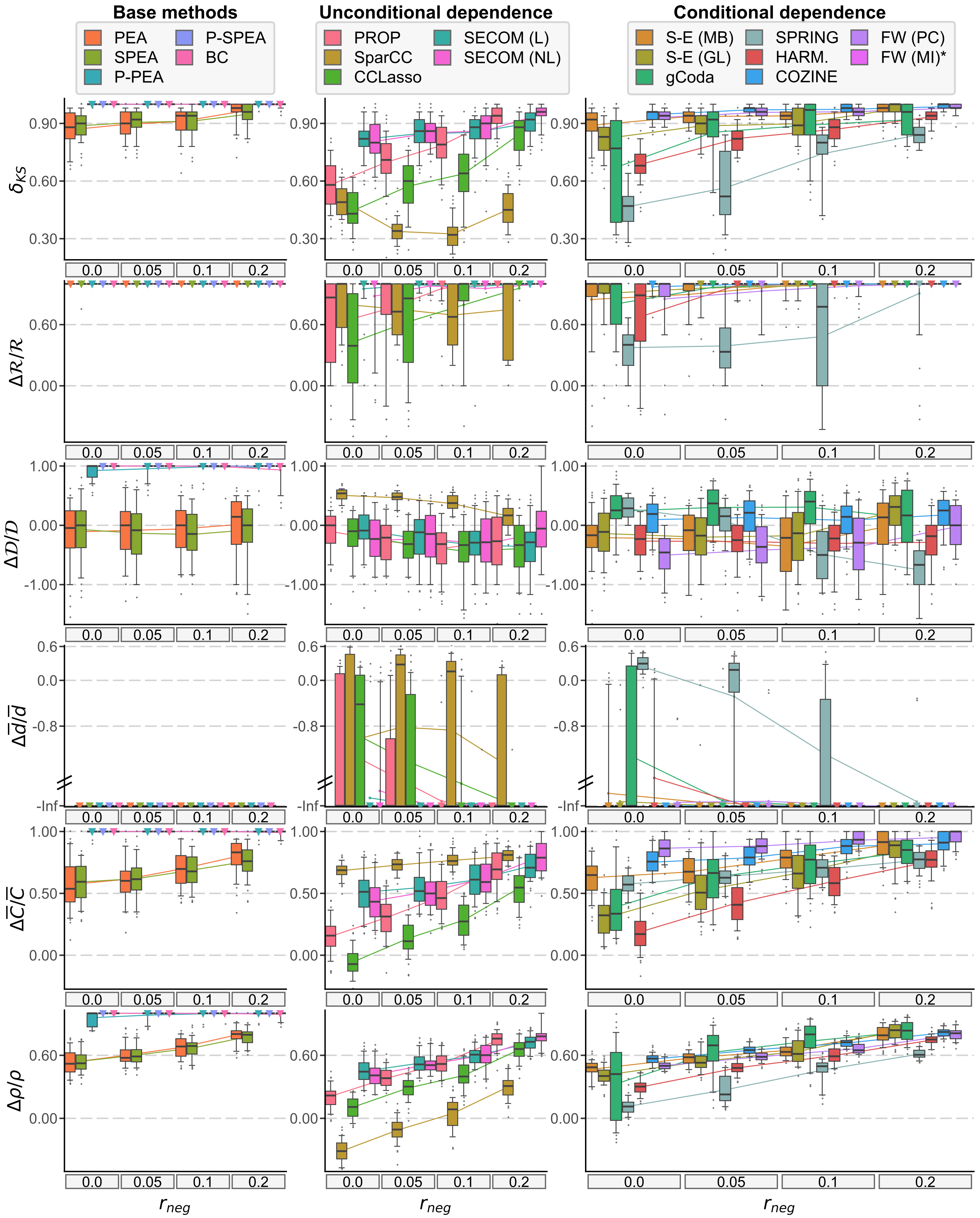

Fig AJ – Graph-wise metrics for WS model with medium β−diversity for increasing negative interaction ratio. *16S rDNA-seq dataset simulations were performed with different levels of negative interaction ratio* $r_{neg} \in [0, 0.05, 0.1, 0.2]$. *All other simulation parameters were set at their default value* $n = 100$, $\rho(\mathcal{G}) = 5\%$, $K = 100\%, p = 50$. *Methods are grouped based on the implementation strategy (different columns). Note that it may happen that the displayed metric over the 50 iterations had the same value, meaning that boxplots collapse to a single line. In such cases, we plotted a coloured triangle corresponding to the median value of the metric over the 50 iterations.*

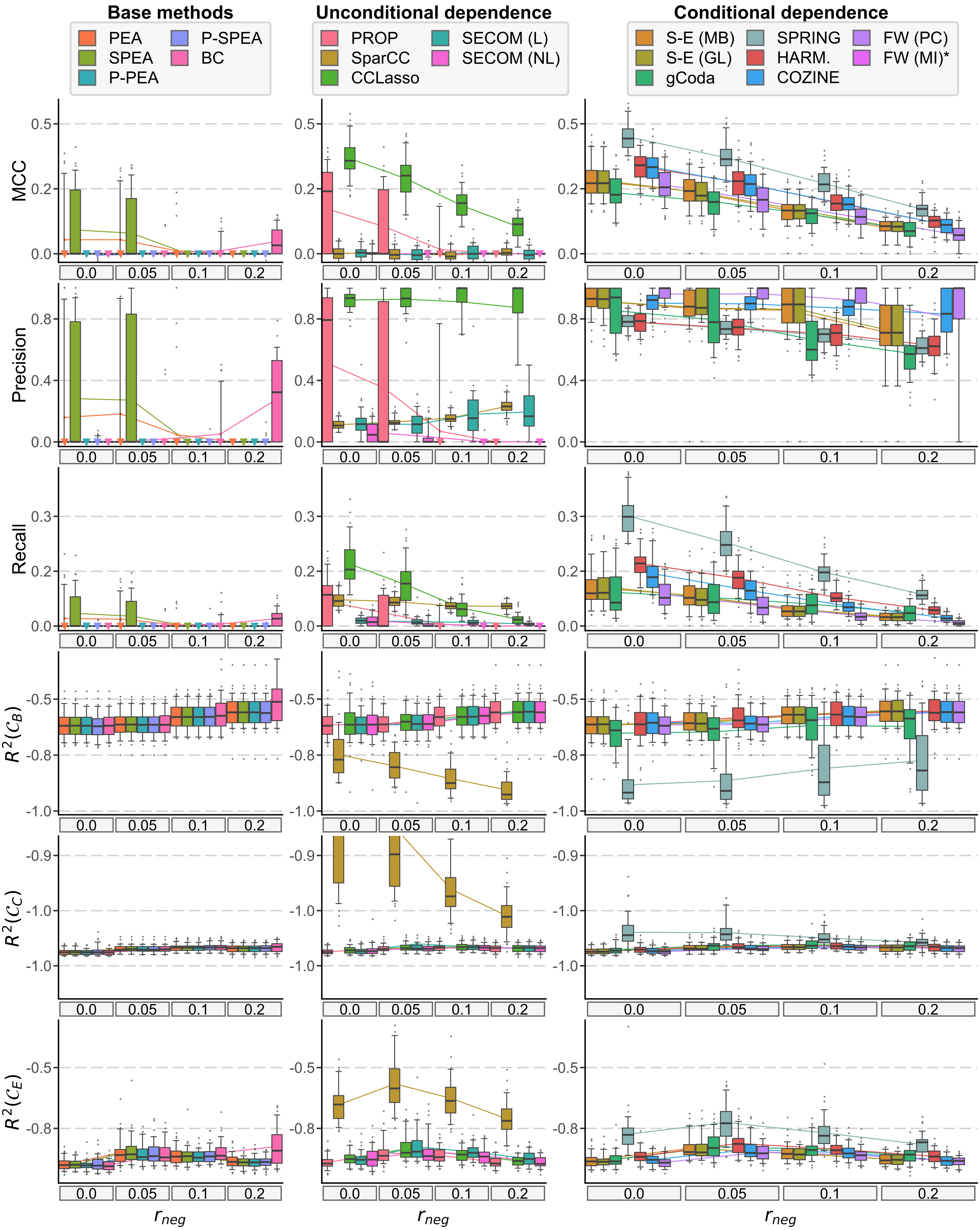

Fig AK – Edge-wise and node-wise metrics for ER model with medium $\beta-$diversity for increasing negative interaction ratio. *16S rDNA-seq dataset simulations were performed with different levels of negative interaction ratio $r_{neg} \in [0, 0.05, 0.1, 0.2]$. All other simulation parameters were set at their default value $n = 100$, $\rho(\mathcal{G}) = 5\%$, $K = 100\%, p = 50$. Methods are grouped based on the implementation strategy (different columns). Note that it may happen that the displayed metric over the 50 iterations had the same value, meaning that boxplots collapse to a single line. In such cases, we plotted a coloured triangle corresponding to the median value of the metric over the 50 iterations.*

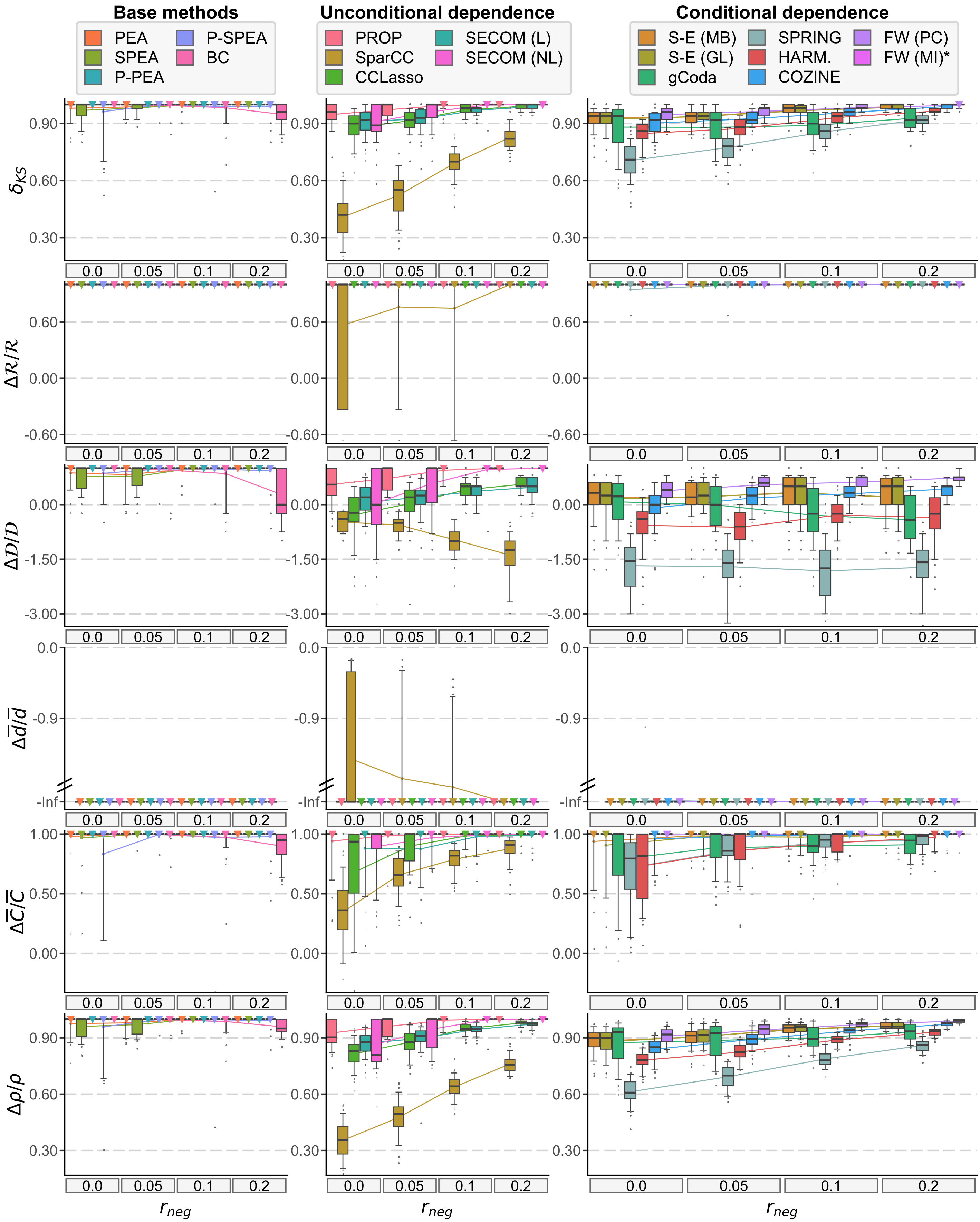

Fig AL – Graph-wise metrics for ER model with medium β−diversity for increasing negative interaction ratio. *16S rDNA-seq dataset simulations were performed with different levels of negative interaction ratio $r_{neg} \in [0, 0.05, 0.1, 0.2]$. All other simulation parameters were set at their default value $n = 100,\ \rho(\mathcal{G}) = 5\%,\ K = 100\%, p = 50$. Methods are grouped based on the implementation strategy (different columns). Note that it may happen that the displayed metric over the 50 iterations had the same value, meaning that boxplots collapse to a single line. In such cases, we plotted a coloured triangle corresponding to the median value of the metric over the 50 iterations.*

7 Reduced library size degrades network inference performance

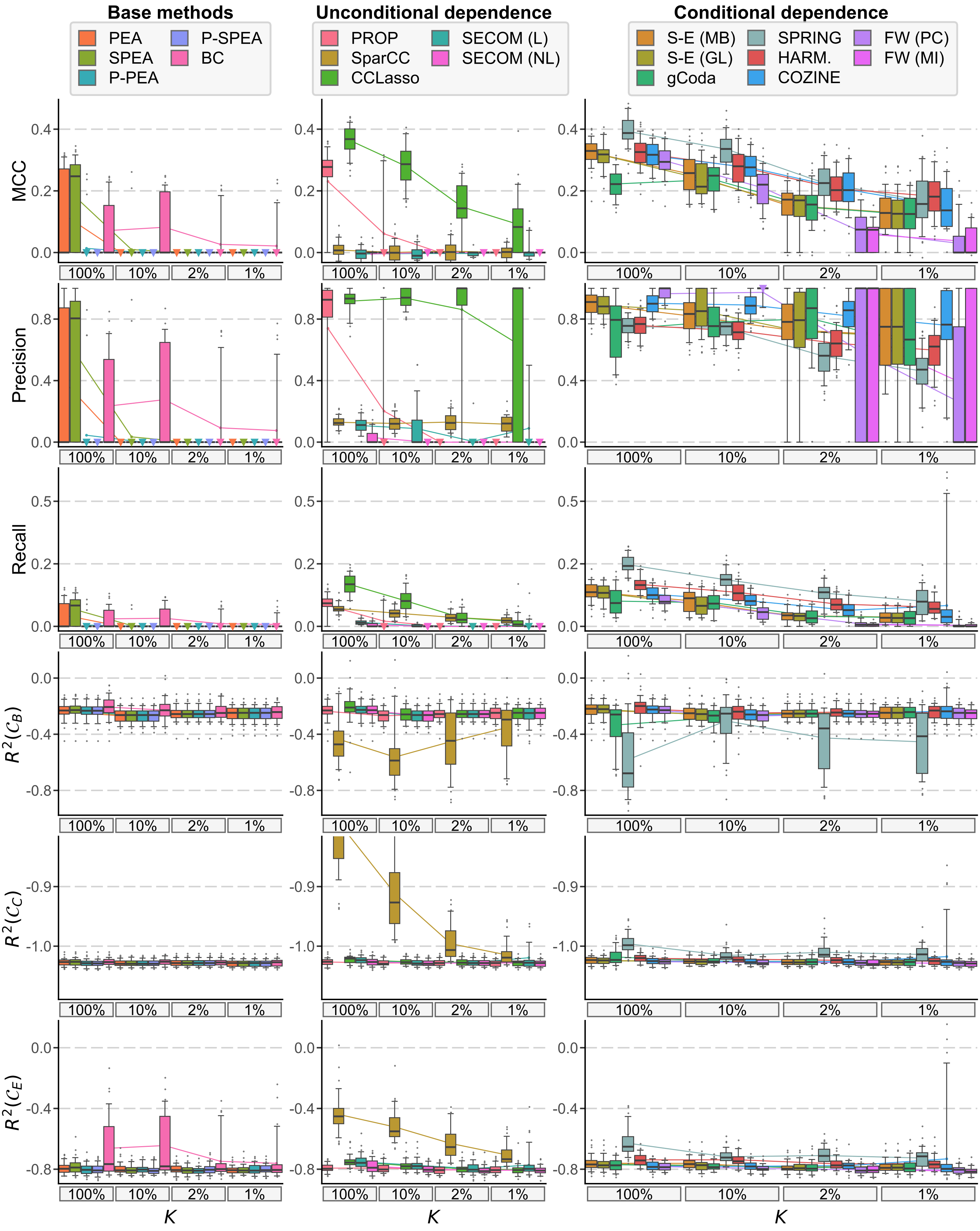

Fig AM – Edge-wise and node-wise metrics for BA model with medium $\beta-$diversity for decreasing number of extractions. *16S rDNA-seq dataset simulations were performed with different library size fraction $K \in [100\%, 10\%, 2\%, 1\%, 0.5\%]$ (here we show a representative subset of the values). All other simulation parameters were set at their default value $n = 100,\ \rho(\mathcal{G}) = 5\%,\ r_{neg} = 0.05, p = 50$. Methods are grouped based on the implementation strategy (different columns). Note that it may happen that the displayed metric over the 50 iterations had the same value, meaning that boxplots collapse to a single line. In such cases, we plotted a coloured triangle corresponding to the median value of the metric over the 50 iterations.*

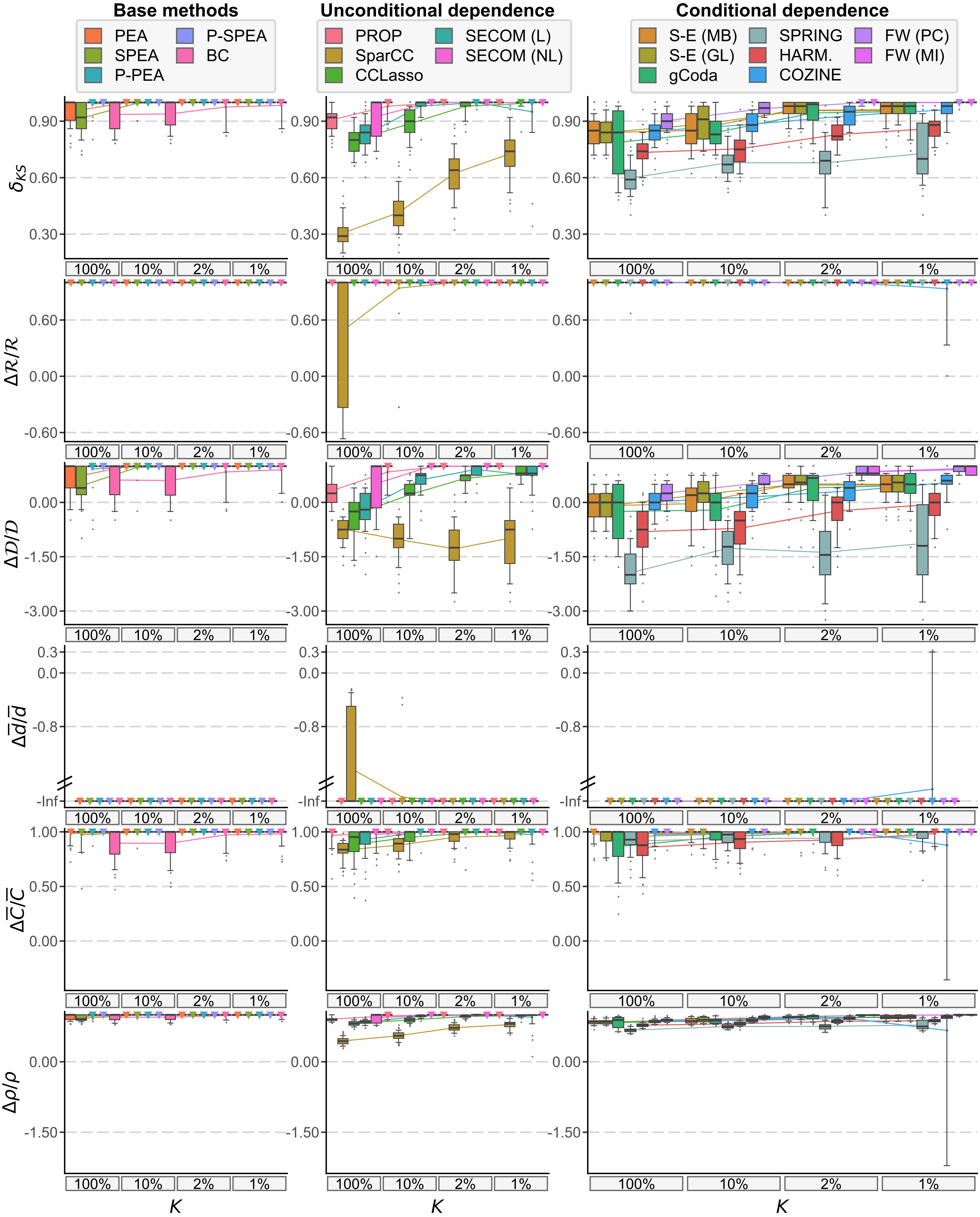

Fig AN – Graph-wise metrics for BA model with medium $\beta-$diversity for decreasing number of extractions. *16S rDNA-seq dataset simulations were performed with different library size fraction* $K \in [100\%, 10\%, 2\%, 1\%, 0.5\%]$ *(here we show a representative subset of the values). All other simulation parameters were set at their default value* $n = 100$, $\rho(\mathcal{G}) = 5\%$, $r_{neg} = 0.05, p = 50$. *Methods are grouped based on the implementation strategy (different columns). Note that it may happen that the displayed metric over the 50 iterations had the same value, meaning that boxplots collapse to a single line. In such cases, we plotted a coloured triangle corresponding to the median value of the metric over the 50 iterations.*

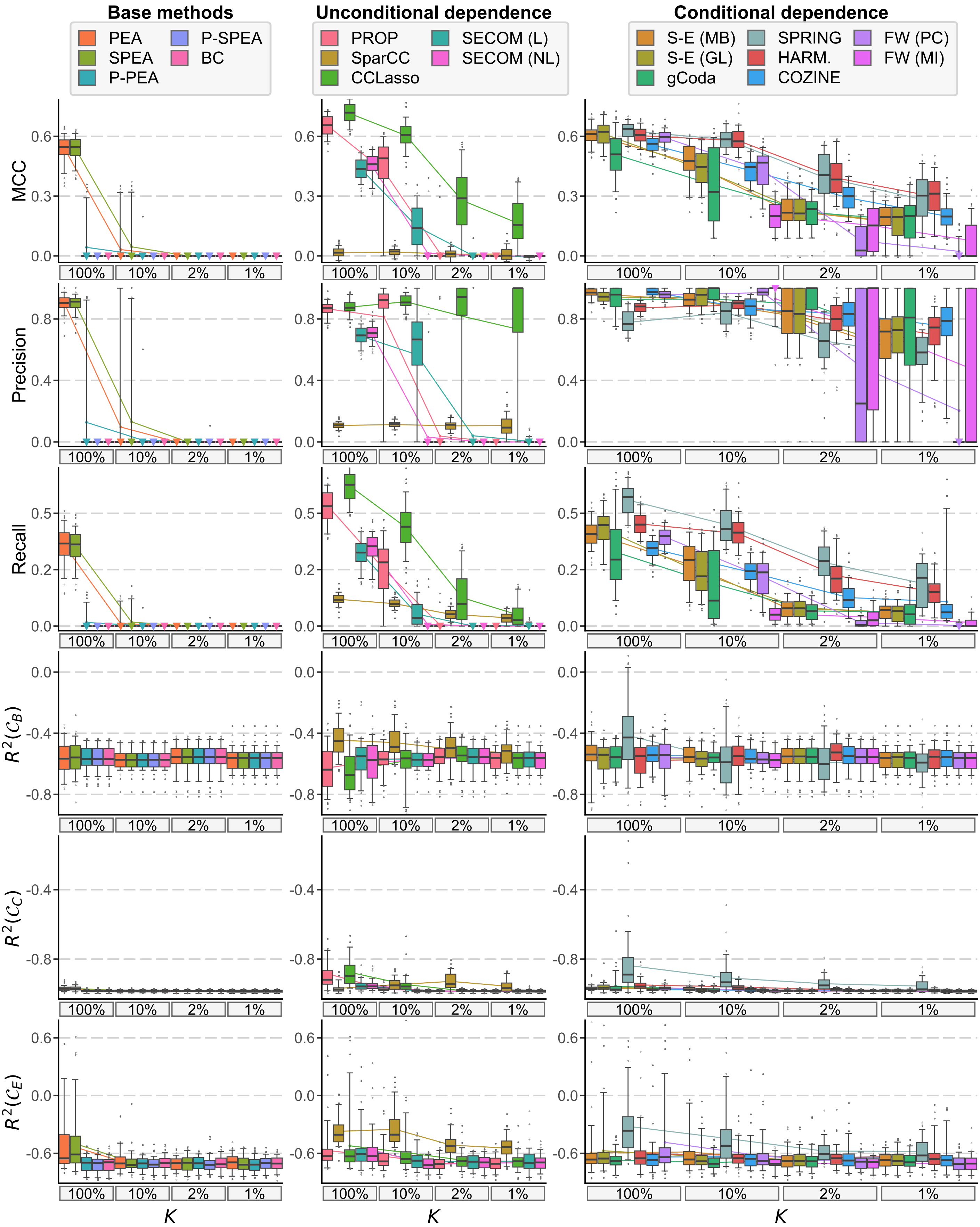

Fig AO – Edge-wise and node-wise metrics for WS model with medium $\beta-$diversity for decreasing number of extractions. *16S rDNA-seq dataset simulations were performed with different library size fraction $K \in [100\%, 10\%, 2\%, 1\%, 0.5\%]$ (here we show a representative subset of the values). All other simulation parameters were set at their default value $n = 100$, $\rho(\mathcal{G}) = 5\%$, $r_{neg} = 0.05, p = 50$. Methods are grouped based on the implementation strategy (different columns). Note that it may happen that the displayed metric over the 50 iterations had the same value, meaning that boxplots collapse to a single line. In such cases, we plotted a coloured triangle corresponding to the median value of the metric over the 50 iterations.*

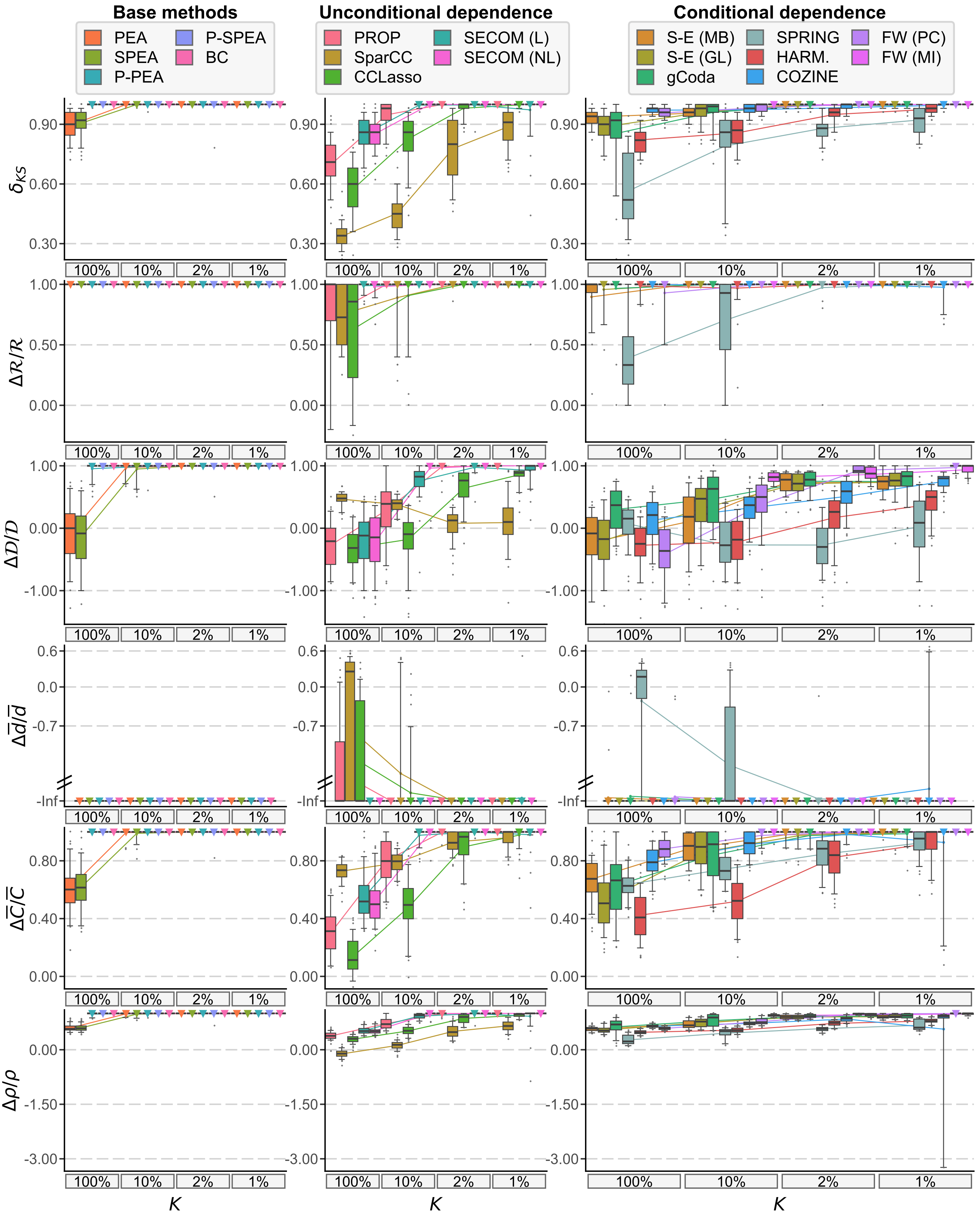

Fig AP – Graph-wise metrics for WS model with medium $\beta-$diversity for decreasing number of extractions. *16S rDNA-seq dataset simulations were performed with different library size fraction $K \in [100\%, 10\%, 2\%, 1\%, 0.5\%]$ (here we show a representative subset of the values). All other simulation parameters were set at their default value $n = 100$, $\rho(\mathcal{G}) = 5\%$, $r_{neg} = 0.05, p = 50$. Methods are grouped based on the implementation strategy (different columns). Note that it may happen that the displayed metric over the 50 iterations had the same value, meaning that boxplots collapse to a single line. In such cases, we plotted a coloured triangle corresponding to the median value of the metric over the 50 iterations.*

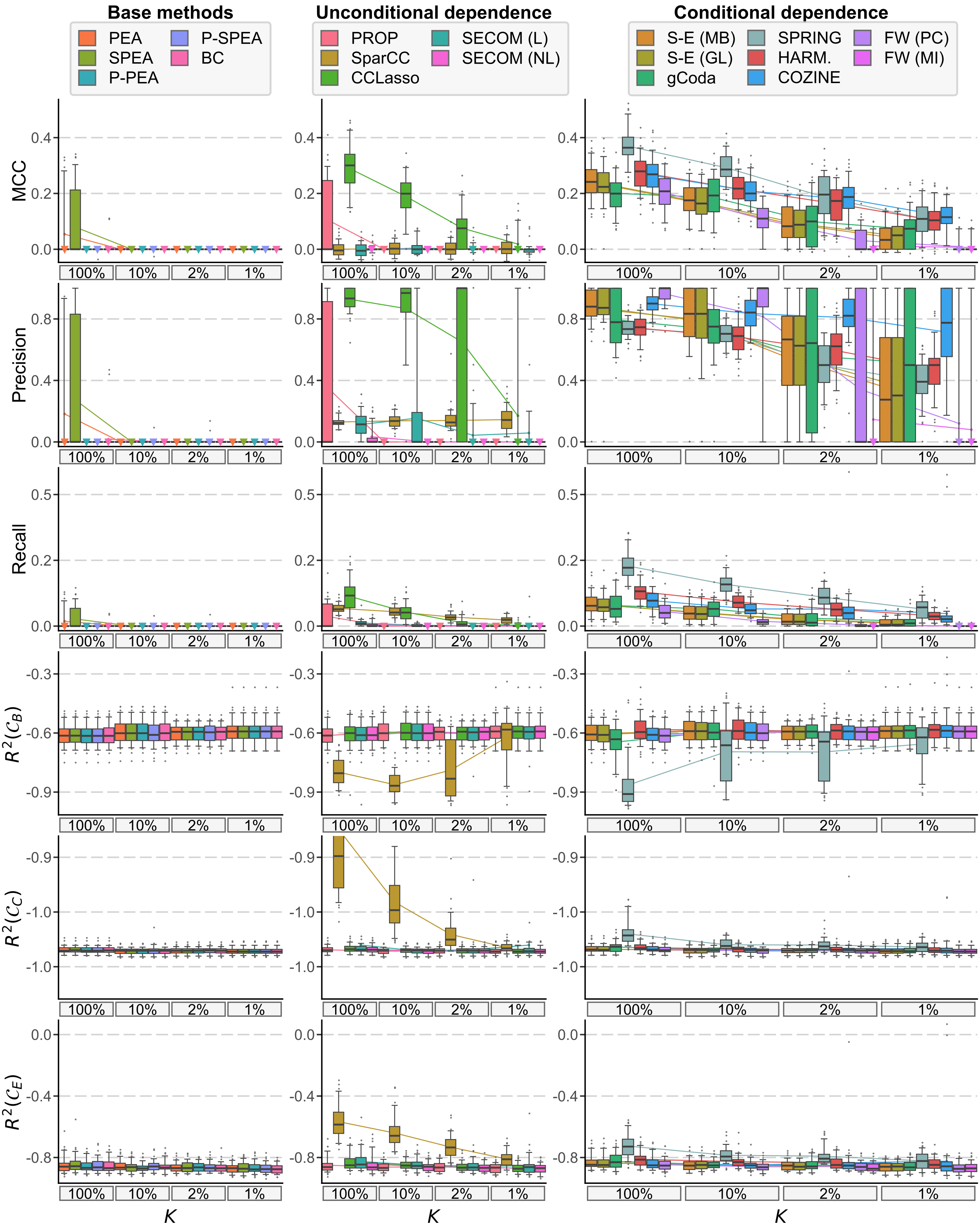

Fig AQ – Edge-wise and node-wise metrics for ER model with medium $\beta-$diversity for decreasing number of extractions. *16S rDNA-seq dataset simulations were performed with different library size fraction* $K \in [100\%, 10\%, 2\%, 1\%, 0.5\%]$ *(here we show a representative subset of the values). All other simulation parameters were set at their default value* $n = 100,\ \rho(\mathcal{G}) = 5\%,\ r_{neg} = 0.05, p = 50$. *Methods are grouped based on the implementation strategy (different columns). Note that it may happen that the displayed metric over the 50 iterations had the same value, meaning that boxplots collapse to a single line. In such cases, we plotted a coloured triangle corresponding to the median value of the metric over the 50 iterations.*

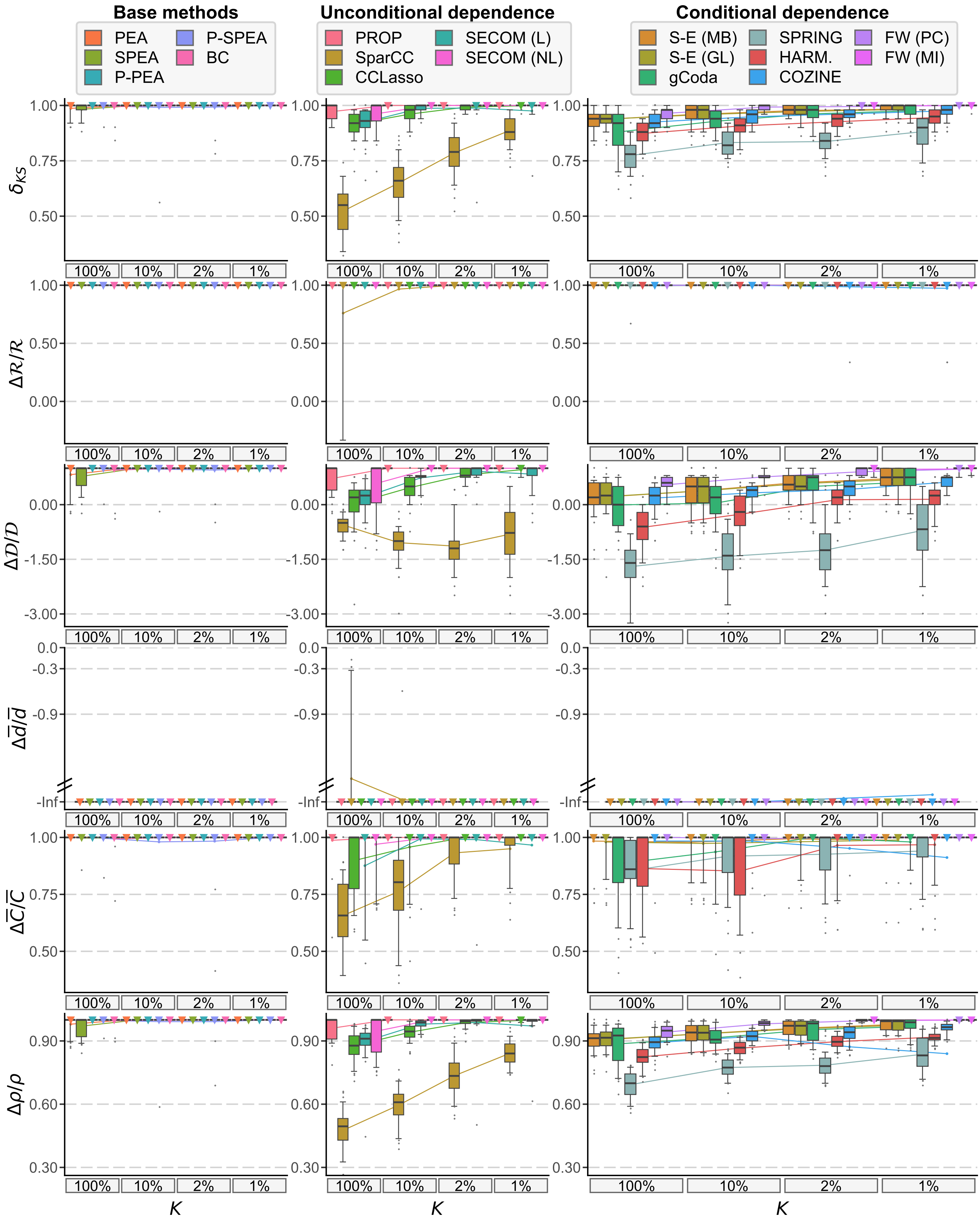

Fig AR – Graph-wise metrics for ER model with medium $\beta-$diversity for decreasing number of extractions. *16S rDNA-seq dataset simulations were performed with different library size fraction $K \in [100\%, 10\%, 2\%, 1\%, 0.5\%]$ (here we show a representative subset of the values). All other simulation parameters were set at their default value $n = 100$, $\rho(\mathcal{G}) = 5\%$, $r_{neg} = 0.05, p = 50$. Methods are grouped based on the implementation strategy (different columns). Note that it may happen that the displayed metric over the 50 iterations had the same value, meaning that boxplots collapse to a single line. In such cases, we plotted a coloured triangle corresponding to the median value of the metric over the 50 iterations.*

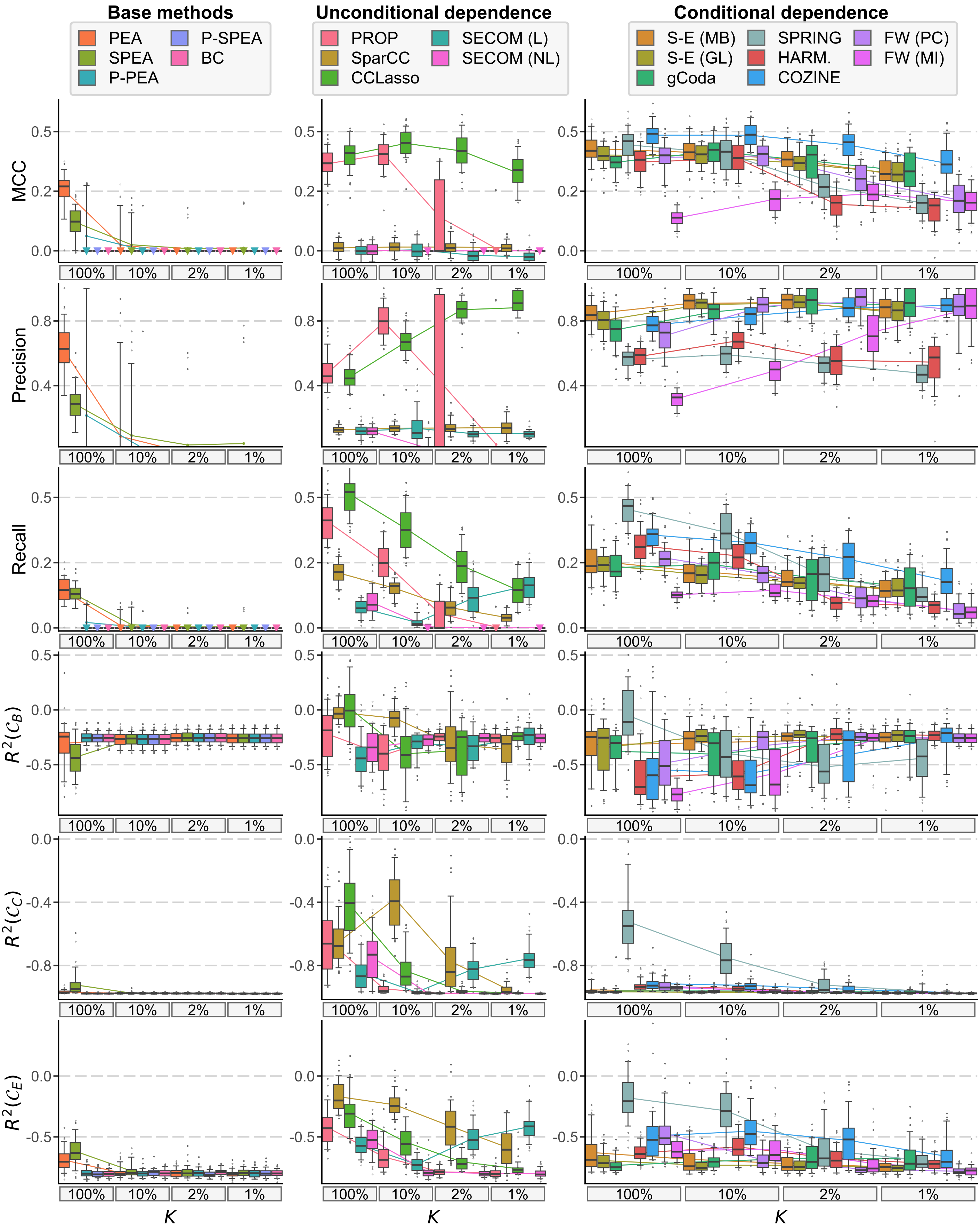

Fig AS – Edge-wise and node-wise metrics for BA model with high $\beta-$diversity for decreasing number of extractions. *16S rDNA-seq dataset simulations were performed with different library size fraction* $K \in [100\%, 10\%, 2\%, 1\%, 0.5\%]$ *(here we show a representative subset of the values). All other simulation parameters were set at their default value* $n = 100$, $\rho(\mathcal{G}) = 5\%$, $r_{neg} = 0.05, p = 50$. *Methods are grouped based on the implementation strategy (different columns). Note that it may happen that the displayed metric over the 50 iterations had the same value, meaning that boxplots collapse to a single line. In such cases, we plotted a coloured triangle corresponding to the median value of the metric over the 50 iterations.*

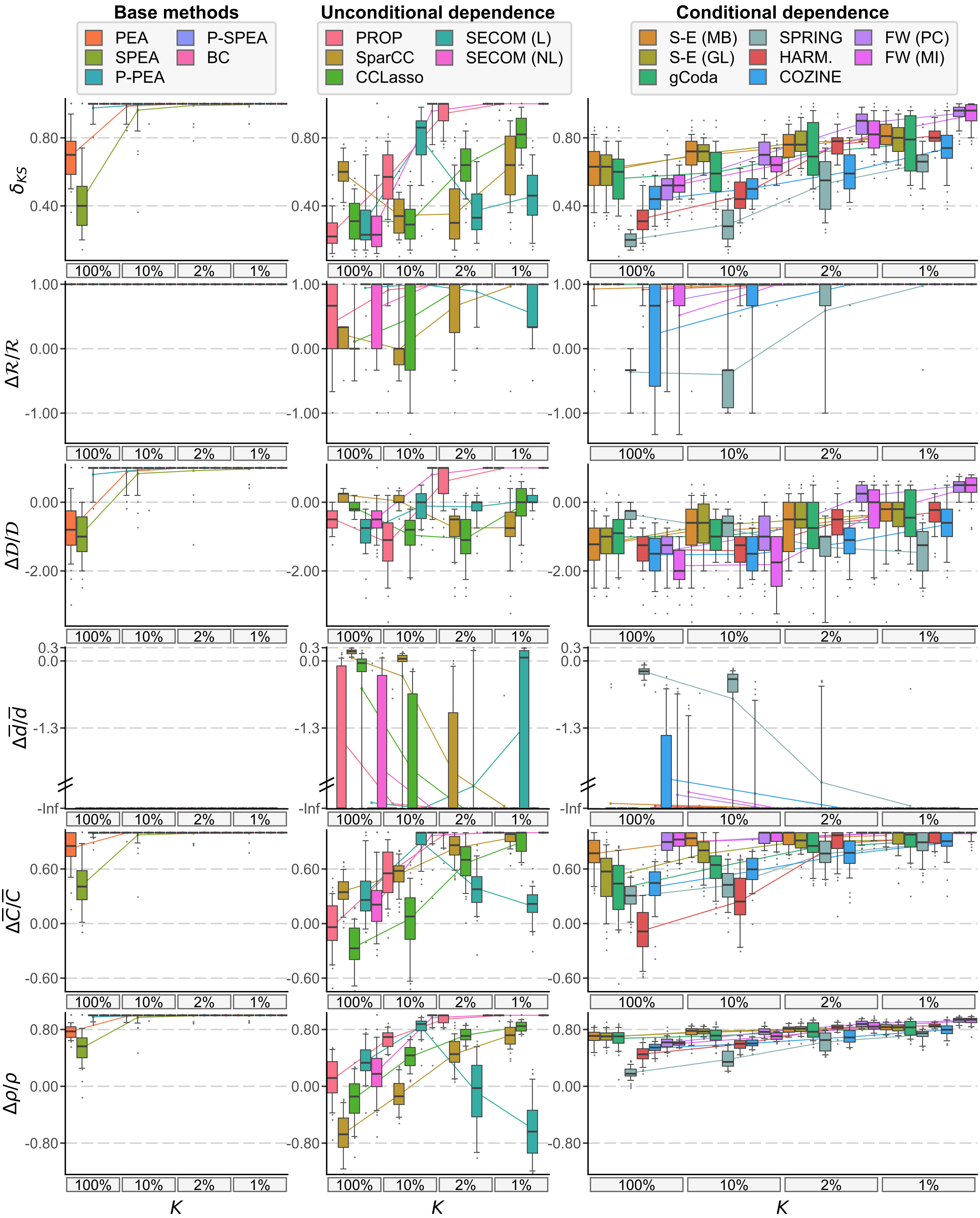

Fig AT – Graph-wise metrics for BA model with high $\beta-$diversity for decreasing number of extractions. *16S rDNA-seq dataset simulations were performed with different library size fraction $K \in [100\%, 10\%, 2\%, 1\%, 0.5\%]$ (here we show a representative subset of the values). All other simulation parameters were set at their default value $n = 100$, $\rho(\mathcal{G}) = 5\%$, $r_{neg} = 0.05, p = 50$. Methods are grouped based on the implementation strategy (different columns). Note that it may happen that the displayed metric over the 50 iterations had the same value, meaning that boxplots collapse to a single line. In such cases, we plotted a coloured triangle corresponding to the median value of the metric over the 50 iterations.*

8 Conclusions

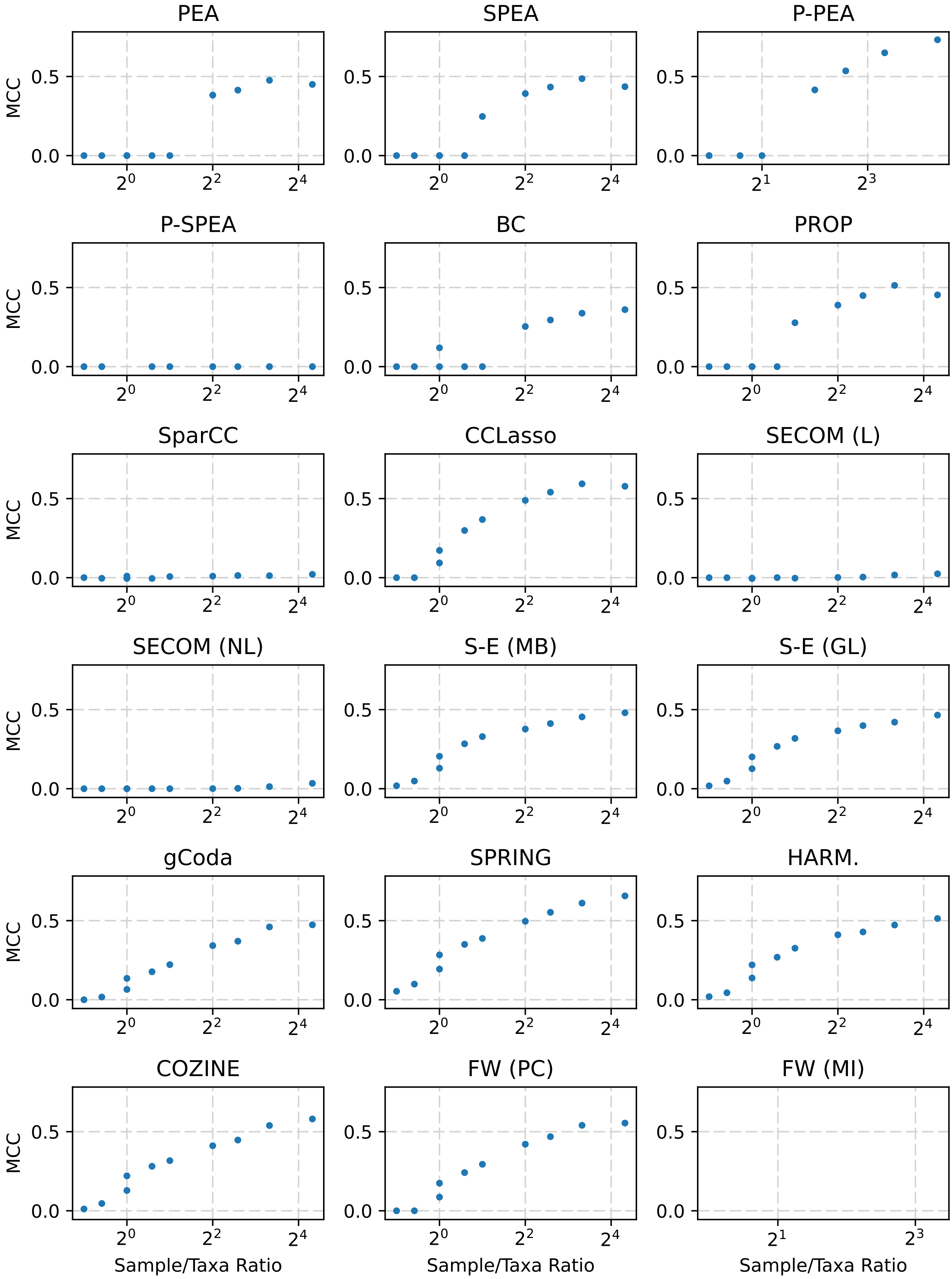

Fig AU – Median MCC with varying sample/taxa ratio. *Values obtained with BA topology and medium β-diversity at the varying of the ratio between the number of samples and the number of species (all other simulation parameters are set to the default values $\rho(\mathcal{G}) = 5\%,\ r_{neg} = 0.05, K = 100\%$). Each panel refers to one method and the x-axis in all panels are in log2-scale.*

References

D. Agyapong, J. R. Propster, J. Marks, and T. D. Hocking. Cross-validation for training and testing co-occurrence network inference algorithms. *BMC Bioinformatics*, 26(1), 2025. doi: 10.1186/s12859-025-06083-7. URL http://dx.doi.org/10.1186/s12859-025-06083-7.

D. Berry and S. Widder. Deciphering microbial interactions and detecting keystone species with co-occurrence networks. *Frontiers in Microbiology*, Volume 5 - 2014, 2014. doi: 10.3389/fmicb.2014.00219. URL https://www.frontiersin.org/journals/microbiology/articles/10.3389/fmicb.2014.00219.

J. D. Brunner, A. J. Robinson, and P. S. G. Chain. Combining compositional data sets introduces error in covariance network reconstruction. *ISME Communications*, 4(1):ycae057, 2024. doi: 10.1093/ismeco/ycae057. URL https://doi.org/10.1093/ismeco/ycae057.

Z. Ghaeli, R. Aghdam, and C. Eslahchi. Evaluating microbial network inference methods: moving beyond synthetic data with reproducibility-driven benchmarks. *BMC Bioinformatics*, 2026. doi: 10.1186/s12859-026-06510-3. URL http://dx.doi.org/10.1186/s12859-026-06510-3.

H. Hirano and K. Takemoto. Difficulty in inferring microbial community structure based on co-occurrence network approaches. *BMC Bioinformatics*, 20:329, 2019. doi: 10.1186/s12859-019-2915-1. URL https://doi.org/10.1186/s12859-019-2915-1.

R. B. Nelsen. *An Introduction to Copulas*. Springer Series in Statistics - Springer, 2006.

S. Weiss, W. Van Treuren, C. Lozupone, K. Faust, J. Friedman, Y. Deng, L. C. Xia, Z. Z. Xu, L. Ursell, E. J. Alm, A. Birmingham, J. A. Cram, J. A. Fuhrman, J. Raes, F. Sun, J. Zhou, and R. Knight. Correlation detection strategies in microbial data sets vary widely in sensitivity and precision. *The ISME Journal*, 10(7): 1669–1681, 2016. doi: 10.1038/ismej.2015.235. URL https://doi.org/10.1038/ismej.2015.235.
