# Supplementary material for: From 16S rDNA-seq count data to microbial interactions: A comprehensive benchmark of co-occurrence network inference methods
Source: PLoS Comput Biol. 2026 Oct 29;22(10):e1014898. doi: 10.1371/journal.pcbi.1014898 (PMC13750663; doi:10.1371/journal.pcbi.1014898)
Supplement: Supplementary file 4 [file pcbi.1014898.s011.pdf]

Dear Editor,

We have addressed all points raised by the three reviewers and incorporated the corresponding changes into the main manuscript, where revisions are highlighted in red, and into the Supplementary Material.

Specifically, we:

- revised the manuscript for grammar, typographical errors, and terminology, and clarified several passages as requested by Reviewers #1, #2, and #3;
- clarified the definition of the ground truth used in our study, as requested by Reviewer #1;
- clarified how failed runs are handled in the evaluation and described the measures adopted to minimize technical issues related to our computing environment, as requested by Reviewer #2;
- corrected errors in the formulas for recall and the coefficient of determination, as requested by Reviewer #2; and corrected figures displaying R^2 to expand the range to negative values;
- expanded the rationale for harmonizing method inputs, and highlighted a preliminary sensitivity analysis comparing default and non-default parameter settings for selected tools, as requested by Reviewer #2;
- revised the manuscript to avoid overstating the realism of simulated data or implying full transferability from synthetic to empirical datasets, as requested by Reviewer #3;
- extended our literature search to include recent benchmarking studies (as requested by Reviewer #3), identifying three additional manuscripts that met our inclusion criteria and bringing the total number of previous benchmarking studies considered to six. We updated Supplementary Table 3 accordingly and revised the main manuscript both to acknowledge these recent benchmarking efforts and to incorporate the information they provide;
- added an analysis comparing N2SIMBA-generated count data with real datasets, as requested by Reviewer #3. Based on characteristics previously investigated in the literature, as well as those suggested by the reviewer, we assessed sparsity/prevalence, variability, abundance, and the relationships among these properties on our simulated data and 6 real dataset;
- assessed the consistency of our findings, obtained using the N2SIMBA simulation framework, with results from previous studies based on different simulation approaches. Rather than generating additional synthetic datasets, we leveraged the six published benchmarks using different simulation models (i.e. parametric distributions, generalized Lotka–Volterra models, NORTA, and resampling of real data), and compared their findings with ours in terms of method ranking and the effects of data characteristics on performance.

We believe that the manuscript has been substantially improved by the reviewers' comments and suggestions, and we hope that it is now suitable for publication in *PLOS Computational Biology*.

We refer the Editor to the detailed point-by-point responses to the reviewers for further information.

Best regards,
Giacomo Baruzzo

Reviewer #1

I enjoyed reading this extensive and thorough analysis of different approaches used to infer community interaction networks. The manuscript is well written and I appreciate how clearly the authors describe each method tested, parameters considered/ controlled, and the metrics used to assess results. While I do not have the expertise to scrutinize the mathematics, I greatly appreciate the detailed explanation the authors provided for their methodology. They clearly detail how they build on prior analyses and extend the scope of methods and scenarios tested, and emphasize the limitations of every approach.
Although the authors do not advocate for any specific approach (as it does not appear feasible due to the complexity of different real-world environmental datasets), this study serves as a useful resource for anyone performing community interaction network assessments, providing a comprehensive overview of the limitations of many common approaches. Reseachers would benefit from considering these results both before planning an experiment, to inform study design, as well during data analysis, to reflect on which method to employ and the limitations of whatever model(s) is selected.
While I do find the paper to be very well written overall, I have provided specific edits for grammar (mostly minor, such as frequently missing or incorrectly placed commas), as well as a few points that require clarifying.
Please see below for specific comments/edits:

We thank the reviewer for the positive feedback. We carefully revised the grammar, and rephrased or clarified the reported points.

Abstract
Rephrase the sentence:
"To derive ecological associations many inference approaches have been developed, here we focus on co-occurrence computational methods, in which associations are inferred from metagenomic abundance data by measuring the statistical dependence between species counts across different sampled populations."
to:
"Many inference approaches have been developed to derive ecological associations. Here, we focus on co-occurrence computational methods, in which associations are inferred from metagenomic abundance data by measuring the statistical dependence between species counts across different sampled populations."
Remove "a" in phrase "leading to a scarce application":
The limitations in culture-based experiments have hindered the ability to assess these methods' validity, leading to a scarce application in metagenomics studies.

We applied the proposed modifications to the Abstract, they are highlighted in red in the revised version of the manuscript.

Author summary
Rephrase: "The use of realistic simulated data was necessary due to the lack of reliable ground- truth references from culture-based experiments that has limited systematic evaluation of method performance."
to
"The use of realistic simulated data was necessary due to the lack of reliable ground- truth references from culture-based experiments, which has limited systematic evaluation of method performance."

We applied the proposed modifications to the Author Summary, they are highlighted in red in the revised version of the manuscript.

Introduction
- Line 16 – rephrase "to a level" to "to reach a level"
- Line 50: add comma to "To solve part of these issues, several co-occurrence-based methods 50 have been proposed during the years."
- Lines 53-55: Rephrase to "Different metrics can be used to measure different types of associations, including Pearson Correlation (linear), Spearman Correlation (monotonic), Bray-Curtis dissimilarity, and Maximal Information Coefficient (MIC) [35] for non-linear associations."
- Line 57: NGS is written but the acronym is never identified. Please add "(NGS)" after the first mention of next generation sequencing, on line 15
- Line 58: add comma after "(like 16S rDNA-seq data)"
- Line 62: add comma after "To avoid this limitation"
- Line 63: add comma after "SparCC [38]"
- Line 64: remove comma after "expensive"
- Line 67: add comma after "However"
- Line 87: replace "then" with "to"
- Line 89: add comma after "have criticised this approach [48]"
- Line 601: be consistent in how you write "nonlinear"…sometimes written in doc as non linear or non-linear". Change to "non-linear"
- Line 109: add comma after "measures (Precision, Recall, and Matthews correlation coefficient (MCC))"
- Line 115: add comma after "results"

We applied the proposed modifications in the Introduction section. They are highlighted in red in the revised version of the manuscript.

Materials and methods
- Line 161: I may be misinterpreting, as I am unfamiliar with the mathematics employed in this study. To clarify: based on the example in the subsequent sentence, do you mean that $D_{\alpha\beta}$ denotes the fraction of resource R_β that is converted to R_α (as written in the manuscript), or does it denote the fraction of resource R_α that is converted to R_β?

The $D\alpha\beta$ denotes the fraction of resource $R\beta$ that is converted to $R\alpha$

- As written, “if $c_{i,\alpha} \neq 0, D_{\alpha\beta} \neq 0, c_{j,\beta} \neq 0$ then species i is beneficial to species j”, but if $D_{\alpha\beta}$ truly denotes the fraction of resource R_β that is converted to R_α, would it be more appropriate to say that species j is beneficial to species i?

As pointed out the species relationship was swapped, we corrected the manuscript with the correct relationship “species j is beneficial to species i” (line 171 Methods section)

- Lines 277-278: maintain consistent tone. The rest of the paragraph was in past tense, so change “is determined” to “was determined” and “is then” to “was then”. Also, add a comma after “$A_{ij} = 1$”

Addressed, changed to the past tense

- Lines 299, Figure 1 title & caption, 324: these are the only instances of the words “unconditioned” and “conditioned” appearing. Are these interchangable with “unconditional” and “conditional”? If not, please replace all 6 instances accordingly.

The terms “unconditioned” and “conditioned” were used as synonyms of the terms “unconditional” and “conditional”. To improve clarity we replaced all the instances with the“unconditioned” and “conditioned” terms to be uniform across the manuscript. We retained the last “conditioned” in Figure 1 caption as in this occasion it is the verb “to condition” and not an adjective that we use to classify the different methods.

- Line 139: remove “As” from “As in this study,”
- Line 140: change comma after “graphs” to a semicolon
- Line 146: add comma after “N2SIMBA [54]”
- Line 153: “Where” should be lowercase
- Line 157: add “and” between “in S1 File,” and “ for a detailed…”
- Line 165: add comma after “N2SIMBA” and remove comma after “generated”
- Line 166: add comma after “details”
- Line 169: add comma after “known”
- Line 172: change “which” to “that”
- Line 173: remove “it”
- Line 174: add comma after “details”
- Line 184: add comma after “initialized”
- Line 186: add commas after “Then” and “community”
- Line 187: remove extra “the” in “…simulating the the different environmental…”
- Line 243: remove comma after “[48–50],”
- Line 263: add comma after “data”
- Line 265: change comma after “pseudocount” to a semicolon
- Line 268: change comma after “matrix Σ [63]” to a semicolon
- Line 269: add comma after “Spearman correlation”
- Line 270: add comma after “network”
- Line 276: remove comma after “testing”
- Line 281: remove “that have been”
- Line 285: add comma after “methods”
- Line 286: add comma after “SECOM [46]”
- Lines 288-289: rephrase to “Spearman correlation coefficient and distance correlation coefficient [66]), respectively.”
- Lines 289 – 290: rephrase “For all the methods no preprocessing, like log-ratio transformations, was performed as they are methods developed” to “No preprocessing, such as log-ratio transformations, was performed for any of the methods, as they are methods developed…”
- Line 292: add comma after “Proportionality”
- Line 295: add comma after “matrix A”
- Line 296: remove comma after “testing”
- Figure 1 caption: add comma after “In the real interaction network”
- Line 300: replace “As” with “By”
- Line 316: add comma after “normality”
- Line 323: change “which” to “that”
- Line 324: add comma after “of the methods”
- Line 339: add comma after “j)”
- Line 341: add comma after “matrix **A**”
- Line 345: add comma after “classification [69]”
- Line 358: add comma after “$i \in V$”
- Line 360: add comma after “(i,j)”
- Lines 361 - 362: change “paths length” to “paths’ lengths”
- Line 367: remove “it”
- Line 372: remove “Eigenvector centrality” after the colon
- Line 373: change comma after “neighbours” to semicolon
- Line 381: rephrase to “As a summary statistic for all node-wise metrics, we computed the coefficient…”
- Line 386: add comma after “measures”
- Line 388: add comma after “structure”
- Line 391: remove extra “the” in “expresses the the maximum…”
- Line 395: add comma after “0”
- Line 396: add commas after “similar” and “1”
- Line 400: remove “is”
- Line 403: remove “is”
- Line 406: add comma after “vertex”
- Line 407: change comma after “undefined” to a period and start a new sentence: “In this work, we…”
- Line 416: remove period after “clusters”
- Line 417: “With” should be lowercase
- Line 420: add comma after “graph $\hat{M}$”

We applied the proposed modifications in the Material and Method section. They are highlighted in red in the revised version of the manuscript.

Results and Discussion

Lines 586-588: rephrase. What do you mean by “if not for”?

We mean that all the metrics with the exception of the diameter did not resemble the true ones. We rephrase as follow: Rephrased “if not for” in “except”.

- Line 459: add comma after “File)”
- Line 459: change “see consistently” to “consistently observe”
- Line 463: change “the one with” to “yielding”
- Line 474: add comma after “level” and change “methods” to “methods’”
- Line 591: change “had also” to “also had”
- Line 592: add comma after “general”
- Line 594: “(Linear)” should be lowercase
- Line 596: change “less” to “least”
- Line 600: change semicolon after “methods” to a comma

• Figure 2, Figure 3, Figure 7, Figure 8, and Figure 9 titles: change "Methods" to "Methods'"<br>• Line 481: add comma after "gut)" and remove comma after "difficult"<br>• Line 483: change "have" to "has"<br>• Line 499: add comma after "one" and change "often it" to "it often"<br>• Line 506: add comma after "Fig 3A"<br>• Line 516: change comma after "performance)" to period and start a new sentence (remove "also,": "Pearson and Spearman's correlation…"<br>• Line 517: change "lower" to "poorer"<br>• Line 526: add comma after "SPRING (R^2 = 0.43 at n = 1000)"<br>• Line 528: add comma after "graph"<br>• Line 529: change "at" to "to", and add a comma after "Restricting the comparison at the 5 highest centrality nodes"<br>• Figure 3, Figure 5, Figure 7, Figure 8, and Figure 9 captions: make "results" plural in "Here we show the result"<br>• Line 530: change comma after "value" to semicolon<br>• Line 531: add comma after "SPRING"<br>• Line 533: add comma after "gains"<br>• Line 538: add comma after "Fig. 3C"<br>• Line 539: remove comma after "similar"<br>• Line 541: add comma after "(Non Linear)", and make lowercase and add dash to be "non-linear". "(Linear)" should also be lowercase<br>• Line 542: add comma after "0"<br>• Line 544: add comma after "increases"<br>• Line 547: remove commas after "sparser" and "precise", and add comma after "graphs"<br>• Line 554: add "us" after "allows"<br>• Line 556: add comma after "implementations)" and add "a" after "with"<br>• Line 557: change comma after "1s" to a period and start a new sentence: "In contrast, HARMONIES…". Change "worse" to "worst", and add "a" after "with"<br>• Figure 4 caption: change "where" to "were"<br>• Line 571: change "previously" to "before"<br>• Line 575: add comma after "p = 50"<br>• Line 576: add comma after "(Fig. 5A)"<br>• Line 581: add comma after "SPRING"<br>• Line 589: remove ", however,". Sentence should read as "The only exceptions are SparCC, which has MCC =0, and SPRING."	• Line 601: be consistent in how you write "non-linear"…sometimes written in doc as non linear or nonlinear". Change to "Non-linear"<br>• Line 603: remove comma after "species"<br>• Line 613: add comma after "methods"<br>• Line 619: remove "from" and add a comma after "increases"<br>• Line 620: change comma after "networks" to semicolon<br>• Line 622: remove "in" in "(in S3 File)" and add comma after parentheses<br>• Line 628: change comma after "S3 File)" to period and start new sentence: "Taking the mean loss in recall across methods, we obtain"<br>• Line 629: add comma after "-0.14", change "being" to "begin", and change comma after "with" to period and start a new sentence: "Looking at the best…"<br>• Line 630: add comma after "methods"<br>• Line 642: add comma after "increases"<br>• Line 643: add comma after "10%"<br>• Line 645: change comma after "none" to semicolon<br>• Line 648: add comma after "WS model"<br>• Line 650: remove comma after "network"<br>• Lines 650 – 652: rephrase to "The true network topology, the β-diversity between samples, and the network density did not have any major impacts on the computational burden…"<br>• Line 659: change "infer" to "inference" and add comma after "resources"<br>• Line 664: add comma after "Fig. 8A"<br>• Line 666: rephrase to "Conditional dependence methods also experience…"<br>• Line 671: remove "Also" and add "metrics" after "graph-wise"<br>• Line 674: add comma after "Fig. 8"<br>• Line 677: rephrase to "…dynamics and causes interactions…"<br>• Line 678: add "to" before "cancel", and add "-" between "3" and "species" to be "3-species"<br>• Line 680: add comma after "magnitude"<br>• Line 682: change "method'" to "methods'"<br>• Line 688: rephrase to "Due to the compositional nature of sequencing count data, the role of library size has…"<br>• Line 690: change "also" to "as well as"<br>• Line 703: change "decreases" to "decreased"<br>• Line 704: add comma after "Fig. 9B"<br>• Line 707: remove "however"

We applied the proposed modifications in the Results and Discussion section. They are highlighted in red in the revised version of the manuscript.

Conclusion

- Not sure if I'm misinterpreting this but in the Figure 10A image, columns corresponding to Density = 10% and = 15%, and Neg. ratio = 20% are missing a rank "3", and columns corresponding to #Taxa = 200 and Library size = 10% are missing a rank "2"

The missing ranks are a result of a quirk the R package used to generate the figure and how it handles ties in the ranks. Basically for columns Density = 10% and = 15%, and Neg. ratio = 20% since there are two methods tied at second place but the total number of ranked entries is 3 no other rank is assigned and thus rank 3 is missing. For columns #Taxa = 200 and Library size = 10% since there are 2 methods tied for first place rank 2 is skipped (because there are already 2 ranked entries) but since the number of ranked entries is < 3 it assigns another rank (rank 3).
We revised the figure so that each column now displays all three rankings, regardless of ties.

- *Note – "scalability" is not mentioned until the Conclusion section. Try to reference it at least once before, when mentioning the different metrics used to assess computational burden

We removed the term "scalability" and replaced it with "computational burden". We also modified Fig 10 to remove the term scalability from it.

- *throughout document – be consistent in how you denote figures. Sometimes they are referenced as "Fig #", other times as "Fig. #". Select one format and change all instances accordingly.

Changed figure notation to a consistent format using "Fig #"

• Line 716: remove "also"<br>• Line 717: change "by considering" to "to"	• Line 738: rephrase to "The β−diversity between samples also had an impact…"

• Line 719: change “at” to “by”<br>• Line 720: change “level” to “metrics”<br>• Line 724: add “network” before “density” and replace “and” with a comma to read “network density, ratio…”<br>• Line 725: change “Consistently” to “Consistent”<br>• Line 730: change “cautions” to “caution”<br>• Lines 734-735: rephrase to “The methods obtained the best results with the Watts-Strogatz model (Fig. 2), as found by…”<br>• Line 737: change “model” to “models”	Figure 10 caption: change “methods” to “methods’” in “A summary of the methods performances at various…”. Two instances of “methods ranks” should also be changed to “methods’ ranks”<br>Line 819: add “by” after “gaps”<br>Line 842: add comma after “example”<br>Line 851: add “underscore the fact that” after between “and” and “that” to read “…and underscore the fact that the problem…”

We applied the proposed modifications in the Conclusion section. They are highlighted in red in the revised version of the manuscript.

*I am not providing detailed reviews of the supplementary materials, but please take these notes into consideration and revise accordingly when appropriate (for ex., consistent writing of “non-linear”, correct punctionation, etc.).

A few examples:

- S1 File – Figure 3 caption: change “tree” to “three”
- S1 File – pg 7 – change “it” to “they” in phrase “and thus it will no longer be supplied externally…”
- S2 File – Figure 2 caption: change “pannels” to “panels”
- S3 File – Table 1 – change “non linear” to “non-linear”
- S3 File – Figure 46 – change title to “Median MCC with varying sample/taxa ratio”

We addressed the specific points raised and did a general revision of the supplementary materials to improve consistency of the writing, grammar and correct punctuation.

Reviewer #2

This paper focus on a difficult task in microbiome analysis, such as the identification of microbial interactions, that while addressed by a plethora of tools, remains difficult to evaluate for the limited availability of experimentally validated "ground truth" datasets.
The authors produced the largest benchmark to date, evaluating 18 approaches across 189 parameters (and 50 replicats). The simulations were done using N2SIMBA, which is built on a consumer-resource model, which should avoid baking a covariance structure directly into the ground truth.

The analysis found the methods work only in a narrow and often unrealistic scenario, when many samples (N >> P) a good library size and low density and limited negative interactions are dominating the picture.
Interestingly, the network density results being a key factor that every method fails to successfully deal with.

Noteworthy is the finding that higher beta diversity, contrary to previous reports, helps the methods.

I would like to thank the authors for the efforts done in making this study reproducible, with a Zenodo dataset, GitLab repository and singularity containers.
I recommend the publication of this paper with some suggestions to improve the overall clarity

We thank the reviewer for the positive feedback. We addressed reviewer suggestions in the following.

CLARIFICATIONS
----

1) Is the "ground truth" the right thing to grade against?

The authors build their simulated data with N2SIMBA. They start from a network A and from that they construct the matrix C and D, which say who eats which resource and how resources get converted. So A is the starting point, and then the metrics is how well a method did recover A?
A describes a mechanistic, resource-mediated interaction, while the methods only see the counts X and can measure statistical patterns in these counts. Some confounding factors can be if species 1 and species 2 both feed on the same by-produc of species C. Similarly as the authors demonstrate, real interaction can disappear (positive and negative interactions will cancel each other.)

We thank the reviewer for raising this important point. We agree that starting from count data, in both real and simulated settings, inherently limits the recoverability of certain interactions. We also recognize that experimental and sequencing biases further complicate this task, and that some classes of methods are intrinsically unable to correctly identify specific interaction types (for example, conditional-dependence approaches may infer spurious edges).

However, because the methods included in our benchmark are explicitly designed to reconstruct microbial interaction networks from count data, and because this is also how their outputs are intended to be interpreted by end users, we believe that evaluating them against the original interaction network A used to generate the data provides the most

transparent assessment of their performance. Moreover, benchmarking against the original adjacency matrix A is direct and unambiguous, as the evaluated methods return either an inferred network or a corresponding list of edges.

We have added a paragraph to the Methods section (line 355) to explicitly clarify this rationale. The paragraph is also reported here for reviewer convenience:

> ***"To evaluate method performance, we compared the ground-truth network used to generate the simulated data with the inferred association network. Some microbial interactions may be intrinsically unidentifiable from count data alone, in both real and simulated settings, and experimental or sequencing biases can introduce additional artefacts. Nevertheless, because network inference methods aim to reconstruct the underlying community interaction network, benchmarking against the original adjacency matrix $\mathbf{A}$ provides the most direct and unambiguous measure of performance. This comparison is also straightforward, as the evaluated methods return either an inferred network or a corresponding list of edges."***

2) Sensitivity setting

Every method has defaults like the stability cutoff of SPIC-EASY and SPRING and penalty lambda in lasso based methods. The authors only used the package defaults (and the ability of the authors to define a good default, or a default that performs well in this simulation).
Methods that were found having a low recall might be methods offering a weak default threshold.
Could it be useful to sweep the cutoffs with a threshold-free metric like AUPRC?

We fully agree that parameter setting can have an impact on the methods' performance. We considered using threshold-free metric like AUPRC in our study but we concluded that it would not be feasible as some methods do not output an easily interpretable score, or at least a score that can be used for thresholding. For example the methods based on the StARS algorithm output a stability score, however the stability is only used to select the optimal lambda value which ensures that the mean instability is < threshold) and the presence/absence of edges is not determined by the instability score but from the regularization parameter lambda.
On this subject we did perform a limited sensitivity analysis (in supplementary File S2) that did not show particularly strong effects of the parameters on the methods' results. We think that a more in depth analysis could be valuable but is outside the scope of this benchmark. We better referenced the small sensitivity analysis performed by adding the phrase:

> ***"We also conducted a preliminary sensitivity analysis to explore the effects of the parameters for some of the methods (Section 3 in S2 File), which did not highlight strong effects on the methods performance and was thus not explored further. "***

in the Methods section at line 275.

3) Treating failed runs

Failed runs shouldn't be scored as bad accuracy, i.e. "the method ran and got the wrong answer" and "the method never ran at all" are two very different things, but the ranking mixes them together.
I would separate these and also add clarifications on why some methods didnt run to see if it's likely a package insrinsinc limitation or environmental (our HPC does not support X).

We agree with the reviewer that failed runs should not be treated as incorrect inferences, and we therefore considered these two outcomes separately in our evaluation. Based on our tests, failures were most likely due to software bugs or unhandled edge cases rather than to the specific computing environment. We reproduced the same errors on a different system, namely a workstation in our laboratory. In addition, we used containerized versions of the software to further reduce the possibility that failures were caused by system-specific configurations.
We better clarified this in the manuscript and corrected any statement that may incorrectly state otherwise:

- ***changed caption of Fig 10 in the Conclusions section to "The mean was computed over the successful iterations off of the 50 simulated with the same parameter values"***
- ***added statement in the Methods section at line 368 "Runs in which a method failed were excluded from the evaluation of that method's performance. Only successful runs were considered, and performance for a given simulation scenario was reported only when at least 10 of the 50 iterations completed successfully. In our tests, these failures did not appear to be specific to our computing environment, as they were also observed on other systems. Moreover, the use of software containers reduces the likelihood that the failures were caused by system-specific software configurations."***

MINOR POINTS TO REVIEW
----

1) Terminology: I would consider using 'association network' rather than 'interaction network' when referring to the output of cross-sectional co-occurrence methods, and reserve interaction for the simulator's mechanistic reference.

We changed the term interaction network to association network when referring to the output of co-occurrence inference methods throughout the manuscript. Here a list of the changes

- ***Line 34, 72, 82, 87 and 109 in the Introduction section***

- ***Line 294, 319 and 335 in the Methods section***
- ***Caption of Fig 4 and 6***

2) Recall formula is printed TP/(TP+FP), while it should be TP/(TP+FN)

Addressed

3) In the Node-wise metrics section the author write that as a summary statistics they compute "the coefficient of the determination in R2" R2=∑i(Ci−C¯)2∑i(C^i−C¯)2, while R2 is usually 1-∑i(Ci−C¯)2∑i(Ci−C^i)2, where in the numerator we measure the errors between true and inferred values. A further hint is that a coefficient of determination can go negative while the paper reports positive numbers, that might be a squared Pearson correlation?

The metric used in the study is the coefficient of determination computed as R2 = 1 - SS_res / SS_tot = 1-∑i(Ci−C^)2 / ∑i(Ci −C¯)2 . Inn the paper it was written with the alternative formulation, valid for ordinary least square regression, of R2 = SS_reg / SS_tot = ∑i(C^i−C¯)2 / ∑i(Ci−C¯)2 which was not correct as in this case the prediction does not derive from OLS regression. The formula in the paper is now corrected to R2 = 1-∑i(Ci−C^)2 / ∑i(Ci−C¯)2. Please note that the correct formulation was used in all computations; the error is limited to the formula reported in the manuscript.
In the original version we clipped values between 0 and 1 for ease of representation, we modified this to restrict values between -1 and 1 by applying this transformation R^2 = R^2 / (1 - R^2) for negative values (R^2<0). We updated all the figures containing any node-wise summary statistic R^2.

4) Turning off internal filtering to equalize the input is reasonable for comparability, but it removes methods (SECOM) from their intended pipeline, and the discussion rightly flags this for SECOM. I'd foreground this trade-off earlier.

Added a statement in the Methods section at line 273 to better explicit the possible impact on performance: "Although this takes the methods out of their intended pipeline and may affect performance, it was necessary to ensure that the results remained comparable"

Reviewer #3

This manuscript presents a large benchmark of 18 co-occurrence network inference methods for 16S rDNA-seq count data, using N2SIMBA/MiCRM simulations with known interaction networks to compare edge-level accuracy, topology-level behavior, and computational performance across a wide range of simulated scenarios. The study is valuable because method choice in microbial association analysis remains difficult, and the authors make a serious attempt to evaluate many commonly used tools under controlled conditions. My main concerns focus on whether the reported method rankings can be generalized beyond the specific synthetic design space. The manuscript should more clearly qualify its practical recommendations, provide a minimal fitness-for-purpose comparison between simulated and real 16S count tables, and assess whether the main rankings are stable under alternative data-generating processes or perturbations.

Major Comments:

1. Abstract, Manuscript Page 1: "valid results only in a restricted ... more realistic scenarios"; Author summary, Page 2: "realistic simulated data ... general applicability"; Results and Discussion, Page 14, lines 441-447: "generated realistic 16S datasets ... default simulator parameters"; Results and Discussion, Page 19, lines 601-604: "more realistic scenarios ... increasing the number of species."

The manuscript's broad practical conclusion remains stronger than the validation basis supports. The concern is not the use of simulation itself, which is a reasonable way to obtain known ground truth, but the repeated use of "realistic" language around simulated scenarios. The abstract first states that the benchmark uses synthetic 16S count data, but then frames the main conclusion as a decline in "more realistic scenarios"; the author summary describes "realistic simulated data" as supporting general applicability; and the Results/Discussion again calls simulator-generated datasets or scenarios realistic. Because true empirical microbiome data generally lack experimentally verified ground-truth ecological networks, the benchmark does not solve the core unverifiability problem for real datasets. It evaluates methods under synthetic, 16S-like count-generating assumptions. The authors should revise the abstract, discussion, and conclusion to distinguish real empirical validation from more realistic simulated scenarios, avoid implying that real-data performance has been established, and make practical recommendations conditional on simulator realism and transferability from synthetic to empirical data.

We thank the reviewer for this insightful comment and agree that results obtained from simulated datasets cannot serve as a full proxy for real-world empirical validation. To ensure our language does not overstate the transferability of our findings to real data, we have revised the manuscript to remove terminology that might imply full equivalence between synthetic and empirical datasets. Specifically:

- ***We removed the term "realistic" in the Author Summary.***
- ***We removed the term "realistic" in the Results and Discussion section at line 483***

Regarding the statements noting a decline in performance under "more realistic scenarios", our intention was strictly to refer to the simulation parameters rather than the generated data itself. For example, increasing the parameter that controls simulated community size from 50 to 200 species better reflects the complexity of empirical microbial communities and can therefore be considered more realistic in this respect. This increase consistently led to lower performance across the evaluated methods.
To prevent any misinterpretation while preserving this important consideration, we have updated these specific sentences in the manuscript to explicitly refer to "more realistic simulation parameters" rather than "realistic scenarios," thereby maintaining a clear distinction between simulated complexity and true empirical validation. Specifically:

- ***We changed the statement "in more realistic scenarios" to "with more realistic simulation parameters" in the Abstract***
- ***We changed the statement "more realistic scenarios" to "more realistic simulation parameters" in the Results and Discussion section at line 644***
- ***We changed the term "unrealistic" to "obtained with unrealistic simulation parameters" in the Conclusion section at line 838***

2. Introduction, Page 4, lines 98-100: "comprehensive benchmark ... with respect to previous comparative studies [48-50]"; Introduction, Page 4, lines 101-105: "largest selection ... broad range of scenarios"; Materials and methods, Manuscript Page 7: "standalone inference methods ... selected from previous comparative studies."

The authors use three previous comparative studies in the Introduction to substantiate the breadth of method coverage and to motivate the selection of methods included in the benchmark. However, the three comparative studies used as the main benchmark anchors appear to date mainly from 2014–2019. Given the rapid development of microbial co-occurrence and association-network benchmarking, this literature base may no longer be sufficient to support the manuscript's claim of comprehensive methodological comparison. I suggest that the authors expand this section by explicitly including more recent benchmark or validation studies in the co-occurrence network field, such as work on the error introduced by combining compositional data sets in covariance network reconstruction, as well as studies examining the difficulty of inferring microbial community structure from co-occurrence network approaches. The authors should provide a concise and up-to-date table of relevant comparative or validation studies, indicating which methods, simulation scenarios, compositionality issues, and network-inference assumptions each study addressed. They should then clarify how these more recent studies informed the present benchmark design, method selection, performance metrics, and interpretation, or explain why particular studies or frameworks were excluded.

We thank the reviewer for this constructive feedback and for highlighting the rapid development of microbial co-occurrence network benchmarking.
When we initiated this project in 2024, we conducted an extensive literature search for existing benchmarking studies and identified the three studies included in our original analysis. We focused on manuscripts devoted entirely, or primarily, to comparing network inference methods for metagenomic data and providing a standalone, independent benchmarking effort. We excluded studies in which benchmarking was limited in scope or not the main objective, such as articles introducing a new network inference method or metagenomic analysis pipeline. Using the same inclusion criteria, we have now updated our literature search.

As a first approach, we performed a systematic and structured search in PubMed using the following query:

> *(((network[Title/Abstract]) OR (graph[Title/Abstract]))) AND (((benchmark[Title/Abstract]) OR (assessment[Title/Abstract]))) AND (((microbiota[Title/Abstract]) OR (microbial[Title/Abstract])))*

Among the retrieved studies, we identified the following article as meeting our inclusion criteria:

- ***Ghaeli, Z., Aghdam, R. & Eslahchi, C. Evaluating microbial network inference methods: moving beyond synthetic data with reproducibility-driven benchmarks. BMC Bioinformatics (2026). https://doi.org/10.1186/s12859-026-06510-3***

This study, published on 20 June 2026 and currently available only as an unedited version, compares six approaches (gCoda, OIPCQ, two SPIEC-EASI variants, SPRING and SparCC) and evaluates their performance using NORTA simulations. The only method not included in our benchmark, OIPCQ, was originally developed for gene regulatory network inference and appears to have been adapted to metagenomic data in this study (OIPCQ methods was developed by most of the benchmarking study authors, so we guess they have the knowledge for using it outside its intended scope). Notably, it was not among the best-performing approaches reported in that benchmark.

As a second approach, we examined all papers citing the most recent benchmark previously included in our manuscript, Hirano and Takemoto (2019) "Difficulty in inferring microbial community structure based on co-occurrence network approaches." Our rationale was that recent benchmarking studies would likely reference this earlier benchmarking effort. In addition to the study above, this search identified two further publications that met our inclusion criteria:

- ***James D Brunner, Aaron J Robinson, Patrick S G Chain, Combining compositional data sets introduces error in covariance network reconstruction, ISME Communications, Volume 4, Issue 1, 2024, ycae057, https://doi.org/10.1093/ismeco/ycae057***

This study compares six approaches, including three ad hoc baselines based on covariance of CLR-transformed data, SparCC, and two GLASSO variants, and evaluates them using data simulated with a generalized Lotka–Volterra model.

- ***Agyapong, D., Propster, J.R., Marks, J. et al. Cross-validation for training and testing co-occurrence network inference algorithms. BMC Bioinformatics 26, 74 (2025). https://doi.org/10.1186/s12859-025-06083-7***

This study compares four methods (Pearson correlation, Spearman correlation, LASSO, and GLASSO) using a novel evaluation framework based on real data.

We have now incorporated these three additional studies into the manuscript and updated the relevant text accordingly, both to acknowledge these recent benchmarking efforts and to incorporate the information they provide.

In particular, we have modified the Introduction and Conclusion sections to explicitly integrate these 3 additional recent studies:

- *Added citation to newly included benchmarks in the Introduction section at line 94*
  "The simplest approach to generate simulated data is the use of parametric distributions and to then consider the covariance matrix as a proxy for the ground truth interaction network [40, 48, 49]"
- *Added citation to newly included benchmarks in the Introduction section at line 107*
  "Our contribution, with respect to previous comparative studies [48–52, 56] ..."
- *Added citation to newly included benchmarks in the Methods section at line263*
  "All standalone microbial associations inference methods present in previous comparative studies [48–52, 56]..."
- *Added citation to newly included benchmarks in the Conclusion section at line 762*
  "Considering also previous valuable studies [48 –52,56], ..."
- *Added the newly included benchmarks in the Conclusion section when discussing the limitations of co-occurrence inference methods, line 772*
  "Consistent with previous studies [48– 52], the results show the limitations of co-occurrence methods in inferring microbial associations, …"
- *Added a new statement discussing the effect of network topology that includes the newly included benchmarks in the Conclusion section, line 784*
  "The specific challenge of recovering scale-free (Barab´asi–Albert) network structures, in particular, is a consistently observed in many previous assessments [49–51]."
- *Added the newly included benchmarks in the Conclusion section when discussing the effect of the number of samples, line 795*
  "Concerning specific simulation parameters, the methods showed an increase in performance as the number of samples increased (Fig 3), as expected and in agreement with previous studies [48, 50, 51, 56]."
- *Updated a phrase in the Conclusion section at line 833*
  "In general, conditional dependence methods have higher and more consistent performance, with SPRING achieving the best results (consistent with the findings of the only previous study that included this tool [49]), across both the various scenarios considered (Fig 10A) and the different evaluation metrics (Fig 10B).
- *Added the newly included benchmarks in the Conclusion section when discussing the performance of baseline methods compared to purpose developed tools, line 835*
  "Although it is true that some base methods, coming from classical correlation statistics, have comparable results with ad-hoc tools, in some occasions, as highlighted by previous studies [48, 50, 56],…"
- *Replaced the paragraph in the Conclusion section, at line 851, from*
  "Among the methods previously included in other benchmarking studies, SparCC, SPIEC-EASI, and CCLasso were evaluated in [50]. Our edge-wise results are consistent with their findings: SparCC generally exhibits lower performance than SPIEC-EASI and CCLasso, whereas the latter two often achieve comparable results. The remaining methods (SPRING, gCoda, SECOM, HARMONIES, COZINE, and FlashWeave) were not included in previous benchmarking studies. In our study, SPRING and COZINE rank first and third, respectively, while gCoda and HARMONIES fall within the top half of the rankings."
  to
  "Among the methods previously included in other benchmarking studies, SparCC, SPIEC-EASI, and CCLasso were evaluated in [50], while SPRING, gCoda, SparCC and SPIEC-EASI were evaluated in [49]. Our edge-wise results are consistent with their findings: SparCC generally exhibits lower performance than SPRING, CCLasso and SPIEC-EASI, which are consistently among the top performing methods when included in the assessment. The remaining methods (SECOM, HARMONIES, COZINE, and FlashWeave) were not included in previous benchmarking studies. In our study, COZINE ranks third and HARMONIES falls within the top half of the rankings."
- *Updated statement in the Conclusion (line 862) section in light of the newly included benchmarks from:*
  "These results suggest that more recent methods, developed specifically for microbial network inference, have achieved substantial advances since the last systematic evaluations, which primarily focused on base methods."
  to
  "These results suggest that more recent methods, developed specifically for microbial network inference, have achieved substantial advances."

Furthermore, we have added the new studies to Table 3 in the Supplementary file S3. The updated table is also reported here for reviewer convenience:

Table 2 – Comparison with previous benchmark studies. *We restricted our review to studies dedicated entirely, or predominantly, to comparing network inference methods for metagenomic data and offering a standalone, independent benchmarking analysis. We left out works where benchmarking was secondary or narrow in scope, such as papers presenting a new network inference approach or a metagenomic analysis pipeline.*

		**This work**	Berry and Widder 2014	Weiss et al. 2016	Hirano and Takemoto 2019	Brunner et al. 2024	Agyapong et al. 2025	Ghaeli et al. 2026
**General**	Data	MiCRM	gLV	Parametric distributions and gLV	gLV	Parametric distributions	Real dataset[1]	NORTA[2] and real dataset[1]
	Number of methods	18	6[†]	8	9	6	4	6
**Evaluation metrics**	Edge-wise metrics	MCC, Precision, Recall	Recall, Specificity	Recall, Specificity, Precision	Precision, Recall, cAUPR[3]	R^2, AUROC	✗	Precision, Recall, F1 score
	Node-wise metrics	$\mathcal{C}_B$, $\mathcal{C}_C$, $\mathcal{C}_E$	$p(k)$	✗	✗	$\mathcal{C}_B$, $\mathcal{C}_D$, $\mathcal{C}_E$	✗	✗
	Graph-wise metrics	δ_{KS}, $\rho(\mathcal{G})$, $\mathcal{D}$, $\mathcal{R}$, $\overline{d}$, $\overline{C}$	$\overline{k}$, $\overline{d}$, $\overline{C}$, $\overline{\mathcal{C}_B}$, $\overline{\mathcal{C}_C}$, keystoness[4]	✗	✗	community detection	MSE[5]	✗
	Computational burden	✓	✗	✗	✗	✗	✗	✗
**Parameters explicitly tested**	Topologies	ER, BA, WS	ER, BA, WS, KE[6]	✗	ER, BA, WS	✗	✗	ER, BA, banded, clustered
	Number of species	50, 100, 150, 200	From 10 to 400*	✓	From 10 to 100*	✗	✗	✗
	Number of samples	50, 75, 100, 200, 300, 500, 1000	From ∼2 to 300*	✗	From ∼10 to 500*	75, 150, 300	From 10 to 85*	✗
	Network density	2%, 5%, 8%, 10%, 15%	From ∼0% to 60%*	✗	From $\overline{k}$=2 to $\overline{k}$=20[§]*	dense/ sparse*	✗	✗
	Interaction types	$r_{neg}=0$, 0.05, 0.1, 0.2	✗	✓	random, mutualism, competition, mixed, predator-pray	positively/ negatively biased*	✗	✗
	Library size	100%, 10%, 2%, 1%, 0.5%	✗	✗	✗	✗	✗	✗
	Other	Uniform configuration of common methods' parameters, β-diversity	Intra-sample evenness (α-diversity), Inter-sample heterogeneity (β-diversity)	Different normalisa-tion approaches	✗	Inter-kingdom interactions	Cross-validation evaluation	Reproducibility assessment

[1]No ground-truth interaction network available.
[2]NORmal To Anything algorithm Nelsen 2006, used to generate synthetic data from an a priori covariance matrix and a real count matrix.
[3] Corrected area under the PR curve, calculated as (AUPR_{obs}–$\text{AUPR}_{\text{rand}}$) / (1–$\text{AUPR}_{\text{rand}}$), where AUPR_{obs} and $\text{AUPR}_{\text{rand}}$ correspond to the observed AUPR value and the AUPR value obtained from random prediction.
[4]The number of lost species in the steady state community after the removal of a specie.
[5]Mean Square Error (MSE) between the counts and the predicted values of species' counts for the samples in the test set.
[6] Klemm-Eguiluz (KE) model: generates topologies with small world and scale-free properties as well as modularity.
[†]Only base metrics: Bray-Curtis dissimilarity, Jaccard index, Mutual Information, Kendall correlation, Pearson correlation, Spearman correlation.
[*] Values not explicitly stated in the main paper. Ranges were estimated visually from the paper figures.
[§]In the paper the authors express the level of connectivity using the average degree $\overline{k}$ which can be converted to density as $\rho(\mathcal{G}) = \overline{k}/(p-1)$.

3. The manuscript would benefit from a concise fitness-for-purpose check comparing the N2SIMBA-generated count tables with representative real 16S datasets. Since the authors justify N2SIMBA as producing realistic 16S rDNA-seq data and use these simulations to derive practical method rankings, the simulated datasets should be shown to resemble real microbiome count matrices in key distributional and dependence properties.

A minimal check could include four summaries: (i) per-taxon marginal abundance distributions between real and simulated data, for example using per-OTU Kolmogorov–Smirnov tests and reporting the proportion of taxa with significant deviation; (ii) sparsity and zero-inflation structure, using per-OTU zero rates and the median absolute zero-rate error; (iii) global multivariate structure, using Bray–Curtis dissimilarities and PERMANOVA to test whether real and simulated samples are strongly separable by data source; and (iv) dependence structure, which is particularly important for a network-inference benchmark, for example using CLR-transformed Spearman correlation matrices and a Mantel-type or equivalent rank-based matrix correlation. If substantial discrepancies are observed, the authors should qualify the generality of the method rankings, clarify which aspects of real 16S data are not well captured by the current N2SIMBA simulations, and report how these discrepancies may bias the benchmark conclusions.

We thank the reviewer for this detailed suggestion and agree that the realism of simulated data is a major concern in assessing the validity of a benchmarking. However, we respectfully note a technical limitation inherent to generative simulations that prevents the execution of the specific 1:1 statistical comparisons proposed.

As the reviewer rightfully points out, the suggested metric (specifically the per-OTU Kolmogorov-Smirnov tests, the Bray-Curtis PERMANOVA, and the Mantel test for dependence structure) require a direct 1:1 correspondence between the features (taxa) in the real dataset and the simulated dataset. Because N2SIMBA generates synthetic interaction networks and corresponding abundances de novo rather than resampling or transforming an existing empirical count matrix, the i-th simulated taxa do not biologically map to specific i-th empirical OTUs. The same principle applies to other de novo simulation frameworks, including generalized Lotka–Volterra models. Consequently, aligned matrix comparisons and paired per-taxon tests are not applicable in this setting.

However, we recognize the importance of the reviewer's underlying point and the value of assessing how closely simulated data resemble real-world data. Looking for a suitable framework to assess simulated data in the context of network inference, we reviewed six previous benchmarking studies. However, none explicitly evaluated the resemblance between de-novo simulated datasets and real microbiome data.

*In the absence of an established framework for comparing simulated and empirical data for network inference analyses, we therefore assessed whether our simulated datasets reproduce several well-characterized properties of real microbiome data and qualitatively compared the resulting trends with those observed across six empirical datasets. Based on characteristics previously investigated in the literature [*Marsland et al., 2020, Grilli, 2020, Ho et al., 2022, Wilcox & López, 2026*], and the ones suggested by the reviewer, we evaluated properties related to sparsity/prevalence, variability, and abundance, as well as the relationships among these features.*

In particular we analyse:

New Fig S6: rank versus abundance, also known as species abundance curves, plot the number of species (y-axis) observed at specific abundance levels (x-axis). These curves are generally well fit by a Fisher log-series [Fisher et al., 1943; Magurran, 2005], reflecting the inflation of rare species in these ecosystems. Comparing empirical and simulated counts using these curves helps assess the realism of the simulated count distributions [Ho et al., 2022; Wilcox & López, 2026].

New Fig S7: variability versus abundance curves plot the mean abundance of each taxa against its variance. It has been shown that this curve follows Taylor's Law [Grilli, 2020] meaning that the variance of a species scales as a power-law function of the mean population.

New Fig S8: prevalence versus abundance curves display the taxa prevalence (i.e., the fraction of samples in which they are present) against their abundance. This relationship is known to be nonlinear, with more abundant taxa generally exhibiting higher prevalence [Wang et al., 2024], and it has been used in previous studies to compare simulated data with real one [Wilcox & López, 2026, Ho et al., 2022], and helps to assess if the sparsity and zero inflation structure of real datasets is captured by the simulation.

Among the 189 simulated scenarios generated in this study, we analysed these characteristics using datasets generated with the default simulation parameters: graph generation models, ER, BA, and WS; network density=5%; negative interaction rate=0.05; beta-diversity levels, low, medium, and high; number of samples=100; number of species=50; and library size fraction=100%. In addition, we varied the library size fraction ∈ [100%, 10%, 2%, 1%] as it is known to directly affect sparsity and abundance.

The full analysis is provided in a new dedicated Section 3 of Supplementary File S1, together with new Supplementary Table 4 and Supplementary Figures S6, S7, and S8.
Here is a summary of the main results of this analysis:

- *Even before considering the simulated data, the empirical datasets showed substantial variability across the analysed characteristics. All followed the expected trends in rank–abundance and abundance–variability relationships, whereas prevalence patterns differed considerably across datasets.*
- *The simulated datasets spanned abundance ranges comparable to those observed empirically and reproduced the expected decreasing rank–abundance relationship. However, simulations with low beta diversity deviated from the expected Fisher log-series fit at low abundances, suggesting that rare taxa were under-represented in that configuration.*
- *The positive relationship between mean abundance and variance was also preserved in the simulated data. Compared with the empirical datasets, the dynamic range of mean abundance was narrower, although this difference does not directly violate the assumptions underlying the evaluated network inference methods.*
- *The prevalence–abundance relationship was the most variable characteristic in both empirical and simulated datasets.Simulations with low beta diversity, and some scenarios with medium beta diversity, tended to underrepresent low-prevalence, highly sparse taxa, whereas high-beta-diversity simulations better reproduced the empirical prevalence range, although extremely sparse taxa remained underrepresented.*
- *This suggests that some simulated datasets may not fully capture scenarios dominated by taxa at very low abundance and with many zero observations. In practice, such taxa are often removed during preprocessing; when retained, however, they are likely to pose a greater challenge for network inference methods. In this respect, these simulated datasets may represent a somewhat easier inference setting than real microbiome data.*

We also discuss these results in the main manuscript in the Methods section at line 250, highlighting which characteristics of real 16S data are captured by the simulated datasets and how these similarities and differences may affect the inference methods.

> *"To assess whether the simulated data reproduce key characteristics of real microbiome data, we compared them with six representative empirical datasets (see Table S4 in S1 File) examining: species abundance curves, mean-variance curves and mean-prevalence curves. The complete comparative evaluation is provided in Section 3 of S1 File. In general, the simulated datasets successfully reproduced the fundamental characteristics of real microbiome data.*

> ***Simulations with low beta diversity, and some scenarios with medium beta diversity, tended to under-represent low-abundance, highly sparse taxa. However, the extreme zero inflation observed for some taxa in real datasets is often reduced in practice through standard prevalence filtering before network inference. When retained, such taxa can pose a substantial challenge for inference methods; accordingly, these simulated datasets may represent a somewhat simplified input for network inference benchmarking.***

Together with removing the term "realistic" when referring to the simulated count data (Revision #1), we believe that this evaluation addresses the reviewer's concerns regarding the structural validity of the datasets generated with N2SIMBA and provides readers with a balanced account of the characteristics and limitations of the simulated data.

4. The study would also benefit from a limited cross-simulator robustness analysis to test whether the reported method rankings are specific to N2SIMBA or remain stable under different data-generation mechanisms. N2SIMBA is based on a consumer-resource logic, which is useful but necessarily encodes a particular set of assumptions about how microbial interactions generate abundance patterns. Because the manuscript uses the simulated benchmark to provide practical method-ranking guidance, it would be important to know whether these rankings are robust when the synthetic data are generated under alternative ecological or statistical assumptions.

I suggest that the authors repeat a compact subset of the benchmark using at least one simulator based on a different modeling framework, for example a gLV-based simulator such as MTIST or a dFBA-based simulator such as μBialSim. The robustness check should assess not only whether the final top-ranked methods remain the same, but also whether the metric-level performance patterns are stable. For each performance metric used in the manuscript, the authors should assess whether method-level performance patterns remain stable across simulators. This could be summarized by reporting: (i) the correlation of method rankings between N2SIMBA and the alternative simulator for each metric; (ii) the overlap of the top-performing methods across simulators; (iii) methods showing large rank instability; (iv) changes in absolute metric values; and (v) whether simulator-specific differences are associated with sparsity, compositionality, library-size variation, interaction topology, or network density.

We agree that understanding how the choice of simulation model affects benchmarking results is important for assessing the robustness of the findings. However, repeating the benchmark with alternative simulation models would require substantial additional effort and falls beyond the scope of the present study, warranting dedicated future investigation. Notably, none of the six prior benchmarking studies utilized multiple count simulation frameworks to evaluate the impact of the simulator on inference performance. Moreover, there is a technical limitation: except for the number of taxa and samples, different simulation models expose different sets of tunable parameters, making it difficult or impossible to reproduce most of our simulation scenarios across alternative frameworks.
To nevertheless assess the potential impact of different simulation frameworks, we compared our results with those of six previously published benchmarking studies based on substantially different data-generation approaches, including parametric distributions, generalized Lotka–Volterra models, NORTA, and resampling of real data. This cross-study comparison provides an indirect robustness check across simulation frameworks, broadens the range of data-generation models considered, and builds on previous benchmarking efforts.
Comparing our results with these previously published studies, we observed strong and consistent agreement in the relative ranking of methods evaluated across studies. Moreover, the main trends identified in our analysis, including the effects of specific data characteristics and simulation parameters on method performance, were largely consistent with findings from previous studies that examined the same factors.

To ensure this critical point is clear to the readers, we have added a dedicated paragraph to the Conclusion section (lines 868):

> ***"In summary, comparing our results with previously published studies, relying on alternative simulation strategies, we observed strong and consistent agreement in the relative ranking of methods [49, 50]. Moreover, part of the main trends identified in our analysis, including the effects of some data characteristics on method performance [50–52] and the comparability of base methods with ad-hoc tools [48,50,56], were largely consistent with findings from previous studies that examined the same factors. This suggests that the methods' performance is stable across different data-generating mechanisms. However, dedicated studies that systematically assess how various simulation frameworks influence inferential results remain an important direction for future research."***

Moreover, we updated the Conclusion section to extend the comparison with previous studies in terms of tools and performance varying data characteristics and simulation parameters.
The list changes is also reported here for reviewer convenience:

- ***Added the newly included benchmarks in the Conclusion section when discussing the limitations of co-occurrence inference methods, line 772***
  "Consistent with previous studies [48– 52], the results show the limitations of co-occurrence methods in inferring microbial associations, …"
- ***Added a new statement discussing the effect of network topology that includes the newly included benchmarks in the Conclusion section, line 784***
  "The specific challenge of recovering scale-free (Barab´asi–Albert) network structures, in particular, is a consistently observed in many previous assessments [49–51]."
- ***Added the newly included benchmarks in the Conclusion section when discussing the effect of the number of samples, line 795***
  "Concerning specific simulation parameters, the methods showed an increase in performance as the number of samples increased (Fig 3), as expected and in agreement with previous studies [48, 50, 51, 56]."
- ***Updated a phrase in the Conclusion section at line 833***

"In general, conditional dependence methods have higher and more consistent performance, with SPRING achieving the best results (consistent with the findings of the only previous study that included this tool [49]), across both the various scenarios considered (Fig 10A) and the different evaluation metrics (Fig 10B).

- ***Added the newly included benchmarks in the Conclusion section when discussing the performance of baseline methods compared to purpose developed tools, line 835***
  "Although it is true that some base methods, coming from classical correlation statistics, have comparable results with ad-hoc tools, in some occasions, as highlighted by previous studies [48, 50, 56],…"
- ***Replaced the paragraph in the Conclusion section, at line 851, from***
  "Among the methods previously included in other benchmarking studies, SparCC, SPIEC-EASI, and CCLasso were evaluated in [50]. Our edge-wise results are consistent with their findings: SparCC generally exhibits lower performance than SPIEC-EASI and CCLasso, whereas the latter two often achieve comparable results. The remaining methods (SPRING, gCoda, SECOM, HARMONIES, COZINE, and FlashWeave) were not included in previous benchmarking studies. In our study, SPRING and COZINE rank first and third, respectively, while gCoda and HARMONIES fall within the top half of the rankings."
  to
  "Among the methods previously included in other benchmarking studies, SparCC, SPIEC-EASI, and CCLasso were evaluated in [50], while SPRING, gCoda, SparCC and SPIEC-EASI were evaluated in [49]. Our edge-wise results are consistent with their findings: SparCC generally exhibits lower performance than SPRING, CCLasso and SPIEC-EASI, which are consistently among the top performing methods when included in the assessment. The remaining methods (SECOM, HARMONIES, COZINE, and FlashWeave) were not included in previous benchmarking studies. In our study, COZINE ranks third and HARMONIES falls within the top half of the rankings."
- ***Updated statement in the Conclusion (line 862) section in light of the newly included benchmarks from:***
  "These results suggest that more recent methods, developed specifically for microbial network inference, have achieved substantial advances since the last systematic evaluations, which primarily focused on base methods."
  to
  "These results suggest that more recent methods, developed specifically for microbial network inference, have achieved substantial advances."

5. Conclusion, Page 28: "best-case scenario or an upper bound for performance";
The statement in the Conclusion that the benchmark represents a "best-case scenario" or an "upper bound for performance" should be softened. Given the reliance on a specific simulation framework and the limited engagement with more recent benchmark or validation studies, this claim appears broader than the evidence currently supports. The authors could instead describe the results as an optimistic assessment of method performance under the controlled N2SIMBA simulation settings used here, while noting that generalizability to other simulators or real 16S datasets remains to be tested.

We agree with the reviewer that our original phrasing was too definitive. We have softened this claim to ensure it does not overextend the evidence supported by our controlled simulation framework.
We have revised the paragraph in the Conclusion, at line 884, section from:

> ***"Furthermore, even if simulated data may not capture the full complexity of real datasets, we argue that the results can still be regarded as a best-case scenario or an upper bound for performance, thus still providing valuable information for users"***

to:

> ***"Furthermore, even if simulated data may not capture the full complexity of real datasets, we argue that these results provide an optimistic assessment of method performance under the controlled simulation settings used here."***

6. Page 23-24 Fig. 8 and Fig. 9 legends: The legends appear to repeat "p = 50" and may omit or confuse one of the fixed parameters. Please check and correct the fixed-parameter descriptions.

Addressed, changed on the repeating "p=50" to the missing parameter "n=100"

References

- Fisher, R. A., Corbet, A. S., & Williams, C. B. (1943). The relation between the number of species and the number of individuals in a random sample of an animal population. *The Journal of Animal Ecology*.
- Grilli, J. (2020). Macroecological laws describe variation and diversity in microbial communities. *Nature Communications*. https://doi.org/10.1038/s41467-020-18529-y
- Ho, P.-Y., Good, B. H., & Huang, K. C. (2022). Competition for fluctuating resources reproduces statistics of species abundance over time across wide-ranging microbiotas. *eLife*. https://doi.org/10.7554/eLife.75168
- Magurran, A. E. (2005). Species abundance distributions: pattern or process? *Functional Ecology*. https://doi.org/10.1111/j.0269-8463.2005.00930.x
- Marsland, R., Cui, W., & Mehta, P. (2020). A minimal model for microbial biodiversity can reproduce experimentally observed ecological patterns. *Scientific Reports*. https://doi.org/10.1038/s41598-020-60130-2
- Wang, T., Weiss, A., & You, L. (2024). A generic approach to infer community-level fitness of microbial genes. *Proceedings of the National Academy of Sciences*. https://doi.org/10.1073/pnas.2318380121
- Wilcox, C., & López, R. C. (2026). Imbalance in gut microbial interactions as a marker of health and disease. *Science*. Retrieved August 10, 2026, from https://www.science.org/doi/10.1126/science.ady1729
