# Supplementary material for: From 16S rDNA-seq count data to microbial interactions: A comprehensive benchmark of co-occurrence network inference methods
Source: PLoS Comput Biol. 2026 Oct 29;22(10):e1014898. doi: 10.1371/journal.pcbi.1014898 (PMC13750663; doi:10.1371/journal.pcbi.1014898)
Supplement: Supplementary file 5 [file pcbi.1014898.s012.pdf]

Dear Editor,

We have addressed the remaining comments raised by Reviewer #3 and revised both the main manuscript and the Supplementary Material accordingly. Revisions to the main manuscript are highlighted in red.

Specifically, we have made the following changes:

1. **Added a quantitative summary of the comparison between empirical and simulated data.** We quantified concordance between empirical and simulated data and reported the median and interquartile range across all 50 simulation replicates. These results are presented in additional text and Figure 9 in Supplementary File S1.
2. **Documented the selection of the datasets shown in Figures S6–S8.** We clarified that the five datasets were selected by random sampling using a specified seed and provided instructions for reproducing both the dataset selection and figure generation in Supplementary File S1.
3. **Expanded the documentation of the empirical datasets.** Table 4 in Supplementary File S1 now reports, for each dataset, the taxonomic level, filtering procedure, number of taxa, number of samples, literature reference, and source link.
4. **Clarified the scope and limitations of the comparison between empirical and simulated data.** We revised the Conclusion section to explain that this comparison provides a qualitative assessment of selected macroecological patterns. We also explicitly acknowledge the observed discrepancies in prevalence and rare-taxon structure.
5. **Made the cross-study ranking comparison transparent and auditable.** We added a dedicated section, "Comparison with previous benchmark studies," to Supplementary File S3, including Table 2, explanatory text, and a new Figure 1. For each previous benchmark, we specify the data-generating mechanism or empirical data source, identify the subset of methods shared with the present study, and report the performance metrics used for the comparison. We explicitly restrict the scope of these comparisons to edge-wise performance metrics. We also report the relative rankings of the overlapping methods from each benchmark and assess their agreement with our results, quantifying agreement using rank correlation where possible.
6. **Expanded the discussion of cross-study agreement and disagreement.** In the Conclusion section, we discuss the similarities and differences between our method rankings and those reported in previous studies. We emphasize that these comparisons provide only indirect evidence and do not constitute validation of the complete ranking of all 18 methods.

We have also corrected the remaining ambiguous wording, typographical errors, and other textual inaccuracies.

We thank Reviewer #3 for the constructive comments and suggestions, which have helped us improve the clarity, transparency, and rigor of the manuscript.

We hope that the revised manuscript is now suitable for publication in *PLOS Computational Biology*. Further details are provided in the accompanying point-by-point response to the reviewer.

Best regards,
Giacomo Baruzzo

Line reference refer to the manuscript with track changes

Reviewer #1

Thank you for addressing all comments and making the appropriate revisions.

Reviewer #2

The revised version substantially improves the first draft and I would recommend its publication. I thank the authors for addressing the comments raised.

We sincerely thank both reviewers for their positive and constructive feedback. We are grateful for their careful assessment of the revised manuscript and are pleased that the revisions have satisfactorily addressed their comments.

Reviewer #3

The revision addresses several of my original concerns. Notably, the authors have added comparisons against six empirical datasets, expanded their discussion of recent benchmark literature, moderated the "upper-bound" phrasing, and corrected several figure legends. I do not consider it necessary to rerun the entire benchmark using another simulator, as the authors have identified previous benchmarks based on NORTA, generalized Lotka–Volterra (gLV) models, parametric distributions, and real-data resampling. These studies could provide valuable indirect cross-generator evidence, provided that the overlapping methods and their performance are systematically compared. The remaining revisions mainly concern: (i) adding a concise numerical summary to the simulation–real-data comparison, including results across all 50 replicates; (ii) making the cross-study ranking evidence auditable; and (iii) correcting several factual, terminological, and supplementary-reference inconsistencies.

Comments

1. Quantify and qualify the simulation–real-data comparison

I appreciate the new comparisons of rank–abundance, mean–variance, and mean–prevalence relationships in S1 File. They show that the simulations reproduce several broad macroecological trends, while also revealing an under-representation of low-abundance, highly sparse taxa under some beta-diversity settings. However, the statement that the simulations "successfully reproduced the fundamental characteristics of real microbiome data" (PDF p.25, lines 243–253) is stronger than this qualitative analysis supports. Moreover, Figures S6–S8 display only five randomly selected simulations from the 50 replicates per setting.
Please: (i) explain how the five displayed simulations were selected and provide their seed or dataset identifiers; (ii) calculate a simple concordance measure for each curve class; (iii) report the median and IQR of these measures across all 50 replicates for each setting; (iv) provide the taxonomic level, filtering rules, and number of retained taxa for each empirical dataset; and (v) revise the main-text conclusion to describe the results as qualitative agreement for selected macroecological patterns while acknowledging the discrepancies in prevalence and rare-taxon structure.

We thank the reviewer for this suggestion. We have expanded the simulation vs. real-data comparison and clarified both the reproducibility of the displayed examples and the quantitative analysis.

(i) The 5 runs shown in Figures 6–8 in File S1 were selected randomly without replacement using `np.random.choice(range(50), size=5, replace=False)` after setting a fixed seed (`np.random.seed(42)`) to ensure complete reproducibility. All figures can be reproduced directly using the notebook `simulation_validation/plot_by_metric.ipynb` available in the GitLab repository. We also added a statement in Supplementary File S1 to clarify how the dataset were randomly selected.

(ii)-(iii) To quantify the agreement between simulated and empirical curves, we now compute the mean absolute error (MAE) between the two curves after interpolation onto a common x-grid over their shared numerical range:

$$MAE=\sum|y_1 - y_2| / \#points$$

We use MAE as a simple curve-discrepancy measure for the three curve classes considered in the analysis: rank–abundance, mean–variance, and mean–prevalence relationships. Lower MAE therefore indicates greater

agreement between the simulated and empirical curves. For each simulation setting and each curve class, we now report the median and interquartile range of the MAE across all 50 replicates. These results are provided in Figure 9 of Supplementary File S1. The exact interpolation procedure and the treatment of the curves prior to MAE calculation are also described in the supplementary material.

(iv) For the empirical datasets, we integrated Table 4 in Supplementary File S1 so that it now reports the taxonomic level, the filtering procedure, the number of taxa, the number of samples, the reference and the source link for each dataset. Where no additional filtering was performed, this is explicitly reported as such. The comparison therefore uses the datasets as provided by the original studies, with the processing steps and feature counts now explicitly documented.

(v) Finally, we agree that the previous wording was too broad. We have revised the corresponding statement in the main text to emphasize qualitative agreement with selected macroecological patterns while explicitly acknowledging the observed discrepancies:

> **"Overall, the simulations showed qualitative agreement with selected macroecological patterns observed in the empirical datasets, while also showing discrepancies in prevalence and the representation of rare taxa."**

2. Make the cross-study ranking comparison auditable

The Conclusion claims "strong and consistent agreement in the relative ranking of methods" and concludes that method performance is stable across data-generating mechanisms (manuscript p.23, PDF p.41, lines 847–855). The cited studies provide potentially valuable indirect evidence, but Table 2 in S3 File currently describes study designs without systematically demonstrating ranking stability.

Please extend this table to report, for each benchmark: (i) the data-generating mechanism; (ii) the exact methods overlapping with the present study; (iii) the performance metric or metric class used and its comparability with the present analysis; (iv) the relative performance or ordering of the overlapping methods; (v) whether that ordering agrees, partially agrees, or conflicts with the corresponding findings of the present study; and (vi) the scope of the comparison, such as edge-wise performance, graph-wise performance, or network reproducibility. Rank correlation or top-performing-method overlap may be reported where a numerical comparison is justified; otherwise, a transparent qualitative classification would suffice.

We thank the reviewer for this constructive suggestion. We agree that a systematic and auditable comparison of method rankings across studies is important for appropriately qualifying the cross-study conclusions.

To address the six requested aspects, we retained Table 2 in Supplementary File S3 and added a dedicated section entitled "Comparison with previous benchmark studies," including a new subsection entitled "Methods' ranking" and Figure 1. Specifically:

1. **Data-generating mechanism: Table 2 in File S3 reports the data-generating mechanism or data source considered in each benchmark study.**
2. **Overlapping methods: Table 2 in File S3 identifies the number of methods evaluated in each benchmark, while Figure 1 in File S3 reports the subsets of methods overlapping with the present study.**
3. **Performance metrics and comparability: Table 2 in File S3 reports the performance metrics used by each study and their corresponding metric class. For the cross-study ranking comparison, we focus on edge-wise performance, which is the metric class most consistently reported across the considered benchmarks. The scope and limitations of this comparison are now explicitly described in Supplementary File S3 (Section "Comparison with previous benchmark studies").**
4. **Relative ordering: Figure 1 in File S3 reports the relative ordering of the overlapping methods as derived from each previous benchmark.**
5. **Agreement with the present study: Figure 1 in File S3 explicitly shows a direct comparison of the ranking with the corresponding ordering observed in the present study. Where at least four methods overlap, and thus numerical comparison is informative, rank correlations are reported; otherwise, the comparison is qualitative and visual.**
6. **Scope of comparison: The comparison is explicitly identified as an edge-wise comparison and should not be interpreted as a comparison of the complete multi-metric, 18-method ranking (as stated in the Conclusion section line 850 and in Supplementary File S3).**

Thus, the information requested by the reviewer is distributed between Table 2 in File S3, which provides the benchmark characteristics and methodological context, and Figure 1 in File S3, which provides the auditable comparison of the overlapping method rankings.

The expanded comparison shows agreement for several subsets of overlapping methods, but also reveals divergences among studies. In particular, the comparisons involving Hirano et al. and Ghaeli et al. provide evidence of agreement among several overlapping methods despite the use of different data-generation frameworks (gLV and NORTA, respectively). We have nevertheless avoided interpreting these partial agreements as direct and explicit validation of the complete ranking obtained in the present study.

We acknowledge that differences between studies may arise from the data-generation mechanism, simulation parameters, performance metrics, and study scope. A comprehensive meta-analysis of the influence of the simulation framework on inference performance would therefore require a dedicated analysis and is beyond the scope of the present work.

The Discussion should also acknowledge disagreements reported across studies regarding beta diversity (PDF pp.39–40, lines 772–806), the number of species, and competitive interactions. Agreement among a limited subset of methods on edge-wise outcomes should not be presented as validation of the complete 18-method overall ranking. The Conclusion should characterize the available evidence as indirect and explicitly acknowledge that method rankings may vary across data-generating mechanisms.

We agree with the reviewer on the importance of these caveats. While the manuscript does not have a separate Discussion section, we address these points directly within the Conclusion. We have already explicitly acknowledged the cross-study disagreements regarding specific parameters. Specifically

- **β-diversity: "Higher β−diversity between samples improved method performance, a finding that deviates from what was previously reported [51]" at line 776**
- **Number of species: "Furthermore, we observed a drop in the methods' performance when the number of species in the community increased (Fig 5), a trend less evident in previous studies where the number of species did not considerably affect the performance [50, 51]" at line 783**
- **Competitive interactions: "Increasing the percentage of negative interactions, and thus the level of competition, significantly reduced the methods' performance (Fig 8). Our results align with [52] regarding the limitations of inferring competitive versus mutualistic interactions, but contrast with [50], which reported better performance in competitive communities." at line 803**

We have also revised the corresponding paragraph in the Conclusion (line 849 to 868) to make explicit that the cross-study comparison provides only indirect evidence and does not constitute validation of the complete 18-method ranking:

> **"In summary, when comparing our edge-wise results with previously published studies [49, 50] that relied on alternative simulation strategies, we observed substantial agreement in the partial ranking of methods (see Figure 1 in S3 File). However, we emphasize that this provides only indirect evidence and does not serve as a complete validation of our overall 18-method ranking, and that method ranking may vary across data-generation frameworks. While several main trends identified in our analysis, such as the effects of specific data characteristics [50–52] and the comparability of base methods with ad-hoc tools [48,50,56], were largely consistent with previous findings, further investigations that systematically assess how simulation framework influence inference results remain an important direction for future research**"

The classification of Brunner et al. (2024) also requires verification. The response letter describes it as using gLV-generated data, whereas Table 2 in S3 File classifies it under "Parametric distributions." Because the robustness argument depends on correctly identifying the data-generating mechanisms, please reconcile this discrepancy and reassess whether the study provides comparable ranking evidence. If it primarily assesses covariance reconstruction from parametrically generated paired compositional data, it should not be counted as gLV-based evidence, and its relevance to cross-generator ranking robustness should be characterized as indirect.

We apologise for the confusion; the response letter contained a typo. Brunner et al. (2024) uses parametric distributions to generate the synthetic data, as reported in Table 2 of the S3 File. This was only an error in the response letter; the study was already treated as a parametric-distribution-based benchmark in our cross-study analysis and in Table 2 in File S3, so no change to the analysis or classification in the manuscript was required.

Finally, the cross-study table is cited as "Table 2 in S2 File" in the Introduction and "Table 3 in S3 File" in the response

letter. Its actual location is Table 2 in S3 File. Please correct these references and use the table designation consistently throughout the revision materials.

Addressed, the citation in the Introduction was corrected to “Table 2 in S3 File”. We also verified that the cross-study benchmark table is consistently identified as “Table 2 in S3 File.”

3. Include all relevant methods from Hirano and Takemoto (2019)

The manuscript states that SparCC, SPIEC-EASI, and CCLasso were evaluated by Hirano and Takemoto (2019) (PDF p.40, lines 832–835). However, that benchmark also evaluated Pearson, Spearman, partial Pearson, and partial Spearman, which overlap with the present study. Please include these methods when assessing the consistency of the cross-study rankings.

We thank the reviewer for pointing this out. In the main text’s Conclusion section, we deliberately separated our discussion of the base methods (such as Pearson, Spearman, partial Pearson, and partial Spearman correlations) (Conclusion section line 820) from the discussion regarding the comparison of method rankings across independent studies (Conclusion section line 835).

The newly added subsection “Methods’ ranking” in Supplementary File S3 that explicitly presents a complete comparison of rankings across benchmarks, includes the study Hirano and Takemoto (2019) and a full comparison of all the methods shared with this work (Pearson, Spearman, partial Pearson, partial Spearman, SparCC, SPIEC-EASI, and CCLasso). We believe this expanded supplementary analysis satisfies the request to evaluate the consistency of these methods across studies.

4. Revise the remaining “realistic scenarios” wording

The phrase “realistic scenarios” remains in the Conclusion (PDF p.42, lines 880–885), despite the response letter stating that it had been replaced. Please use wording such as “conditions commonly encountered in empirical microbiome studies,” which does not imply that the realism of the simulated scenarios has been established.

The term “realistic scenarios” in the Conclusion (line 884) was intended to refer to actual empirical settings (e.g., where species outnumber samples), not to our simulated benchmarks. Nevertheless, we agree that absolute clarity is paramount. To ensure no reader mistakenly connects this phrase to our simulations, we have revised the manuscript to: “conditions commonly encountered in empirical microbiome studies ”

5. Several typographical, grammatical, and caption errors remain

- PDF p.21, line 94: “apriori” should be “a priori.”
- PDF p.22, line 101: “to asses their applicability” should be “to assess their applicability.”
- PDF p.23, line 157: “parameters values” should be “parameter values.”
- PDF p.25, lines 256–259: “with the addition of newly implemented methods, adding up to 13 inference tools” is repetitive and should be rewritten.
- PDF p.26, line 270: “methods performance” should be “methods’ performance.”
- PDF p.29, lines 366–367: “an estimate adjacency matrix” should be “an estimated adjacency matrix.”
- PDF p.37, lines 689–690: “methods inference ability” should be possessive, and “predator-pray” should be “predator–prey.”
- PDF p.38, Fig. 9 legend: \(K=100\%\) is incorrectly listed among the fixed parameters, although \(K\) is varied in this analysis. Please remove \(K=100\%\) from the list of fixed parameters and report \(r_{\mathrm{neg}}=0.05\) instead.
- PDF p.39, line 765: insert a space after “performance.” Line 770 contains an unnecessary article in “is a consistently observed.”
- PDF p.40, line 811: “Aitchinson” should be “Aitchison.”
- PDF p.41, Fig. 10 legend: revise “successful iterations off of the 50 simulated” and remove the duplicated word in “represent the the rank.” Line 842 should use “16S,” not “16s.”
- PDF p.41, line 873: add a comma after “Finally.”
- PDF p.42, line 883: “contained number of negative interactions” is unclear and should be replaced with “a limited proportion of negative interactions” or equivalent wording.

All of the typographical, grammatical, terminology, and figure-caption issues listed by the reviewer have been corrected
