# Supplementary material for: From 16S rDNA-seq count data to microbial interactions: A comprehensive benchmark of co-occurrence network inference methods
Source: PLoS Comput Biol. 2026 Oct 29;22(10):e1014898. doi: 10.1371/journal.pcbi.1014898 (PMC13750663; doi:10.1371/journal.pcbi.1014898)
Supplement: Supplementary file 6 [file pcbi.1014898.s010.docx]

Notes for PLOS Computational Biology manuscript:

I enjoyed reading this extensive and thorough analysis of different approaches used to infer community interaction networks. The manuscript is well written and I appreciate how clearly the authors describe each method tested, parameters considered/ controlled, and the metrics used to assess results. While I do not have the expertise to scrutinize the mathematics, I greatly appreciate the detailed explanation the authors provided for their methodology. They clearly detail how they build on prior analyses and extend the scope of methods and scenarios tested, and emphasize the limitations of every approach.

Although the authors do not advocate for any specific approach (as it does not appear feasible due to the complexity of different real-world environmental datasets), this study serves as a useful resource for anyone performing community interaction network assessments, providing a comprehensive overview of the limitations of many common approaches. Reseachers would benefit from considering these results both before planning an experiment, to inform study design, as well during data analysis, to reflect on which method to employ and the limitations of whatever model(s) is selected.

While I do find the paper to be very well written overall, I have provided specific edits for grammar (mostly minor, such as frequently missing or incorrectly placed commas), as well as a few points that require clarifying.

Please see below for specific comments/edits:

**Abstract**

Rephrase the sentence:

“To derive ecological associations many inference approaches have been developed, here we focus on co-occurrence computational methods, in which associations are inferred from metagenomic abundance data by measuring the statistical dependence between species counts across different sampled populations.”

to:

“Many inference approaches have been developed to derive ecological associations. Here, we focus on co-occurrence computational methods, in which associations are inferred from metagenomic abundance data by measuring the statistical dependence between species counts across different sampled populations.”

Remove “a” in phrase “leading to a scarce application”:

The limitations in culture-based experiments have hindered the ability to assess these methods' validity, leading to a scarce application in metagenomics studies.

**Author summary**

Rephrase: “The use of realistic simulated data was necessary due to the lack of reliable ground- truth references from culture-based experiments that has limited systematic evaluation of method performance.”

to

“The use of realistic simulated data was necessary due to the lack of reliable ground- truth references from culture-based experiments, which has limited systematic evaluation of method performance.”

**Introduction**

Line 16 – rephrase “to a level” to “to reach a level”

Line 50: add comma to “To solve part of these issues, several co-occurrence-based methods 50 have been proposed during the years.”

Lines 53-55: Rephrase to “Different metrics can be used to measure different types of associations, including Pearson Correlation (linear), Spearman Correlation (monotonic), Bray-Curtis dissimilarity, and Maximal Information Coefficient (MIC) [35] for non-linear associations.”

Line 57: NGS is written but the acronym is never identified. Please add “(NGS)” after the first mention of next generation sequencing, on line 15

Line 58: add comma after “(like 16S rDNA-seq data)”

Line 62: add comma after “To avoid this limitation”

Line 63: add comma after “SparCC [38]”

Line 64: remove comma after “expensive”

Line 67: add comma after “However”

Line 87: replace “then” with “to”

Line 89: add comma after “have criticised this approach [48]”

Line 601: be consistent in how you write “nonlinear”…sometimes written in doc as non linear or non-linear”. Change to “non-linear”

Line 109: add comma after “measures (Precision, Recall, and Matthews correlation coefficient (MCC))“

Line 115: add comma after “results”

**Materials and methods**

Line 139: remove “As” from “As in this study,”

Line 140: change comma after “graphs” to a semicolon

Line 146: add comma after “N2SIMBA [54]”

Line 153: “Where” should be lowercase

Line 157: add “and” between “in S1 File,” and “ for a detailed…”

Line 161: I may be misinterpreting, as I am unfamiliar with the mathematics employed in this study. To clarify: based on the example in the subsequent sentence, do you mean that Dαβ denotes the fraction of resource Rβ that is converted to Rα (as written in the manuscript), or does it denote the fraction of resource Rα that is converted to Rβ?

As written, “if ci,α ≠ 0,Dαβ ̸= 0,cj,β ̸= 0 then species i is beneficial to species j”, but if Dαβ truly denotes the fraction of resource Rβ that is converted to Rα, would it be more appropriate to say that species j is beneficial to species i?

Line 165: add comma after “N2SIMBA” and remove comma after “generated”

Line 166: add comma after “details”

Line 169: add comma after “known”

Line 172: change “which” to “that”

Line 173: remove “it”

Line 174: add comma after “details”

Line 184: add comma after “initialized”

Line 186: add commas after “Then” and “community”

Line 187: remove extra “the” in “…simulating the the different environmental…”

Line 243: remove comma after “[48–50],”

Line 263: add comma after “data”

Line 265: change comma after “pseudocount” to a semicolon

Line 268: change comma after “matrix Σ [63]” to a semicolon

Line 269: add comma after “Spearman correlation”

Line 270: add comma after “network”

Line 276: remove comma after “testing”

Lines 277-278: maintain consistent tone. The rest of the paragraph was in past tense, so change “is determined” to “was determined” and “is then” to “was then”. Also, add a comma after “Aij = 1”

Line 281: remove “that have been”

Line 285: add comma after “methods”

Line 286: add comma after “SECOM [46]”

Lines 288-289: rephrase to “Spearman correlation coefficient and distance correlation coefficient [66]), respectively.”

Lines 289 – 290: rephrase “For all the methods no preprocessing, like log-ratio transformations, was performed as they are methods developed” to “No preprocessing, such as log-ratio transformations, was performed for any of the methods, as they are methods developed…”

Line 292: add comma after “Proportionality”

Line 295: add comma after “matrix A”

Line 296: remove comma after “testing”

Lines 299, Figure 1 title & caption, 324: these are the only instances of the words “unconditioned” and “conditioned” appearing. Are these interchangable with “unconditional” and “conditional”? If not, please replace all 6 instances accordingly.

Figure 1 caption: add comma after “In the real interaction network”

Line 300: replace “As” with “By”

Line 316: add comma after “normality”

Line 323: change “which” to “that”

Line 324: add comma after “of the methods”

Line 339: add comma after “j)”

Line 341: add comma after “matrix **A**”

Line 345: add comma after “classification [69]”

Line 358: add comma after “i ∈ V”

Line 360: add comma after “(i,j)”

Lines 361 - 362: change “paths length” to “paths’ lengths”

Line 367: remove “it”

Line 372: remove “Eigenvector centrality” after the colon

Line 373: change comma after “neighbours” to semicolon

Line 381: rephrase to “As a summary statistic for all node-wise metrics, we computed the coefficient…”

Line 386: add comma after “measures”

Line 388: add comma after “structure”

Line 391: remove extra “the” in “expresses the the maximum…”

Line 395: add comma after “0”

Line 396: add commas after “similar” and “1”

Line 400: remove “is”

Line 403: remove “is”

Line 406: add comma after “vertex”

Line 407: change comma after “undefined” to a period and start a new sentence: “In this work, we…”

Line 416: remove period after “clusters”

Line 417: “With” should be lowercase

Line 420: add comma after “graph Mˆ”

**Results and Discussion**

Line 459: add comma after “File)”

Line 459: change “see consistently” to “consistently observe”

Line 463: change “the one with” to “yielding”

Line 474: add comma after “level” and change “methods” to “methods’”

Figure 2, Figure 3, Figure 7, Figure 8, and Figure 9 titles: change “Methods” to “Methods’”

Line 481: add comma after “gut)” and remove comma after “difficult”

Line 483: change “have” to “has”

Line 499: add comma after “one” and change “often it” to “it often”

Line 506: add comma after “Fig 3A”

Line 516: change comma after “performance)” to period and start a new sentence (remove “also,”: “Pearson and Spearman’s correlation…”

Line 517: change “lower” to “poorer”

Line 526: add comma after “SPRING (R2 = 0.43 at n = 1000)”

Line 528: add comma after “graph”

Line 529: change “at” to “to”, and add a comma after “Restricting the comparison at the 5 highest centrality nodes”

Figure 3, Figure 5, Figure 7, Figure 8, and Figure 9 captions: make “results” plural in “Here we show the result”

Line 530: change comma after “value” to semicolon

Line 531: add comma after “SPRING”

Line 533: add comma after “gains”

Line 538: add comma after “Fig. 3C”

Line 539: remove comma after “similar”

Line 541: add comma after “(Non Linear)”, and make lowercase and add dash to be “non-linear”. “(Linear)” should also be lowercase

Line 542: add comma after “0”

Line 544: add comma after “increases”

Line 547: remove commas after “sparser” and “precise”, and add comma after “graphs”

Line 554: add “us” after “allows”

Line 556: add comma after “implementations)” and add “a” after “with”

Line 557: change comma after “1s” to a period and start a new sentence: “In contrast, HARMONIES…”. Change “worse” to “worst”, and add “a” after “with”

Figure 4 caption: change “where” to “were”

Line 571: change “previously” to “before”

Line 575: add comma after “p = 50”

Line 576: add comma after “(Fig. 5A)”

Line 581: add comma after “SPRING”

Lines 586-588: rephrase. What do you mean by “if not for”?

Line 589: remove “, however,”. Sentence should read as “The only exceptions are SparCC, which has MCC =0, and SPRING.”

Line 591: change “had also” to “also had”

Line 592: add comma after “general”

Line 594: “(Linear)” should be lowercase

Line 596: change “less” to “least”

Line 600: change semicolon after “methods” to a comma

Line 601: be consistent in how you write “non-linear”…sometimes written in doc as non linear or nonlinear”. Change to “Non-linear”

Line 603: remove comma after “species”

Line 613: add comma after “methods”

Line 619: remove “from” and add a comma after “increases”

Line 620: change comma after “networks” to semicolon

Line 622: remove “in” in “(in S3 File)” and add comma after parentheses

Line 628: change comma after “S3 File)” to period and start new sentence: “Taking the mean loss in recall across methods, we obtain”

Line 629: add comma after “-0.14”, change “being” to “begin”, and change comma after “with” to period and start a new sentence: “Looking at the best…”

Line 630: add comma after “methods”

Line 642: add comma after “increases”

Line 643: add comma after “10%”

Line 645: change comma after “none” to semicolon

Line 648: add comma after “WS model”

Line 650: remove comma after “network”

Lines 650 – 652: rephrase to “The true network topology, the β-diversity between samples, and the network density did not have any major impacts on the computational burden…”

Line 659: change “infer” to “inference” and add comma after “resources”

Line 664: add comma after “Fig. 8A”

Line 666: rephrase to “Conditional dependence methods also experience…”

Line 671: remove “Also” and add “metrics” after “graph-wise”

Line 674: add comma after “Fig. 8”

Line 677: rephrase to “…dynamics and causes interactions…”

Line 678: add “to” before “cancel”, and add “-” between “3” and “species” to be “3-species”

Line 680: add comma after “magnitude”

Line 682: change “method’” to “methods’”

Line 688: rephrase to “Due to the compositional nature of sequencing count data, the role of library size has…”

Line 690: change “also” to “as well as”

Line 703: change “decreases” to “decreased”

Line 704: add comma after “Fig. 9B”

Line 707: remove “however”

**Conclusion**

Line 716: remove “also”

Line 717: change “by considering” to “to”

Line 719: change “at” to “by”

Line 720: change “level” to “metrics”

Line 724: add “network” before “density” and replace “and” with a comma to read “network density, ratio…”

Line 725: change “Consistently” to “Consistent”

Line 730: change “cautions” to “caution”

Lines 734-735: rephrase to “The methods obtained the best results with the Watts-Strogatz model (Fig. 2), as found by…”

Line 737: change “model” to “models”

Line 738: rephrase to “The β−diversity between samples also had an impact…”

Figure 10 caption: change “methods” to “methods’” in “A summary of the methods performances at various…”. Two instances of “methods ranks” should also be changed to “methods’ ranks”

Not sure if I’m misinterpreting this but in the Figure 10A image, columns corresponding to Density = 10% and = 15%, and Neg. ratio = 20% are missing a rank “3”, and columns corresponding to #Taxa = 200 and Library size = 10% are missing a rank “2”

*Note – “scalability” is not mentioned until the Conclusion section. Try to reference it at least once before, when mentioning the different metrics used to assess computational burden

Line 819: add “by” after “gaps”

Line 842: add comma after “example”

Line 851: add “underscore the fact that” after between “and” and “that” to read “…and underscore the fact that the problem…”

*throughout document – be consistent in how you denote figures. Sometimes they are referenced as “Fig #”, other times as “Fig. #”. Select one format and change all instances accordingly.

*I am not providing detailed reviews of the supplementary materials, but please take these notes into consideration and revise accordingly when appropriate (for ex., consistent writing of “non-linear”, correct punctionation, etc.).

A few examples:

- S1 File – Figure 3 caption: change “tree” to “three”
- S1 File – pg 7 – change “it” to “they” in phrase “and thus it will no longer be supplied externally…”
- S2 File – Figure 2 caption: change “pannels” to “panels”
- S3 File – Table 1 – change “non linear” to “non-linear”
- S3 File – Figure 46 – change title to “Median MCC with varying sample/taxa ratio”
