# Supplementary material for: From 16S rDNA-seq count data to microbial interactions: A comprehensive benchmark of co-occurrence network inference methods
Source: PLoS Comput Biol. 2026 Oct 29;22(10):e1014898. doi: 10.1371/journal.pcbi.1014898 (PMC13750663; doi:10.1371/journal.pcbi.1014898)
Supplement: Peer review history

# Peer review: Decision Letter 0

**DOI:** 10.1371/journal.pcbi.1014898.r001

**Authors:** Ilya Ioshikhes, Juan C. Villada

14 Jul 2026

PCOMPBIOL-D-26-01360

From 16S rDNA-seq count data to microbial interactions: a comprehensive benchmark of co-occurrence network inference methods

PLOS Computational Biology

Dear Dr. Baruzzo,

Thank you for submitting your manuscript to PLOS Computational Biology. After careful consideration, we feel that it has merit but does not fully meet PLOS Computational Biology's publication criteria as it currently stands. Therefore, we invite you to submit a revised version of the manuscript that addresses the points raised during the review process.

Please submit your revised manuscript by Aug 13 2026 11:59PM. If you will need more time than this to complete your revisions, please reply to this message or contact the journal office at ploscompbiol@plos.org. When you're ready to submit your revision, log on to https://www.editorialmanager.com/pcompbiol/ and select the 'Submissions Needing Revision' folder to locate your manuscript file.

Please include the following items when submitting your revised manuscript:

* A letter that responds to each point raised by the editor and reviewer(s). You should upload this letter as a separate file labeled 'Response to Reviewers'. This file does not need to include responses to formatting updates and technical items listed in the 'Journal Requirements' section below.

* A marked-up copy of your manuscript that highlights changes made to the original version. You should upload this as a separate file labeled 'Revised Manuscript with Track Changes'.

* An unmarked version of your revised paper without tracked changes. You should upload this as a separate file labeled 'Manuscript'.

If you would like to make changes to your financial disclosure, competing interests statement, or data availability statement, please make these updates within the submission form at the time of resubmission. Guidelines for resubmitting your figure files are available below the reviewer comments at the end of this letter.

As the corresponding author, your ORCID iD is verified in the submission system and will appear in the published article. PLOS supports the use of ORCID, and we encourage all coauthors to register for an ORCID iD and use it as well. Please encourage your coauthors to verify their ORCID iD within the submission system before final acceptance, as unverified ORCID iDs will not appear in the published article. Only the individual author can complete the verification step; PLOS staff cannot verify ORCID iDs on behalf of authors.

We look forward to receiving your revised manuscript.

Kind regards,

Juan C. Villada, Ph.D.

Guest Editor

PLOS Computational Biology

Ilya Ioshikhes

Section Editor

PLOS Computational Biology

**Additional Editor Comments (if provided):**

**Journal Requirements:**

If the reviewer comments include a recommendation to cite specific previously published works, please review and evaluate these publications to determine whether they are relevant and should be cited. There is no requirement to cite these works unless the editor has indicated otherwise.

1) We ask that a manuscript source file is provided at Revision. Please upload your manuscript file as a .doc, .docx, .rtf or .tex. If you are providing a .tex file, please upload it under the item type u2018LaTeX Source Fileu2019 and leave your .pdf version as the item type u2018Manuscriptu2019.

2) Please upload all main figures as separate Figure files in .tif or .eps format. For more information about how to convert and format your figure files please see our guidelines:

https://journals.plos.org/ploscompbiol/s/figures

3) Please upload a copy of Figures FIG 1, 2, 3A-C, 4, 5A-C, 6, 7A-C, 8AB, 9AB, and 10AB which you refer to in your text on pages 9, 14, 16, 18, 19, 20, 21, 23, 24, and 28. Or, if the figure is no longer to be included as part of the submission please remove all reference to it within the text.

4) Please amend your detailed Financial Disclosure statement. This is published with the article. It must therefore be completed in full sentences and contain the exact wording you wish to be published.

i) State what role the funders took in the study. If the funders had no role in your study, please state: "The funders had no role in study design, data collection and analysis, decision to publish, or preparation of the manuscript."

ii) If any authors received a salary from any of your funders, please state which authors and which funders..

If you did not receive any funding for this study, please simply state: u201cThe authors received no specific funding for this work.u201d.

**Reviewers' comments:**

Reviewer's Responses to Questions

**Comments to the Authors:**

**Please note here if the review is uploaded as an attachment.**

Reviewer #1: Please see attachment for notes

Reviewer #2: This paper focus on a difficult task in microbiome analysis, such as the identification of microbial interactions, that while addressed by a plethora of tools, remains difficult to evaluate for the limited availability of experimentally validated "ground truth" datasets.

The authors produced the largest benchmark to date, evaluating 18 approaches across 189 parameters (and 50 replicats). The simulations wereddone using N2SIMBA, which is built on a consumer-resource model, which should avoid baking a covariance structure directly into the ground truth.

The analysis found the methods work only in a narrow and often unrealistic scenario, when many samples (N >> P) a good library size and low density and limited negative interactions are dominating the picture.

Interestingly, the network density results being a key factor that every method fails to successfully deal with.

Noteworthy is the finding that higher beta diversity, contrary to previous reports, helps the methods.

I would like to thank the authors for the efforts done in making this study reproducible, with a Zenodo dataset, GitLab repository and singularity containers.

I recommend the publication of this paper with some suggestions to improve the overall clarity

CLARIFICATIONS

--------------

1) Is the "ground truth" the right thing to grade against?

The authors build their simulated data with N2SIMBA. They start from a network A and from that they construct the matrix C and D, which say who eats which resource and how resources get converted. So A is the starting point, and then the metrics is how well a method did recover A?

A describes a mechanistic, resource-mediated interaction, while the methods only see the counts X and can measure statistical patterns in these counts. Some confounding factors can be if species 1 and species 2 both feed on the same by-produc of species C.

Similarly as the authors demonstrate, real interaction can disappear (positive and negative interactions will cancel each other.)

2) Sensitivity setting

Every method has defaults like the stability cutoff of SPIC-EASY and SPRING and penalty lambda in lasso based methods. The authors only used the package defaults (and the ability of the authors to define a good default, or a default that performs well in this simulation).

Methods that were found having a low recall might be methods offering a weak default threshold.

Could it be useful to sweep the cutoffs with a threshold-free metric like AUPRC?

3) Treating failed runs

Failed runs shouldn't be scored as bad accuracy, i.e. "the method ran and got the wrong answer" and "the method never ran at all" are two very different things, but the ranking mixes them together.

I would separate these and also add clarifications on why some methods didnt run to see if it's likely a package insrinsinc limitation or environmental (our HPC does not support X).

MINOR POINTS TO REVIEW

----------------------

1) Terminology: I would consider using 'association network' rather than 'interaction network' when referring to the output of cross-sectional co-occurrence methods, and reserve interaction for the simulator's mechanistic reference.

2) Recall forumla is printed TP/(TP+FP), while it should be TP/(TP+FN)

3) In the Node-wise metrics section the author write that as a summary statistics they compute "the coefficient of the determination in R2" R2=∑i(Ci−Cˉ)2∑i(C^i−Cˉ)2, while R2 is usually 1-∑i(Ci−Cˉ)2∑i(Ci−C^i)2, where in the numerator we measure the errors between true and inferred values. A further hint is that a coefficient of determination can go negative while the paper reports positive numbers, that might be a squared Pearson correlation?

4) Turning off internal filtering to equalize the input is reasonable for comparability, but it removes methods ( SECOM) from their intended pipeline, and the discussion rightly flags this for SECOM. I'd foreground this trade-off earlier.

Reviewer #3: This manuscript presents a large benchmark of 18 co-occurrence network inference methods for 16S rDNA-seq count data, using N2SIMBA/MiCRM simulations with known interaction networks to compare edge-level accuracy, topology-level behavior, and computational performance across a wide range of simulated scenarios. The study is valuable because method choice in microbial association analysis remains difficult, and the authors make a serious attempt to evaluate many commonly used tools under controlled conditions. My main concerns focus on whether the reported method rankings can be generalized beyond the specific synthetic design space. The manuscript should more clearly qualify its practical recommendations, provide a minimal fitness-for-purpose comparison between simulated and real 16S count tables, and assess whether the main rankings are stable under alternative data-generating processes or perturbations.

Major Comments:

1. Abstract, Manuscript Page 1: “valid results only in a restricted ... more realistic scenarios”; Author summary, Page 2: “realistic simulated data ... general applicability”; Results and Discussion, Page 14, lines 441-447: “generated realistic 16S datasets ... default simulator parameters”; Results and Discussion, Page 19, lines 601-604: “more realistic scenarios ... increasing the number of species.”

The manuscript’s broad practical conclusion remains stronger than the validation basis supports. The concern is not the use of simulation itself, which is a reasonable way to obtain known ground truth, but the repeated use of “realistic” language around simulated scenarios. The abstract first states that the benchmark uses synthetic 16S count data, but then frames the main conclusion as a decline in “more realistic scenarios”; the author summary describes “realistic simulated data” as supporting general applicability; and the Results/Discussion again calls simulator-generated datasets or scenarios realistic. Because true empirical microbiome data generally lack experimentally verified ground-truth ecological networks, the benchmark does not solve the core unverifiability problem for real datasets. It evaluates methods under synthetic, 16S-like count-generating assumptions. The authors should revise the abstract, discussion, and conclusion to distinguish real empirical validation from more realistic simulated scenarios, avoid implying that real-data performance has been established, and make practical recommendations conditional on simulator realism and transferability from synthetic to empirical data.

2. Introduction, Page 4, lines 98-100: “comprehensive benchmark ... with respect to previous comparative studies [48-50]”; Introduction, Page 4, lines 101-105: “largest selection ... broad range of scenarios”; Materials and methods, Manuscript Page 7: “standalone inference methods ... selected from previous comparative studies.”

The authors use three previous comparative studies in the Introduction to substantiate the breadth of method coverage and to motivate the selection of methods included in the benchmark. However, the three comparative studies used as the main benchmark anchors appear to date mainly from 2014–2019. Given the rapid development of microbial co-occurrence and association-network benchmarking, this literature base may no longer be sufficient to support the manuscript’s claim of comprehensive methodological comparison. I suggest that the authors expand this section by explicitly including more recent benchmark or validation studies in the co-occurrence network field, such as work on the error introduced by combining compositional data sets in covariance network reconstruction, as well as studies examining the difficulty of inferring microbial community structure from co-occurrence network approaches. The authors should provide a concise and up-to-date table of relevant comparative or validation studies, indicating which methods, simulation scenarios, compositionality issues, and network-inference assumptions each study addressed. They should then clarify how these more recent studies informed the present benchmark design, method selection, performance metrics, and interpretation, or explain why particular studies or frameworks were excluded.

3. The manuscript would benefit from a concise fitness-for-purpose check comparing the N2SIMBA-generated count tables with representative real 16S datasets. Since the authors justify N2SIMBA as producing realistic 16S rDNA-seq data and use these simulations to derive practical method rankings, the simulated datasets should be shown to resemble real microbiome count matrices in key distributional and dependence properties.

A minimal check could include four summaries: (i) per-taxon marginal abundance distributions between real and simulated data, for example using per-OTU Kolmogorov–Smirnov tests and reporting the proportion of taxa with significant deviation; (ii) sparsity and zero-inflation structure, using per-OTU zero rates and the median absolute zero-rate error; (iii) global multivariate structure, using Bray–Curtis dissimilarities and PERMANOVA to test whether real and simulated samples are strongly separable by data source; and (iv) dependence structure, which is particularly important for a network-inference benchmark, for example using CLR-transformed Spearman correlation matrices and a Mantel-type or equivalent rank-based matrix correlation. If substantial discrepancies are observed, the authors should qualify the generality of the method rankings, clarify which aspects of real 16S data are not well captured by the current N2SIMBA simulations, and report how these discrepancies may bias the benchmark conclusions.

4. The study would also benefit from a limited cross-simulator robustness analysis to test whether the reported method rankings are specific to N2SIMBA or remain stable under different data-generation mechanisms. N2SIMBA is based on a consumer-resource logic, which is useful but necessarily encodes a particular set of assumptions about how microbial interactions generate abundance patterns. Because the manuscript uses the simulated benchmark to provide practical method-ranking guidance, it would be important to know whether these rankings are robust when the synthetic data are generated under alternative ecological or statistical assumptions.

I suggest that the authors repeat a compact subset of the benchmark using at least one simulator based on a different modeling framework, for example a gLV-based simulator such as MTIST or a dFBA-based simulator such as μBialSim. The robustness check should assess not only whether the final top-ranked methods remain the same, but also whether the metric-level performance patterns are stable. For each performance metric used in the manuscript, the authors should assess whether method-level performance patterns remain stable across simulators. This could be summarized by reporting: (i) the correlation of method rankings between N2SIMBA and the alternative simulator for each metric; (ii) the overlap of the top-performing methods across simulators; (iii) methods showing large rank instability; (iv) changes in absolute metric values; and (v) whether simulator-specific differences are associated with sparsity, compositionality, library-size variation, interaction topology, or network density.

5. Conclusion, Page 28: “best-case scenario or an upper bound for performance”;

The statement in the Conclusion that the benchmark represents a “best-case scenario” or an “upper bound for performance” should be softened. Given the reliance on a specific simulation framework and the limited engagement with more recent benchmark or validation studies, this claim appears broader than the evidence currently supports. The authors could instead describe the results as an optimistic assessment of method performance under the controlled N2SIMBA simulation settings used here, while noting that generalizability to other simulators or real 16S datasets remains to be tested.

6. Page 23-24 Fig. 8 and Fig. 9 legends: The legends appear to repeat “p = 50” and may omit or confuse one of the fixed parameters. Please check and correct the fixed-parameter descriptions.

**********

**Have the authors made all data and (if applicable) computational code underlying the findings in their manuscript fully available?**

The PLOS Data policy requires authors to make all data and code underlying the findings described in their manuscript fully available without restriction, with rare exception (please refer to the Data Availability Statement in the manuscript PDF file). The data and code should be provided as part of the manuscript or its supporting information, or deposited to a public repository. For example, in addition to summary statistics, the data points behind means, medians and variance measures should be available. If there are restrictions on publicly sharing data or code —e.g. participant privacy or use of data from a third party—those must be specified.

Reviewer #1: Yes

Reviewer #2: Yes

Reviewer #3: None

**********

PLOS authors have the option to publish the peer review history of their article (what does this mean?). If published, this will include your full peer review and any attached files.

If you choose “no”, your identity will remain anonymous but your review may still be made public.

**Do you want your identity to be public for this peer review?**  For information about this choice, including consent withdrawal, please see our Privacy Policy.

Reviewer #1: **Yes:**  Daniela Tizabi

Reviewer #2: No

Reviewer #3: No

[NOTE: If reviewer comments were submitted as an attachment file, they will be attached to this email and accessible via the submission site. Please log into your account, locate the manuscript record, and check for the action link "View Attachments". If this link does not appear, there are no attachment files.]

**Figure resubmission:**

**Reproducibility:**

To enhance the reproducibility of your results, we recommend that authors of applicable studies deposit laboratory protocols in protocols.io, where a protocol can be assigned its own identifier (DOI) such that it can be cited independently in the future. Additionally, PLOS ONE offers an option to publish peer-reviewed clinical study protocols. Read more information on sharing protocols at https://plos.org/protocols?utm_medium=editorial-email&utm_source=authorletters&utm_campaign=protocols

Attachment Submitted filename: PCOMPBIOL-D-26-01360_reviewer_notes.docx

# Author comment: Author response to Decision Letter 1

**DOI:** 10.1371/journal.pcbi.1014898.r002

13 Aug 2026

Attachment Submitted filename: Response to Reviewers.pdf

# Peer review: Decision Letter 1

**DOI:** 10.1371/journal.pcbi.1014898.r003

**Authors:** Ilya Ioshikhes, Juan C. Villada

16 Sep 2026

PCOMPBIOL-D-26-01360R1

From 16S rDNA-seq count data to microbial interactions: a comprehensive benchmark of co-occurrence network inference methods

PLOS Computational Biology

Dear Dr. Baruzzo,

Thank you for submitting your manuscript to PLOS Computational Biology. After careful consideration, we feel that it has merit but does not fully meet PLOS Computational Biology's publication criteria as it currently stands. Therefore, we invite you to submit a revised version of the manuscript that addresses the points raised during the review process.

Please submit your revised manuscript by Oct 16 2026 11:59PM. If you will need more time than this to complete your revisions, please reply to this message or contact the journal office at ploscompbiol@plos.org. When you're ready to submit your revision, log on to https://www.editorialmanager.com/pcompbiol/ and select the 'Submissions Needing Revision' folder to locate your manuscript file.

Please include the following items when submitting your revised manuscript:

* A letter that responds to each point raised by the editor and reviewer(s). You should upload this letter as a separate file labeled 'Response to Reviewers'. This file does not need to include responses to formatting updates and technical items listed in the 'Journal Requirements' section below.

* A marked-up copy of your manuscript that highlights changes made to the original version. You should upload this as a separate file labeled 'Revised Manuscript with Track Changes'.

* An unmarked version of your revised paper without tracked changes. You should upload this as a separate file labeled 'Manuscript'.

If you would like to make changes to your financial disclosure, competing interests statement, or data availability statement, please make these updates within the submission form at the time of resubmission. Guidelines for resubmitting your figure files are available below the reviewer comments at the end of this letter.

As the corresponding author, your ORCID iD is verified in the submission system and will appear in the published article. PLOS supports the use of ORCID, and we encourage all coauthors to register for an ORCID iD and use it as well. Please encourage your coauthors to verify their ORCID iD within the submission system before final acceptance, as unverified ORCID iDs will not appear in the published article. Only the individual author can complete the verification step; PLOS staff cannot verify ORCID iDs on behalf of authors.

We look forward to receiving your revised manuscript.

Kind regards,

Juan C. Villada, Ph.D.

Guest Editor

PLOS Computational Biology

Ilya Ioshikhes

Section Editor

PLOS Computational Biology

**Journal Requirements:**

If the reviewer comments include a recommendation to cite specific previously published works, please review and evaluate these publications to determine whether they are relevant and should be cited. There is no requirement to cite these works unless the editor has indicated otherwise.

1) Please amend your detailed Financial Disclosure statement. This is published with the article. It must therefore be completed in full sentences and contain the exact wording you wish to be published.

i) Please clarify all sources of financial support for your study. List the grants, grant numbers, and organizations that funded your study, including funding received from your institution. Please note that suppliers of material support, including research materials, should be recognized in the Acknowledgements section rather than in the Financial Disclosure

ii) State the initials, alongside each funding source, of each author to receive each grant. For example: "This work was supported by the National Institutes of Health (####### to AM; ###### to CJ) and the National Science Foundation (###### to AM)."

iii) State what role the funders took in the study. If the funders had no role in your study, please state: "The funders had no role in study design, data collection and analysis, decision to publish, or preparation of the manuscript."

iv) If any authors received a salary from any of your funders, please state which authors and which funders..

If you did not receive any funding for this study, please simply state: u201cThe authors received no specific funding for this work.u201d

**Reviewers' comments:**

Reviewer's Responses to Questions

**Comments to the Authors:**

**Please note here if the review is uploaded as an attachment.**

Reviewer #1: Thank you for addressing all comments and making the appropriate revisions.

Reviewer #2: The revised version substantially improves the first draft and I would recommend its publication.

I thank the authors for addressing the comments raised.

Reviewer #3: The revision addresses several of my original concerns. Notably, the authors have added comparisons against six empirical datasets, expanded their discussion of recent benchmark literature, moderated the “upper-bound” phrasing, and corrected several figure legends. I do not consider it necessary to rerun the entire benchmark using another simulator, as the authors have identified previous benchmarks based on NORTA, generalized Lotka–Volterra (gLV) models, parametric distributions, and real-data resampling. These studies could provide valuable indirect cross-generator evidence, provided that the overlapping methods and their performance are systematically compared. The remaining revisions mainly concern: (i) adding a concise numerical summary to the simulation–real-data comparison, including results across all 50 replicates; (ii) making the cross-study ranking evidence auditable; and (iii) correcting several factual, terminological, and supplementary-reference inconsistencies.

Comments

1. Quantify and qualify the simulation–real-data comparison

I appreciate the new comparisons of rank–abundance, mean–variance, and mean–prevalence relationships in S1 File. They show that the simulations reproduce several broad macroecological trends, while also revealing an under-representation of low-abundance, highly sparse taxa under some beta-diversity settings. However, the statement that the simulations “successfully reproduced the fundamental characteristics of real microbiome data” (PDF p.25, lines 243–253) is stronger than this qualitative analysis supports. Moreover, Figures S6–S8 display only five randomly selected simulations from the 50 replicates per setting.

Please: (i) explain how the five displayed simulations were selected and provide their seed or dataset identifiers; (ii) calculate a simple concordance measure for each curve class; (iii) report the median and IQR of these measures across all 50 replicates for each setting; (iv) provide the taxonomic level, filtering rules, and number of retained taxa for each empirical dataset; and (v) revise the main-text conclusion to describe the results as qualitative agreement for selected macroecological patterns while acknowledging the discrepancies in prevalence and rare-taxon structure.

2. Make the cross-study ranking comparison auditable

The Conclusion claims “strong and consistent agreement in the relative ranking of methods” and concludes that method performance is stable across data-generating mechanisms (manuscript p.23, PDF p.41, lines 847–855). The cited studies provide potentially valuable indirect evidence, but Table 2 in S3 File currently describes study designs without systematically demonstrating ranking stability.

Please extend this table to report, for each benchmark: (i) the data-generating mechanism; (ii) the exact methods overlapping with the present study; (iii) the performance metric or metric class used and its comparability with the present analysis; (iv) the relative performance or ordering of the overlapping methods; (v) whether that ordering agrees, partially agrees, or conflicts with the corresponding findings of the present study; and (vi) the scope of the comparison, such as edge-wise performance, graph-wise performance, or network reproducibility. Rank correlation or top-performing-method overlap may be reported where a numerical comparison is justified; otherwise, a transparent qualitative classification would suffice.

The Discussion should also acknowledge disagreements reported across studies regarding beta diversity (PDF pp.39–40, lines 772–806), the number of species, and competitive interactions. Agreement among a limited subset of methods on edge-wise outcomes should not be presented as validation of the complete 18-method overall ranking. The Conclusion should characterize the available evidence as indirect and explicitly acknowledge that method rankings may vary across data-generating mechanisms.

The classification of Brunner et al. (2024) also requires verification. The response letter describes it as using gLV-generated data, whereas Table 2 in S3 File classifies it under “Parametric distributions.” Because the robustness argument depends on correctly identifying the data-generating mechanisms, please reconcile this discrepancy and reassess whether the study provides comparable ranking evidence. If it primarily assesses covariance reconstruction from parametrically generated paired compositional data, it should not be counted as gLV-based evidence, and its relevance to cross-generator ranking robustness should be characterized as indirect.

Finally, the cross-study table is cited as “Table 2 in S2 File” in the Introduction and “Table 3 in S3 File” in the response letter. Its actual location is Table 2 in S3 File. Please correct these references and use the table designation consistently throughout the revision materials.

3. Include all relevant methods from Hirano and Takemoto (2019)

The manuscript states that SparCC, SPIEC-EASI, and CCLasso were evaluated by Hirano and Takemoto (2019) (PDF p.40, lines 832–835). However, that benchmark also evaluated Pearson, Spearman, partial Pearson, and partial Spearman, which overlap with the present study. Please include these methods when assessing the consistency of the cross-study rankings.

4. Revise the remaining “realistic scenarios” wording

The phrase “realistic scenarios” remains in the Conclusion (PDF p.42, lines 880–885), despite the response letter stating that it had been replaced. Please use wording such as “conditions commonly encountered in empirical microbiome studies,” which does not imply that the realism of the simulated scenarios has been established.

5. Several typographical, grammatical, and caption errors remain

- PDF p.21, line 94: “apriori” should be “a priori.”

- PDF p.22, line 101: “to asses their applicability” should be “to assess their applicability.”

- PDF p.23, line 157: “parameters values” should be “parameter values.”

- PDF p.25, lines 256–259: “with the addition of newly implemented methods, adding up to 13 inference tools” is repetitive and should be rewritten.

- PDF p.26, line 270: “methods performance” should be “methods’ performance.”

- PDF p.29, lines 366–367: “an estimate adjacency matrix” should be “an estimated adjacency matrix.”

- PDF p.37, lines 689–690: “methods inference ability” should be possessive, and “predator-pray” should be “predator–prey.”

- PDF p.38, Fig. 9 legend: \(K=100\%\) is incorrectly listed among the fixed parameters, although \(K\) is varied in this analysis. Please remove \(K=100\%\) from the list of fixed parameters and report \(r_{\mathrm{neg}}=0.05\) instead.

- PDF p.39, line 765: insert a space after “performance.” Line 770 contains an unnecessary article in “is a consistently observed.”

- PDF p.40, line 811: “Aitchinson” should be “Aitchison.”

- PDF p.41, Fig. 10 legend: revise “successful iterations off of the 50 simulated” and remove the duplicated word in “represent the the rank.” Line 842 should use “16S,” not “16s.”

- PDF p.41, line 873: add a comma after “Finally.”

- PDF p.42, line 883: “contained number of negative interactions” is unclear and should be replaced with “a limited proportion of negative interactions” or equivalent wording.

**********

**Have the authors made all data and (if applicable) computational code underlying the findings in their manuscript fully available?**

The PLOS Data policy requires authors to make all data and code underlying the findings described in their manuscript fully available without restriction, with rare exception (please refer to the Data Availability Statement in the manuscript PDF file). The data and code should be provided as part of the manuscript or its supporting information, or deposited to a public repository. For example, in addition to summary statistics, the data points behind means, medians and variance measures should be available. If there are restrictions on publicly sharing data or code —e.g. participant privacy or use of data from a third party—those must be specified.

Reviewer #1: Yes

Reviewer #2: Yes

Reviewer #3: None

**********

PLOS authors have the option to publish the peer review history of their article (what does this mean?). If published, this will include your full peer review and any attached files.

If you choose “no”, your identity will remain anonymous but your review may still be made public.

**Do you want your identity to be public for this peer review?**  For information about this choice, including consent withdrawal, please see our Privacy Policy.

Reviewer #1: No

Reviewer #2: No

Reviewer #3: No

[NOTE: If reviewer comments were submitted as an attachment file, they will be attached to this email and accessible via the submission site. Please log into your account, locate the manuscript record, and check for the action link "View Attachments". If this link does not appear, there are no attachment files.]

**Figure resubmission:**

**Reproducibility:**

To enhance the reproducibility of your results, we recommend that authors of applicable studies deposit laboratory protocols in protocols.io, where a protocol can be assigned its own identifier (DOI) such that it can be cited independently in the future. Additionally, PLOS ONE offers an option to publish peer-reviewed clinical study protocols. Read more information on sharing protocols at https://plos.org/protocols?utm_medium=editorial-email&utm_source=authorletters&utm_campaign=protocols

# Author comment: Author response to Decision Letter 2

**DOI:** 10.1371/journal.pcbi.1014898.r004

25 Sep 2026

Attachment Submitted filename: Response_to_Reviewer_Letter.pdf

# Peer review: Decision Letter 2

**DOI:** 10.1371/journal.pcbi.1014898.r005

**Authors:** Ilya Ioshikhes, Juan C. Villada

13 Oct 2026

Dear Dr. Baruzzo,

We are pleased to inform you that your manuscript 'From 16S rDNA-seq count data to microbial interactions: a comprehensive benchmark of co-occurrence network inference methods' has been provisionally accepted for publication in PLOS Computational Biology.

Before your manuscript can be formally accepted you will need to complete some formatting changes, which you will receive in a follow up email. A member of our team will be in touch with a set of requests.

Please note that your manuscript will not be scheduled for publication until you have made the required changes, so a swift response is appreciated.

IMPORTANT: The editorial review process is now complete. PLOS will only permit corrections to spelling, formatting or significant scientific errors from this point onwards. Requests for major changes, or any which affect the scientific understanding of your work, will cause delays to the publication date of your manuscript.

Should you, your institution's press office or the journal office choose to press release your paper, you will automatically be opted out of early publication. We ask that you notify us now if you or your institution is planning to press release the article. All press must be co-ordinated with PLOS.

Thank you again for supporting Open Access publishing; we are looking forward to publishing your work in PLOS Computational Biology.

Best regards,

Juan C. Villada, Ph.D.

Guest Editor

PLOS Computational Biology

Ilya Ioshikhes

Section Editor

PLOS Computational Biology

***********************************************************

Reviewer's Responses to Questions

**Comments to the Authors:**

**Please note here if the review is uploaded as an attachment.**

Reviewer #3: I thank the authors for their careful responses and the further revisions. The revised manuscript now identifies the specific cross-study agreements and appropriately limits their interpretation to indirect evidence concerning the edge-wise performance of overlapping methods, rather than validation of the complete 18-method ranking. The authors also explicitly acknowledge the remaining discrepancies between simulated and empirical data, particularly the under-representation of rare, highly sparse taxa, and explain that these differences may simplify the inference task.

These clarifications and the corresponding moderation of the conclusions satisfactorily address my remaining concerns.

A few minor typographical and grammatical errors remain and can be corrected during final proofreading: “predator-pray” should be “predator–prey” (line 692); “simulation framework influence” should be “simulation frameworks influence” (line 857); “Evaluation of methods performance” should be “Evaluation of method performance” (line 346); and “the inference methods test” should be “the inference methods tested” (line 907). Please also check the grammar and possessives in the Supporting Information descriptions.

**********

**Have the authors made all data and (if applicable) computational code underlying the findings in their manuscript fully available?**

The PLOS Data policy requires authors to make all data and code underlying the findings described in their manuscript fully available without restriction, with rare exception (please refer to the Data Availability Statement in the manuscript PDF file). The data and code should be provided as part of the manuscript or its supporting information, or deposited to a public repository. For example, in addition to summary statistics, the data points behind means, medians and variance measures should be available. If there are restrictions on publicly sharing data or code —e.g. participant privacy or use of data from a third party—those must be specified.

Reviewer #3: None

**********

PLOS authors have the option to publish the peer review history of their article (what does this mean?). If published, this will include your full peer review and any attached files.

If you choose “no”, your identity will remain anonymous but your review may still be made public.

**Do you want your identity to be public for this peer review?**  For information about this choice, including consent withdrawal, please see our Privacy Policy.

Reviewer #3: No

# Editor report: Acceptance letter

**DOI:** 10.1371/journal.pcbi.1014898.r006

**Authors:** Ilya Ioshikhes, Juan C. Villada

PCOMPBIOL-D-26-01360R2

From 16S rDNA-seq count data to microbial interactions: a comprehensive benchmark of co-occurrence network inference methods

Dear Dr Baruzzo,

I am pleased to inform you that your manuscript has been formally accepted for publication in PLOS Computational Biology. Your manuscript is now with our production department and you will be notified of the publication date in due course.

The corresponding author will soon be receiving a typeset proof for review, to ensure errors have not been introduced during production. Please review the PDF proof of your manuscript carefully, as this is the last chance to correct any errors. Please note that major changes, or those which affect the scientific understanding of the work, will likely cause delays to the publication date of your manuscript.

Soon after your final files are uploaded, unless you have opted out, the early version of your manuscript will be published online. The date of the early version will be your article's publication date. The final article will be published to the same URL, and all versions of the paper will be accessible to readers.

For Research, Software, and Methods articles, you will receive an invoice from PLOS for your publication fee after your manuscript has reached the completed accept phase. If you receive an email requesting payment before acceptance or for any other service, this may be a phishing scheme. Learn how to identify phishing emails and protect your accounts at https://explore.plos.org/phishing.

Thank you again for supporting PLOS Computational Biology and open-access publishing. We are looking forward to publishing your work!

With kind regards,

Janani Seenivasan

PLOS Computational Biology | Carlyle House, Carlyle Road, Cambridge CB4 3DN | United Kingdom ploscompbiol@plos.org | Phone +44 (0) 1223-442824 | ploscompbiol.org | @PLOSCompBiol
